# Subnational mapping of HIV incidence and mortality among individuals aged 15–49 years in sub-Saharan Africa, 2000–18: a modelling study

**DOI:** 10.1016/S2352-3018(21)00051-5

**Published:** 2021-06-01

**Authors:** Local Burden of Disease HIV Collaborators, Benn Sartorius, John D VanderHeide, Mingyou Yang, Erik A Goosmann, Julia Hon, Emily Haeuser, Michael A Cork, Samantha Perkins, Deepa Jahagirdar, Lauren E Schaeffer, Audrey L Serfes, Kate E LeGrand, Hedayat Abbastabar, Zeleke Hailemariam Abebo, Akine Eshete Abosetugn, Eman Abu-Gharbieh, Manfred Mario Kokou Accrombessi, Oladimeji M Adebayo, Adeyinka Emmanuel Adegbosin, Victor Adekanmbi, Olatunji O Adetokunboh, Daniel Adedayo Adeyinka, Bright Opoku Ahinkorah, Keivan Ahmadi, Muktar Beshir Ahmed, Yonas Akalu, Oluwaseun Oladapo Akinyemi, Rufus Olusola Akinyemi, Addis Aklilu, Chisom Joyqueenet Akunna, Fares Alahdab, Ziyad Al-Aly, Noore Alam, Alehegn Aderaw Alamneh, Turki M Alanzi, Biresaw Wassihun Alemu, Robert Kaba Alhassan, Tilahun Ali, Vahid Alipour, Saeed Amini, Robert Ancuceanu, Fereshteh Ansari, Zelalem Alamrew Anteneh, Davood Anvari, Razique Anwer, Seth Christopher Yaw Appiah, Jalal Arabloo, Mulusew A Asemahagn, Mohammad Asghari Jafarabadi, Wondwossen Niguse Asmare, Desta Debalkie Atnafu, Maha Moh'd Wahbi Atout, Alok Atreya, Marcel Ausloos, Atalel Fentahun Awedew, Beatriz Paulina Ayala Quintanilla, Martin Amogre Ayanore, Yared Asmare Aynalem, Muluken Altaye Ayza, Samad Azari, Zelalem Nigussie Azene, Zaheer-Ud-Din Babar, Atif Amin Baig, Senthilkumar Balakrishnan, Maciej Banach, Till Winfried Bärnighausen, Sanjay Basu, Mohsen Bayati, Neeraj Bedi, Tariku Tesfaye Bekuma, Woldesellassie M Mequanint Bezabhe, Akshaya Srikanth Bhagavathula, Pankaj Bhardwaj, Krittika Bhattacharyya, Zulfiqar A Bhutta, Sadia Bibi, Boris Bikbov, Tsegaye Adane Birhan, Zebenay Workneh Bitew, Moses John Bockarie, Archith Boloor, Oliver J Brady, Nicola Luigi Bragazzi, Andrey Nikolaevich Briko, Nikolay Ivanovich Briko, Sharath Burugina Nagaraja, Zahid A Butt, Rosario Cárdenas, Felix Carvalho, Jaykaran Charan, Souranshu Chatterjee, Soosanna Kumary Chattu, Vijay Kumar Chattu, Mohiuddin Ahsanul Kabir Chowdhury, Dinh-Toi Chu, Aubrey J Cook, Natalie Maria Cormier, Richard G Cowden, Carlos Culquichicon, Baye Dagnew, Saad M A Dahlawi, Giovanni Damiani, Parnaz Daneshpajouhnejad, Farah Daoud, Ahmad Daryani, José das Neves, Nicole Davis Weaver, Meseret Derbew Molla, Kebede Deribe, Abebaw Alemayehu Desta, Keshab Deuba, Samath Dhamminda Dharmaratne, Govinda Prasad Dhungana, Daniel Diaz, Shirin Djalalinia, Paul Narh Doku, Eleonora Dubljanin, Bereket Duko, Arielle Wilder Eagan, Lucas Earl, Jeffrey W Eaton, Andem Effiong, Maysaa El Sayed Zaki, Maha El Tantawi, Rajesh Elayedath, Shaimaa I El-Jaafary, Aisha Elsharkawy, Sharareh Eskandarieh, Oghenowede Eyawo, Sayeh Ezzikouri, Abidemi Omolara Fasanmi, Alebachew Fasil, Nelsensius Klau Fauk, Valery L Feigin, Tomas Y Ferede, Eduarda Fernandes, Florian Fischer, Nataliya A Foigt, Morenike Oluwatoyin Folayan, Masoud Foroutan, Joel Msafiri Francis, Takeshi Fukumoto, Mohamed M Gad, Biniyam Sahiledengle Geberemariyam, Birhan Gebresillassie Gebregiorgis, Berhe Gebremichael, Hailay Abrha Gesesew, Lemma Getacher, Keyghobad Ghadiri, Ahmad Ghashghaee, Syed Amir Gilani, Themba G Ginindza, Mustefa Glagn, Mahaveer Golechha, Philimon N Gona, Mohammed Ibrahim Mohialdeen Gubari, Harish Chander Gugnani, Davide Guido, Rashid Abdi Guled, Brian J Hall, Samer Hamidi, Demelash Woldeyohannes Handiso, Arief Hargono, Abdiwahab Hashi, Soheil Hassanipour, Hadi Hassankhani, Khezar Hayat, Claudiu Herteliu, Hagos Degefa de Hidru, Ramesh Holla, H Dean Hosgood, Naznin Hossain, Mostafa Hosseini, Mehdi Hosseinzadeh, Mowafa Househ, Bing-Fang Hwang, Segun Emmanuel Ibitoye, Olayinka Stephen Ilesanmi, Irena M Ilic, Milena D Ilic, Seyed Sina Naghibi Irvani, Chidozie C D Iwu, Chinwe Juliana Iwu, Ihoghosa Osamuyi Iyamu, Vardhmaan Jain, Mihajlo Jakovljevic, Farzad Jalilian, Ravi Prakash Jha, Kimberly B Johnson, Vasna Joshua, Farahnaz Joukar, Jacek Jerzy Jozwiak, Ali Kabir, Leila R Kalankesh, Rohollah Kalhor, Ashwin Kamath, Naser Kamyari, Tanuj Kanchan, Behzad Karami Matin, André Karch, Salah Eddin Karimi, Ayele Semachew Kasa, Getinet Kassahun, Gbenga A Kayode, Ali Kazemi Karyani, Peter Njenga Keiyoro, Bayew Kelkay, Nauman Khalid, Gulfaraz Khan, Junaid Khan, Md Nuruzzaman Khan, Khaled Khatab, Salman Khazaei, Yun Jin Kim, Adnan Kisa, Sezer Kisa, Sonali Kochhar, Jacek A Kopec, Soewarta Kosen, Sindhura Lakshmi Koulmane Laxminarayana, Ai Koyanagi, Kewal Krishan, Barthelemy Kuate Defo, Nuworza Kugbey, Vaman Kulkarni, Manasi Kumar, Nithin Kumar, Om P Kurmi, Dian Kusuma, Desmond Kuupiel, Hmwe Hmwe Kyu, Carlo La Vecchia, Dharmesh Kumar Lal, Jennifer O Lam, Iván Landires, Savita Lasrado, Jeffrey V Lazarus, Alice Lazzar-Atwood, Paul H Lee, Cheru Tesema Leshargie, Bingyu Li, Xuefeng Liu, Platon D Lopukhov, Hawraz Ibrahim M. Amin, Deepak Madi, Phetole Walter Mahasha, Azeem Majeed, Afshin Maleki, Shokofeh Maleki, Abdullah A Mamun, Navid Manafi, Mohammad Ali Mansournia, Francisco Rogerlândio Martins-Melo, Seyedeh Zahra Masoumi, Benjamin K Mayala, Birhanu Geta Meharie, Hailemariam Abiy Alemu Meheretu, Hagazi Gebre Meles, Mulugeta Melku, Walter Mendoza, Endalkachew Worku Mengesha, Tuomo J Meretoja, Abera M Mersha, Tomislav Mestrovic, Ted R Miller, Andreea Mirica, Mehdi Mirzaei-Alavijeh, Osama Mohamad, Yousef Mohammad, Abdollah Mohammadian-Hafshejani, Jemal Abdu Mohammed, Salahuddin Mohammed, Shafiu Mohammed, Ali H Mokdad, Taklu Marama Mokonnon, Mariam Molokhia, Masoud Moradi, Yousef Moradi, Rahmatollah Moradzadeh, Paula Moraga, Jonathan F Mosser, Sandra B Munro, Ghulam Mustafa, Saravanan Muthupandian, Mehdi Naderi, Ahamarshan Jayaraman Nagarajan, Mohsen Naghavi, Muhammad Naveed, Vinod C Nayak, Javad Nazari, Rawlance Ndejjo, Samata Nepal, Henok Biresaw Netsere, Frida N Ngalesoni, Georges Nguefack-Tsague, Josephine W Ngunjiri, Yeshambel T Nigatu, Samuel Negash Nigussie, Chukwudi A Nnaji, Jean Jacques Noubiap, Virginia Nuñez-Samudio, Bogdan Oancea, Oluwakemi Ololade Odukoya, Felix Akpojene Ogbo, Olanrewaju Oladimeji, Andrew T Olagunju, Bolajoko Olubukunola Olusanya, Jacob Olusegun Olusanya, Muktar Omer Omer, Abidemi E Emmanuel Omonisi, Obinna E Onwujekwe, Orish Ebere Orisakwe, Nikita Otstavnov, Mayowa O Owolabi, Mahesh P A, Jagadish Rao Padubidri, Smita Pakhale, Adrian Pana, Seithikurippu R Pandi-Perumal, Urvish K Patel, Mona Pathak, George C Patton, Shrikant Pawar, Emmanuel K Peprah, Khem Narayan Pokhrel, Maarten J Postma, Faheem Hyder Pottoo, Hadi Pourjafar, Dimas Ria Angga Pribadi, Zahiruddin Quazi Syed, Alireza Rafiei, Fakher Rahim, Mohammad Hifz Ur Rahman, Amir Masoud Rahmani, Pradhum Ram, Juwel Rana, Chhabi Lal Ranabhat, Satish Rao, Sowmya J Rao, Priya Rathi, David Laith Rawaf, Salman Rawaf, Reza Rawassizadeh, Vishnu Renjith, Melese Abate Reta, Nima Rezaei, Aziz Rezapour, Ana Isabel Ribeiro, Jennifer M Ross, Susan Fred Rumisha, Rajesh Sagar, Maitreyi Sahu, S. Mohammad Sajadi, Marwa Rashad Salem, Abdallah M Samy, Brijesh Sathian, Aletta Elisabeth Schutte, Abdul-Aziz Seidu, Feng Sha, Omid Shafaat, Mohammad Shahbaz, Masood Ali Shaikh, Mohammed Feyisso Shaka, Aziz Sheikh, Kenji Shibuya, Jae Il Shin, K M Shivakumar, Negussie Boti Sidemo, Jasvinder A Singh, Valentin Yurievich Skryabin, Anna Aleksandrovna Skryabina, Amin Soheili, Shahin Soltani, Oluwaseyi Dolapo Somefun, Muluken Bekele Sorrie, Emma Elizabeth Spurlock, Mu'awiyyah Babale Sufiyan, Biruk Wogayehu Taddele, Eyayou Girma Tadesse, Zemenu Tamir, Animut Tagele Tamiru, Frank C Tanser, Nuno Taveira, Arash Tehrani-Banihashemi, Yohannes Tekalegn, Fisaha Haile Tesfay, Belay Tessema, Zemenu Tadesse Tessema, Bhaskar Thakur, Musliu Adetola Tolani, Roman Topor-Madry, Marco Torrado, Marcos Roberto Tovani-Palone, Eugenio Traini, Alexander C Tsai, Gebiyaw Wudie Tsegaye, Irfan Ullah, Saif Ullah, Chukwuma David Umeokonkwo, Bhaskaran Unnikrishnan, Constantine Vardavas, Francesco S Violante, Bay Vo, Yohannes Dibaba Wado, Yasir Waheed, Richard G Wamai, Yanzhong Wang, Paul Ward, Andrea Werdecker, Nuwan Darshana Wickramasinghe, Tissa Wijeratne, Charles Shey Wiysonge, Temesgen Gebeyehu Wondmeneh, Tomohide Yamada, Sanni Yaya, Yigizie Yeshaw, Yordanos Gizachew Yeshitila, Mekdes Tigistu Yilma, Paul Yip, Naohiro Yonemoto, Tewodros Yosef, Hasan Yusefzadeh, Syed Saoud Zaidi, Leila Zaki, Maryam Zamanian, Mikhail Sergeevich Zastrozhin, Anasthasia Zastrozhina, Dejene Tesfaye Zewdie, Yunquan Zhang, Zhi-Jiang Zhang, Arash Ziapour, Simon I Hay, Laura Dwyer-Lindgren

## Abstract

**Background:**

High-resolution estimates of HIV burden across space and time provide an important tool for tracking and monitoring the progress of prevention and control efforts and assist with improving the precision and efficiency of targeting efforts. We aimed to assess HIV incidence and HIV mortality for all second-level administrative units across sub-Saharan Africa.

**Methods:**

In this modelling study, we developed a framework that used the geographically specific HIV prevalence data collected in seroprevalence surveys and antenatal care clinics to train a model that estimates HIV incidence and mortality among individuals aged 15–49 years. We used a model-based geostatistical framework to estimate HIV prevalence at the second administrative level in 44 countries in sub-Saharan Africa for 2000–18 and sought data on the number of individuals on antiretroviral therapy (ART) by second-level administrative unit. We then modified the Estimation and Projection Package (EPP) to use these HIV prevalence and treatment estimates to estimate HIV incidence and mortality by second-level administrative unit.

**Findings:**

The estimates suggest substantial variation in HIV incidence and mortality rates both between and within countries in sub-Saharan Africa, with 15 countries having a ten-times or greater difference in estimated HIV incidence between the second-level administrative units with the lowest and highest estimated incidence levels. Across all 44 countries in 2018, HIV incidence ranged from 2·8 (95% uncertainty interval 2·1–3·8) in Mauritania to 1585·9 (1369·4–1824·8) cases per 100 000 people in Lesotho and HIV mortality ranged from 0·8 (0·7–0·9) in Mauritania to 676·5 (513·6–888·0) deaths per 100 000 people in Lesotho. Variation in both incidence and mortality was substantially greater at the subnational level than at the national level and the highest estimated rates were accordingly higher. Among second-level administrative units, Guijá District, Gaza Province, Mozambique, had the highest estimated HIV incidence (4661·7 [2544·8–8120·3]) cases per 100 000 people in 2018 and Inhassunge District, Zambezia Province, Mozambique, had the highest estimated HIV mortality rate (1163·0 [679·0–1866·8]) deaths per 100 000 people. Further, the rate of reduction in HIV incidence and mortality from 2000 to 2018, as well as the ratio of new infections to the number of people living with HIV was highly variable. Although most second-level administrative units had declines in the number of new cases (3316 [81·1%] of 4087 units) and number of deaths (3325 [81·4%]), nearly all appeared well short of the targeted 75% reduction in new cases and deaths between 2010 and 2020.

**Interpretation:**

Our estimates suggest that most second-level administrative units in sub-Saharan Africa are falling short of the targeted 75% reduction in new cases and deaths by 2020, which is further compounded by substantial within-country variability. These estimates will help decision makers and programme implementers expand access to ART and better target health resources to higher burden subnational areas.

**Funding:**

Bill & Melinda Gates Foundation.

## Introduction

As the HIV pandemic enters its fifth decade, several indicators have been proposed to help describe the burden of HIV, measure the effectiveness of public health efforts, and guide decision making. Among the most useful and commonly cited indicators are the HIV incidence rate, the HIV mortality rate, the percentage reduction in the number of incident HIV cases and HIV deaths, and the ratio of incident HIV cases to people living with HIV.^1^ The UN Political Declaration on HIV and AIDS calls for a 75% reduction in new HIV infections and HIV deaths from 2010 to 2020.^2^ Studies have shown that geographical targeting of resources can improve the efficiency and effectiveness of interventions and strategies intended to address HIV.^3^, ^4^ To best aid resource targeting, HIV indicators need to be produced at a refined spatial scale. Yet, for countries in sub-Saharan Africa—those hardest hit by the HIV pandemic—collecting data on indicators of HIV incidence and mortality remains a challenge, particularly at spatially granular levels, where weaknesses in civil registration and vital statistics systems are typically beyond the capacity or funding for HIV programmes alone to address. Historically, most data collection systems for tracking HIV have focused on measuring HIV prevalence, partly because HIV prevalence is inherently more straightforward to measure than HIV incidence or mortality, and partly driven by programmatic needs including daily patient management, ensuring sufficient drug supply, and managing loss to follow-up. The Population-based HIV Impact Assessment survey series and other household surveys have attempted to include direct measures of HIV incidence via recency assays^5^ and many countries are introducing routine recency testing for newly diagnosed individuals;^6^ however, these data are not yet as widespread as data related to HIV prevalence and concerns remain regarding the validity and reliability of recency assays for accurately estimating HIV incidence.^7^

**Research in context**

**Evidence before this study**

We searched PubMed with no language restrictions for articles published since database inception until Dec 31, 2020, using the following search terms: “hiv[MeSH] AND (“mortality” OR “incidence” OR “prevalence”) AND “subnational” AND (trend*)”. Previous research has shown that substantial local (spatial) variation exists in HIV incidence, and modelling studies comparing geographically targeted with non-geographically targeted prevention strategies have suggested that geographically targeted strategies are more efficient in preventing new HIV infections under the same budgetary constraints. Trends in HIV mortality and incidence have varied at both regional and country levels, resulting in differing trends in HIV prevalence, and this dynamic is further complicated by the paucity of directly observed empirical data on HIV incidence and mortality in sub-Saharan Africa and other high-burden low-income and middle-income countries. Renewed commitment is required to assess progress towards global targets at a subnational scale, to ensure no sub-populations are left behind, and to support sub-Saharan Africa in getting on track to bring HIV infection under control by 2030.

**Added value of this study**

Although many initiatives provide national estimates for HIV metrics (and at the administrative level in some countries), there are few HIV incidence and mortality estimates and necessary methodological innovation at more detailed subnational scales. This study suggests substantial variation exists in HIV incidence and mortality rates both between and within countries in sub-Saharan Africa, with highly variable rates of reduction in HIV incidence and mortality and the ratio of new infections to the number of people living with HIV from 2000 to 2018. Although most second-level administrative units had declines in the number of new cases and attributable deaths, nearly all appeared well short of the targeted 75% reduction in new cases and deaths between 2010 and 2020.

**Implications of all the available evidence**

By improving and extending existing HIV incidence and mortality estimates in sub-Saharan Africa at a subnational scale, this study provides valuable estimates to help gauge progress towards ending the HIV epidemic by 2030 (Sustainable Development Goal 3) and provides an important tool to improve the precision and efficiency of targeting interventions within countries.

Because trends in HIV incidence and mortality are largely not directly observed at the national level in sub-Saharan Africa, estimates are developed by fitting mathematical models to data on trends in HIV prevalence. The Estimation and Projection Package (EPP),^8^ developed by UNAIDS and also used by the Global Burden of Disease (GBD) study,^9^, ^10^ provides a well-tested structure for leveraging the HIV prevalence data available from population surveys and antenatal care sentinel surveillance sites to estimate HIV incidence and mortality. UNAIDS, the President's Emergency Plan for AIDS Relief, and others have called for incorporating local data and estimates into country HIV response strategies, given subnational heterogeneity in the HIV epidemic. Although subnational estimates of HIV prevalence and antiretroviral therapy (ART) coverage are increasingly common, to our knowledge, estimates of HIV incidence and mortality are not yet routinely available below the first administrative level.

Here, we present a modified version of the EPP model, which combines developments in spatial demography,^11^ fine-scale HIV prevalence mapping,^12^ and HIV pandemic modelling^9^, ^10^, ^13^ to produce estimates of HIV incidence and mortality for first-level (eg, provinces) and second-level administrative units (eg, districts) across 44 sub-Saharan African countries. Estimates of these indicators at this fine spatial scale can assist in tracking and accelerating progress towards meeting the Sustainable Development Goal of “ending the AIDS pandemic as a public health threat by 2030”.^14^

## Methods

### Study design

Our study follows the Guidelines for Accurate and Transparent Health Estimates Reporting (appendix pp 18, 19).^15^ We used a modified version of the EPP mathematical compartmental model, tailored specifically to estimate HIV incidence and mortality, among individuals aged 15–49 years for first-level and second-level administrative units in 44 countries in sub-Saharan Africa. EPP fits the transmission rate to the prevalence using a series of assumptions about how the different epidemic indicators relate to each other within a given population. This fitting is achieved using the Bayesian incremental mixture importance sampling solver, which aligns the HIV prevalence output of each EPP simulation to the HIV prevalence from our model-based geostatistical prevalence model. Analyses were done using the R (version 3.6.1) and C (version GNU99) programming languages. Further details of the methods, input data types and sources, and assumptions are provided in the appendix (pp 20–30).

### Modelling strategy

Rather than develop a methodology de novo, we sought a tested modelling framework that (1) could leverage HIV prevalence data to estimate HIV incidence and mortality; (2) could use available demographic data without requiring calibration of many behavioural parameters that are rarely available at subnational resolutions; and (3) had a history of producing reliable and widely used results. EPP^8^ meets these needs and this system has been used by both UNAIDS^13^, ^16^ and GBD^9^, ^10^ to produce national and first-administrative level estimates of HIV incidence and mortality across sub-Saharan Africa.

EPP is a compartmental epidemiological model, which is designed to estimate the HIV incidence and mortality rates needed to produce a time trend specific to HIV prevalence**.** The model functions by varying model parameters to identify HIV incidence trends that are most consistent with observed HIV prevalence, given the number of patients on ART (appendix pp 20–30). The version of EPP that we present here is a modified version of that used to create the GBD 2017 HIV estimates^9^ and operates on individuals aged 15–49 years of both sexes as one intermixing population. Developments in EPP^13^ have allowed UNAIDS and GBD to use an age-specific and sex-specific version of EPP (EPP-ASM) for national models and, in some cases, at the first administrative level. However, use of that model was not feasible for our spatially granular implementation because of the additional computational burden of fitting this more complex model and because EPP-ASM benefits from age-specific and sex-specific HIV prevalence estimates, which are not yet widely available for second-level administrative units. We modified the GBD 2017 version of EPP to use the HIV prevalence time series produced using a model-based geostatistical framework,^12^ as opposed to direct survey and antenatal care estimates of HIV prevalence, which are typically used in EPP. Because internal migration between second-level administrative units is potentially important for our model but difficult to measure, we adopted the approach now being used by UNAIDS and the GBD in the EPP-ASM model to adjust the population at the end of each timepoint to the expected population in the next timepoint using a simple scalar. This method removes the need to explicitly model migration and instead relies on the population count estimates in each administrative unit. Our last major modification to EPP was to implement the r-hybrid model employed by UNAIDS in 2018 and GBD in 2019, which has been shown to work best for most geographical areas.^13^ In brief, r-hybrid estimates the HIV transmission rate differently in the early versus later phases of the HIV epidemic to better match observed data.

### Model inputs

The model has five key inputs: (1) the boundaries (or shapes) used to define the second-level administrative units we are modelling; (2) the size of the population aged 15–49 years over time that we used as the demographic bases for the hypothetical epidemics; (3) the modelled HIV prevalence in each of the second-level administrative units; (4) the number of people on ART in each second-level administrative unit; and (5) the assumptions used about how likely a person living with HIV is to die from their infection.

To delineate the boundaries of the second-level administrative units we began with the second-level administrative shapefiles that are publicly available from the Database of Global Administrative Areas. These boundaries were modified to correct for known errors and to accommodate recent boundary changes. A full list of changes and the naming convention for first-level and second-level administrative units across the 44 countries in sub-Saharan Africa can be found in the appendix (pp 49–51).

To estimate populations in second-level administrative units, we used high-resolution gridded population estimates that were age specific and sex specific from WorldPop.^11^ To create a full time series from 1970 to 2020, we interpolated additional years of data using the population growth rate at the pixel level observed in the WorldPop dataset, assuming exponential growth, and scaled the total population in each country to match GBD national population estimates. Finally, these gridded population estimates were aggregated fractionally to the shapefiles as described, to create second-level administrative unit population estimates.

We used an updated version of the model-based geostatistical methodology from our previous work^12^ to produce HIV prevalence estimates for all second-level administrative units across sub-Saharan Africa from 1995 to 2018. This year range was chosen because we were able to extract geolocated sentinel surveillance data for antenatal care and household survey estimates of HIV prevalence over this period. A full list of HIV prevalence data incorporated into this analysis can be found in the appendix (pp 52–101). In summary, 145 surveys (80 surveys with microdata, 28 survey reports, and 37 surveys extracted from published literature) and 134 sources of antenatal care sentinel surveillance data, which in combination resulted in a geopositioned data set of 29 072 survey observations and 11 710 site-years of antenatal care sentinel surveillance, formed the input for the HIV prevalence component.

We used several data sources to estimate the number of individuals on ART in each second-level administrative unit. The UNAIDS annual estimate files^17^ provide the number of adults receiving ART in each country but this information is not sufficiently granular to use at the second administrative unit level. Therefore, we did a systematic data-seeking exercise to extract all available subnational ART data in the 44 countries included in this analysis and successfully identified subnational-level ART information in 29 countries (appendix pp 201–03). In countries where subnational ART information was available, we modelled these data to create a full time series (appendix pp 27–29). In the 15 countries where we were unable to locate subnational ART information, we assumed that the national ART coverage rate was consistent in all second-level administrative units and redistributed ART patients accordingly.

HIV mortality rates were calculated for individuals with HIV of varying disease severity separately for those who were and were not receiving ART.^10^ EPP divides the population with HIV into seven CD4 cell count categories as a proxy for disease severity and into two treatment categories (on or off ART). We tracked progression through the CD4 categories and onto treatment so that at every time step of the EPP model, we had an estimate of the size of the population for each disease severity and treatment category (appendix p 30). We then applied the estimated HIV mortality rate from GBD that is specific to each CD4 and treatment category to the population in these groups to estimate HIV mortality.

### Effect of ART

ART is a key input to this model because the treatment substantially decreases viraemia and thus the probability that an individual with HIV will die from or pass on their infection;^18^ thus, ART fundamentally changes the relationship between HIV incidence, prevalence, and mortality. We were not able to identify and extract subnational ART information in all countries, and so, to assess the effect of using these data, in the 29 countries where we were able to extract subnational ART data we ran EPP using both the extracted subnational ART data and assuming the national ART coverage rate. We then compared the two scenarios to ascertain the effect of ART on our models. As expected, ART coverage in each second-level administrative unit had a substantial effect on the HIV incidence and mortality estimate in that location (appendix pp 31, 32). Subnational variation in HIV incidence and mortality, however, was still present in countries where we assumed the national ART coverage for all second-level administrative units, driven by variation in the level and trend of HIV prevalence. Figures in the appendix (pp 33–36) show the relationship between estimated HIV incidence, mortality, prevalence, and ART coverage.

### Uncertainty interval estimation

To account for uncertainty in our model inputs, including the disease progression and mortality parameters, we ran 100 simulations of EPP varying these parameters. Within each simulation, we generated 1000 draws from the approximated posterior distribution of HIV prevalence, HIV incidence, and HIV mortality. To create a single, combined posterior distribution we sampled ten draws from each of the 100 simulations and then combined these draws. For consistency with national-level estimates using much of the same underlying data, we calibrated our results to match the estimates from the GBD 2019.^10^ Further details are provided in the appendix (pp 24, 25).

To account for uncertainty in our estimates of HIV incidence and mortality when assessing progress towards achievement of the UNAIDS target of a 75% reduction in new HIV infections and HIV deaths, we calculated the posterior probability of achieving these targets as the percentage of draws from the estimated posterior distribution where these targets were achieved.

### Role of the funding source

The funders of this study had no role in the study design, data collection, data analysis, data interpretation, or writing of the report.

## Results

**Figure 1:**
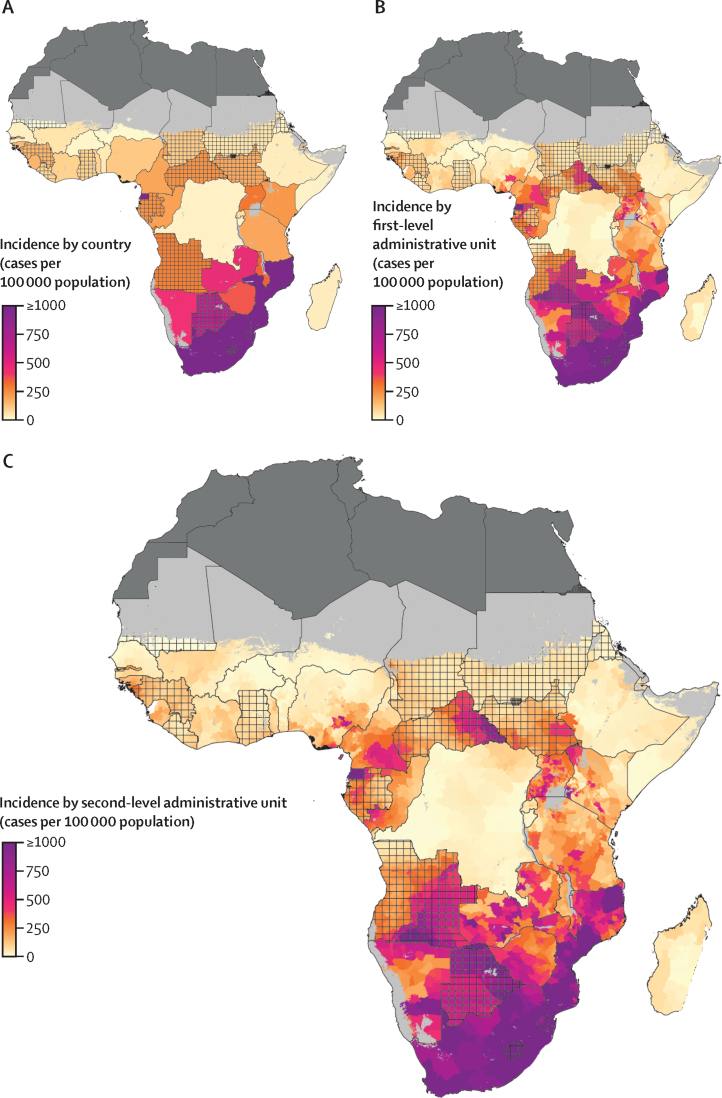
HIV incidence among individuals aged 15–49 years in sub-Saharan Africa in 2018 Incidence among individuals aged 15–49 years by (A) country, (B) first-level administrative unit, and (C) second-level administrative unit. Lakes and areas with fewer than ten people per 1 × 1 km and classified as barren or sparsely vegetated are coloured light grey. Areas in dark grey were not included in the analysis. Estimates in areas that are crossed are based on national, rather than subnational, estimates of antiretroviral therapy coverage only.

**Figure 2:**
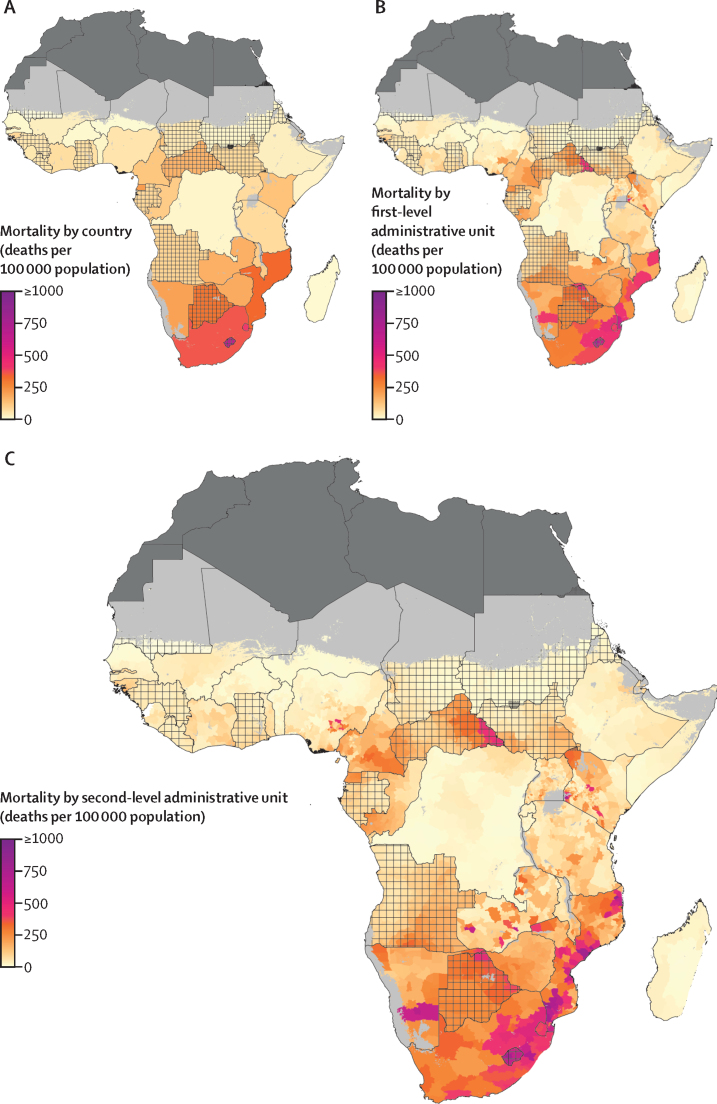
HIV mortality among individuals aged 15–49 years in sub-Saharan Africa in 2018 (A) HIV mortality among individuals aged 15–49 years by country, (B) first-level administrative unit, and (C) second-level administrative unit. Lakes and areas with fewer than ten people per 1 × 1 km and classified as barren or sparsely vegetated are coloured light grey. Areas in dark grey were not included in the analysis. Estimates in areas that are crossed are based on national, rather than subnational, estimates of antiretroviral therapy coverage only.

We found marked regional differences in HIV incidence and mortality among individuals aged 15–49 years from Jan 1, 2000, to Dec 31, 2018. Across the entire modelled region in 2018, the HIV incidence rate was 218·1 (95% uncertainty interval [UI] 196·4–239·1) cases per 100 000 people and the HIV mortality rate was 87·2 (76·6–101·1) deaths per 100 000 people. At the national level in 2018, HIV incidence ranged from 2·8 (2·1–3·8) cases per 100 000 people in Mauritania to 1585·9 (1369·4–1824·8) cases per 100 000 people in Lesotho (figure 1A; appendix p 37), and HIV mortality ranged from 0·8 (0·7–0·9) deaths per 100 000 people in Mauritania and 676·5 (513·6–888·0) deaths per 100 000 people in Lesotho (figure 2A; appendix p 39). The variation in both incidence and mortality was substantially greater at the subnational compared with the national level and the highest estimated rates were accordingly higher. The first-level administrative unit with the highest estimated HIV incidence rate in 2018 was Gaza Province in Mozambique, with an incidence rate of 2805·9 (2118·0–3611·2) cases per 100 000 people (figure 1B). Among second-level administrative units, Guijá District in Gaza Province, Mozambique, had the highest estimated HIV incidence, with 4661·7 (2544·8–8120·3) cases per 100 000 people in 2018 (figure 1C). Among second-level administrative units, Inhassunge District in Zambezia Province, Mozambique, had the highest HIV mortality rate estimate at 1163·0 (679·0–1866·8) deaths per 100 000 people (figure 2C).

In addition to large-scale variation across the region, we also found substantial within-country variation in HIV incidence and mortality. In 2018, 15 countries (Angola, Benin, Burkina Faso, Burundi, Cameroon, Democratic Republic of the Congo, Ethiopia, Kenya, Mozambique, Nigeria, Senegal, Somalia, Tanzania, Uganda, and Zambia) had a greater than ten-times difference in HIV incidence between the second-level administrative units with the lowest and highest estimated incidence levels. Of those 15 countries, 11 also had a greater than ten-times difference in HIV mortality rates between their lowest and highest second-level administrative units. Kenya was a particularly extreme example of this variability, with incidence rate estimates ranging from 14·2 (95% UI 4·1–41·3) cases per 100 000 people in Eldas Constituency, Wajir County, to 1767·0 (939·7–2957·9) cases per 100 000 people in Rarieda Constituency, Siaya County, and HIV mortality rate estimates ranging from 5·7 (2·4–15·2) deaths per 100 000 people in Eldas Constituency, Wajir County, to 789·5 (524·9–1165·1) in Suba Constituency, Homa Bay County, in 2018.

**Figure 3:**
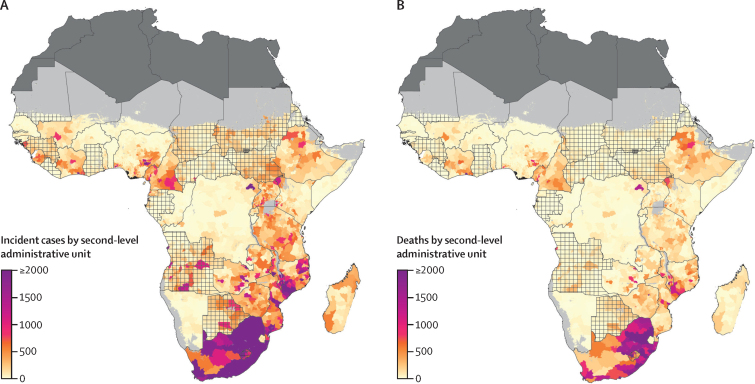
Incident HIV cases and deaths among individuals aged 15–49 years in sub-Saharan Africa in 2018 (A) Number of incident HIV cases and (B) HIV deaths among individuals aged 15–49 years in 2018 by second-level administrative unit. Lakes and areas with fewer than ten people per 1 × 1 km and classified as barren or sparsely vegetated are coloured light grey. Areas in dark grey were not included in the analysis. Estimates in areas that are crossed are based on national, rather than subnational, estimates of antiretroviral therapy coverage only.

In absolute terms, incident HIV cases and HIV deaths were highly concentrated in high-population locations. In 2018, we estimated 1 138 827 (95% UI 1 025 447–1 248 270) incident HIV cases across the 44 modelled countries. 50% of these incident HIV cases in 2018 were located in just 148 (3·6%) of 4087 second-level administrative units that collectively represented 13·7% of the total population in this region (figure 3A). Most of these high-burden administrative units were located in southern sub-Saharan Africa; in particular, both Lesotho and South Africa had more than 50% of their second-level administrative units in this category. Conversely, 2630 (64·4%) of 4087 second-level administrative units, representing 38·2% of the total population, accounted for less than 10% of the total estimated incident HIV cases.

In 2018, we estimated that 455 244 (95% UI 399 851–527 712) HIV deaths took place in the 44 modelled countries. Only 224 (5·5%) of 4087 second-level administrative units, representing 22·3% of the total population, accounted for 50% of the estimated deaths (figure 3B). 2364 (57·8%) of 4087 second-level administrative units, representing 30·0% of the total population, contributed less than 10% of the total estimated HIV deaths in 2018.

**Figure 4:**
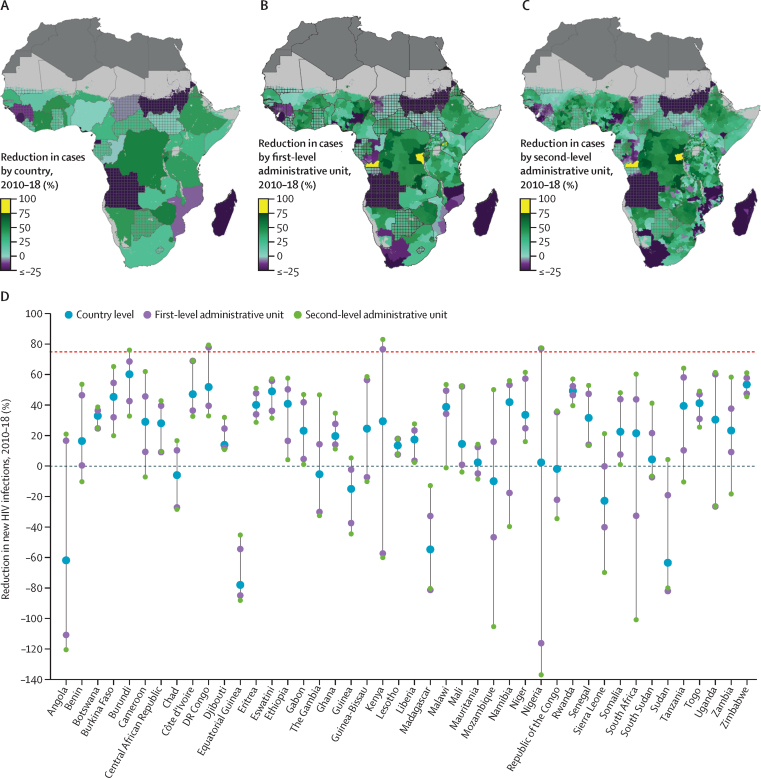
Percentage reduction in incident HIV cases in sub-Saharan Africa from 2010 to 2018 (A) Reduction in the number of incident HIV cases (%) between 2010 and 2018 among individuals aged 15–49 years by country, (B) first-level administrative unit, and (C) second-level administrative unit. Lakes and areas with fewer than ten people per 1 × 1 km and classified as barren or sparsely vegetated are coloured light grey. Areas in dark grey were not included in the analysis. Estimates in areas that are crossed are based on national, rather than subnational, estimates of antiretroviral therapy coverage only. A 75% reduction in HIV incidence by 2020 is a UNAIDS fast-track goal. Progress towards this target by country highlighting the best and worst performing subnational units is shown in panel D.

The UNAIDS fast-track goals,^2^ which are designed to set measurable targets for public health action, call for a 90% reduction in both incident HIV cases and HIV deaths by 2030, as compared with 2010 levels. An additional intermediate target was set to a 75% reduction in both indicators by 2020. Across our modelled region, incident HIV cases reduced by 16·9% (95% UI 6·8–25·1) and the HIV incidence rate reduced by 33·8% (25·8–40·3) between 2010 and 2018, both falling well short of the UNAIDS intermediate goal to reduce HIV incidence by 75% by 2020. We estimated that no country has yet achieved the goal of a 75% reduction in new infections on a national scale (figure 4D). Our estimates show wide variability among subnational areas in progress towards achieving this goal. We estimated that only six (0·9%) of 686 first-level administrative units (figure 4B) and 64 (1·6%) of 4087 second-level administrative units (figure 4C) had already achieved a 75% reduction in incident HIV cases by 2018.

Our estimates suggest that increases in the number of incident HIV cases are far too common. At the national level, Angola (61·2% [95% UI 49·2–73·9] increase), Equatorial Guinea (77·3% [52·1–103·8]), Guinea (14·3% [0·72–32·2]), Madagascar (54·0% [32·7–80·3]), Mozambique (9·3% [0·7–19·5]), Sierra Leone (22·1% [6·4–40·2]), and Sudan (62·7% [48·8–79·6]) all had an increase in incident HIV cases with greater than 95% posterior probability (figure 4A). Increases in estimated incident HIV cases occurred in 130 (19·0%) of 686 first-level administrative units that were distributed across 18 countries (figure 4B). 771 (18·9%) of 4087 second-level administrative units across 24 countries had an increase in the estimated number of new infections between 2010 and 2018 (figure 4C). At the second-level administrative unit, the two districts with the largest estimated increases in HIV incidence from 2010 to 2018 were located in Mozambique and South Africa (figure 4D), with estimated increases in HIV incidence in excess of 50% over this period. Fewer locations saw an increase in the estimated HIV incidence rate and those increases were generally smaller in scale than corresponding changes in the number of new infections (appendix p 41), underscoring the effect of population growth on the pandemic.

**Figure 5:**
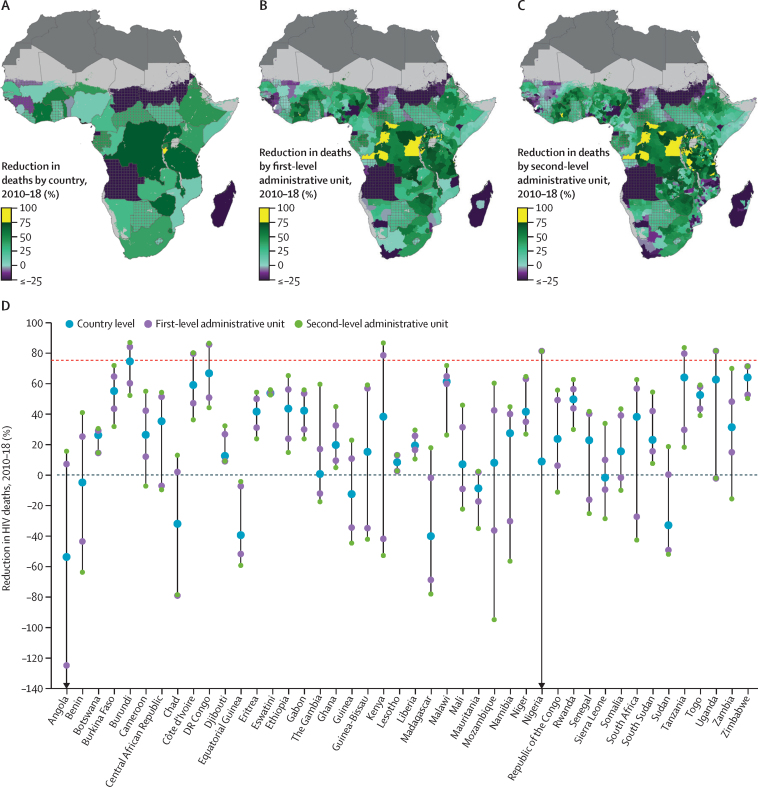
Percentage reduction in HIV deaths in sub-Saharan Africa from 2010 to 2018 (A) Reduction in the number of HIV deaths (%) between 2010 and 2018 among individuals aged 15–49 years by country, (B) first-level administrative unit, and (C)second-level administrative unit. Lakes and areas with fewer than ten people per 1 × 1 km and classified as barren or sparsely vegetated are coloured light grey. Areas in dark grey were not included in the analysis. Estimates in areas that are crossed are based on national, rather than subnational, estimates of antiretroviral therapy coverage only. A 75% reduction in HIV deaths by 2020 is a UNAIDS fast-track goal. Progress towards this target by country highlighting the best and worst performing subnational units is shown in panel D.

We estimated reductions in HIV deaths that were more widespread. Across the modelled countries, HIV deaths fell by 38·3% (95% UI 34·6–41·5) from 2010 to 2018. Although a substantial improvement, this reduction was still well short of the 75% reduction hoped to be achieved by 2020. Only one country, Burundi, achieved this benchmark at a national scale (figure 5A, D). Conversely, Angola, Equatorial Guinea, Madagascar, and Sudan had the largest increases in estimated HIV deaths at the national scale from 2010 to 2018 (figure 5D). 44 (6·4%) of 686 first-level administrative units had achieved the 75% reduction in HIV deaths by 2018 (figure 5B) and 263 (6·4%) of 4087 second-level administrative units showed a 75% or greater reduction in HIV deaths since 2010. These second-level administrative units were spread across seven countries: Burundi, Côte d'Ivoire, Democratic Republic of the Congo, Kenya, Nigeria, Tanzania, and Uganda (figure 5C).

Angola (53·0% increase [95% UI 45·1–61·8]), Chad (31·3% [12·6–51·4]), Equatorial Guinea (38·7% [19·1–59·3]), Guinea (11·9% [0·1–24·9]), Madagascar (39·4% [26·0–55·8]), and Sudan (32·3% [24·1–42·4]) all had increases in the number of estimated HIV deaths between 2010 and 2018 (figure 5A, D). At the first-level administrative unit scale, 129 (18·8%) of 686 units showed an increase in HIV deaths (figure 5B). Almost a fifth (18.6%; 762 of 4087) of second-level administrative units had an estimated increase in HIV deaths over the 2010–18 period. These units were spread across 24 countries but Angola, Chad, Equatorial Guinea, Madagascar, Mauritania, and Sudan had more than 75% of their second-level administrative units show increases in HIV deaths over the period (figure 5C). Population growth over the period 2010–18 meant that reductions in the HIV mortality rate were more common and generally on a larger scale than reductions in the number of HIV deaths (appendix p 42).

We found that very few locations had even a moderate posterior probability of having had achieved the UNAIDS targeted reduction in HIV incidence (appendix pp 43, 44). More locations, in Burundi, Democratic Republic of Congo, Nigeria, Tanzania, and Uganda, had at least a 50% posterior probability of having achieved the UNAIDS targeted reduction in HIV deaths (appendix pp 45, 46).

**Figure 6:**
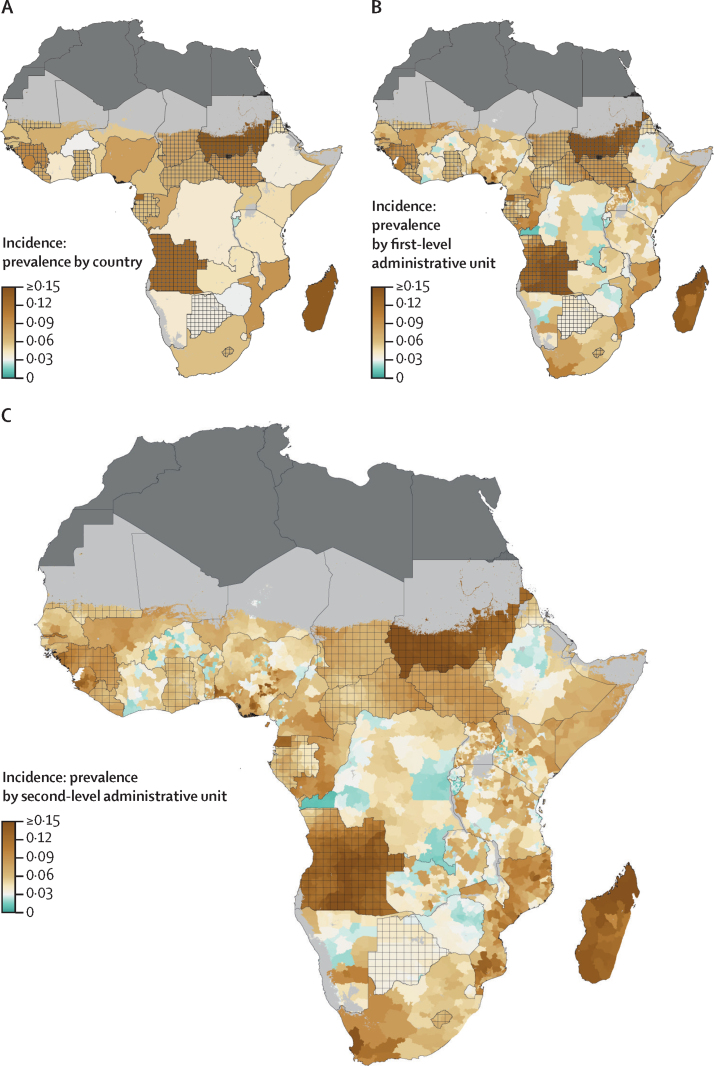
HIV incidence to prevalence ratio in sub-Saharan Africa in 2018 Number of new infections among individuals aged 15–49 years divided by the number of individuals living with HIV aged 15–49 years by (A) country, (B) first-level administrative unit, and (C) second-level administrative unit. Lakes and areas with fewer than ten people per 1 × 1 km and classified as barren or sparsely vegetated are coloured light grey. Areas in dark grey were not included in the analysis. Estimates in areas that are crossed are based on national, rather than subnational, estimates of antiretroviral therapy coverage only. Achieving a sustained incidence to prevalence ratio of less than 0·03 by 2020 is a UNAIDS fast-track goal.

Understanding the ratio of the number of new infections to the number of people living with HIV (IPR) provides critical information regarding how well the HIV pandemic is being brought under control. An IPR below 0·03 has been suggested as a benchmark value because the number of people living with HIV is expected to shrink over time below this threshold.^1^ At the national level, two countries (Burundi and Zimbabwe) had achieved this goal in 2018 (figure 6A). 66 first-level administrative units in 19 countries had estimated IPR values below 0·03 in 2018 (figure 6B); 583 second-level administrative units in 22 countries also had estimated IPR values below 0·03 in 2018 (figure 6C). To account for uncertainty in whether or not an administrative unit had achieved this goal as a result of uncertainty in our incidence and prevalence estimates, we calculated the posterior probability that the IPR in each administrative unit was below 0·03 (appendix p 47).

## Discussion

Our estimates, the first set of HIV incidence and mortality estimates available for all second-level administrative units across sub-Saharan Africa, suggest highly variable levels of HIV incidence and mortality as well as variable rates of reduction. Nonetheless, most second-level administrative units in the region appear to be falling well short of the targeted 75% reduction in new cases and deaths by 2020. Previous spatial analyses of the HIV pandemic across sub-Saharan Africa have mainly focused on prevalence because of the lack of spatially explicit HIV incidence and mortality data. HIV prevalence estimates and the corresponding estimates of the number of people living with HIV are useful for measuring the need for treatment and related services; nonetheless, most benchmarks and targets are related to HIV incidence and mortality because these metrics are more sensitive, timely indicators of the progression of the HIV epidemic, compared with prevalence estimates. The estimates we present here, in conjunction with the HIV prevalence estimates^12^ on which they are based, provide a granular picture of how the HIV pandemic is progressing in communities across sub-Saharan Africa. These estimates can be used for programmatic targeting and subnational goal setting in the fight against HIV. Modelling studies have shown improved programme efficiency when prevention services are geographically targeted using an understanding of local epidemiology.^3^, ^4^ Moreover, studies in South Africa have identified geographical hotspots as crucial to the spread of HIV more broadly.^19^ Our estimates provide a mechanism to identify second-level administrative units where HIV incidence rates are predicted to be particularly high and might warrant further investigation as potential hotspots of HIV infection as well as areas requiring additional investment to improve both coverage and quality of services. Although the rapid scale-up of ART towards the UNAIDS 90-90-90 goals has already contributed to substantial reductions in HIV incidence and mortality in sub-Saharan Africa, there has been considerable debate as to whether universal test and treat can end the epidemic in the region. A modelling study using Eswatini as a case study suggests that even when assuming the most ideal scenarios, meeting ART coverage targets alone will be insufficient to bring infections below epidemic control levels in the most intense HIV epidemic settings.^20^ This finding suggests that highly endemic countries could achieve the 90-90-90 target thresholds but still have incidence rates above the epidemic control threshold (incidence <1 infection per 1000 person-years) and further highlights the need for incidence and mortality estimates at subnational scales to assess progress in addition to the universal test and treat targets. Lastly, these estimates make it possible to identify areas where HIV mortality rates are still predicted to be high, which similarly warrant further investigation to identify and rectify the root causes of poor outcomes among people living with HIV.

Despite substantial progress in reducing both HIV incidence and mortality over the past decade—faster in sub-Saharan Africa than in many other regions^21^—neither the number of incident HIV cases nor the number of HIV deaths has declined sufficiently to achieve the UNAIDS fast-track goals for 2020,^2^ and the world is not on track to achieve the Sustainable Development Goal of ending the HIV and AIDS epidemic by 2030.^14^ Further progress might even be more challenging, given the stagnation of development assistance for health focused on HIV^22^ and the widespread disruptions of intervention efforts due to the myriad detrimental influences of COVID-19 on health systems.^23^ Renewed efforts and new tools are needed to ultimately bring HIV infection under control in sub-Saharan Africa and globally. The methods and resulting estimates described here provide one such tool to contribute to the monitoring and assessment of these efforts.

A comparison of our country-level estimates with those from UNAIDS, although largely similar, suggests a few notable differences, for example in Lesotho and Sierra Leone. Although the methodological approach and data inputs used by UNAIDS, the present study, and GBD (to which our estimates are calibrated) are broadly similar, several differences in the approach exist nonetheless. Differences include, for example, differences in the disease progression and mortality parameters used in EPP and in the data inputs used, which can lead to meaningful differences in the resulting estimates of HIV burden. More generally, in some cases the underlying data are ambiguous and different analyses of these data might reasonably reach different conclusions, although generally with substantial uncertainty. In these cases, the differences between estimates generated using different approaches and by different research groups present an opportunity to compare estimates and to further interrogate the underlying data. Despite these differences, comparison with available and recent estimates for Lesotho^24^ and Sierra Leone,^25^ for example, suggest that our estimates have face validity.

Our methods for estimating subnational HIV incidence and mortality are subject to several limitations, as are most studies of this type. Estimates derived from EPP are only as reliable as the inputs provided. We included no direct measures of HIV incidence or mortality in our modelling process, primarily because of the rarity of such data at local levels. This paucity of directly observed, gold-standard data makes validating our estimates difficult and, as such, these estimates should be used with caution and in conjunction with local HIV programme information. Furthermore, large-scale household surveys are generally powered to produce design-based estimates at the scale of the national or first-administrative level only.

A second limitation relates to migration. Human movement was an important driver of the early HIV pandemic^26^ and still remains one of the key features driving HIV incidence.^19^, ^27^ Subnational migration data are scarce and limited in terms of geographical representativeness and generally do not capture differential rates of migration among HIV-positive and HIV-negative individuals, and so our model assumes that any in-migrant population has the same HIV prevalence as that of the population already residing in that area. Furthermore, data on circular migration for labour are also scarce and limited in terms of geographical coverage and difficult to model dynamically in space-time. Our modelled HIV incidence should be interpreted as the number of new infections in the resident population of a second-level administrative unit needed to maintain the measured HIV prevalence in that population, thus, we make no claims about where HIV transmission took place among mobile populations. Future modelling efforts that can explicitly model long-term and circular human movement will be a substantial improvement in this regard.

A third limitation of our model is ART data with poor spatial granularity. In 15 countries, we were only able to identify ART data at the national level and this lack of subnational ART information has meaningful effects on our modelled estimates. Furthermore, even in cases where ART data were available for each second-level administrative unit, these data were most often tabulated by where ART was received, rather than by the patient's residence. Although evidence exists that people living with HIV in some cases cross internal borders when seeking treatment,^28^ we were not able to account for this in our analysis. ART coverage also has limitations as a proxy effect indicator because variations in adherence and retention imply ART coverage might only be a loose measure of actual clinical efficacy.^29^ Further, ART is not the only intervention expected to affect HIV incidence and mortality and future work should consider incorporating other important interventions (eg, pre-exposure prophylaxis^30^) as data allow. Moreover, incorporation of additional steps in the treatment cascade, particularly diagnosis,^31^ into the modelling framework could strengthen the estimates and provide additional useful indicators.

A fourth limitation relates to the age groups modelled. Specifically, EPP treats the entire population aged 15–49 years as a single group, whereas EPP-ASM stratifies the underlying population by age and sex. We chose to use EPP for this analysis to limit computational burden and because we expected the benefits of EPP-ASM to be most apparent when fit to prevalence data that are age specific and sex specific, which are not yet widely available at a fine subnational scale. However, we have ongoing work aimed at estimating age-specific and sex-specific prevalence on a local scale, which will help to fill this gap, and using these estimates and EPP-ASM to produce age-stratified and sex-stratified estimates of HIV incidence and mortality on a subnational scale is an important area for future research. Additionally, our study was limited to individuals aged 15–49 years and did not consider trends in paediatric HIV, although local variation in paediatric HIV incidence and mortality is likely to exist. Future work should aim to include relevant paediatric data sources to account for this potential heterogeneity.

Finally, many of the inputs used in this analysis—including HIV prevalence, ART coverage, population size and age and sex structure, and disease and mortality progression parameters—are themselves estimates and are subject to error, which will likely be propagated into our estimates of HIV incidence and mortality. Where possible (ie, for HIV prevalence and the disease progression and mortality parameters) we have propagated the uncertainty inherent in these underlying inputs into the reported UIs for HIV incidence and mortality. However, we could not quantify the uncertainty in ART coverage or population size or structure and could not reflect this uncertainty in our reported UIs. Consequently, the UIs are likely to underestimate true uncertainty to some degree, although the extent of this underestimation is difficult to assess.

Correspondence to: Dr Laura Dwyer-Lindgren, Institute for Health Metrics and Evaluation, Seattle, WA 98195, USA ladwyer@uw.edu

## Data sharing

All national and subnational estimates can be explored via a customised visualisation tool (https://vizhub.healthdata.org/lbd/hiv-inc-mort) and are available to download from the Global Health Data Exchange (http://ghdx.healthdata.org/record/ihme-data/sub-saharan-africa-hiv-incidence-mortality-geospatial-estimates-2000-2018). The source code and study data, including full sets of estimates at the first and second administrative levels, are available via the Global Health Data Exchange (http://ghdx.healthdata.org/record/ihme-data/sub-saharan-africa-hiv-incidence-mortality-geospatial-estimates-2000-2018).

## Declaration of interests

R Ancuceanu reports consultancy or speakers' fees from UCB, Sandoz, Abbvie, Zentiva, Teva, Larophram, Cegedim, Angelini, Biessen Pharma, Hofigal, AstraZeneca, and Stada. J W Eaton reports grants from Bill & Melinda Gates Foundation, the US National Institutes of Health, and UNAIDS, during the conduct of the study. J J Jozwiak reports personal fees from Boehringer Ingelheim, Teva, Zentiva, and Amgen, outside the submitted work. K Krishan reports non-financial support from University Grants Commission Centre of Advanced Study, (CAS II), awarded to the Department of Anthropology, Panjab University, Chandigarh, India, outside the submitted work. J F Mosser reports grants from Bill & Melinda Gates Foundation, during the conduct of the study. S R Pandi-Perumal reports non-financial support from Somnogen Canada; and personal feesf rom royalties associated with editing volumes, during the conduct of the study. M J Postma reports grants and personal fees from MSD, GlaxoSmithKline, Pfizer, Boehringer Ingelheim, Novavax, Bristol Myers Squibb, Astra Zeneca, Sanofi, IQVIA, and Seqirus; personal fees from Quintiles, Novartis, and Pharmerit; grants from Bayer, BioMerieux, WHO, EU, Foundation for Innovative New Diagnostics, Antilope, Ministry of Research, Technology and Higher Education of the Republic of Indonesia, Indonesia Endowment Fund for Education, and Budi; stock options in Health-Ecore and PAG; and acting as advisor to Asc Academics, all outside the submitted work. A E Schutte reports personal fees from Servier, Takeda, Abbott, and Novartis, all outside the submitted work. J A Singh reports personal fees from Crealta/Horizon, Medisys, Fidia, Two labs, Adept Field Solutions, Clinical Care Options, Clearview Healthcare Partners, Putnam Associates, Focus forward, Navigant Consulting, Spherix, MedIQ, UBM, Trio Health, Medscape, WebMD, Practice Point Communications, Simply Speaking, the US National Institutes of Health, and the American College of Rheumatology; currently or previously owning stock options in TPT Global Tech, Vaxart Pharmaceuticals, Charlotte's Web Holdings, Amarin, Viking, and Moderna; and membership with OMERACT, an international organisation that develops measures for clinical trials and receives arm's length funding from 12 pharmaceutical companies, the US Food and Drug Administration Arthritis Advisory Committee, the Veterans Affairs Rheumatology Field Advisory Committee, and the University of Alabama at Birmingham Cochrane Musculoskeletal Group Satellite Center on Network Meta-analysis. A C Tsai reports personal fees from Elsevier and the Public Library of Science, outside the submitted work. All other authors declare no competing interests.
