# Supplementary material for: Subnational mapping of HIV incidence and mortality among individuals aged 15–49 years in sub-Saharan Africa, 2000–18: a modelling study
Source: Lancet HIV. 2021 Jun 1;8(6):e363–75. doi: 10.1016/S2352-3018(21)00051-5 (PMC8187986; doi:10.1016/S2352-3018(21)00051-5)
Supplement: Supplementary appendix [file mmc1.pdf]

THE LANCET
HIV

Supplementary appendix

This appendix formed part of the original submission and has been peer reviewed. We post it as supplied by the authors.

Supplement to: Local Burden of Disease HIV Collaborators. Subnational mapping of HIV incidence and mortality among individuals aged 15–49 years in sub-Saharan Africa, 2000–18: a modelling study. *Lancet HIV* 2021; **8:** e363–75.

Appendix:

Subnational mapping of HIV incidence and mortality among individuals aged 15 to 49 in sub-Saharan Africa, 2000-18: a modelling study

Contents

1 LBD HIV Incidence Mortality Collaborators 1

2 Author Contributions 13

3 Compliance with the Guidelines for Accurate and Transparent Health Estimates Reporting (GATHER) 18

4 Methods Appendix 20

4.1 Epidemiological model 20

4.1.1 Model overview 20

4.1.2 Initiation of random model components 22

4.1.3 Fitting 23

4.1.4 Disease progression and mortality calculations 24

4.1.5 Final posterior creation and post estimation 24

4.1.6 Summary of changes from previous versions of EPP 25

4.2 Data inputs 26

4.2.1 Administrative boundaries and demographic data 26

4.2.2 HIV prevalence and PLHIV for fitting 26

4.2.3 HIV treatment data 27

Appendix Figures 30

Appendix Figure 1: EPP schematic 30

Appendix Figure 2: Impact of subnational ART data on HIV incidence estimates 31

Appendix Figure 3: Impact of subnational ART data on HIV mortality estimates 32

Appendix Figure 4: Incidence, mortality, prevalence, and ART coverage by second-level administrative unit in 2000 33

Appendix Figure 5: Incidence, mortality, prevalence, and ART coverage by second-level administrative unit in 2005 34

Appendix Figure 6: Incidence, mortality, prevalence, and ART coverage by second-level administrative unit in 2010 35

Appendix Figure 7: Incidence, mortality, prevalence, and ART coverage by second-level administrative unit in 2018 36

Appendix Figure 8: HIV incidence rate among individuals aged 15–49 in 2018 with uncertainty 37

Appendix Figure 9: HIV mortality rate among individuals aged 15–49 in 2018 with uncertainty 39

Appendix Figure 10: Percent reduction in the HIV incidence rate from 2010 to 2018 41

Appendix Figure 11: Percent reduction in the HIV mortality rate from 2010 to 2018 42

Appendix Figure 12: Posterior probability of a 75% or greater reduction in incident HIV cases from 2010 to 2018 43

Appendix Figure 13: Posterior probability of an increase in incident HIV cases from 2010 to 2018 44
Appendix Figure 14: Posterior probability of a 75% or greater reduction in HIV deaths from 2010 to 2018 45
Appendix Figure 15: Posterior probability of an increase in HIV deaths from 2010 to 2018 46
Appendix Figure 16: Posterior probability that the ratio of incident HIV cases to people living with HIV was less than 0.03 in 2018 47
Appendix Tables 48
Appendix Table 1a: Impact of down sampling HIV mortality and CD4 progression parameters on mean estimate variability. 48
Appendix Table 1b: Impact of down sampling HIV mortality and CD4 progression parameters on uncertainty interval width variability. 48
Appendix Table 2: Edits to GADM shapes 49
Appendix Table 3: Geographic administrative units 50
Appendix Table 4: HIV seroprevalence survey data 52
Appendix Table 5: ANC sentinel surveillance data 82
Appendix Table 6: HIV covariate survey data 102
Appendix Table 7: HIV treatment administrative data 201
Appendix Table 8: ART model parameters 204
References 205

1 LBD HIV Incidence Mortality Collaborators

LBD HIV Incidence Mortality Collaborators

Benn Sartorius, John D VanderHeide, Mingyou Yang, Erik A Goosmann, Julia Hon, Emily Haeuser, Michael A Cork, Samantha Perkins, Deepa Jahagirdar, Lauren E Schaeffer, Audrey L Serfes, Kate E LeGrand, Hedayat Abbastabar, Zeleke Hailemariam Abebo, Akine Eshete Abosetugn, Eman Abu-Gharbieh, Manfred Mario Kokou Accrombessi, Oladimeji M Adebayo, Adeyinka Emmanuel Adegbosin, Victor Adekanmbi, Olatunji O Adetokunboh, Daniel Adedayo Adeyinka, Bright Opoku Ahinkorah, Keivan Ahmadi, Muktar Beshir Ahmed, Yonas Akalu, Oluwaseun Oladapo Akinyemi, Rufus Olusola Akinyemi, Addis Aklilu, Chisom Joyqueenet Akunna, Fares Alahdab, Ziyad Al-Aly, Noore Alam, Alehegn Aderaw Alamneh, Turki M Alanzi, Biresaw Wassihun Alemu, Robert Kaba Alhassan, Tilahun Ali, Vahid Alipour, Saeed Amini, Robert Ancuceanu, Fereshteh Ansari, Zelalem Alamrew Anteneh, Davood Anvari, Razique Anwer, Seth Christopher Yaw Appiah, Jalal Arabloo, Mulusew A Asemahagn, Mohammad Asghari Jafarabadi, Wondwossen Niguse Asmare, Desta Debalkie Atnafu, Maha Moh'd Wahbi Atout, Alok Atreya, Marcel Ausloos, Atalel Fentahun Awedew, Beatriz Paulina Ayala Quintanilla, Martin Amogre Ayanore, Yared Asmare Aynalem, Muluken Altaye Ayza, Samad Azari, Zelalem Nigussie Azene, Zaheer-Ud-Din Babar, Atif Amin Baig, Senthilkumar Balakrishnan, Maciej Banach, Till Winfried Bärnighausen, Sanjay Basu, Mohsen Bayati, Neeraj Bedi, Tariku Tesfaye Bekuma, Woldesellassie M Mequanint Bezabhe, Akshaya Srikanth Bhagavathula, Pankaj Bhardwaj, Krittika Bhattacharyya, Zulfiqar A Bhutta, Sadia Bibi, Boris Bikbov, Tsegaye Adane Birhan, Zebenay Workneh Bitew, Moses John Bockarie, Archith Boloor, Oliver J Brady, Nicola Luigi Bragazzi, Andrey Nikolaevich Briko, Nikolay Ivanovich Briko, Sharath Burugina Nagaraja, Zahid A Butt, Rosario Cárdenas, Felix Carvalho, Jaykaran Charan, Souranshu Chatterjee, Soosanna Kumary Chattu, Vijay Kumar Chattu, Mohiuddin Ahsanul Kabir Chowdhury, Dinh-Toi Chu, Aubrey J Cook, Natalie Maria Cormier, Richard G Cowden, Carlos Culquichicon, Baye Dagnew, Saad M A Dahlawi, Giovanni Damiani, Parnaz Daneshpajouhnejad, Farah Daoud, Ahmad Daryani, José das Neves, Nicole Davis Weaver, Meseret Derbew Molla, Kebede Deribe, Abebaw Alemayehu Desta, Keshab Deuba, Samath Dhamminda Dharmaratne, Govinda Prasad Dhungana, Daniel Diaz, Shirin Djalalinia, Paul Narh Doku, Eleonora Dubljanin, Bereket Duko, Arielle Wilder Eagan, Lucas Earl, Jeffrey W Eaton, Andem Effiong, Maysaa El Sayed Zaki, Maha El Tantawi, Rajesh Elayedath, Shaimaa I El-Jaafary, Aisha Elsharkawy, Sharareh Eskandarieh, Oghenowede Eyawo, Sayeh Ezzikouri, Abidemi Omolara Fasanmi, Alebachew Fasil, Nelsensius Klau Fauk, Valery L Feigin, Tomas Y Ferede, Eduarda Fernandes, Florian Fischer, Nataliya A Foigt, Morenike Oluwatoyin Folayan, Masoud Foroutan, Joel Msafiri Francis, Takeshi Fukumoto, Mohamed M Gad, Biniyam Sahiledengle Geberemariyam, Birhan Gebresillassie Gebregiorgis, Berhe Gebremichael, Hailay Abrha Gesesew, Lemma Getacher, Keyghobad Ghadiri, Ahmad Ghashghaee, Syed Amir Gilani, Themba G Ginindza, Mustefa Glagn, Mahaveer Golechha, Philimon N Gona, Mohammed Ibrahim Mohialdeen Gubari, Harish Chander Gugnani, Davide Guido, Rashid Abdi Guled, Brian J Hall, Samer Hamidi, Demelash Woldeyohannes Handiso, Arief Hargono, Abdiwahab Hashi, Soheil Hassanipour, Hadi Hassankhani, Khezar Hayat, Claudiu Herteliu, Hagos Degefa de Hidru, Ramesh Holla, H Dean Hosgood, Naznin Hossain, Mostafa Hosseini, Mehdi Hosseinzadeh, Mowafa Househ, Bing-Fang Hwang, Segun Emmanuel Ibitoye, Olayinka Stephen Ilesanmi, Irena M Ilic, Milena D Ilic, Seyed Sina Naghibi Irvani, Chidozie C D Iwu, Chinwe Juliana Iwu, Ihoghosa Osamuyi Iyamu, Vardhmaan Jain, Mihajlo Jakovljevic, Farzad Jalilian, Ravi Prakash Jha, Kimberly B Johnson, Vasna Joshua, Farahnaz Joukar, Jacek Jerzy Jozwiak, Ali Kabir, Leila R Kalankesh, Rohollah Kalhor, Ashwin Kamath, Naser Kamyari, Tanuj Kanchan, Behzad Karami Matin, André Karch, Salah Eddin Karimi, Ayele Semachew Kasa, Getinet Kassahun, Gbenga A Kayode, Ali Kazemi Karyani, Peter Njenga Keiyoro, Bayew Kelkay, Nauman Khalid, Gulfaraz Khan, Junaid Khan, Md Nuruzzaman Khan, Khaled Khatab, Salman Khazaei, Yun Jin Kim, Adnan

Kisa, Sezer Kisa, Sonali Kochhar, Jacek A Kopec, Soewarta Kosen, Sindhura Lakshmi Koulmane Laxminarayana, Ai Koyanagi, Kewal Krishan, Barthelemy Kuate Defo, Nuworza Kugbey, Vaman Kulkarni, Manasi Kumar, Nithin Kumar, Om P Kurmi, Dian Kusuma, Desmond Kuupiel, Hmwe Hmwe Kyu, Carlo La Vecchia, Dharmesh Kumar Lal, Jennifer O Lam, Iván Landires, Savita Lasrado, Jeffrey V Lazarus, Alice Lazzar-Atwood, Paul H Lee, Cheru Tesema Leshargie, Bingyu Li, Xuefeng Liu, Platon D Lopukhov, Hawraz Ibrahim M. Amin, Deepak Madi, Phetole Walter Mahasha, Azeem Majeed, Afshin Maleki, Shokofeh Maleki, Abdullah A Mamun, Navid Manafi, Mohammad Ali Mansournia, Francisco Rogerlândio Martins-Melo, Seyedeh Zahra Masoumi, Benjamin K Mayala, Birhanu Geta Meharie, Hailemariam Abiy Alemu Meheretu, Hagazi Gebre Meles, Mulugeta Melku, Walter Mendoza, Endalkachew Worku Mengesha, Tuomo J Meretoja, Abera M Mersha, Tomislav Mestrovic, Ted R Miller, Andreea Mirica, Mehdi Mirzaei-Alavijeh, Osama Mohamad, Yousef Mohammad, Abdollah Mohammadian-Hafshejani, Jemal Abdu Mohammed, Salahuddin Mohammed, Shafiu Mohammed, Ali H Mokdad, Taklu Marama Mokonnon, Mariam Molokhia, Masoud Moradi, Yousef Moradi, Rahmatollah Moradzadeh, Paula Moraga, Jonathan F Mosser, Sandra B Munro, Ghulam Mustafa, Saravanan Muthupandian, Mehdi Naderi, Ahamarshan Jayaraman Nagarajan, Mohsen Naghavi, Muhammad Naveed, Vinod C Nayak, Javad Nazari, Rawlance Ndejjo, Samata Nepal, Henok Biresaw Netsere, Frida N Ngalesoni, Georges Nguefack-Tsague, Josephine W Ngunjiri, Yeshambel T Nigatu, Samuel Negash Nigussie, Chukwudi A Nnaji, Jean Jacques Noubiap, Virginia Nuñez-Samudio, Bogdan Oancea, Oluwakemi Ololade Odukoya, Felix Akpojene Ogbo, Olanrewaju Oladimeji, Andrew T Olagunju, Bolajoko Olubukunola Olusanya, Jacob Olusegun Olusanya, Muktar Omer Omer, Abidemi E Emmanuel Omonisi, Obinna E Onwujekwe, Orish Ebere Orisakwe, Nikita Otstavnov, Mayowa O Owolabi, Mahesh P A, Jagadish Rao Padubidri, Smita Pakhale, Adrian Pana, Seithikurippu R Pandi-Perumal, Urvish K Patel, Mona Pathak, George C Patton, Shrikant Pawar, Emmanuel K Peprah, Khem Narayan Pokhrel, Maarten J Postma, Faheem Hyder Pottoo, Hadi Pourjafar, Dimas Ria Angga Pribadi, Zahiruddin Quazi Syed, Alireza Rafiei, Fakher Rahim, Mohammad Hifz Ur Rahman, Amir Masoud Rahmani, Pradhum Ram, Juwel Rana, Chhabi Lal Ranabhat, Satish Rao, Sowmya J Rao, Priya Rathi, David Laith Rawaf, Salman Rawaf, Reza Rawassizadeh, Vishnu Renjith, Melese Abate Reta, Nima Rezaei, Aziz Rezapour, Ana Isabel Ribeiro, Jennifer M Ross, Susan Fred Rumisha, Rajesh Sagar, Maitreyi Sahu, S. Mohammad Sajadi, Marwa Rashad Salem, Abdallah M Samy, Brijesh Sathian, Aletta Elisabeth Schutte, Abdul-Aziz Seidu, Feng Sha, Omid Shafaat, Mohammad Shahbaz, Masood Ali Shaikh, Mohammed Feyisso Shaka, Aziz Sheikh, Kenji Shibuya, Jae Il Shin, K M Shivakumar, Negussie Boti Sidemo, Jasvinder A Singh, Valentin Yurievich Skryabin, Anna Aleksandrovna Skryabina, Amin Soheili, Shahin Soltani, Oluwaseyi Dolapo Somefun, Muluken Bekele Sorrie, Emma Elizabeth Spurlock, Mu'awiyyah Babale Sufiyan, Biruk Wogayehu Taddele, Eyayou Girma Tadesse, Zemenu Tamir, Animut Tagele Tamiru, Frank C Tanser, Nuno Taveira, Arash Tehrani-Banihashemi, Yohannes Tekalegn, Fisaha Haile Tesfay, Belay Tessema, Zemenu Tadesse Tessema, Bhaskar Thakur, Musliu Adetola Tolani, Roman Topor-Madry, Marco Torrado, Marcos Roberto Tovani-Palone, Eugenio Traini, Alexander C Tsai, Gebiyaw Wudie Tsegaye, Irfan Ullah, Saif Ullah, Chukwuma David Umeokonkwo, Bhaskaran Unnikrishnan, Constantine Vardavas, Francesco S Violante, Bay Vo, Yohannes Dibaba Wado, Yasir Waheed, Richard G Wamai, Yanzhong Wang, Paul Ward, Andrea Werdecker, Nuwan Darshana Wickramasinghe, Tissa Wijeratne, Charles Shey Wiysonge, Temesgen Gebeyehu Wondmeneh, Tomohide Yamada, Sanni Yaya, Yigizie Yeshaw, Yordanos Gizachew Yeshitila, Mekdes Tigistu Yilma, Paul Yip, Naohiro Yonemoto, Tewodros Yosef, Hasan Yusefzadeh, Syed Saoud Zaidi, Leila Zaki, Maryam Zamanian, Mikhail Sergeevich Zastrozhin, Anasthasia Zastrozhina, Dejene Tesfaye Zewdie, Yunquan Zhang, Zhi-Jiang Zhang, Arash Ziapour, Simon I Hay, and Laura Dwyer-Lindgren.

Affiliations

Centre for Tropical Medicine and Global Health, Nuffield Department of Medicine (B Sartorius PhD), Malaria Atlas Project (S F Rumisha PhD), The George Institute for Global Health (Prof S Yaya PhD),

University of Oxford, Oxford, UK; Department of Health Metrics Sciences, School of Medicine (B Sartorius PhD, Prof S D Dharmaratne MD, H H Kyu PhD, Prof A H Mokdad PhD, Prof M Naghavi MD, Prof S I Hay FMedSci, L Dwyer-Lindgren PhD), Institute for Health Metrics and Evaluation (M Yang BS, E A Goosmann MS, J Hon MLS, E Haeuser PhD, M A Cork MPH, S Perkins MPH, D Jahagirdar PhD, A L Serfes P.S.M, K E LeGrand MPH, A J Cook MSPH, N M Cormier MPSA, F Daoud BS, N Davis Weaver MPH, Prof S D Dharmaratne MD, L Earl MSc, J W Eaton PhD, Prof V L Feigin PhD, K B Johnson MS, H H Kyu PhD, A Lazzar-Atwood BSc, B K Mayala PhD, Prof A H Mokdad PhD, J F Mosser MD, S B Munro PhD, Prof M Naghavi MD, J M Ross MD, M Sahu MS, E E Spurlock BA, Prof S I Hay FMedSci, L Dwyer-Lindgren PhD), Department of Global Health (S Kochhar MD, J M Ross MD), Department of Medicine (J M Ross MD), University of Washington, Seattle, WA, USA; Insights Program (J D VanderHeide MSc), Bill & Melinda Gates Foundation, Seattle, WA, USA; Global Programs (L E Schaeffer MSc), Medical Teams International, Seattle, WA, USA; Department of Pediatric Newborn Medicine (L E Schaeffer MSc), Brigham and Women's Hospital, Boston, MA, USA; Advanced Diagnostic and Interventional Radiology Research Center (H Abbastabar PhD), Multiple Sclerosis Research Center (S Eskandarieh PhD), Department of Epidemiology and Biostatistics (Prof M Hosseini PhD, M Mansournia PhD), Pediatric Chronic Kidney Disease Research Center (Prof M Hosseini PhD), Department of Environmental Health Engineering (Prof A Maleki PhD), Metabolomics and Genomics Research Center (F Rahim PhD), Research Center for Immunodeficiencies (Prof N Rezaei PhD), Tehran University of Medical Sciences, Tehran, Iran; Department of Public Health (Z H Abebo MSc, M Glagn MPH, N B Sidemo MPH, M B Sorrie MPH), Department of Medical Laboratory Sciences (A Aklilu MSc), College of Medicine and Health Science (B W Alemu MSc), Department of Nursing (A M Mersha MSc, Y G Yeshitila MSc), Department of Biomedical Sciences (E G Tadesse MSc), Arba Minch University, Arba Minch, Ethiopia; Department of Public Health (A E Abosetugn MPH), Debre Berhan University, Debre Brehan, Ethiopia; Department of Clinical Sciences (E Abu-Gharbieh PhD), University of Sharjah, Sharjah, United Arab Emirates; Department of Disease Control (M M K Accrombessi PhD), Department of Infectious Disease Epidemiology (O J Brady PhD), London School of Hygiene & Tropical Medicine, London, UK; Department of Clinical Research (M M K Accrombessi PhD), Clinical Research Institute of Benin (IRCB), Abomey-Calavi, Benin; College of Medicine (O M Adebayo MD), Department of Health Policy and Management (O O Akinyemi FWACP), Department of Community Medicine (O S Ilesanmi PhD), Department of Medicine (Prof M O Owolabi DrM), University College Hospital, Ibadan, Ibadan, Nigeria; School of Medicine (A E Adegbosin MD), Griffith University, Gold Coast, QLD, Australia; Department of Population Health Sciences (V Adekanmbi PhD), Faculty of Life Sciences and Medicine (M Molokhia PhD), Institute for Population Health (Prof K Shibuya MD), School of Population Health and Environmental Sciences (Y Wang PhD), King's College London, London, UK; Centre of Excellence for Epidemiological Modelling and Analysis (O O Adetokunboh PhD), Stellenbosch University, Stellenbosch, South Africa; Department of Global Health (O O Adetokunboh PhD, C J Iwu PhD), Stellenbosch University, Cape Town, South Africa; Department of Community Health and Epidemiology (D A Adeyinka MPH), University of Saskatchewan, Saskatoon, SK, Canada; Department of Public Health (D A Adeyinka MPH), Federal Ministry of Health, Abuja, Nigeria; The Australian Centre for Public and Population Health Research (ACPPHR) (B O Ahinkorah MPH), University of Technology Sydney, Sydney, NSW, Australia; Lincoln Medical School (K Ahmadi PhD), Universities of Nottingham & Lincoln, Lincoln, UK; Department of Epidemiology (M B Ahmed MPH), Jimma University, Jimma, Ethiopia; Australian Center for Precision Health (M B Ahmed MPH), University of South Australia, Adelaide, SA, Australia; Department of Medical Physiology (Y Akalu MSc), Department of Reproductive Health (Z N Azene MPH), Department of Environmental and Occupational Health and Safety (T A Birhan MSc), Department of Human Physiology (B Dagnew MSc), Department of Biochemistry (M Derbew Molla MSc), Department of Surgical Nursing (A A Desta MSc), Department of Clinical Chemistry (A Fasil MSc), Department of Midwifery (B Kelkay MSc, A T Tamiru MSc), Department of Hematology and Immunohematology (M Melku MSc), School of Nursing (H B Netsere MS), Department of Medical

Microbiology (B Tessema PhD), Department of Epidemiology and Biostatistics (Z T Tessema MSc, Y Yeshaw MPH), University of Gondar, Gondar, Ethiopia; Department of Health Policy and Management (O O Akinyemi FWACP), Institute for Advanced Medical Research and Training (R O Akinyemi PhD), Department of Health Promotion and Education (S E Ibitoye MPH), Department of Community Medicine (O S Ilesanmi PhD), Department of Medicine (Prof M O Owolabi DrM), University of Ibadan, Ibadan, Nigeria; Institute of Neuroscience (R O Akinyemi PhD), Newcastle University, Newcastle upon Tyne, UK; Department of Public Health (C J Akunna DMD), The Intercountry Centre for Oral Health (ICOH) for Africa, Jos, Nigeria; Department of Public Health (C J Akunna DMD), Federal Ministry of Health, Garki, Nigeria; Mayo Evidence-based Practice Center (F Alahdab MSc), Mayo Clinic Foundation for Medical Education and Research, Rochester, MN, USA; John T. Milliken Department of Internal Medicine (Z Al-Aly MD), Washington University in St. Louis, St. Louis, MO, USA; Clinical Epidemiology Center (Z Al-Aly MD), Department of Veterans Affairs, St Louis, MO, USA; Prevention Division (N Alam MPH), Queensland Health, Brisbane, QLD, Australia; Centre for Environment and Population Health (N Alam MPH), Griffith University, Nathan, QLD, Australia; Department of Human Nutrition and Food Sciences (A A Alamneh MPH), Department of Public Health (H A A Meheretu MPH), Debre Markos University, Debre Markos, Ethiopia; Health Information Management and Technology Department (T M Alanzi PhD), Environmental Health Department (S M A Dahlawi PhD), Department of Pharmacology (F H Pottoo PhD), Imam Abdulrahman Bin Faisal University, Dammam, Saudi Arabia; Department of Midwifery (B W Alemu MSc), Arba Minch University, Injbara, Ethiopia; Institute of Health Research (R K Alhassan PhD), Department of Health Policy Planning and Management (M A Ayanore PhD), Department of Family and Community Health (N Kugbey PhD), University of Health and Allied Sciences, Ho, Ghana; Department of Psychiatry (T Ali MSc), Department of Medical Microbiology (S Balakrishnan PhD), School of Public Health (B Gebremichael MPH), Department of Psychiatric Nursing (D T Zewdie MSc), Haramaya University, Harar, Ethiopia; Health Management and Economics Research Center (V Alipour PhD, J Arabloo PhD, S Azari PhD, A Ghashghaee BSc, M Hosseinzadeh PhD, A Rezapour PhD), Health Economics Department (V Alipour PhD), Student Research Committee (A Ghashghaee BSc), Minimally Invasive Surgery Research Center (A Kabir MD), School of Medicine (N Manafi MD), Preventive Medicine and Public Health Research Center (A Tehrani-Banihashemi PhD), Department of Community and Family Medicine (A Tehrani-Banihashemi PhD), Iran University of Medical Sciences, Tehran, Iran; Health Services Management Department (S Amini PhD), Department of Epidemiology (R Moradzadeh PhD, M Zamanian PhD), Department of Pediatrics (J Nazari MD), Arak University of Medical Sciences, Arak, Iran; Pharmacy Department (Prof R Ancuceanu PhD), Carol Davila University of Medicine and Pharmacy, Bucharest, Romania; Research Center for Evidence Based Medicine (F Ansari PhD), Department of Biostatistics and Epidemiology (Prof M Asghari Jafarabadi PhD), School of Nursing and Midwifery (H Hassankhani PhD), School of Management and Medical Informatics (L R Kalankesh PhD), Social Determinants of Health Research Center (S Karimi PhD), Tabriz University of Medical Sciences, Tabriz, Iran; Razi Vaccine and Serum Research Institute (F Ansari PhD), Agricultural Research, Education, and Extension Organization (AREEO), Tehran, Iran; Department of Epidemiology (Z A Anteneh MPH), School of Public Health (M A Asemahagn PhD, H A A Meheretu MPH), Department of Health System and Health Economics (D D Atnafu MPH), Department of Adult Health Nursing (A Kasa MSc), Department of Reproductive Health and Population Studies (E W Mengesha MPH), College of Medicine and Health Sciences (G W Tsegaye MPH), Bahir Dar University, Bahir Dar, Ethiopia (W M M Bezabhe BSc); Department of Parasitology (D Anvari PhD), Toxoplasmosis Research Center (Prof A Daryani PhD), Department of Immunology (Prof A Rafiei PhD), Molecular and Cell Biology Research Center (Prof A Rafiei PhD), Mazandaran University of Medical Sciences, Sari, Iran; Department of Parasitology (D Anvari PhD), Iranshahr University of Medical Sciences, Iranshahr, Iran; Department of Pathology (R Anwer PhD), Imam Mohammad Ibn Saud Islamic University, Riyadh, Saudi Arabia; Department of Sociology and Social Work (S Appiah PhD), Kwame Nkrumah University of Science and Technology, Kumasi, Ghana; Center for International Health (S

Appiah PhD), Ludwig Maximilians University, Munich, Germany; Department of Biostatistics and Epidemiology (Prof M Asghari Jafarabadi PhD), Zanjan University of Medical Sciences, Zanjan, Iran; Department of Nursing (W N Asmare MSc), Department of Public Health (S N Nigussie MPH, T Yosef MPH), Mizan-Tepi University, Mizan Teferi, Ethiopia; Faculty of Nursing (M M W Atout PhD), Philadelphia University, Amman, Jordan; Department of Forensic Medicine (A Atreya MD), Lumbini Medical College, Palpa, Nepal; School of Business (Prof M Ausloos PhD), Department of Health Sciences (P H Lee PhD), University of Leicester, Leicester, UK; Department of Statistics and Econometrics (Prof M Ausloos PhD, Prof C Herteliu PhD, A Mirica PhD, A Pana MD), Bucharest University of Economic Studies, Bucharest, Romania; Department of Surgery (A F Awedew MD), School of Public Health (K Deribe PhD), Department of Medical Laboratory Science (Z Tamir MSc), Addis Ababa University, Addis Ababa, Ethiopia; The Judith Lumley Centre (B Ayala Quintanilla PhD), La Trobe University, Melbourne, VIC, Australia; Department of Nursing (Y A Aynalem MSc, B G Gebregiorgis MSc), Department of Public Health (L Getacher MPH), Debre Berhan University, Debre Berhan, Ethiopia; Department of Pharmacology and Toxicology (M A Ayza MSc), Department of Epidemiology (H A Gesesew PhD), Department of Microbiology and Immunology (S Muthupandian PhD), School of Public Health (F H Tesfay PhD), Mekelle University, Mekelle, Ethiopia (H G Meles MPH); Department of Pharmacy (Prof Z Babar PhD), University of Huddersfield, Huddersfield, UK; Unit of Biochemistry (A A Baig PhD), Universiti Sultan Zainal Abidin (Sultan Zainal Abidin University), Kuala Terengganu, Malaysia; Department of Hypertension (Prof M Banach PhD), Medical University of Lodz, Lodz, Poland; Polish Mothers' Memorial Hospital Research Institute, Lodz, Poland (Prof M Banach PhD); Heidelberg Institute of Global Health (HIGH) (Prof T W Bärnighausen MD, S Mohammed PhD), Heidelberg University, Heidelberg, Germany; T.H. Chan School of Public Health (Prof T W Bärnighausen MD), Center for Primary Care (S Basu PhD), Department of Global Health and Social Medicine (A W Eagan MSW), Division of General Internal Medicine (Prof A Sheikh MD), Harvard University, Boston, MA, USA; School of Public Health (S Basu PhD), Department of Infectious Disease Epidemiology (J W Eaton PhD), Imperial College Business School (D Kusuma DSc), Department of Primary Care and Public Health (Prof A Majeed MD, Prof S Rawaf MD), WHO Collaborating Centre for Public Health Education and Training (D L Rawaf MD), Imperial College London, London, UK; Health Human Resources Research Center (M Bayati PhD), Shiraz University of Medical Sciences, Shiraz, Iran; School of Public Health (Prof N Bedi MD), Dr. D. Y. Patil University, Mumbai, India; Jazan University, Jazan, Saudi Arabia (Prof N Bedi MD); Department of Public Health (T T Bekuma MPH, M T Yilma MPH), Wollega University, Nekemte, Ethiopia; University of Tasmania, Australia (W M M Bezabhe BSc); Department of Social and Clinical Pharmacy (A S Bhagavathula PharmD), Charles University, Hradec Kralova, Czech Republic; Institute of Public Health (A S Bhagavathula PharmD), Department of Medical Microbiology & Immunology (Prof G Khan PhD), United Arab Emirates University, Al Ain, United Arab Emirates; Department of Community Medicine and Family Medicine (P Bhardwaj MD), School of Public Health (P Bhardwaj MD), Department of Pharmacology (J Charan MD), Department of Forensic Medicine and Toxicology (T Kanchan MD), All India Institute of Medical Sciences, Jodhpur, India; Department of Statistical and Computational Genomics (K Bhattacharyya MSc), National Institute of Biomedical Genomics, Kalyani, India; Department of Statistics (K Bhattacharyya MSc), University of Calcutta, Kolkata, India; Centre for Global Child Health (Prof Z A Bhutta PhD), Department of Medicine (V Chattu MD), University of Toronto, Toronto, ON, Canada; Centre of Excellence in Women & Child Health (Prof Z A Bhutta PhD), Aga Khan University, Karachi, Pakistan; Institute of Soil and Environmental Sciences (S Bibi PhD), University of Agriculture - Faisalabad, Faisalabad, Pakistan; Mario Negri Institute for Pharmacological Research, Ranica, Italy (B Bikbov MD); Nutrition Department (Z W Bitew MPH), St. Paul's Hospital Millennium Medical College, Addis Ababa, Ethiopia (Z W Bitew MPH); European & Developing Countries Clinical Trials Partnership, Cape Town, South Africa (Prof M J Bockarie MSc); Department of Medicine (Prof M J Bockarie MSc), School of Public Health and Family Medicine (C A Nnaji MPH, Prof C S Wiysonge MD), University of Cape Town, Cape Town, South Africa; Department of

Internal Medicine (A Boloor MD, D Madi MD), Department of Community Medicine (V Kulkarni MD, N Kumar MD), Department of Infectious Disease (Prof S Rao MD), Kasturba Medical College (Prof B Unnikrishnan MD), Manipal Academy of Higher Education, Mangalore, India; University of Genoa, Genoa, Italy (N L Bragazzi PhD); Department of Biomedical Technologies (A N Briko MSc), Bauman Moscow State Technical University, Moscow, Russia; Department of Epidemiology and Evidence Based Medicine (Prof N I Briko DSc, P D Lopukhov Cand. of Sci. (Med.)), I.M. Sechenov First Moscow State Medical University, Moscow, Russia; Department of Community Medicine (Prof S Burugina Nagaraja MD), Employee State Insurance Post Graduate Institute of Medical Sciences and Research, Bangalore, India; School of Public Health and Health Systems (Z A Butt PhD), University of Waterloo, Waterloo, ON, Canada; Al Shifa School of Public Health (Z A Butt PhD), Al Shifa Trust Eye Hospital, Rawalpindi, Pakistan; Department of Health Care (Prof R Cárdenas DSc), Metropolitan Autonomous University, Mexico City, Mexico; Research Unit on Applied Molecular Biosciences (UCIBIO) (Prof F Carvalho PhD), Institute for Research and Innovation in Health (J das Neves PhD), Institute of Biomedical Engineering (INEB) (J das Neves PhD), Associated Laboratory for Green Chemistry (LAQV) (Prof E Fernandes PhD), Epidemiology Research Unit Institute of Public Health (EPIUnit-ISPUP) (A Ribeiro PhD), University of Porto, Porto, Portugal; Department of Microbiology & Infection Control (S Chatterjee MD), Medanta Medicity, Gurugram, India; Department of Public Health (S Chattu PhD), Texila American University, Georgetown, Guyana; Global Institute of Public Health (GIPH), Thiruvananthapuram, India (V Chattu MD); Maternal and Child Health Division (M Chowdhury MPH), International Centre for Diarrhoeal Disease Research, Bangladesh, Dhaka, Bangladesh; Department of Epidemiology and Biostatistics (M Chowdhury MPH), University of South Carolina, Columbia, SC, USA; Center for Biomedicine and Community Health (D Chu PhD), VNU-International School, Hanoi, Vietnam; Department of Psychology (R G Cowden PhD), University of the Free State, Park West, South Africa; Research Unit and Training Consortium on Emerging Diseases and Climate Change (Emerge) (C Culquichicon MD), Peruvian University Cayetano Heredia, Lima, Peru; IRCCS Istituto Ortopedico Galeazzi (Galeazzi Orthopedic Institute IRCCS) (G Damiani MD), Department of Clinical Sciences and Community Health (Prof C La Vecchia MD), University of Milan, Milan, Italy; Department of Dermatology (G Damiani MD), Case Western Reserve University, Cleveland, OH, USA; Department of Pathology (P Daneshpajouhnejad MD), Johns Hopkins University School of Medicine, Baltimore, MD, USA; Department of Pathology (P Daneshpajouhnejad MD), Department of Radiology and Interventional Neuroradiology (O Shafaat MD), Isfahan University of Medical Sciences, Isfahan, Iran; Wellcome Trust Brighton and Sussex Centre for Global Health Research (K Deribe PhD), Brighton and Sussex Medical School, Brighton, UK; National Centre for AIDS and STD Control (K Deuba DrPH), Save the Children, Kathmandu, Nepal; Department of Global Public Health (K Deuba DrPH), Karolinska Institute, Stockholm, Sweden; Department of Community Medicine (Prof S D Dharmaratne MD), University of Peradeniya, Peradeniya, Sri Lanka; Department of Microbiology (G P Dhungana MSc), Far Western University, Mahendranagar, Nepal; Center of Complexity Sciences (Prof D Diaz PhD), National Autonomous University of Mexico, Mexico City, Mexico; Faculty of Veterinary Medicine and Zootechnics (Prof D Diaz PhD), Autonomous University of Sinaloa, Culiacán Rosales, Mexico; Development of Research and Technology Center (S Djalalinia PhD), Ministry of Health and Medical Education, Tehran, Iran; School of Nursing and Midwifery (P N Doku PhD), Department of Population and Health (A Seidu MPhil), University of Cape Coast, Cape Coast, Ghana; Institute of Microbiology and Immunology (E Dubljanin PhD), Faculty of Medicine (I M Ilic PhD), University of Belgrade, Belgrade, Serbia; School of Public Health (B Duko MPH), School of Nursing (T Y Ferede MSc), School of Midwifery (G Kassahun MSc), Hawassa University, Hawassa, Ethiopia; School of Public Health (B Duko MPH, T R Miller PhD), Curtin University, Perth, WA, Australia; Department of Social Services (A W Eagan MSW), Tufts Medical Center, Boston, MA, USA; Centre Clinical Epidemiology and Biostatistics (A Effiong MB), University of Newcastle, Newcastle, NSW, Australia; Clinical Pathology Department (Prof M El Sayed Zaki PhD), Mansoura Faculty of Medicine, Mansoura, Egypt; Pediatric Dentistry and Dental

Public Health Department (Prof M El Tantawi PhD), Alexandria University, Alexandria, Egypt; School of Behavioural Sciences (R Elayedath MSc), Mahatma Gandhi University of Medical Sciences and Technology, Kottayam, India; Department of Neurology (S I El-Jaafary MD), Endemic Medicine and Hepatogastroentrology Department (A Elsharkawy MD), Cairo University, Cairo, Egypt; Faculty of Health (O Eyawo PhD), York University, Toronto, BC, Canada; Department of Virology (S Ezzikouri PhD), Pasteur Institute of Morocco, Casablanca, Morocco; Satcher Health Leadership Institute (A O Fasanmi PhD), Morehouse School of Medicine, Atlanta, GA, USA; School of Medicine (A O Fasanmi PhD), Department of Cardiology (P Ram MD), Emory University, Atlanta, GA, USA; College of Medicine and Public Health (N K Fauk MSc, H A Gesesew PhD, Prof P Ward PhD), Southgate Institute for Health and Society (F H Tesfay PhD), Flinders University, Adelaide, SA, Australia; Institute of Resource Governance and Social Change, Kupang, Indonesia (N K Fauk MSc); National Institute for Stroke and Applied Neurosciences (Prof V L Feigin PhD), Auckland University of Technology, Auckland, New Zealand; Research Center of Neurology, Moscow, Russia (Prof V L Feigin PhD); Institute of Gerontological Health Services and Nursing Research (F Fischer PhD), Ravensburg-Weingarten University of Applied Sciences, Weingarten, Germany; Institute of Gerontology (N A Foigt PhD), National Academy of Medical Sciences of Ukraine, Kyiv, Ukraine; Department of Child Dental Health (Prof M O Folayan FWACS), Obafemi Awolowo University, Ile-Ife, Nigeria; Department of Medical Parasitology (M Foroutan PhD), Abadan Faculty of Medical Sciences, Abadan, Iran; Department of Family Medicine and Primary Care (J M Francis PhD), University of the Witwatersrand, Johannesburg, South Africa; Department of Dermatology (T Fukumoto PhD), Kobe University, Kobe, Japan; Department of Cardiovascular Medicine (M M Gad MD), Department of Internal Medicine (V Jain MD), Cleveland Clinic, Cleveland, OH, USA; Gillings School of Global Public Health (M M Gad MD), University of North Carolina Chapel Hill, Chapel Hill, NC, USA; Department of Public Health (B S Geberemariyam MPH, Y Tekalegn MPH), Madda Walabu University, Bale Robe, Ethiopia; Infectious Disease Research Center (Prof K Ghadiri MD), Pediatric Department (Prof K Ghadiri MD), Social Development and Health Promotion Research Center (F Jalilian PhD, M Mirzaei-Alavijeh PhD), Research Center for Environmental Determinants of Health (Prof B Karami Matin PhD, A Kazemi Karyani PhD, M Moradi PhD, S Soltani PhD), Clinical Research Development Center (S Maleki MSc, M Naderi PhD), Department of Health Education and Health Promotion (A Ziapour PhD), Kermanshah University of Medical Sciences, Kermanshah, Iran; Faculty of Allied Health Sciences (Prof S Gilani PhD), The University of Lahore, Lahore, Pakistan; Afro-Asian Institute, Lahore, Pakistan (Prof S Gilani PhD); Discipline of Public Health Medicine (T G Ginindza PhD), School of Nursing and Public Health (D Kuupiel PhD), University of KwaZulu-Natal, Durban, South Africa; Health Systems and Policy Research (M Golechha PhD), Indian Institute of Public Health Gandhinagar, Gandhinagar, India; Department of Exercise and Health Sciences (P N Gona PhD), University of Massachusetts Boston, Boston, MA, USA; Department of Family and Community Medicine (M I M Gubari PhD), University Of Sulaimani, Sulaimani, Iraq; Department of Microbiology (Prof H C Gugnani PhD), Department of Epidemiology (Prof H C Gugnani PhD), Saint James School of Medicine, The Valley, Anguilla; UO Neurologia, Salute Pubblica e Disabilità (Neurology, Public Health and Disability Unit) (D Guido PhD), Fondazione IRCCS Istituto Neurologico Carlo Besta (Carlo Besta Neurological Institute), Milan, Italy; College of Medicine and Health Science (R A Guled PhD), Department of Public Health (A Hashi PhD, M O Omer MSc), Jigjiga University, Jijiga, Ethiopia; NYU Shanghai, Shanghai, China (B J Hall PhD); School of Health and Environmental Studies (Prof S Hamidi DrPH), Hamdan Bin Mohammed Smart University, Dubai, United Arab Emirates; Department of Public Health (D Handiso MPH), Wachemo University, Hossana, Ethiopia; Department of Epidemiology (A Hargono Dr.), Universitas Airlangga (Airlangga University), Surabaya, Indonesia; Gastrointestinal and Liver Diseases Research Center (S Hassanipour PhD, F Joukar PhD), Caspian Digestive Disease Research Center (S Hassanipour PhD, F Joukar PhD), Guilan University of Medical Sciences, Rasht, Iran; Independent Consultant, Tabriz, Iran (H Hassankhani PhD); Institute of Pharmaceutical Sciences (K Hayat MS), University of Veterinary and Animal Sciences, Lahore, Pakistan; Department of Pharmacy

Administration and Clinical Pharmacy (K Hayat MS), Xian Jiaotong University, Xian, China; School of Business (Prof C Herteliu PhD), London South Bank University, London, UK; Department of Public Health (H D de Hidru MPH), Adigrat University, Adigrat, Ethiopia; Kasturba Medical College, Mangalore (R Holla MD, A Kamath MD, J Padubidri MD, P Rathi MD), Department of Forensic Medicine and Toxicology (Prof V C Nayak MD), Manipal Academy of Higher Education, Manipal, India; Department of Epidemiology and Population Health (H Hosgood PhD), Albert Einstein College of Medicine, Bronx, NY, USA; Department of Pharmacology (N Hossain MPhil), Bangladesh Industrial Gases Limited, Tangail, Bangladesh; College of Science and Engineering (Prof M Househ PhD), Hamad Bin Khalifa University, Doha, Qatar; Department of Occupational Safety and Health (Prof B Hwang PhD), China Medical University, Taichung, Taiwan; Department of Epidemiology (Prof M D Ilic PhD), Department of Global Health, Economics and Policy (Prof M Jakovljevic PhD), University of Kragujevac, Kragujevac, Serbia; Research Institute for Endocrine Sciences (S N Irvani MD), Department of Epidemiology (M Shahbaz MSc), Shahid Beheshti University of Medical Sciences, Tehran, Iran; School of Health Systems and Public Health (C C D Iwu MSc), Department of Medical Microbiology (M A Reta MSc), University of Pretoria, Pretoria, South Africa; Grants, Innovation and Product Development Unit (P W Mahasha PhD), South African Medical Research Council, Cape Town, South Africa (C J Iwu PhD, C A Nnaji MPH, Prof C S Wiysonge MD); Knowledge Translation Program (I O Iyamu MD), Centre for Health Evaluation and Outcome Sciences, Vancouver, BC, Canada; School of Population and Public Health (I O Iyamu MD, J A Kopec PhD), University of British Columbia, Vancouver, BC, Canada; Institute of Comparative Economic Studies (Prof M Jakovljevic PhD), Hosei University, Tokyo, Japan; Department of Community Medicine (R P Jha MSc), Dr. Baba Saheb Ambedkar Medical College & Hospital, Delhi, India; Department of Community Medicine (R P Jha MSc), Banaras Hindu University, Varanasi, India; National Institute of Epidemiology (V Joshua PhD), Indian Concil of Medical Research, Chennai, India; Department of Family Medicine and Public Health (J J Jozwiak PhD), University of Opole, Opole, Poland; Institute for Prevention of Non-communicable Diseases (R Kalhor PhD), Health Services Management Department (R Kalhor PhD), Qazvin University of Medical Sciences, Qazvin, Iran; Manipal Academy of Higher Education, India (A Kamath MD); Department of Biostatistics (N Kamyari MSc), Department of Epidemiology (S Khazaei PhD), Department of Midwifery (S Masoumi PhD), Hamadan University of Medical Sciences, Hamadan, Iran; Institute for Epidemiology and Social Medicine (A Karch MD), University of Münster, Münster, Germany; International Research Center of Excellence (G A Kayode PhD), Institute of Human Virology Nigeria, Abuja, Nigeria; Julius Centre for Health Sciences and Primary Care (G A Kayode PhD), Institute for Risk Assessment Sciences (IRAS) (E Traini MSc), Utrecht University, Utrecht, Netherlands; Open, Distance and eLearning Campus (Prof P N Keiyoro PhD), Department of Psychiatry (M Kumar PhD), School of Public Health (R G Wamai PhD), University of Nairobi, Nairobi, Kenya; School of Food and Agricultural Sciences (N Khalid PhD), University of Management and Technology, Lahore, Pakistan; Department of Population Studies (J Khan MPhil), International Institute for Population Sciences, Mumbai, India; Department of Population Science (M Khan PhD), Jatiya Kabi Kazi Nazrul Islam University, Mymensingh, Bangladesh; Faculty of Health and Wellbeing (K Khatab PhD), Sheffield Hallam University, Sheffield, UK; College of Arts and Sciences (K Khatab PhD), Ohio University, Zanesville, OH, USA; School of Traditional Chinese Medicine (Y Kim PhD), Xiamen University Malaysia, Sepang, Malaysia; School of Health Sciences (Prof A Kisa PhD), Kristiania University College, Oslo, Norway; Global Community Health and Behavioral Sciences (Prof A Kisa PhD), Tulane University, New Orleans, LA, USA; Department of Nursing and Health Promotion (S Kisa PhD), Oslo Metropolitan University, Oslo, Norway; Global Healthcare Consulting, New Delhi, India (S Kochhar MD); Arthritis Research Canada, Richmond, BC, Canada (J A Kopec PhD); Independent Consultant, Jakarta, Indonesia (S Kosen MD); Kasturba Medical College, Udupi, India (S Koulmane Laxminarayana MD); CIBERSAM (A Koyanagi MD), San Juan de Dios Sanitary Park, Sant Boi de Llobregat, Spain; Catalan Institution for Research and Advanced Studies (ICREA), Barcelona, Spain (A Koyanagi MD); Department of Anthropology (K Krishan PhD), Panjab University, Chandigarh, India; Department of Demography (Prof

B Kuate Defo PhD), Department of Social and Preventive Medicine (Prof B Kuate Defo PhD), University of Montreal, Montreal, QC, Canada; Division of Psychology and Language Sciences (M Kumar PhD), University College London, London, UK; Faculty of Health and Life Sciences (O P Kurmi PhD), Coventry University, Coventry, UK; Department of Medicine (O P Kurmi PhD), Department of Psychiatry and Behavioural Neurosciences (A T Olagunju MD), McMaster University, Hamilton, ON, Canada; Faculty of Public Health (D Kusuma DSc), University of Indonesia, Depok, Indonesia; Department of Nursing (D Kuupiel PhD), Research for Sustainable Development Consult, Sunyani, Ghana; Public Health Foundation of India, Gurugram, India (D K Lal MD); Division of Research (J O Lam PhD), Kaiser Permanente, Oakland, CA, USA; Unit of Genetics and Public Health (Prof I Landires MD), Unit of Microbiology and Public Health (V Nuñez-Samudio PhD), Institute of Medical Sciences, Las Tablas, Panama; Department of Public Health (V Nuñez-Samudio PhD), Ministry of Health, Herrera, Panama (Prof I Landires MD); Department of Otorhinolaryngology (S Lasrado MS), Father Muller Medical College, Mangalore, India; Barcelona Institute for Global Health, Barcelona, Spain (Prof J V Lazarus PhD); School of Public Health (C T Leshargie MPH), Haramaya University, Debre Markos, Ethiopia; Department of Sociology (B Li PhD), Shenzhen University, Shenzhen, China; Department of Systems, Populations, and Leadership (X Liu PhD), University of Michigan, Ann Arbor, MI, USA; Department of Pharmaceutical Science (H I M Amin PhD), University of Eastern Piedmont, Novara, Italy; Chemistry Department (H I M Amin PhD), International University of Erbil, Erbil, Iraq; Environmental Health Research Center (Prof A Maleki PhD), Social Determinants of Health Research Center (Y Moradi PhD), Kurdistan University of Medical Sciences, Sanandaj, Iran; Institute for Social Science Research (A A Mamun PhD), The University of Queensland, Indooroopilly, QLD, Australia; School of Medicine (N Manafi MD), University of Manitoba, Winnipeg, MB, Canada; Campus Caucaia (F R Martins-Melo PhD), Federal Institute of Education, Science and Technology of Ceará, Caucaia, Brazil; ICF International (B K Mayala PhD), DHS Program, Rockville, MD, USA; Department of Pharmacy (B Meharie MSc), Wollo University, Dessie, Ethiopia; Peru Country Office (W Mendoza MD), United Nations Population Fund (UNFPA), Lima, Peru; Breast Surgery Unit (T J Meretoja MD), Helsinki University Hospital, Helsinki, Finland; University of Helsinki, Helsinki, Finland (T J Meretoja MD); Clinical Microbiology and Parasitology Unit (T Mestrovic PhD), Dr. Zora Profozic Polyclinic, Zagreb, Croatia; University Centre Varazdin (T Mestrovic PhD), University North, Varazdin, Croatia; Pacific Institute for Research & Evaluation, Calverton, MD, USA (T R Miller PhD); Department of Radiation Oncology (O Mohamad MD), University of California San Francisco, San Francisco, CA, USA; Internal Medicine Department (Y Mohammad MD), King Saud University, Riyadh, Saudi Arabia; Department of Epidemiology and Biostatistics (A Mohammadian-Hafshejani PhD), Shahrekord University of Medical Sciences, Shahrekord, Iran; Department of Public Health (J A Mohammed MPH), Samara University, Semera, Ethiopia; Department of Biomolecular Sciences (S Mohammed MSc), University of Mississippi, Oxford, MS, USA; Department of Pharmacy (S Mohammed MSc), Mizan-Tepi University, Mizan, Ethiopia; Health Systems and Policy Research Unit (S Mohammed PhD), Department of Community Medicine (M B Sufiyan MD), Department of Surgery (M A Tolani FWACS, M A Tolani FWACS), Ahmadu Bello University, Zaria, Nigeria; Department of Midwifery (T M Mokonnon MPH), Wolaita Sodo University, Wolaita Sodo, Ethiopia; Wolaita Sodo University, Wolkite, Ethiopia (T M Mokonnon MPH); Computer, Electrical, and Mathematical Sciences and Engineering Division (P Moraga PhD), King Abdullah University of Science and Technology, Thuwal, Saudi Arabia; Department of Pediatric Medicine (Prof G Mustafa MD), The Children's Hospital & The Institute of Child Health, Multan, Pakistan; Department of Pediatrics & Pediatric Pulmonology (Prof G Mustafa MD), Institute of Mother & Child Care, Multan, Pakistan; Department of Pharmacology, Saveetha Dental College (S Muthupandian PhD), Saveetha Institute of Medical and Technical Sciences (SIMATS), Chennai, India; Research and Analytics Department (A J Nagarajan M.Tech), Initiative for Financing Health and Human Development, Chennai, India; Department of Research and Analytics (A J Nagarajan M.Tech), Bioinsilico Technologies, Chennai, India; Department of Biotechnology (M Naveed PhD), University of Central Punjab, Lahore, Pakistan; Disease

Control and Environmental Health (R Ndejjo MSc), Makerere University, Kampala, Uganda; Department of Community Medicine (S Nepal MD), Kathmandu University, Palpa, Nepal; College of Medicine and Health Sciences, (H B Netsere MS), Bahir Dar University, Gondar, Ethiopia; Ministry of Health, Community Development, Gender, Elderly and Children, Dar es Salaam, Tanzania (F N Ngalesoni PhD); Department of Public Health (G Nguefack-Tsague PhD), University of Yaoundé I, Yaoundé, Cameroon; Department of Biological Sciences (J W Ngunjiri DrPH), University of Embu, Embu, Kenya; Institute for Mental Health and Policy (Y T Nigatu PhD), Centre for Addiction and Mental Health, Toronto, ON, Canada; Department of Clinical Epidemiology (Y T Nigatu PhD), Institute for Clinical Evaluative Sciences, Ottowa, ON, Canada; Centre for Heart Rhythm Disorders (J Noubiap MD), University of Adelaide, Adelaide, SA, Australia; Administrative and Economic Sciences Department (Prof B Oancea PhD), University of Bucharest, Bucharest, Romania; Department of Community Health and Primary Care (O O Odukoya MSc), University of Lagos, Idi Araba, Nigeria; Department of Family and Preventive Medicine (O O Odukoya MSc), University of Utah, Salt Lake City, Utah, USA; Translational Health Research Institute (F A Ogbo PhD), Western Sydney University, Sydney, NSW, Australia; Department of Public Health (O Oladimeji PhD), Walter Sisulu University, Mthatha, South Africa; School of Public Health (O Oladimeji PhD), Center for Community Healthcare, Research, and Development, Abuja, Nigeria; Department of Psychiatry (A T Olagunju MD), University of Lagos, Lagos, Nigeria; Centre for Healthy Start Initiative, Lagos, Nigeria (B O Olusanya PhD, J O Olusanya MBA); Department of Anatomic Pathology (A E E Omonisi FWACP), Ekiti State University, Ado- Ekiti, Nigeria; Department of Anatomic Pathology (A E E Omonisi FWACP), Ekiti State University Teaching Hospital, Ado- Ekiti, Nigeria; Department of Pharmacology and Therapeutics (Prof O E Onwujekwe PhD), University of Nigeria Nsukka, Enugu, Nigeria; University of Port Harcourt, Port Harcourt, Nigeria (Prof O E Orisakwe PhD); Laboratory of Public Health Indicators Analysis and Health Digitalization (N Otstavnov BA), Moscow Institute of Physics and Technology, Dolgoprudny, Russia; Department of Respiratory Medicine (Prof M P A DNB), Jagadguru Sri Shivarathreeswara Academy of Health Education and Research, Mysore, India; Department of Medicine (S Pakhale MD), Ottawa Hospital Research Institute, Ottawa, ON, Canada; Department of Health Metrics (A Pana MD), Center for Health Outcomes & Evaluation, Bucharest, Romania; Corporate (S R Pandi-Perumal MSc), Somnogen Canada Inc, Toronto, ON, Canada; Department of Neurology and Public Health (U K Patel MD), Icahn School of Medicine at Mount Sinai, New York, NY, USA; Research & Development Department (M Pathak PhD), Kalinga Institute of Medical Sciences, Bhubaneswar, India; Department of Pediatrics (Prof G C Patton MD), Department of Neurology (Prof T Wijeratne MD), University of Melbourne, Melbourne, VIC, Australia; Population Health Theme (Prof G C Patton MD), Murdoch Children's Research Institute, Melbourne, VIC, Australia; Department of Genetics (S Pawar PhD), Yale University, New Haven, CT, USA; School of Global Public Health (E K Peprah PhD), New York University, New York, NY, USA; HIV and Mental Health Department (K N Pokhrel PhD), Integrated Development Foundation Nepal, Kathmandu, Nepal; University Medical Center Groningen (Prof M J Postma PhD), School of Economics and Business (Prof M J Postma PhD), University of Groningen, Groningen, Netherlands; Department of Nutrition and Food Sciences (H Pourjafar PhD), Maragheh University of Medical Sciences, Maragheh, Iran; Dietary Supplements and Probiotic Research Center (H Pourjafar PhD), Alborz University of Medical Sciences, Karaj, Iran; Health Sciences Department (D R A Pribadi MSc), Muhammadiyah University of Surakarta, Sukoharjo, Indonesia; Department of Community Medicine (Prof Z Quazi Syed PhD), Datta Meghe Institute of Medical Sciences, Wardha, India; Thalassemia and Hemoglobinopathy Research Center (F Rahim PhD), Ahvaz Jundishapur University of Medical Sciences, Ahvaz, Iran; Department of Community Medicine (M Rahman PhD), Maharishi Markandeshwar Medical College & Hospital, Solan, India; Future Technology Research Center (A Rahmani PhD), National Yunlin University of Science and Technology, Yunlin, Taiwan; Institute of Research and Development (A Rahmani PhD), Duy Tan University, Da Nang, Vietnam; Department of Public Health (J Rana MPH), North South University, Dhaka, Bangladesh; Department of Biostatistics and Epidemiology (J Rana MPH),

University of Massachusetts Amherst, Amherst, MA, USA; Research Department (C L Ranabhat PhD), Policy Research Institute, Kathmandu, Nepal; Health and Public Policy Department (C L Ranabhat PhD), Global Center for Research and Development, Kathmandu, Nepal; Department of Oral Pathology (S Rao MDS), Srinivas Institute of Dental Sciences, Mangalore, India; University College London Hospitals, London, UK (D L Rawaf MD); Academic Public Health England (Prof S Rawaf MD), Public Health England, London, UK; Department of Computer Science (R Rawassizadeh PhD), Boston University, Boston, MA, USA; School of Nursing and Midwifery (V Renjith PhD), Royal College of Surgeons in Ireland - Bahrain, Muharraq Governorate, Bahrain; Department of Medical Laboratory Science (M A Reta MSc), Woldia University, Woldia, Ethiopia; Network of Immunity in Infection, Malignancy and Autoimmunity (NIIMA) (Prof N Rezaei PhD), Universal Scientific Education and Research Network (USERN), Tehran, Iran; Department of Health Statistics (S F Rumisha PhD), National Institute for Medical Research, Dar es Salaam, Tanzania; Department of Psychiatry (Prof R Sagar MD), All India Institute of Medical Sciences, New Delhi, India; Department of Phytochemistry (Prof S Sajadi PhD), Soran University, Soran, Iraq; Department of Nutrition (Prof S Sajadi PhD), Cihan University-Erbil, Erbil, Iraq; Public Health and Community Medicine Department (M R Salem MD), Cairo University, Giza, Egypt; Department of Entomology (A M Samy PhD), Ain Shams University, Cairo, Egypt; Department of Geriatrics and Long Term Care (B Sathian PhD), Hamad Medical Corporation, Doha, Qatar; Faculty of Health & Social Sciences (B Sathian PhD), Bournemouth University, Bournemouth, UK; School of Public Health and Community Medicine (Prof A E Schutte PhD), University of New South Wales, Sydney, NSW, Australia; The George Institute for Global Health, Sydney, NSW, Australia (Prof A E Schutte PhD); College of Public Health, Medical and Veterinary Sciences (A Seidu MPhil), James Cook University, Townsville, QLD, Australia; Center for Biomedical Information Technology (F Sha PhD), Shenzhen Institutes of Advanced Technology, Shenzhen, China; Department of Radiology and Radiological Science (O Shafaat MD), Johns Hopkins University, Baltimore, MD, USA; Independent Consultant, Karachi, Pakistan (M A Shaikh MD); School of Public Health (M F Shaka MPH), Dilla University, Dilla, Ethiopia; Centre for Medical Informatics (Prof A Sheikh MD), University of Edinburgh, Edinburgh, UK; College of Medicine (Prof J Shin MD), Yonsei University, Seoul, South Korea; Public Health Dentistry Department (Prof K M Shivakumar PhD), Krishna Institute of Medical Sciences Deemed to be University, Karad, India; School of Medicine (Prof J A Singh MD), University of Alabama at Birmingham, Birmingham, AL, USA; Medicine Service (Prof J A Singh MD), US Department of Veterans Affairs (VA), Birmingham, AL, USA; Department No.16 (V Y Skryabin MD), Laboratory of Genetics and Genomics (Prof M S Zastrozhin PhD), Moscow Research and Practical Centre on Addictions, Moscow, Russia; Therapeutic Department (A A Skryabina MD), Balashiha Central Hospital, Balashikha, Russia; Nursing Care Research Center (A Soheili PhD), Semnan University of Medical Sciences, Semnan, Iran; Demography and Population Studies Department (O D Somefun PhD), University of Witwatersrand, Johannesburg, South Africa; Department of Pharmacy (B W Taddele MPH), Arbaminch College of Health Sciences, Arba Minch, Ethiopia; University of KwaZulu-Natal, South Africa (Prof F C Tanser PhD); Africa Health Research Institute, Berea, South Africa (Prof F C Tanser PhD); University Institute "Egas Moniz", Monte da Caparica, Portugal (Prof N Taveira PhD); Research Institute for Medicines (Prof N Taveira PhD), Psychiatry and Medical Psychology Department (M Torrado PhD), University of Lisbon, Lisbon, Portugal; Division of Biostatistics and Epidemiology (B Thakur PhD), Texas Tech University Health Sciences Center, El Paso, TX, USA; Institute of Public Health (R Topor-Madry PhD), Jagiellonian University Medical College, Kraków, Poland; Agency for Health Technology Assessment and Tariff System, Warsaw, Poland (R Topor-Madry PhD); Child and Adolescent Mental Health Services (CAMHS) (M Torrado PhD), Hospital Garcia de Orta, Almada, Portugal; Department of Pathology and Legal Medicine (M R Tovani-Palone PhD), University of São Paulo, Ribeirão Preto, Brazil; Modestum LTD, London, UK (M R Tovani-Palone PhD); Department of Psychiatry (A C Tsai MD), Massachusetts General Hospital, Boston, MA, USA; Mbarara University of Science and Technology, Mbarara, Uganda (A C Tsai MD); Department of Allied Health Sciences (I Ullah PhD), Iqra National University, Peshawar, Pakistan;

Institute of Soil and Environmental Sciences (S Ullah PhD), University of Agriculture, Faisalabad, Faisalabad, Pakistan; Department of Community Medicine (C D Umeokonkwo MPH), Alex Ekwueme Federal University Teaching Hospital Abakaliki, Abakaliki, Nigeria; Laboratory of Toxicology (C Vardavas PhD), University of Crete, Heraklion, Crete, Greece; Department of Medical and Surgical Sciences (Prof F S Violante MD), University of Bologna, Bologna, Italy; Occupational Health Unit (Prof F S Violante MD), Sant'Orsola Malpighi Hospital, Bologna, Italy; Faculty of Information Technology (B Vo PhD), Ho Chi Minh City University of Technology (HUTECH), Ho Chi Minh City, Vietnam; Population Dynamics and Sexual and Reproductive Health (Y D Wado PhD), African Population and Health Research Center, Nairobi, Kenya; Foundation University Medical College (Prof Y Waheed PhD), Foundation University Islamabad, Islamabad, Pakistan; Cultures, Societies and Global Studies, & Integrated Initiative for Global Health (R G Wamai PhD), Northeastern University, Boston, MA, USA; Demographic Change and Aging Research Area (A Werdecker PhD), Federal Institute for Population Research, Wiesbaden, Germany; Department of Community Medicine (N D Wickramasinghe MD), Rajarata University of Sri Lanka, Anuradhapura, Sri Lanka; Department of Medicine (Prof T Wijeratne MD), University of Rajarata, Saliyapura Anuradhapuraya, Sri Lanka; Department of Public Health (T G Wondmeneh MPH), Samara University, Samara, Ethiopia; Department of Diabetes and Metabolic Diseases (T Yamada MD), University of Tokyo, Tokyo, Japan; School of International Development and Global Studies (Prof S Yaya PhD), University of Ottawa, Ottawa, ON, Canada; Centre for Suicide Research and Prevention (Prof P Yip PhD), Department of Social Work and Social Administration (Prof P Yip PhD), University of Hong Kong, Hong Kong, China; Department of Neuropsychopharmacology (N Yonemoto MPH), National Center of Neurology and Psychiatry, Kodaira, Japan; Department of Public Health (N Yonemoto MPH), Juntendo University, Tokyo, Japan; Department of Health Care Management and Economics (H Yusefzadeh PhD), Urmia University of Medical Science, Urmia, Iran; Department of Pharmaceutics (S Zaidi PhD), Dow University of Health Sciences, Karachi, Pakistan; Department of Parasitology and Entomology (L Zaki PhD), Tarbiat Modares University, Tehran, Iran; Addictology Department (Prof M S Zastrozhin PhD), Pediatrics Department (A Zastrozhina PhD), Russian Medical Academy of Continuous Professional Education, Moscow, Russia; School of Public Health (Y Zhang PhD), Hubei Province Key Laboratory of Occupational Hazard Identification and Control (Y Zhang PhD), Wuhan University of Science and Technology, Wuhan, China; School of Medicine (Z Zhang PhD), Wuhan University, Wuhan, China.

2 Author Contributions

Managing the estimation or publications process
Benn Sartorius, Samantha Perkins, Kate E LeGrand, Natalie Marie Cormier, Simon I Hay, and Laura Dwyer-Lindgren.

Writing the first draft of the manuscript
Benn Sartorius, John D VanderHeide, Lauren E Schaeffer, and Laura Dwyer-Lindgren.

Primary responsibility for seeking, cataloguing, extracting, or cleaning data; designing or coding figures and tables
Mingyou Yang, Julia Hon, Erik A Goosmann, and Audrey L Serfes.

Providing data or critical feedback on data sources
John D VanderHeide, Erik A Goosmann, Julia Hon, Emily Haeuser, Deepa Jahagirdar, Audrey L Serfes, Zeleke Hailemariam Abebo, Akine Eshete Abosetugn, Manfred Mario Kokou Accrombessi, Oladimeji M Adebayo, Victor Adekanmbi, Olatunji O Adetokunboh, Bright Opoku Ahinkorah, Muktar Beshir Ahmed, Oluwaseun Oladapo Akinyemi, Addis Aklilu, Chisom Joyqueenet Akunna, Fares Alahdab, Noore Alam, Turki M Alanzi, Biresaw Wassihun Alemu, Tilahun Ali, Saeed Amini, Robert Ancuceanu, Fereshteh Ansari, Davood Anvari, Razique Anwer, Seth Christopher Yaw Appiah, Jalal Arabloo, Mulusew A Asemahagn, Wondwossen Niguse Asmare, Alok Atreya, Marcel Ausloos, Beatriz Paulina Ayala Quintanilla, Yared Asmare Aynalem, Muluken Altaye Ayza, Samad Azari, Senthilkumar Balakrishnan, Maciej Banach, Till Winfried Bärnighausen, Sanjay Basu, Neeraj Bedi, Akshaya Srikanth Bhagavathula, Boris Bikbov, Archith Boloor, Nicola Luigi Bragazzi, Sharath Burugina Nagaraja, Souranshu Chatterjee, Soosanna Kumary Chattu, Vijay Kumar Chattu, Dinh-Toi Chu, Carlos Culquichicon, Giovanni Damiani, Parnaz Daneshpajouhnejad, Ahmad Daryani, Meseret Derbew Molla, Govinda Prasad Dhungana, Lucas Earl, Jeffrey W Eaton, Andem Effiong, Maysaa El Sayed Zaki, Rajesh Elayedath, Abidemi Omolara Fasanmi, Morenike Oluwatoyin Folayan, Joel Msafiri Francis, Takeshi Fukumoto, Mohamed M Gad, Birhan Gebresillassie Gebregiorgis, Keyghobad Ghadiri, Ahmad Ghashghaee, Syed Amir Gilani, Themba G Ginindza, Mahaveer Golechha, Harish Chander Gugnani, Brian J Hall, Arief Hargono, Soheil Hassanipour, Hadi Hassankhani, Claudiu Herteliu, Mehdi Hosseinzadeh, Mowafa Househ, Segun Emmanuel Ibitoye, Seyed Sina Naghibi Irvani, Chidozie C D Iwu, Ihoghosa Osamuyi Iyamu, Vardhmaan Jain, Mihajlo Jakovljevic, Farzad Jalilian, Kimberly B Johnson, Farahnaz Joukar, Jacek Jerzy Jozwiak, Rohollah Kalhor, Naser Kamyari, Tanuj Kanchan, André Karch, Salah Eddin Karimi, Gbenga A Kayode, Nauman Khalid, Md Nuruzzaman Khan, Khaled Khatab, Yun Jin Kim, Adnan Kisa, Sezer Kisa, Soewarta Kosen, Sindhura Lakshmi Koulmane Laxminarayana, Kewal Krishan, Barthelemy Kuate Defo, Manasi Kumar, Dian Kusuma, Dharmesh Kumar Lal, Alice Lazzar-Atwood, Bingyu Li, Xuefeng Liu, Hawraz Ibrahim M. Amin, Navid Manafi, Francisco Rogerlândio Martins-Melo, Benjamin K Mayala, Walter Mendoza, Endalkachew Worku Mengesha, Abdollah Mohammadian-Hafshejani, Jemal Abdu Mohammed, Salahuddin Mohammed, Shafiu Mohammed, Ali H Mokdad, Taklu Marama Mokonnon, Masoud Moradi, Ghulam Mustafa, Saravanan Muthupandian, Ahamarshan Jayaraman Nagarajan, Mohsen Naghavi, Javad Nazari, Samata Nepal, Henok Biresaw Netsere, Frida N Ngalesoni, Josephine W Ngunjiri, Chukwudi A Nnaji, Jean Jacques Noubiap, Bogdan Oancea, Oluwakemi Ololade Odukoya, Felix Akpojene Ogbo, Olanrewaju Oladimeji, Andrew T Olagunju, Bolajoko Olubukunola Olusanya, Jacob Olusegun Olusanya, Abidemi E Emmanuel Omonisi, Obinna E Onwujekwe, Mayowa O Owolabi, Mahesh P A, Jagadish Rao Padubidri, Adrian Pana, Shrikant Pawar, Khem Narayan Pokhrel, Maarten J Postma, Hadi Pourjafar, Dimas Ria Angga Pribadi, Zahiruddin Quazi Syed, Alireza Rafiei, Fakher Rahim, Mohammad Hifz Ur Rahman, Amir Masoud

Rahmani, Chhabi Lal Ranabhat, Sowmya J Rao, David Laith Rawaf, Salman Rawaf, Reza Rawassizadeh, Nima Rezaei, Rajesh Sagar, S. Mohammad Sajadi, Marwa Rashad Salem, Abdallah M Samy, Brijesh Sathian, Abdul-Aziz Seidu, Feng Sha, Masood Ali Shaikh, Mohammed Feyisso Shaka, Jae Il Shin, Negussie Boti Sidemo, Jasvinder A Singh, Valentin Yurievich Skryabin, Anna Aleksandrovna Skryabina, Amin Soheili, Emma Elizabeth Spurlock, Animut Tagele Tamiru, Frank C Tanser, Nuno Taveira, Musliu Adetola Tolani, Marco Torrado, Marcos Roberto Tovani-Palone, Alexander C Tsai, Irfan Ullah, Chukwuma David Umeokonkwo, Bhaskaran Unnikrishnan, Bay Vo, Yasir Waheed, Richard G Wamai, Tissa Wijeratne, Charles Shey Wiysonge, Temesgen Gebeyehu Wondmeneh, Sanni Yaya, Yordanos Gizachew Yeshitila, Naohiro Yonemoto, Tewodros Yosef, Syed Saoud Zaidi, Dejene Tesfaye Zewdie, Zhi-Jiang Zhang, Arash Ziapour, Simon I Hay, and Laura Dwyer-Lindgren.

Developing methods or computational machinery
John D VanderHeide, Mingyou Yang, Emily Haeuser, Deepa Jahagirdar, Kate E LeGrand, Akine Eshete Abosetugn, Muktar Beshir Ahmed, Davood Anvari, Yared Asmare Aynalem, Farah Daoud, Ahmad Daryani, Lucas Earl, Alebachew Fasil, Mehdi Hosseinzadeh, Mowafa Househ, Salah Eddin Karimi, Adnan Kisa, Sezer Kisa, Jemal Abdu Mohammed, Salahuddin Mohammed, Ali H Mokdad, Mohsen Naghavi, Josephine W Ngunjiri, Samuel Negash Nigussie, Abidemi E Emmanuel Omonisi, Urvish K Patel, Amir Masoud Rahmani, Chhabi Lal Ranabhat, Reza Rawassizadeh, Abdallah M Samy, Negussie Boti Sidemo, Emma Elizabeth Spurlock, Animut Tagele Tamiru, Bay Vo, Temesgen Gebeyehu Wondmeneh, Yordanos Gizachew Yeshitila, Syed Saoud Zaidi, Arash Ziapour, Simon I Hay, and Laura Dwyer-Lindgren.

Primary responsibility for applying analytical methods to produce estimates
John D VanderHeide, Emily Hauser, Michael A Cork, and Deepa Jahagirdar.

Providing critical feedback on methods or results
John D VanderHeide, Mingyou Yang, Michael A Cork, Lauren E Schaeffer, Kate E LeGrand, Hedayat Abbastabar, Zeleke Hailemariam Abebo, Akine Eshete Abosetugn, Eman Abu-Gharbieh, Oladimeji M Adebayo, Adeyinka Emmanuel Adegbosin, Victor Adekanmbi, Olatunji O Adetokunboh, Daniel Adedayo Adeyinka, Bright Opoku Ahinkorah, Keivan Ahmadi, Muktar Beshir Ahmed, Oluwaseun Oladapo Akinyemi, Rufus Olusola Akinyemi, Addis Aklilu, Chisom Joyqueenet Akunna, Fares Alahdab, Ziyad Al-Aly, Noore Alam, Alehegn Aderaw Alamneh, Turki M Alanzi, Biresaw Wassihun Alemu, Robert Kaba Alhassan, Tilahun Ali, Vahid Alipour, Saeed Amini, Robert Ancuceanu, Fereshteh Ansari, Zelalem Alamrew Anteneh, Davood Anvari, Razique Anwer, Seth Christopher Yaw Appiah, Jalal Arabloo, Mulusew A Asemahagn, Mohammad Asghari Jafarabadi, Wondwossen Niguse Asmare, Desta Debalkie Atnafu, Maha Moh'd Wahbi Atout, Alok Atreya, Marcel Ausloos, Atalel Fentahun Awedew, Beatriz Paulina Ayala Quintanilla, Martin Amogre Ayanore, Yared Asmare Aynalem, Muluken Altaye Ayza, Samad Azari, Zelalem Nigussie Azene, Zaheer-Ud-Din Babar, Atif Amin Baig, Senthilkumar Balakrishnan, Maciej Banach, Till Winfried Bärnighausen, Sanjay Basu, Mohsen Bayati, Tariku Tesfaye Bekuma, Woldesellassie M Mequanint Bezabhe, Pankaj Bhardwaj, Krittika Bhattacharyya, Sadia Bibi, Boris Bikbov, Tsegaye Adane Birhan, Zebenay Workneh Bitew, Moses John Bockarie, Archith Boloor, Oliver J Brady, Nicola Luigi Bragazzi, Sharath Burugina Nagaraja, Zahid A Butt, Rosario Cárdenas, Jaykaran Charan, Souranshu Chatterjee, Soosanna Kumary Chattu, Vijay Kumar Chattu, Mohiuddin Ahsanul Kabir Chowdhury, Dinh-Toi Chu, Richard G Cowden, Carlos Culquichicon, Baye Dagnew, Saad M A Dahlawi, Giovanni Damiani, Ahmad Daryani, José das Neves, Meseret Derbew Molla, Kebede Deribe, Abebaw Alemayehu Desta, Keshab Deuba, Govinda Prasad Dhungana, Daniel Diaz, Shirin Djalalinia, Paul Narh Doku, Bereket Duko, Arielle Wilder Eagan, Jeffrey W Eaton, Andem Effiong, Maysaa El Sayed Zaki, Rajesh Elayedath, Aisha Elsharkawy, Sharareh Eskandarieh, Sayeh Ezzikouri, Abidemi Omolara Fasanmi, Alebachew Fasil, Valery L Feigin, Florian Fischer, Nataliya A Foigt, Morenike Oluwatoyin Folayan, Masoud Foroutan, Joel Msafiri

Francis, Takeshi Fukumoto, Mohamed M Gad, Biniyam Sahiledengle Geberemariyam, Birhan Gebresillassie Gebregiorgis, Berhe Gebremichael, Hailay Abrha Gesesew, Lemma Getacher, Ahmad Ghashghaee, Themba G Ginindza, Mustefa Glagn, Mahaveer Golechha, Philimon N Gona, Harish Chander Gugnani, Davide Guido, Rashid Abdi Guled, Brian J Hall, Samer Hamidi, Demelash Woldeyohannes Handiso, Abdiwahab Hashi, Soheil Hassanipour, Hadi Hassankhani, Khezar Hayat, Claudiu Herteliu, Hagos Degefa de Hidru, Ramesh Holla, H Dean Hosgood, Mehdi Hosseinzadeh, Mowafa Househ, Bing-Fang Hwang, Segun Emmanuel Ibitoye, Olayinka Stephen Ilesanmi, Irena M Ilic, Milena D Ilic, Seyed Sina Naghibi Irvani, Chidozie C D Iwu, Chinwe Juliana Iwu, Vardhmaan Jain, Mihajlo Jakovljevic, Ravi Prakash Jha, Vasna Joshua, Farahnaz Joukar, Jacek Jerzy Jozwiak, Ali Kabir, Leila R Kalankesh, Rohollah Kalhor, Ashwin Kamath, Naser Kamyari, Tanuj Kanchan, Behzad Karami Matin, André Karch, Salah Eddin Karimi, Ayele Semachew Kasa, Getinet Kassahun, Gbenga A Kayode, Ali Kazemi Karyani, Peter Njenga Keiyoro, Bayew Kelkay, Nauman Khalid, Junaid Khan, Md Nuruzzaman Khan, Khaled Khatab, Salman Khazaei, Yun Jin Kim, Adnan Kisa, Sezer Kisa, Sonali Kochhar, Jacek A Kopec, Sindhura Lakshmi Koulmane Laxminarayana, Ai Koyanagi, Kewal Krishan, Barthelemy Kuate Defo, Nuworza Kugbey, Vaman Kulkarni, Manasi Kumar, Nithin Kumar, Om P Kurmi, Dian Kusuma, Desmond Kuupiel, Hmwe Hmwe Kyu, Carlo La Vecchia, Dharmesh Kumar Lal, Jennifer O Lam, Cheru Tesema Leshargie, Xuefeng Liu, Phetole Walter Mahasha, Azeem Majeed, Shokofeh Maleki, Abdullah A Mamun, Navid Manafi, Mohammad Ali Mansournia, Francisco Rogerlândio Martins-Melo, Seyedeh Zahra Masoumi, Benjamin K Mayala, Hagazi Gebre Meles, Mulugeta Melku, Walter Mendoza, Endalkachew Worku Mengesha, Tuomo J Meretoja, Abera M Mersha, Ted R Miller, Andreea Mirica, Mehdi Mirzaei-Alavijeh, Osama Mohamad, Yousef Mohammad, Abdollah Mohammadian-Hafshejani, Jemal Abdu Mohammed, Salahuddin Mohammed, Shafiu Mohammed, Ali H Mokdad, Taklu Marama Mokonnon, Mariam Molokhia, Yousef Moradi, Rahmatollah Moradzadeh, Jonathan F Mosser, Saravanan Muthupandian, Mehdi Naderi, Ahamarshan Jayaraman Nagarajan, Mohsen Naghavi, Muhammad Naveed, Javad Nazari, Rawlance Ndejjo, Samata Nepal, Henok Biresaw Netsere, Frida N Ngalesoni, Georges Nguefack-Tsague, Josephine W Ngunjiri, Yeshambel T Nigatu, Samuel Negash Nigussie, Chukwudi A Nnaji, Jean Jacques Noubiap, Bogdan Oancea, Oluwakemi Ololade Odukoya, Felix Akpojene Ogbo, Olanrewaju Oladimeji, Andrew T Olagunju, Bolajoko Olubukunola Olusanya, Jacob Olusegun Olusanya, Muktar Omer Omer, Abidemi E Emmanuel Omonisi, Obinna E Onwujekwe, Nikita Otstavnov, Mayowa O Owolabi, Mahesh P A, Jagadish Rao Padubidri, Smita Pakhale, Adrian Pana, Seithikurippu R Pandi-Perumal, Urvish K Patel, Mona Pathak, Shrikant Pawar, Emmanuel K Peprah, Khem Narayan Pokhrel, Maarten J Postma, Faheem Hyder Pottoo, Hadi Pourjafar, Zahiruddin Quazi Syed, Alireza Rafiei, Fakher Rahim, Mohammad Hifz Ur Rahman, Amir Masoud Rahmani, Pradhum Ram, Juwel Rana, Chhabi Lal Ranabhat, Satish Rao, Sowmya J Rao, Priya Rathi, David Laith Rawaf, Salman Rawaf, Reza Rawassizadeh, Vishnu Renjith, Melese Abate Reta, Nima Rezaei, Aziz Rezapour, Ana Isabel Ribeiro, Jennifer M Ross, Susan Fred Rumisha, Rajesh Sagar, Maitreyi Sahu, S. Mohammad Sajadi, Marwa Rashad Salem, Abdallah M Samy, Brijesh Sathian, Aletta Elisabeth Schutte, Abdul-Aziz Seidu, Omid Shafaat, Mohammad Shahbaz, Masood Ali Shaikh, Mohammed Feyisso Shaka, Aziz Sheikh, Jae Il Shin, Negussie Boti Sidemo, Jasvinder A Singh, Valentin Yurievich Skryabin, Anna Aleksandrovna Skryabina, Amin Soheili, Shahin Soltani, Oluwaseyi Dolapo Somefun, Muluken Bekele Sorrie, Mu'awiyyah Babale Sufiyan, Biruk Wogayehu Taddele, Eyayou Girma Tadesse, Zemenu Tamir, Animut Tagele Tamiru, Frank C Tanser, Nuno Taveira, Arash Tehrani-Banihashemi, Yohannes Tekalegn, Fisaha Haile Tesfay, Belay Tessema, Zemenu Tadesse Tessema, Bhaskar Thakur, Musliu Adetola Tolani, Marco Torrado, Marcos Roberto Tovani-Palone, Eugenio Traini, Alexander C Tsai, Gebiyaw Wudie Tsegaye, Irfan Ullah, Saif Ullah, Chukwuma David Umeokonkwo, Bhaskaran Unnikrishnan, Constantine Vardavas, Francesco S Violante, Bay Vo, Yohannes Dibaba Wado, Yasir Waheed, Richard G Wamai, Yanzhong Wang, Paul Ward, Andrea Werdecker, Nuwan Darshana Wickramasinghe, Tissa Wijeratne, Charles Shey Wiysonge, Temesgen Gebeyehu Wondmeneh, Tomohide Yamada, Sanni Yaya, Yordanos Gizachew Yeshitila, Mekdes Tigistu

Yilma, Paul Yip, Naohiro Yonemoto, Tewodros Yosef, Hasan Yusefzadeh, Syed Saoud Zaidi, Leila Zaki, Maryam Zamanian, Mikhail Sergeevich Zastrozhin, Anasthasia Zastrozhina, Dejene Tesfaye Zewdie, Yunquan Zhang, Zhi-Jiang Zhang, Arash Ziapour, Simon I Hay, and Laura Dwyer-Lindgren.

Drafting the work or revising it critically for important intellectual content
John D VanderHeide, Mingyou Yang, Lauren E Schaeffer, Kate E LeGrand, Hedayat Abbastabar, Eman Abu-Gharbieh, Oladimeji M Adebayo, Victor Adekanmbi, Olatunji O Adetokunboh, Daniel Adedayo Adeyinka, Bright Opoku Ahinkorah, Muktar Beshir Ahmed, Yonas Akalu, Oluwaseun Oladapo Akinyemi, Rufus Olusola Akinyemi, Fares Alahdab, Alehegn Aderaw Alamneh, Robert Kaba Alhassan, Saeed Amini, Robert Ancuceanu, Seth Christopher Yaw Appiah, Jalal Arabloo, Mulusew A Asemahagn, Mohammad Asghari Jafarabadi, Maha Moh'd Wahbi Atout, Alok Atreya, Marcel Ausloos, Martin Amogre Ayanore, Zaheer-Ud-Din Babar, Atif Amin Baig, Senthilkumar Balakrishnan, Maciej Banach, Till Winfried Bärnighausen, Sanjay Basu, Mohsen Bayati, Neeraj Bedi, Tariku Tesfaye Bekuma, Woldesellassie M Mequanint Bezabhe, Akshaya Srikanth Bhagavathula, Krittika Bhattacharyya, Boris Bikbov, Moses John Bockarie, Oliver J Brady, Nicola Luigi Bragazzi, Andrey Nikolaevich Briko, Nikolay Ivanovich Briko, Sharath Burugina Nagaraja, Felix Carvalho, Souranshu Chatterjee, Soosanna Kumary Chattu, Vijay Kumar Chattu, Dinh-Toi Chu, Richard G Cowden, Carlos Culquichicon, Baye Dagnew, Giovanni Damiani, Ahmad Daryani, José das Neves, Nicole Davis Weaver, Meseret Derbew Molla, Kebede Deribe, Abebaw Alemayehu Desta, Samath Dhamminda Dharmaratne, Govinda Prasad Dhungana, Daniel Diaz, Eleonora Dubljanin, Jeffrey W Eaton, Andem Effiong, Maysaa El Sayed Zaki, Maha El Tantawi, Shaimaa I El-Jaafary, Aisha Elsharkawy, Sharareh Eskandarieh, Oghenowede Eyawo, Sayeh Ezzikouri, Abidemi Omolara Fasanmi, Nelsensius Klau Fauk, Tomas Y Ferede, Eduarda Fernandes, Florian Fischer, Nataliya A Foigt, Morenike Oluwatoyin Folayan, Masoud Foroutan, Joel Msafiri Francis, Takeshi Fukumoto, Mohamed M Gad, Biniyam Sahiledengle Geberemariyam, Berhe Gebremichael, Hailay Abrha Gesesew, Lemma Getacher, Mustefa Glagn, Mahaveer Golechha, Philimon N Gona, Mohammed Ibrahim Mohialdeen Gubari, Brian J Hall, Abdiwahab Hashi, Claudiu Herteliu, Hagos Degefa de Hidru, Ramesh Holla, H Dean Hosgood, Naznin Hossain, Mostafa Hosseini, Mowafa Househ, Segun Emmanuel Ibitoye, Olayinka Stephen Ilesanmi, Irena M Ilic, Milena D Ilic, Seyed Sina Naghibi Irvani, Chidozie C D Iwu, Ihoghosa Osamuyi Iyamu, Vardhmaan Jain, Mihajlo Jakovljevic, Ravi Prakash Jha, Jacek Jerzy Jozwiak, Ali Kabir, Tanuj Kanchan, André Karch, Ayele Semachew Kasa, Getinet Kassahun, Gbenga A Kayode, Bayew Kelkay, Nauman Khalid, Gulfaraz Khan, Junaid Khan, Md Nuruzzaman Khan, Khaled Khatab, Yun Jin Kim, Adnan Kisa, Sezer Kisa, Sindhura Lakshmi Koulmane Laxminarayana, Ai Koyanagi, Kewal Krishan, Barthelemy Kuate Defo, Nuworza Kugbey, Vaman Kulkarni, Om P Kurmi, Dian Kusuma, Hmwe Hmwe Kyu, Carlo La Vecchia, Dharmesh Kumar Lal, Jennifer O Lam, Iván Landires, Jeffrey V Lazarus, Paul H Lee, Platon D Lopukhov, Deepak Madi, Azeem Majeed, Afshin Maleki, Abdullah A Mamun, Navid Manafi, Mohammad Ali Mansournia, Francisco Rogerlândio Martins-Melo, Birhanu Geta Meharie, Mulugeta Melku, Walter Mendoza, Endalkachew Worku Mengesha, Tuomo J Meretoja, Tomislav Mestrovic, Ted R Miller, Osama Mohamad, Yousef Mohammad, Abdollah Mohammadian-Hafshejani, Jemal Abdu Mohammed, Salahuddin Mohammed, Shafiu Mohammed, Ali H Mokdad, Taklu Marama Mokonnon, Mariam Molokhia, Paula Moraga, Jonathan F Mosser, Sandra B Munro, Ghulam Mustafa, Saravanan Muthupandian, Ahamarshan Jayaraman Nagarajan, Vinod C Nayak, Javad Nazari, Samata Nepal, Frida N Ngalesoni, Georges Nguefack-Tsague, Josephine W Ngunjiri, Jean Jacques Noubiap, Virginia Nuñez-Samudio, Oluwakemi Ololade Odukoya, Felix Akpojene Ogbo, Andrew T Olagunju, Bolajoko Olubukunola Olusanya, Jacob Olusegun Olusanya, Muktar Omer Omer, Abidemi E Emmanuel Omonisi, Obinna E Onwujekwe, Orish Ebere Orisakwe, Nikita Otstavnov, Mayowa O Owolabi, Mahesh P A, Jagadish Rao Padubidri, Smita Pakhale, Adrian Pana, Seithikurippu R Pandi-Perumal, Urvish K Patel, Mona Pathak, George C Patton, Shrikant Pawar, Maarten J Postma, Zahiruddin Quazi Syed, Alireza Rafiei, Fakher Rahim, Mohammad Hifz Ur Rahman, Pradhum Ram, Chhabi Lal Ranabhat, Sowmya J Rao, David Laith Rawaf, Salman Rawaf, Vishnu

Renjith, Nima Rezaei, Ana Isabel Ribeiro, Jennifer M Ross, Susan Fred Rumisha, Rajesh Sagar, Maitreyi Sahu, Marwa Rashad Salem, Abdallah M Samy, Aletta Elisabeth Schutte, Abdul-Aziz Seidu, Mohammed Feyisso Shaka, Kenji Shibuya, K M Shivakumar, Negussie Boti Sidemo, Jasvinder A Singh, Amin Soheili, Oluwaseyi Dolapo Somefun, Muluken Bekele Sorrie, Mu'awiyyah Babale Sufiyan, Eyayou Girma Tadesse, Zemenu Tamir, Animut Tagele Tamiru, Nuno Taveira, Yohannes Tekalegn, Bhaskar Thakur, Musliu Adetola Tolani, Marcos Roberto Tovani-Palone, Alexander C Tsai, Irfan Ullah, Bhaskaran Unnikrishnan, Constantine Vardavas, Yasir Waheed, Richard G Wamai, Yanzhong Wang, Paul Ward, Nuwan Darshana Wickramasinghe, Charles Shey Wiysonge, Temesgen Gebeyehu Wondmeneh, Sanni Yaya, Yigizie Yeshaw, Yordanos Gizachew Yeshitila, Mekdes Tigistu Yilma, Naohiro Yonemoto, Syed Saoud Zaidi, Maryam Zamanian, Mikhail Sergeevich Zastrozhin, Anasthasia Zastrozhina, Simon I Hay, and Laura Dwyer-Lindgren.

Managing the overall research enterprise
John D VanderHeide, Samantha Perkins, Aubrey J Cook, Jacek A Kopec, Ali H Mokdad, Mohsen Naghavi, George C Patton, Roman Topor-Madry, Simon I Hay, and Laura Dwyer-Lindgren.

3 Compliance with the Guidelines for Accurate and Transparent Health Estimates Reporting (GATHER)

Item #	Checklist item	Description of Compliance
**Objectives and funding**		
**1**	Define the indicator(s), populations (including age, sex, and geographic entities), and time period(s) for which estimates were made.	Methods section.
**2**	List the funding sources for the work.	Acknowledgments section.
**Data Inputs**		
For all data inputs from multiple sources that are synthesized as part of the study:		
**3**	Describe how the data were identified and how the data were accessed.	Methods section; Appendix section 4.2.
**4**	Specify the inclusion and exclusion criteria. Identify all ad-hoc exclusions.	Methods section; Appendix section 4.2.
**5**	Provide information on all included data sources and their main characteristics. For each data source used, report reference information or contact name/institution, population represented, data collection method, year(s) of data collection, sex and age range, diagnostic criteria or measurement method, and sample size, as relevant.	Appendix section 4.2; Appendix Tables 4-7.
**6**	Identify and describe any categories of input data that have potentially important biases (e.g., based on characteristics listed in item 5).	Limitations section.
For data inputs that contribute to the analysis but were not synthesized as part of the study:		
**7**	Describe and give sources for any other data inputs.	Appendix section 4.2; Appendix Tables 4-7.
For all data inputs:		
**8**	Provide all data inputs in a file format from which data can be efficiently extracted (e.g., a spreadsheet rather than a PDF), including all relevant meta-data listed in item 5. For any data inputs that cannot be shared because of ethical or legal reasons, such as third-party ownership, provide a contact name or the name of the institution that retains the right to the data.	Available through Global Health Data Exchange: http://ghdx.healthdata.org/record/ihme-data/sub-saharan-africa-hiv-incidence-mortality-geospatial-estimates-2000-2018.
**Data analysis**		

**9**	Provide a conceptual overview of the data analysis method. A diagram may be helpful.	Methods section; Appendix section 4.1; Appendix Figure 1.
**10**	Provide a detailed description of all steps of the analysis, including mathematical formulae. This description should cover, as relevant, data cleaning, data pre-processing, data adjustments and weighting of data sources, and mathematical or statistical model(s).	Methods section; Appendix section 4.
**11**	Describe how candidate models were evaluated and how the final model(s) were selected.	Methods section; Appendix section 4.1.
**12**	Provide the results of an evaluation of model performance, if done, as well as the results of any relevant sensitivity analysis.	Methods section; Appendix section 4.1.
**13**	Describe methods for calculating uncertainty of the estimates. State which sources of uncertainty were, and were not, accounted for in the uncertainty analysis.	Methods section; Appendix section 4.
**14**	State how analytic or statistical source code used to generate estimates can be accessed.	Data sharing section.
**Results and Discussion**		
**15**	Provide published estimates in a file format from which data can be efficiently extracted.	Available through Global Health Data Exchange: http://ghdx.healthdata.org/record/ihme-data/sub-saharan-africa-hiv-incidence-mortality-geospatial-estimates-2000-2018.
**16**	Report a quantitative measure of the uncertainty of the estimates (e.g. uncertainty intervals).	Results section.
**17**	Interpret results in light of existing evidence. If updating a previous set of estimates, describe the reasons for changes in estimates.	Discussion section.
**18**	Discuss limitations of the estimates. Include a discussion of any modelling assumptions or data limitations that affect interpretation of the estimates.	Limitations section.

4 Methods Appendix

4.1 Epidemiological model

To create estimates of HIV incidence and HIV mortality we used a mathematical compartmental model, the Estimation and Projection Package (EPP). Since its inception in the early 2000s, EPP has been a part of the UNAIDS toolbox for modelling HIV epidemics globally.[1] It has the advantage of being able to generate an incidence curve that is fitted to and consistent with the observed prevalence in a given country and is able to do so while requiring relatively few parameters be estimated independently and it is therefore easily generalizable to a wide variety of locations. This section will describe the model structure and the following sections will describe the various key inputs and their provenance.

Over its long history, EPP has gone through a large number of iterations and improvements. The version of EPP used for this analysis is a modified version of the model used by the 2017 Global Burden of Disease Study (GBD). It differs from the current versions in use by UNAIDS[2] and the GBD[3] in that it treats the both sexes, aged 15–49 population as a single population while both the GBD and UNAIDS have moved to an age and sex disaggregated version of EPP (EPP-ASM). The EPP-ASM requires significant additional inputs that are not presently widely available at a fine subnational scale and was thus not used here. A summary of how we have changed the model from previous iterations is provided in section 1.6.

4.1.1 Model overview

At its core, EPP is a compartmental mathematical model, which divides the population into HIV- and HIV+ compartments and then simulates how people move between compartments based on a series of assumptions about HIV transmission and mortality. A graphical representation of the model and its specific compartments can be found in Appendix Figure 1. The model is initiated with a both sexes, aged 15–49 population ($N(t)$). More detail on how we set the base population size is provided in section 2.1 of this document. At each modeled time step:

- A number of individuals become 15 years old and enter into the population ($E(t)$), this number is derived from our base demographic model, see section 2.1.

- That group of individuals is split into the HIV-negative and HIV-positive populations using the HIV prevalence among 15-year olds ($P15(t)$). This parameter is derived from the UNAIDS national Spectrum files.

- HIV incidence in the population aged 15–49 is calculated ($I(t)$) and individuals move from being HIV negative to being HIV positive (see section 1.2).

- Newly infected individuals are distributed between 7 different CD4 cell count categories (j) according to pre-specified probabilities derived from the UNAIDS national Spectrum files.

- HIV-positive individuals progress through the CD4 cell count categories based on transition parameters ($T1(t)$) estimated by the GBD. This transition models increasing disease severity, (see section 1.4).

- HIV-positive individuals who are not on antiretroviral therapy (ART) have the possibility of initiating treatment ($T2(t)$). The number of treatment initiations is determined by the

availability of ART in a given location and the stated treatment policies of the location being modeled (see section 2.3). Information on national policies regarding ART eligibility are available in the UNIADS Spectrum country files.

- Individuals exit the 15–49-year-old population in three ways.
  - Ageing out ($A(t)$). This is applied to all disease, CD4 count, and treatment status categories. This parameter is a feature of our underlying demographic model described in section 2.1.
  - Death due to a cause other than HIV (non-HIV mortality, $\mu(t)$), which is applied to all people in the modeled population (see section 1.4).
  - Death due to HIV ($\mu HIV(t)$). This is applied only to the HIV-positive population and the mortality rate is specific to each CD4 count and treatment category (ij). This is also described in section 1.4.

- After the population has progressed one time step we compare the size of the population to the expected population size the next time step as determined by our demographic model (section 2.1). Any difference is assumed to be due to migration($M(t)$). Here we assume that out-migrants are equally likely to come from any of the disease, CD4 count, and treatment status categories. In-migrants have the same assumption placed on them. As a result, only net migration is considered and it is applied equally to the entire 15-49 year old population.

After we adjust the total population to the appropriate size to begin the next time step of the simulation, the process begins again by aging in a new group of individuals and processing the population as described above. We used a 0.1-year time step, as is standard for other implementations of EPP.

A mathematical expression of the description provided above is given by these differential equations:

$$\frac{dZ(t)}{dt} = E(t)\big(1 - P15(t)\big) - r(t)\frac{Y(t)}{N(t)}\big(1 - \omega\,\alpha(t)\big)Z(t) \\ - \mu(t)Z(t) - A(t)\,Z(t) + M(t)Z(t)$$

$$\frac{dY_{ij}(t)}{dt} = r(t)\frac{Y(t)}{N(t)}\big(1 - \omega\,\alpha(t)\big)Z(t)P_{ij} + E(t)P15_{ij}(t) - \mu(t)Y_{ij}(t) \\ - \mu HIV_{ij}Y_{ij}(t) - A(t)\,Y_{ij}(t) + T1_{ij}(t) + T2_{ij}(t) + M(t)Y_{ij}(t)$$

Demographic parameters:

- $Z(t)$ is the HIV- population;
- $Y(t)$ is the HIV+ population, thus the total HIV+ population is the sum of all $Y_{ij}(t)$;
- i is the treatment status;
- j is the CD4 count category;
- $N(t)$ is the total population aged 15–49;
- $E(t)$ are the new entrants aging into the population;
- $M(t)$ is the net migration rate into or out of the population;
- and $A(t)$ is the rate individuals are aging out of the population.

Epidemic and disease progression parameters:

- $r(t)$ is the transmission rate of HIV per infected and untreated individual;
- $\alpha(t)$ is the ART coverage rate in the age 15–49 population;
- ω is the impact (reduction) of the transmission rate for individuals receiving ART;
- $P_{ij}(t)$ is the probability that a newly infected person will fall into treatment and CD4 category ij;
- $P15_{ij}(t)$ is the probability that a 15-year-old entering the model population is HIV positive and belongs in treatment and CD4 category ij;
- $P15(t)$ is the sum across all $P15_{ij}(t)$ and equals the probability that a 15-year-old entering the population is HIV positive;
- $\mu(t)$ is the non-HIV mortality rate for individuals aged 15–49;
- $\mu HIV_{ij}(t)$ is the HIV mortality rate for a person aged 15–49 in treatment group i and CD 4 category j;
- $T1_{ij}(t)$ is the number of people transitioning into or out of the treatment and CD4 category ij due to disease progression;
- and $T2_{ij}(t)$ is the number of people transitioning into or out of the treatment and CD4 category ij due to treatment initiation.

4.1.2 Initiation of random model components

All locations are initiated in 1970 with an HIV prevalence of 0%, meaning there were no HIV-positive individuals in the location at that time. To begin the HIV epidemic, a random number of individuals (ι) are converted from HIV negative to HIV positive in the epidemic start year. That year is determined by UNAIDS in consultation with individual countries and recorded in the UNAIDS country Spectrum files. For this analysis, we set the same start year in all second-level administrative units within a given country. The prior for the number of initial cases was set as:

$$\log(\iota) \sim \mathrm{Uniform}(\log(1 \cdot 10^{-14}), \log(2.5 \cdot 10^{-5}))$$

The HIV transmission rate ($r(t)$) is the primary parameter that is fitted when using EPP. For this analysis, we utilize the the r-hybrid model described in Eaton *et al.*[2] to model this parameter. In this model the transmission rate is parameterized starting with a logistic decay following the form:

$$\log(r(t)) = r_0 - \frac{r_0 - r_\infty}{1 + e^{-\lambda(t - t_{mid})}}$$

where r_0 is the log of the initial transmission rate, r_∞ is the log of the stabilized transmission rate, λ is the rate of decline in the log transmission rate, and t_{mid} is the inflection point of the logistic decay function. We used the following priors on these 4 parameters:

$$r_0 \sim \mathrm{Normal}(\log(0.35), 0.5)$$
$$r_\infty \sim \mathrm{Normal}(\log(0.09), 0.3)$$
$$\log(\lambda) \sim \mathrm{Normal}(\log(0.2), 0.5)$$
$$t_{mid} \sim \mathrm{Normal}(1993, 5.0)$$

Beginning in 2003, $r(t)$ is parameterized as a piecewise linear spline using a first-order random walk penalty on the spline coefficients. The spline started at time τ_0 = 2003 and the knots (τ_i) were spaced with a 5 year Δ_τ Thus the form for the latter portion of the transmission rate vector can be written as:

$$\log\big(r(t)\big) = \sum_{i=1}^{K} \beta_i \sqrt{\Delta_\tau}\ b_i(t)$$

where:

$$b_i(t) = 0 \text{ if } t \leq \tau_{i-1}$$
$$b_i(t) = t - \tau_{i-1} \text{ if } \tau_{i-1} < t \leq \tau_i$$
$$b_i(t) = 1 \text{ if } t \geq \tau_i$$

The prior on the coefficients is: $\beta_i \sim \text{Normal}(0, 0.06)$

To ensure a smooth transition between the two parameterization regimes, a 5-year linear interpolation is used over the period 2003-2008 such that before 2003 the logistic specification is used; after 2008 the random walk specification is used; and between those years a weighted average of the two forms is used.

Incidence is then calculated using the following formula:

$$I(t) = r(t)\frac{Y(t)}{N(t)}\big(1 - \omega\,\alpha(t)\big)Z(t)$$

where $I(t)$ is the number of incident HIV cases, and all other parameters are defined as above.

4.1.3 Fitting

As stated earlier, EPP is essentially fitting the transmission rate to the prevalence using a series of assumptions about how the different epidemic indicators relate to each other. This fitting is achieved using the Bayesian Incremental Mixture Importance Sampling (IMIS) solver.[4] IMIS compares the HIV prevalence output of each EPP simulation to the HIV prevalence from our Model Based Geostatistical (MBG) prevalence model (described in section 2.2 below). The MBG prevalence model produces 1,000 draws of HIV prevalence for each administrative unit-year pair that we modeled. We assume that the logit-transform of these draws are multivariate normal, with mean and covariance calculated from the 1,000 draws. The likelihood for each EPP simulation can then be written:

$$\text{logit}(\hat{p}) \sim \text{MVN}\big(\text{logit}\big(p(\theta)\big), \textstyle\sum \hat{p}\big)$$

where:

- $\hat{p}$ is the vector of mean HIV prevalence produced by the MBG model;
- $p(\theta)$ is the vector of HIV prevalence estimates produced when running EPP with input vector θ;
- and $\sum \hat{p}$ is the covariance matrix of the logit transformed 1000 MBG draws of HIV prevalence.

IMIS then uses this likelihood to develop a 1,000 draw posterior distribution for the force of infection vector ($r(t)$). The 1000 draws of force of infection are then used to produce 1,000 draws of HIV incidence, prevalence, and mortality per EPP simulation.

4.1.4 Disease progression and mortality calculations

Disease progression is a critical component of HIV mortality, as individuals with lower CD4+ cell counts have higher HIV mortality than do individuals with higher CD4+ cell counts. To reflect this, EPP divides the HIV-positive population into seven CD4 cell count categories (CD4 counts <50, 50-99, 100-199, 200-249, 250-349, 350-500, and >500). The rate at which HIV-positive individuals transition between the CD4 count categories is determined by a series of transition parameters. For this analysis, we use the parameter values estimated by the GBD[5] using a meta-analysis of HIV progression in treated and un-treated individuals. They estimate the likelihood that an individual will transition between CD4 categories given that individual's age, sex, and treatment status. To best approximate the value of these parameters for the entire HIV-positive population (ages 15–49) in a given location we use the age and sex distribution of the underlying population and country-age-sex-specific incidence rate ratios (using 25-29 year-old men as the base) from GBD to weight the transition parameters such that the final transition parameters are the weighted average of the age- and sex-specific transition parameters and the weight used is:

$$w_{as}(t) = {N_{as}(t) \cdot IRR_{as}(t)} \Big/ {\sum N_{as}(t) \cdot IRR_{as}(t)}$$

where:

- $N_{as}(t)$ is the age- and sex-specific population for age group a and sex s in our modelled location;
- and $IRR_{as}(t)$ is the age- and sex-specific national incidence rate ratio for age group a and sex s.

We similarly use the GBD estimates for the HIV+ on- and off-ART mortality rates which are also based on meta-analysis of existing studies.[4] These estimates are also age and sex specific and are combined to a representative value for ages 15–49 and both sexes combined using the same method outlined above.

The all-cause non-HIV mortality rate is estimated by the GBD at the country level for each age and sex. The age and sex population breakdowns of each administrative unit were used to re-weight those estimates to create an estimate of this parameter for both sexes, ages 15–49 within each area.

The GBD meta-analyses of CD4 transition parameters and HIV mortality rates produce 1000 draws of these parameters. To properly account for uncertainty in those parameters without having to run the prohibitively large number of simulations that would come from utilizing all of the posterior draws from the meta-analyses, we down sample to 100 draws and run 100 EPP simulations using those 100 draws.

4.1.5 Final posterior creation and post estimation

As described above, we run 100 simulations of EPP to account for uncertainty in inputs including the disease progression and mortality parameters; each simulation results in 1,000 draws of HIV prevalence, incidence, and mortality. To create a single posterior distribution for prevalence, incidence, and mortality for each unit, we sample 10 draws from each of the 100 simulations and then combine these draws into a single 1000 draw posterior for each indicator.

This strategy of running 100 simulations of EPP and utilizing 10 draws from each simulation is a departure from the process typically utilized by the GBD, which involves running 1,000 simulations and utilizing 1 draw from each simulation. This approach was too computationally intensive to implement for all 4087 second-level administrative units, which motivated the change to 100 simulations of EPP

and 10 draws from each simulation. We conducted a sensitivity analysis to confirm that the down sampling of the HIV+ mortality and CD4 progression parameters and the creation of the hybrid posterior was an appropriate approximation of using the full 1,000 draws. To do so, we first ran EPP simulations in six countries with varying epidemic structures using all the full 1,000 draws of the HIV+ and CD4 progression parameters and created a final single posterior by selecting an individual draw from each EPP simulation. We then repeated that process running another 1000 simulations using the same inputs and creating another 1000 draw posterior to assess the level of variability between two different runs of EPP which used identical inputs. Finally, we ran a third model in these six countries which used identical inputs with the exception of the HIV+ mortality and CD4 progression parameters: in place of the full 1000 draws of the HIV+ mortality parameters we selected 100 at random and proceeded to create a 1000 draw EPP posterior as described above. We used the difference in mean level and the difference in uncertainty interval width to assess if the differences between the baseline 1000 simulation model and the second 1000 simulation model were similar in magnitude to the differences between the baseline 1000 simulation model and the 100 simulation model. We determined that the differences were minimal and thus the model using only 100 draws of the HIV+ mortality parameters was sufficient. The results of this sensitivity analysis are shown in Appendix Table 1.

For consistency with national-level estimates utilizing much of the same underlying data, we calibrate our results to match the estimates from the GBD 2019.[3] To do so, we first find the population-weighted average of the incidence and mortality indicators across all units within a country, then calculate the ratio of this weighted average to the GBD national estimate for corresponding indicator, and finally scale each subnational until by multiplying each draw for each indicator by this ratio. After this is completed, the population-weighted average of HIV incidence and HIV mortality across all units will match the corresponding GBD estimates for a given country and year. In cases where subnational (typically first-level administrative unit) estimates are available from the GBD, we carry out the same raking procedure at the subnational level reported by GBD. The final posterior distributions were summarized via the mean, 2.5 percentile, and 97.5 percentiles of the calibrated draws.

4.1.6 Summary of changes from previous versions of EPP.

In summary, we made the following modifications to EPP in order to facilitate generating estimates for the second-level administrative units:

1. As implemented by UNAIDS[1,6–8] and the GBD,[5,9,10] EPP fits to household survey data and Antenatal Care (ANC) clinic data. The geographic and temporal coverage of this data is too sparse to allow EPP to fit reasonable epidemics in all second-level administrative units in our countries of interest. We used an MBG approach[11] to create estimates of HIV prevalence for all second-level administrative units we wished to model for the time period 1995-2017.

2. Because the prevalence time-series used was derived from a single model rather than a series of independent observations we converted the survey likelihood from a summation of univariate likelihoods to one multivariate normal likelihood.

3. Because internal migration between second-level administrative units is important for our model and exceedingly difficult to measure, we chose to use the approach now being adopted by UNAIDS and the GBD in the EPP-ASM model[2] mentioned above, simply to adjust the population and the end of each time step to the expected population in the next time step using a simple scalar. This

removes the need to explicitly model migration and instead relies on the more certain population count estimates in each 2nd administrative unit.

4. Previous implementations of the GBD EPP model only output HIV incidence and not HIV mortality. We implemented tracking of HIV mortality to allow us to create estimates of prevalence, incidence, and mortality all from EPP.

5. We implemented the new r-hybrid parameterization of the transmission rate in the age 15–49 version of EPP. This has previously been implemented for EPP-ASM, but not EPP.

4.2 Data inputs

4.2.1 Administrative boundaries and demographic data

Our implementation of the EPP epidemic model operates on defined populations at the second administrative level. To delineate the second-level administrative units that we would be modelling we used the Global Administrative Layers (GADM) 3.6 release[12] as our base and then made appropriate edits when we encountered quality issues or were informed by partners of boundary changes. A list of countries that have been edited and the nature of these edits is available in Appendix Table 2.

EPP requires population estimates for all years 1970 to 2018. To estimate the spatial distribution of the population going back to the start year of our EPP simulations, age- and sex-specific WorldPop population rasters[13] that had been aggregated to approximately 5x5km pixels were backcast by calculating the pixel-level exponential rate of change between the years 2000 and 2005 and then applying that annual rate of change going back to 1970. Age- and sex-specific populations for each second-level administrative unit were created by aggregating the WorldPop raster to the modified GADM shapes, utilizing a fractional aggregation process where the population of a given pixel that spanned the boundary of two or more second-level administrative units was divided between those units based on the proportion of the pixel area that fell into each administrative unit. The age- and sex-specific populations for second-level administrative areas were then scaled to the national population estimates derived from the GBD.[14] This resulted in age- and sex-specific population estimates for each second-level administrative unit which aligned with the GBD national population size and structure. Aligning to the GBD national totals allowed us to incorporate information from the historical censuses and shocks modelling that are part of the GBD population estimation process. This is a critical step to improve our population time-series as the annual growth rate between 2000 and 2005 is only an approximate predictor of population in 1970. Despite the power that we gain from aligning with the GBD we do not expect our historical population estimates to be particularly accurate, but this has little impact on our estimates in later years as these are primarily informed by the prevalence trend post-1995 and the ART scale up in the mid-2000s.

4.2.2 HIV prevalence and PLHIV for fitting

To estimate HIV prevalence and the number of people living with HIV (PLHIV) in each administrative unit where we would be fitting EPP we replicated the analytical process of Dwyer-Lindgren *et al*.[11] We began by updating our database of all available HIV seroprevalence survey data for both sexes, ages 15–49 and ANC sentinel surveillance data in the 44 African countries modelled. The resulting geopositioned data set of 29,072 survey observations and 11,710 site years of ANC-SS observations was modelled using the model based geostatistical framework previously published[15–17] to create a time-series for each second-level administrative unit in our modelled countries. Survey seroprevalence data can be found in

Appendix Table 4, ANC-SS seroprevalence data can be found in Appendix Table 5, and data used to build covariate data layers can be found in Appendix Table 6.

The major difference between the analysis used here and previously published work is that since EPP is sensitive to the peak HIV prevalence year[5] and we sought to present estimates of HIV incidence over the period 2000-2018, we included HIV prevalence data back to 1995, allowing us to produce prevalence estimates from 1995-2018. Including this extra data improved the time trend information available to EPP, allowing us to improve our year 2000 estimates.

4.2.3 HIV treatment data

To create the ART input we conducted an extensive HIV treatment data-seeking exercise to identify sources of these data. We identified three main sources of data. The data source with the best temporal and geographic coverage is the UNAIDS Spectrum country files which generally report a full time-series of either national or first-level administrative unit counts of individuals aged 15–49 receiving ART. These estimates are developed in coordination with country governments and were used as the authoritative standard for the national or first-level administrative unit ART totals. The main disadvantage of this data source for the purposes of our analysis is that it is never available for second-level administrative units.

The second source of ART information was country-level reports or Health Management Information Systems databases. To identify these type of data, we searched the GHDx[18] for records that were tagged with the "Antiretroviral therapy" keyword. To supplement this, we searched the Ministry of Health, National Statistics Bureau, and National AIDS Control Board websites (or their analogous agency) in each of the countries that we are modelling. If data were not publically available on the website, we sent emails to the agency to seek additional information. We also reached out to members of the GBD collaborator network[19] who indicated HIV/AIDS as an area of expertise and had country-specific expertise. Sources identified through this process were used when they provided greater geographic granularity than the UNAIDS data and provided a total within 10% of the national or first-level administrative unit estimates from UNAIDS.

Our third data source was the data dashboards for the U.S. President’s Emergency Plan for AIDS Relief (PEPFAR).[20] These data were considered in cases where they were able to improve the geographic specificity or temporal coverage of the data released by the national governments and the national or first-level administrative unit total was consistent with the total from the UNAIDS Spectrum country files. All together, we were able to identify 39 data sources covering 14 country years of data with improved geographic granularity as compared to the UNAIDS data series. These sources are described in Appendix Table 7.

In the few situations where the ART data were available at a finer geographic resolution (geolocated facility or third-level administrative unit), we aggregated those data to the second-level administrative units. In the much more common situation where ART data were available at a larger spatial scale than the second-level administrative unit, we assumed that ART coverage was constant across that larger spatial unit to split the tabulations made on larger boundaries down to second-level administrative units. To achieve this, we multiplied the number of ART patients in the larger unit by the fraction of the total PLHIV in that larger unit which lived in each of the smaller units of interest. This had the effect of distributing the number of people on ART from the larger spatial unit to the smaller spatial units while maintaining the same underlying ART coverage rate.

In most cases, the second-level administrative unit ART information had significant temporal gaps, particularly prior to 2010. To fill in these gaps, we developed a model that predicts the ART coverage in a given second-level administrative unit as a function of the ART coverage of the larger unit that it is a part of for which we have a complete time-series. In cases where the only time-series was at the national level we fit second-level administrative ART coverage as a function of national coverage and in cases where we had complete first-level administrative unit time-series we estimated second-level administrative unit ART coverage as a function of first-level administrative ART coverage. The model is structured as:

$$Y_{it} \sim \mathrm{Normal}(\mu_{it}, \sigma)$$
$$\mu_{it} = (\beta + \gamma_i + z_i) \cdot C_t$$

where:

- Y_{it} is the observed ART coverage in area i in year t;
- μ_{it} is the mean ART coverage in area i and year t;
- C_t is the ART coverage for the larger spatial unit (national or first-level administrative unit) in year t;
- β is a fixed effect describing the general relationship between national ART coverage and ART coverage among second-level administrative units;
- γ_i and z_i are spatially unstructured and structured, respectively, random effects describing area-specific deviations in the relationship between national ART coverage and second-level administrative unit ART coverage.

No intercept was included in this model because we expect ART coverage to be 0 in all second-level administrative units when the national ART coverage is 0.

The model was fit in R-INLA[21] using the BYM2[22] model to “borrow strength” from the geographic pattern in ART coverage rates while still allowing for non-spatially structured variation. We used first-order queen contiguity of the spatial units to which we had mapped the ART information to form the graph for the spatial model. We chose this model over the classic BYM model because it parameterizes the relationship between the spatially structured random effect γ_i and the unstructured random effect z_i in terms of two hyperparameters: τ which is the marginal precision and φ which is the portion of the marginal variance described by the spatially structured random effect. This change improves the interpretability of the hyperparameters. We used the uninformative default penalized complexity priors[22,23] available in INLA for these hyperparameters:

$$\varphi = PC(0.5, 0.5)$$
$$\tau = PC(1, 0.01)$$

In the first case, this prior indicates a 50% probability that 50% or less of the variation is spatially autocorrelated. In the second, this prior indicates a 1% chance that the log precision is less than 1. Appendix Table 8 displays the posteriors for these hyperparameters as well as the precision for the Gaussian observations ($1/\sigma^2$) and the fixed effect (β) in each country-specific model that we fit.

This model was used to make predictions of ART coverage in each second-level administrative unit. We then converted these predictions into counts using the PLHIV numbers coming from the geostatistical prevalence model described above. To ensure consistency with the UNAIDS national time-series we

scaled the modeled total alive on ART numbers in a given UNAIDS geography (first-level administrative unit or national) to the UNAIDS reported total.

Appendix Figures

Appendix Figure 1: EPP schematic

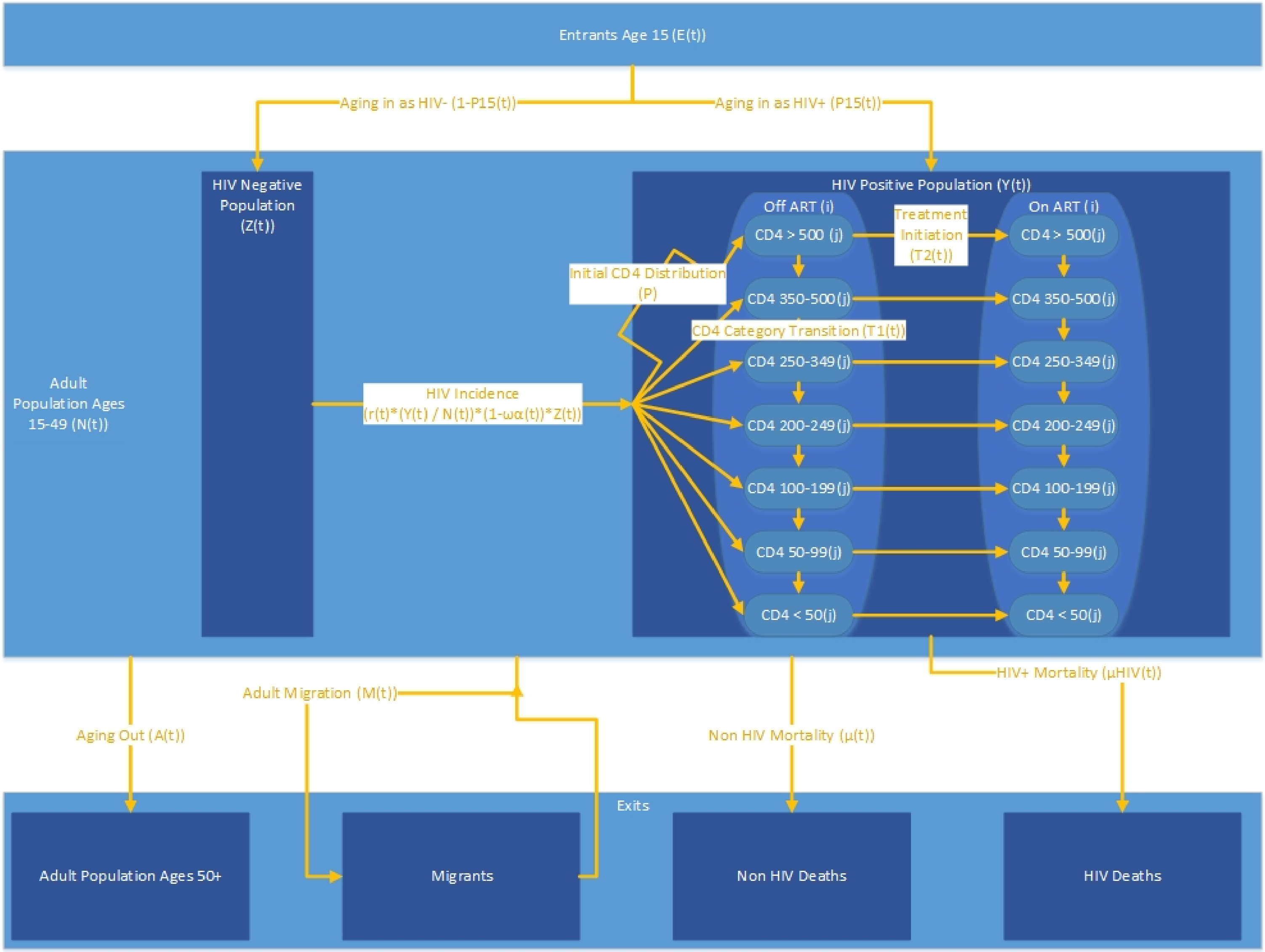

Appendix Figure 1. EPP schematic. Flowchart showing the movement of people through the different compartments of the Estimation and Projection Package (EPP) model used to produce HIV incidence and HIV mortality estimates.

Appendix Figure 2: Impact of subnational ART data on HIV incidence estimates

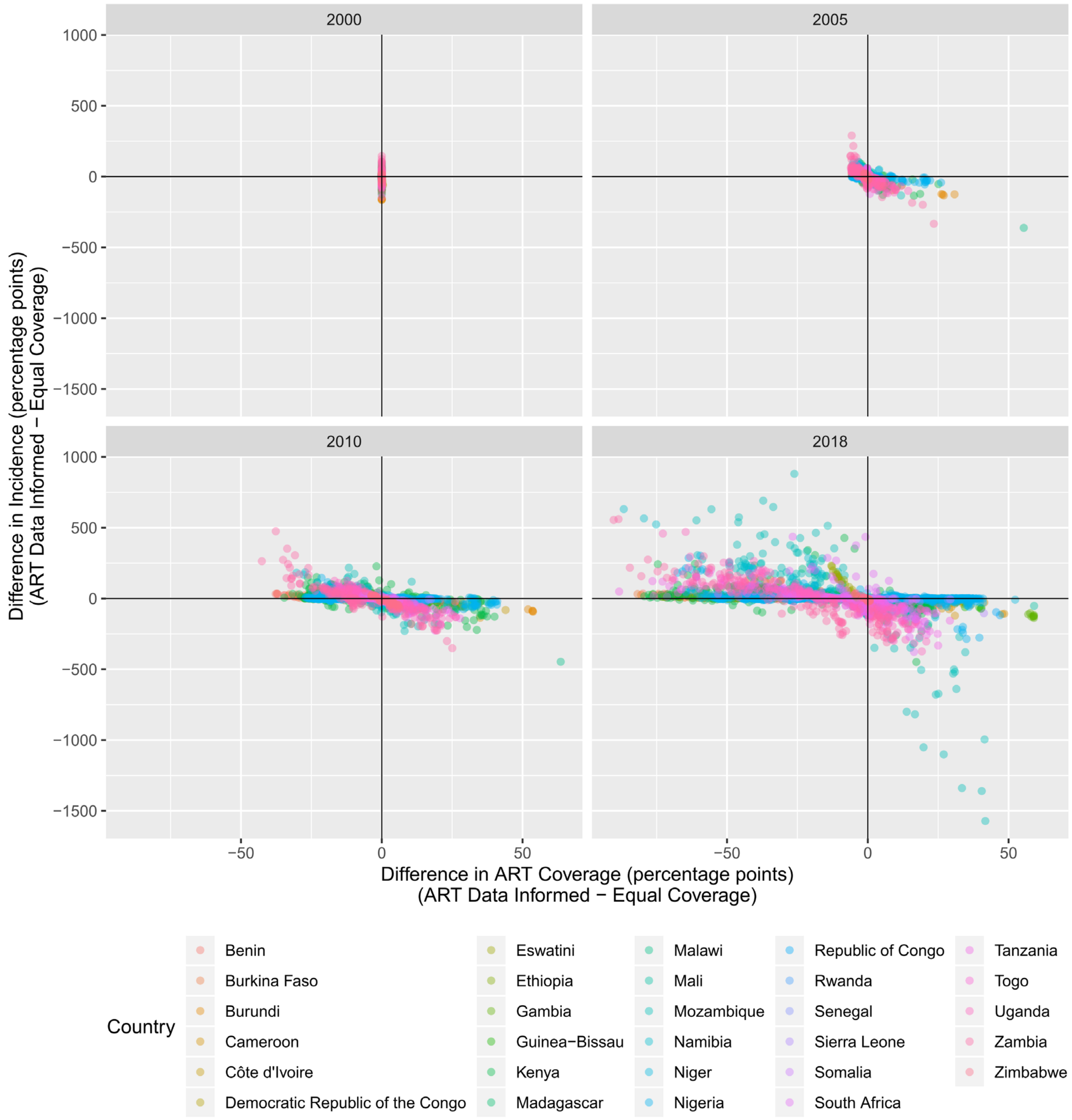

Appendix Figure 2. Impact of subnational ART data on HIV incidence estimates. Difference in ART coverage as a % of people living with HIV scattered against the difference in HIV incidence rate estimate measures in cases per 100,000 people. “ART Data Informed” are estimates made with subnational ART data, “Equal Coverage” are estimates made assuming the national level of ART coverage in all subnational units.

Appendix Figure 3: Impact of subnational ART data on HIV mortality estimates

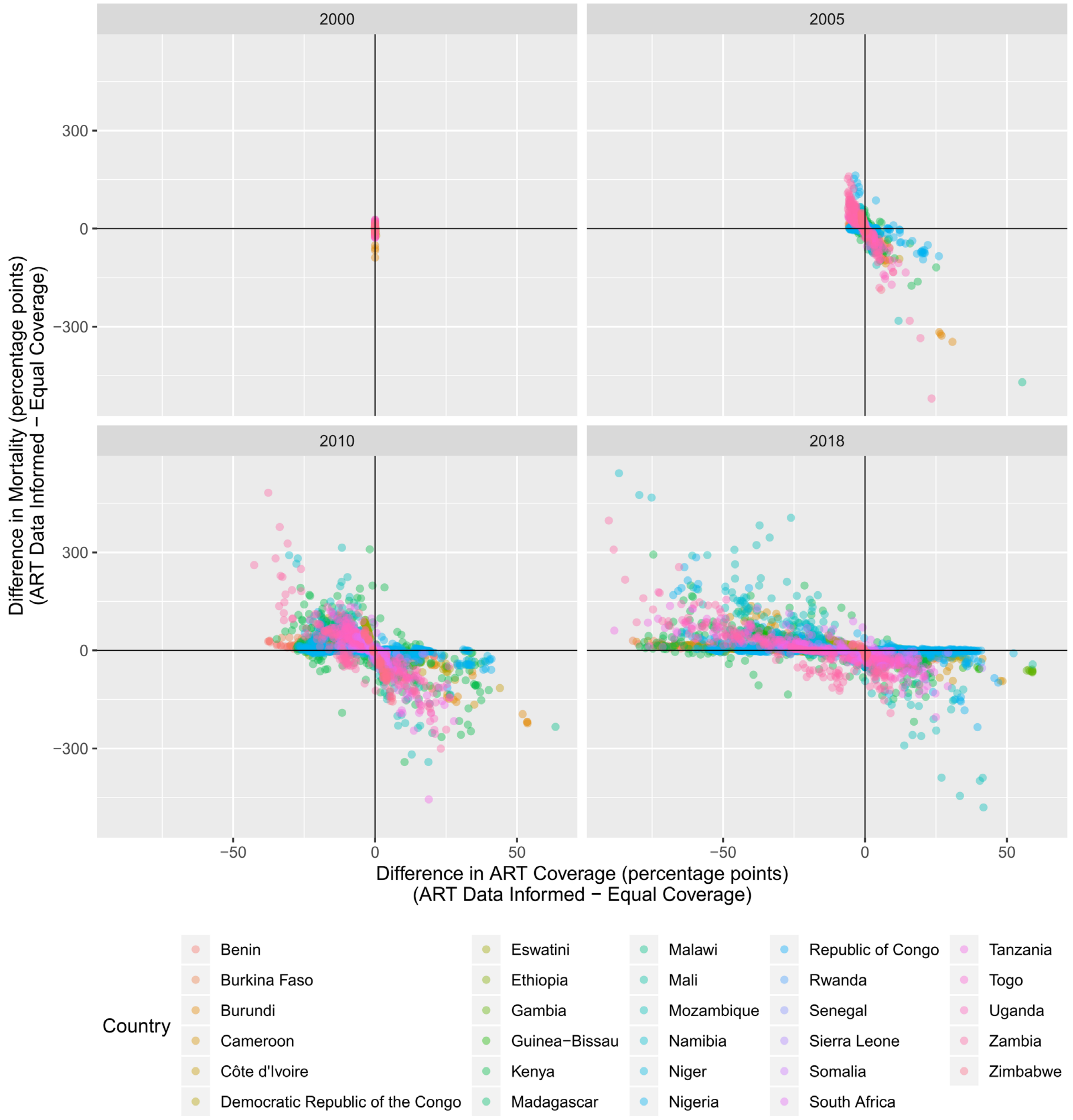

Appendix Figure 3. Impact of subnational ART data on HIV mortality estimates. Difference in ART coverage as a % of people living with HIV scattered against the difference in HIV mortality rate estimate measured in deaths per 100,000 people. “ART Data Informed” are estimates made with subnational ART data, “Equal Coverage” are estimates made assuming the national level of ART coverage in all subnational units.

Appendix Figure 4: Incidence, mortality, prevalence, and ART coverage by second-level administrative unit in 2000

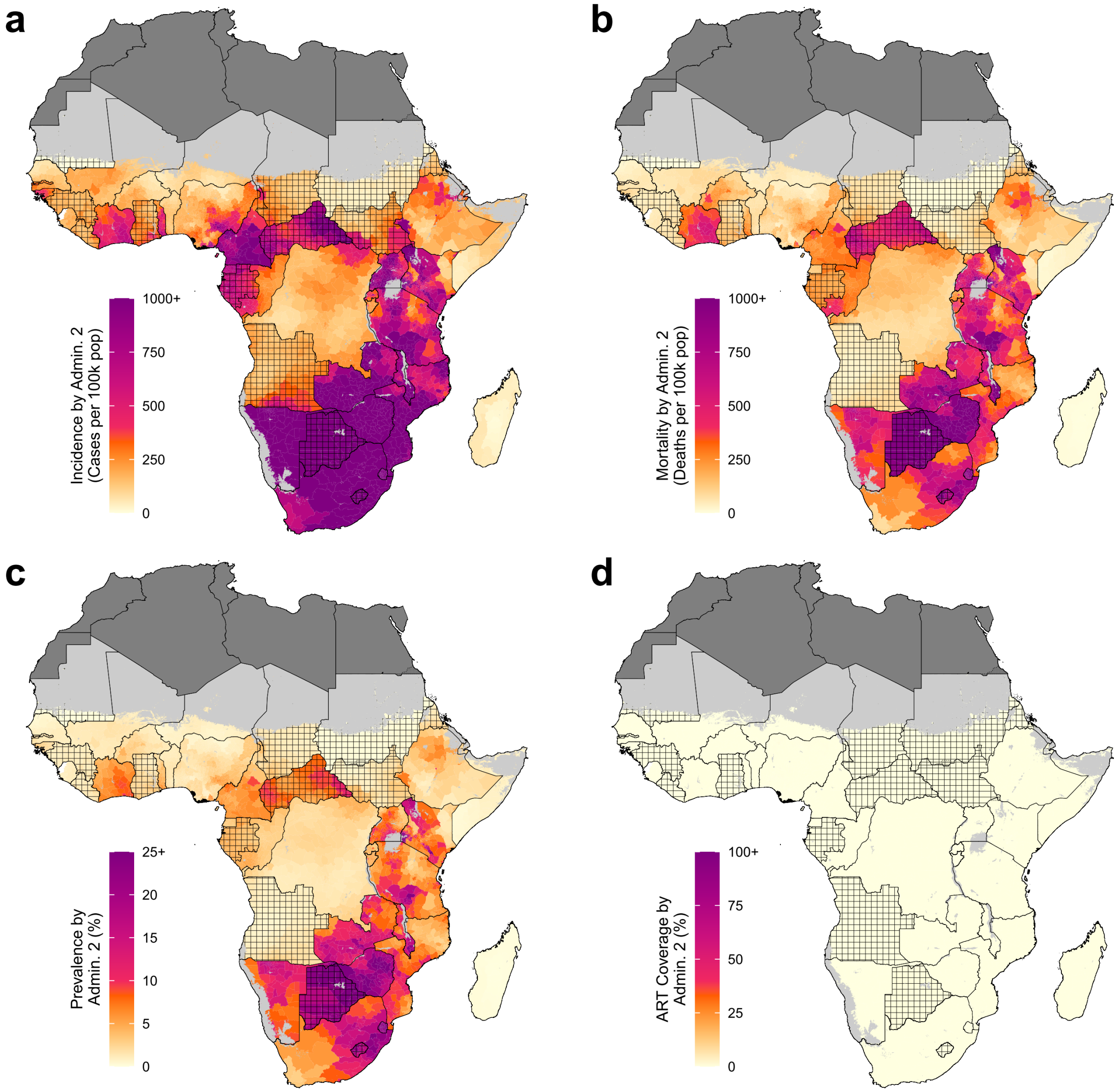

Appendix Figure 4. Incidence, mortality, prevalence, and ART coverage by second-level administrative unit in 2000. HIV incidence rate (**a**), HIV mortality rate (**b**), HIV prevalence (**c**) and ART coverage (**d**) by second-level administrative unit in 2000. Lakes and areas with fewer than ten people per 1 × 1 km and classified as barren or sparsely vegetated are colored light grey.[12,13,24–27] Areas in dark grey were not included in the analysis. Estimates in areas that are hatched are based on national, rather than subnational, ART coverage estimates only (see methods).

Appendix Figure 5: Incidence, mortality, prevalence, and ART coverage by second-level administrative unit in 2005

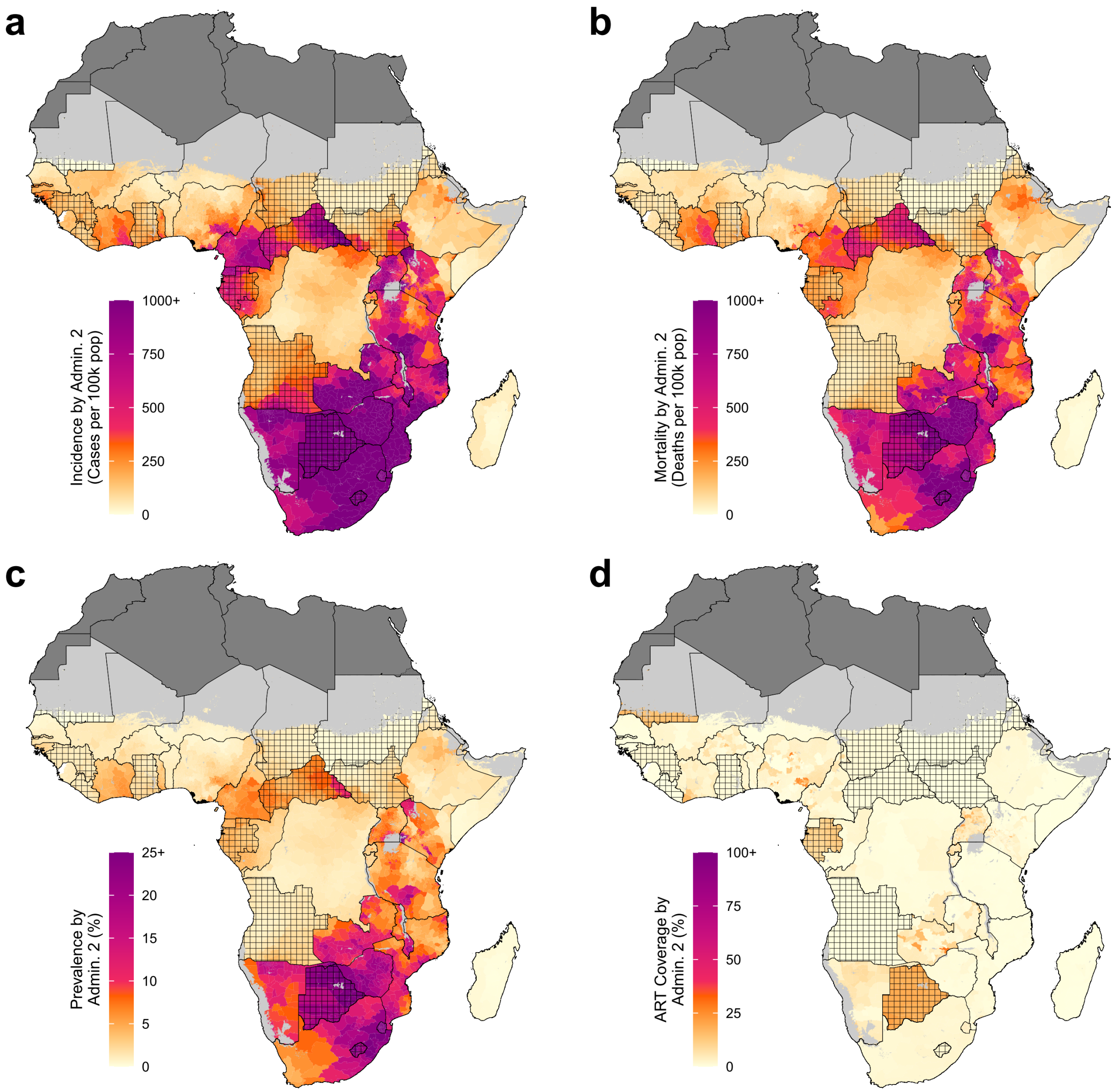

Appendix Figure 5. Incidence, mortality, prevalence, and ART coverage by second-level administrative unit in 2005. HIV incidence rate (**a**), HIV mortality rate (**b**), HIV prevalence (**c**) and ART coverage (**d**) by second-level administrative unit in 2005. Lakes and areas with fewer than ten people per 1 × 1 km and classified as barren or sparsely vegetated are colored light grey.[12,13,24–27] Areas in dark grey were not included in the analysis. Estimates in areas that are hatched are based on national, rather than subnational, ART coverage estimates only (see methods).

Appendix Figure 6: Incidence, mortality, prevalence, and ART coverage by second-level administrative unit in 2010

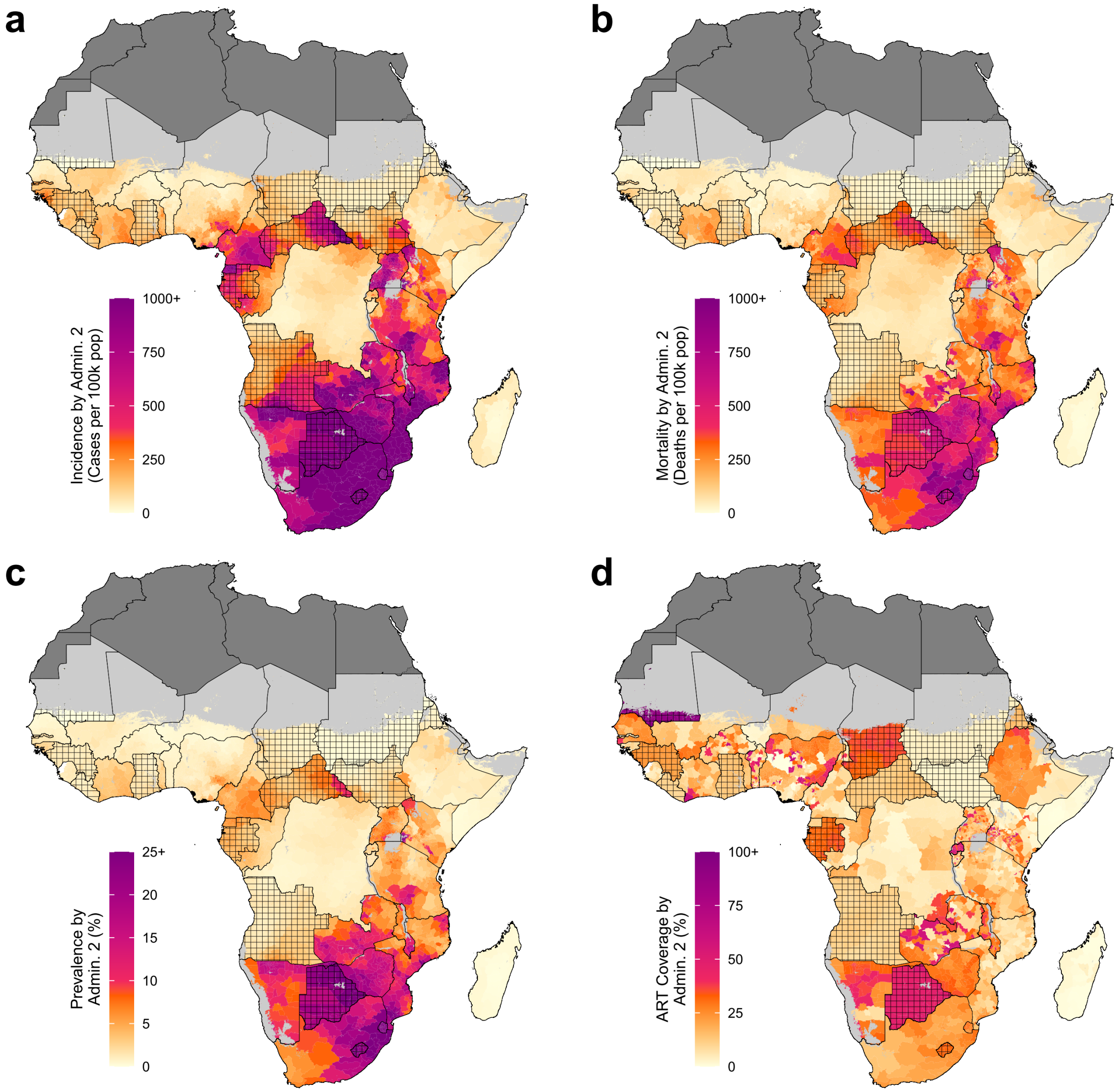

Appendix Figure 6. Incidence, mortality, prevalence, and ART coverage by second-level administrative unit in 2010. HIV incidence rate (**a**), HIV mortality rate (**b**), HIV prevalence (**c**) and ART coverage (**d**) by second-level administrative unit in 2010. Lakes and areas with fewer than ten people per 1 × 1 km and classified as barren or sparsely vegetated are colored light grey.[12,13,24–27] Areas in dark grey were not included in the analysis. Estimates in areas that are hatched are based on national, rather than subnational, ART coverage estimates only (see methods).

Appendix Figure 7: Incidence, mortality, prevalence, and ART coverage by second-level administrative unit in 2018

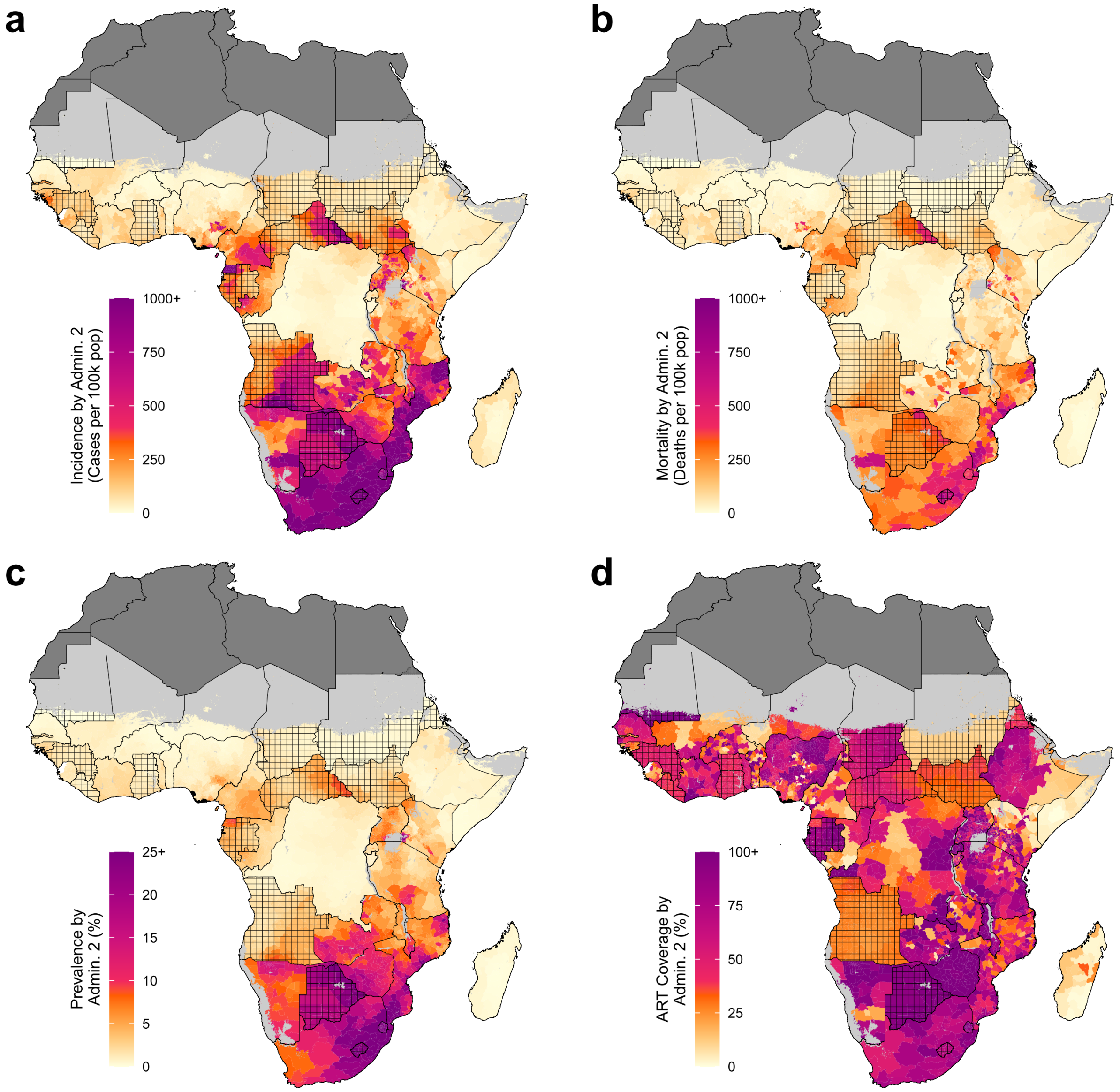

Appendix Figure 7. Incidence, mortality, prevalence, and ART coverage by second-level administrative unit in 2018. HIV incidence rate (**a**), HIV mortality rate (**b**), HIV prevalence (**c**) and ART coverage (**d**) by second-level administrative unit in 2018. Lakes and areas with fewer than ten people per 1 × 1 km and classified as barren or sparsely vegetated are colored light grey.[12,13,24–27] Areas in dark grey were not included in the analysis. Estimates in areas that are hatched are based on national, rather than subnational, ART coverage estimates only (see methods).

Appendix Figure 8: HIV incidence rate among individuals aged 15–49 in 2018 with uncertainty

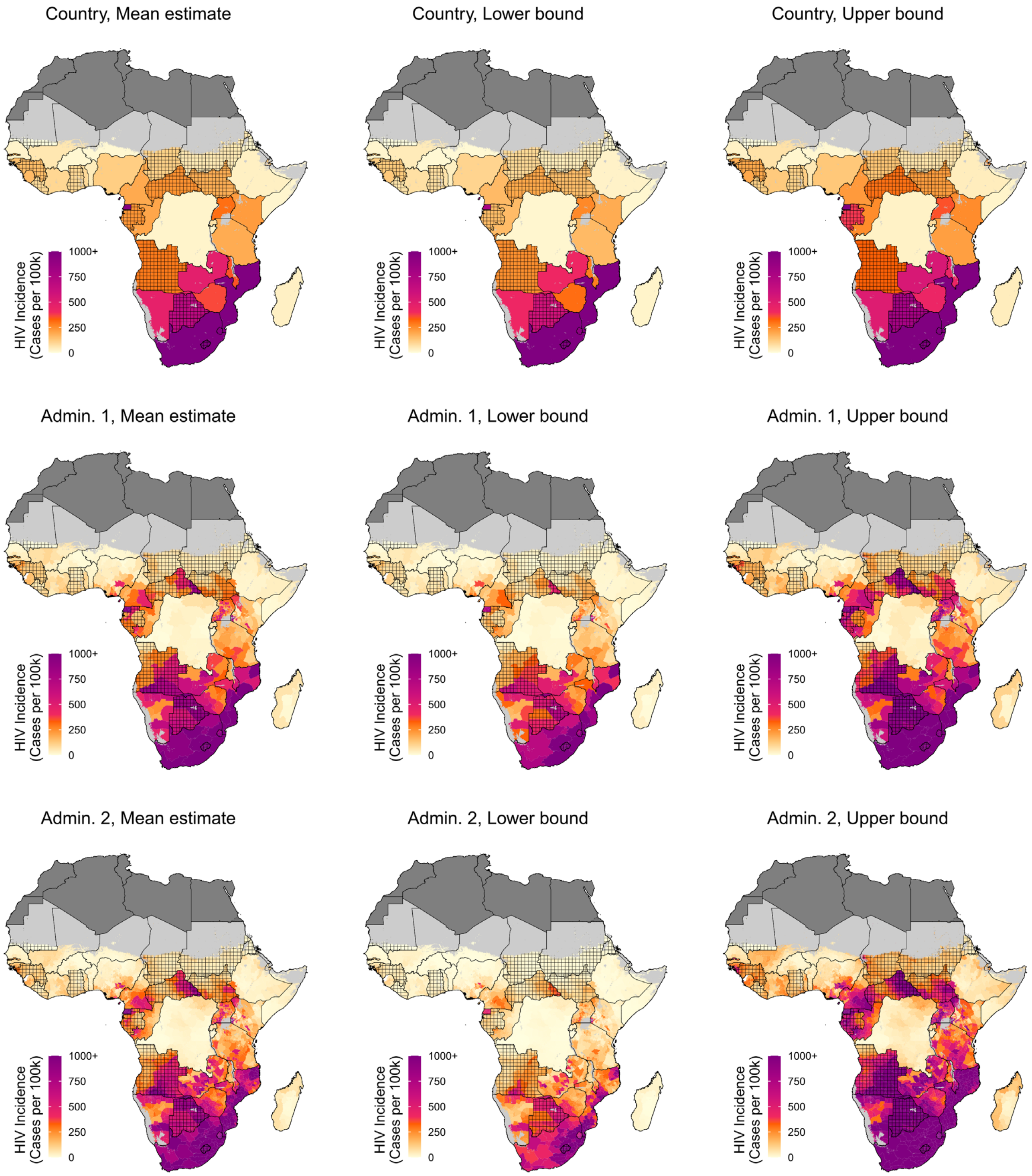

Appendix Figure 8. HIV incidence rate among individuals aged 15–49 in 2018 with uncertainty. HIV incidence rate among individuals aged 15–49 by country (first row), first-level administrative unit (second row), and second-level administrative unit (third row), with mean estimates (first column) and lower (second column) and upper (third column) 95% uncertainty bounds. Lakes and areas with fewer

than ten people per 1 × 1 km and classified as barren or sparsely vegetated are colored light grey.[12,13,24–27] Areas in dark grey were not included in the analysis. Estimates in areas that are hatched are based on national, rather than subnational, ART coverage estimates only (see methods).

Appendix Figure 9: HIV mortality rate among individuals aged 15–49 in 2018 with uncertainty

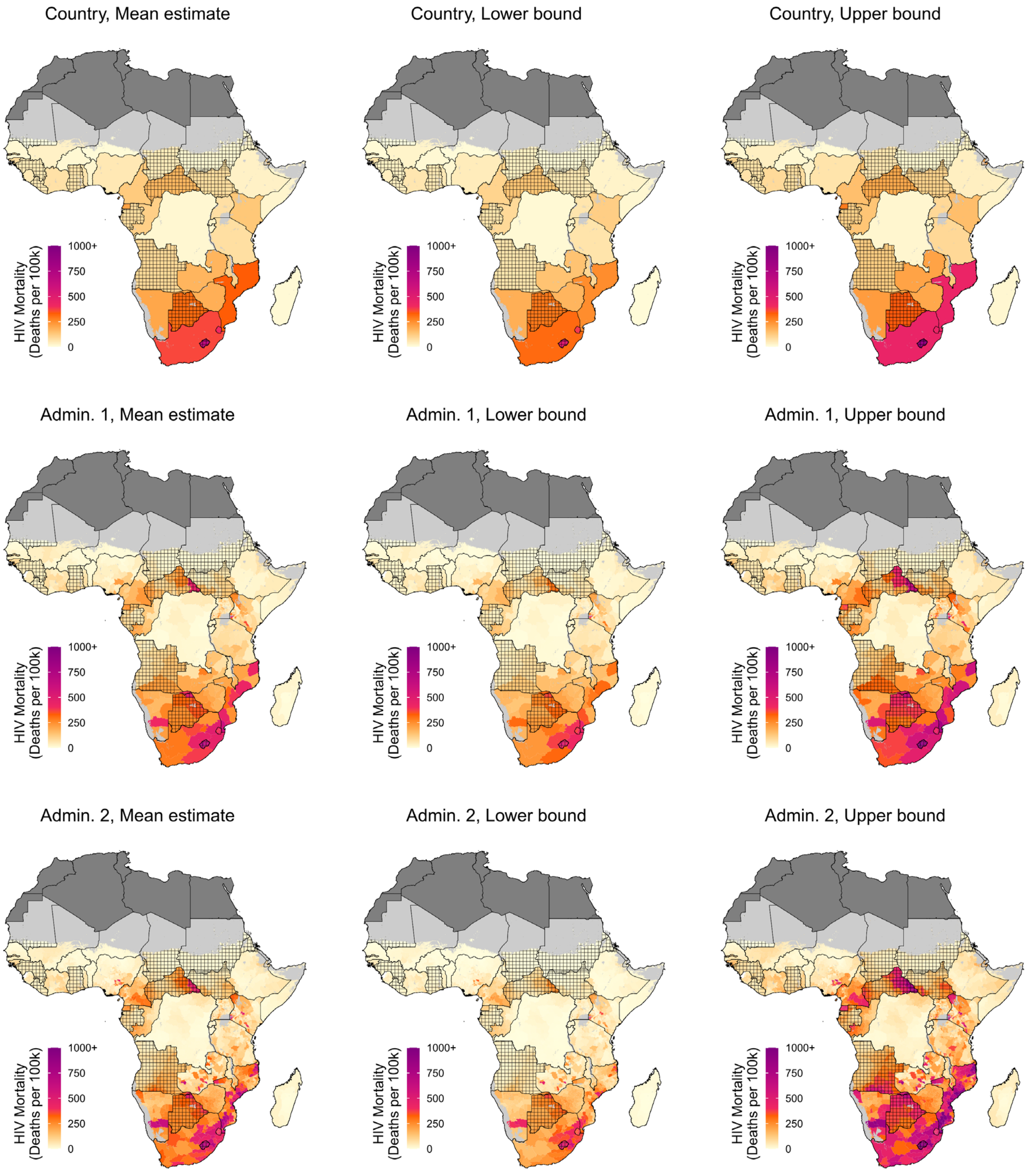

Appendix Figure 9. HIV mortality rate among individuals aged 15–49 in 2018 with uncertainty. HIV mortality rate among individuals aged 15–49 by country (first row), first-level administrative unit (second row), and second-level administrative unit (third row), with mean estimates (first column) and lower (second column) and upper (third column) 95% uncertainty bounds. Lakes and areas with fewer

than ten people per 1 × 1 km and classified as barren or sparsely vegetated are colored light grey.[12,13,24–27] Areas in dark grey were not included in the analysis. Estimates in areas that are hatched are based on national, rather than subnational, ART coverage estimates only (see methods)

Appendix Figure 10: Percent reduction in the HIV incidence rate from 2010 to 2018

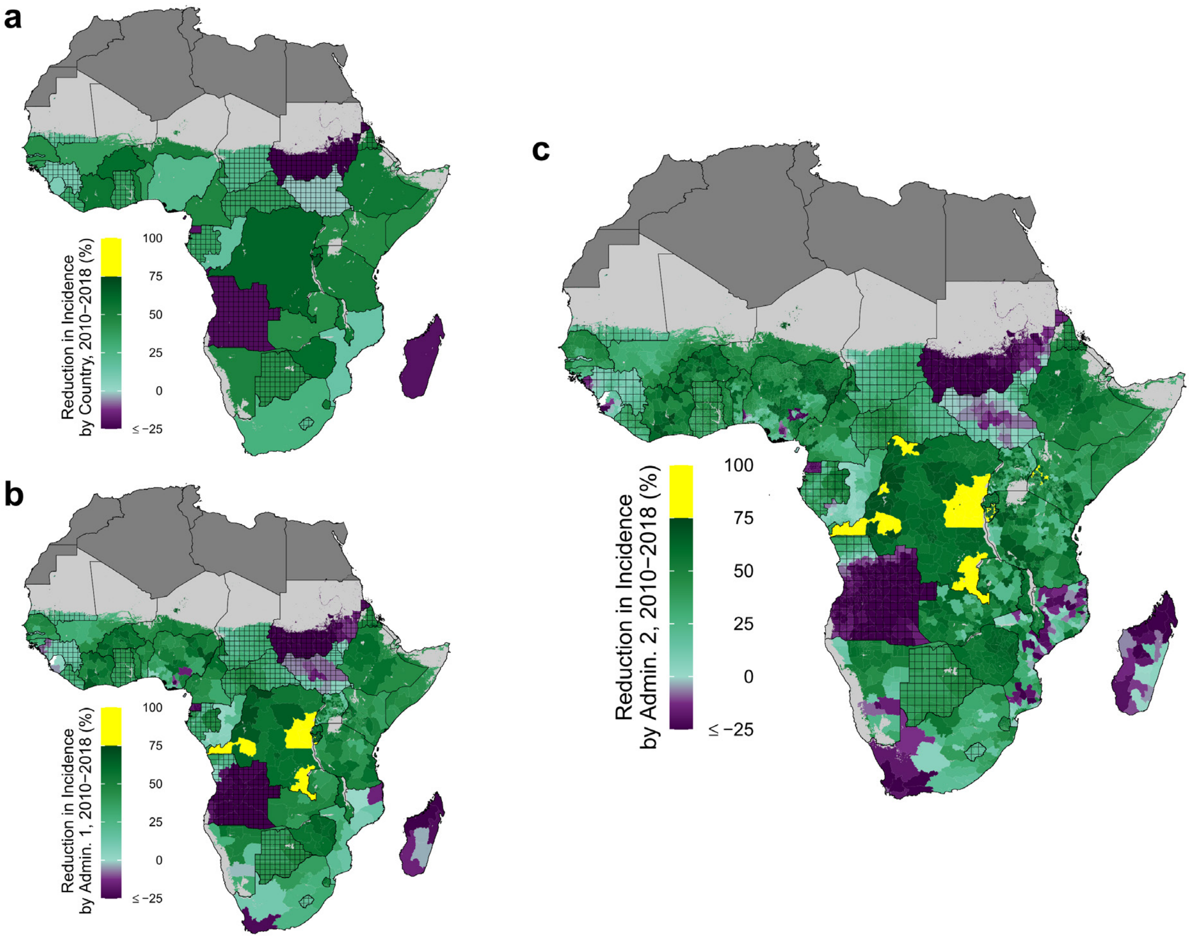

Appendix Figure 10. Percent reduction in HIV incidence rate from 2010 to 2018. Reduction in the HIV incidence rate (%) between 2010 and 2018 among individuals aged 15–49 by country (**a**), first-level administrative unit (**b**), and second-level administrative unit (**c**). Lakes and areas with fewer than ten people per 1 × 1 km and classified as barren or sparsely vegetated are colored light grey.[12,13,24–27] Areas in dark grey were not included in the analysis. Estimates in areas that are hatched are based on national, rather than subnational, ART coverage estimates only (see methods).

Appendix Figure 11: Percent reduction in the HIV mortality rate from 2010 to 2018

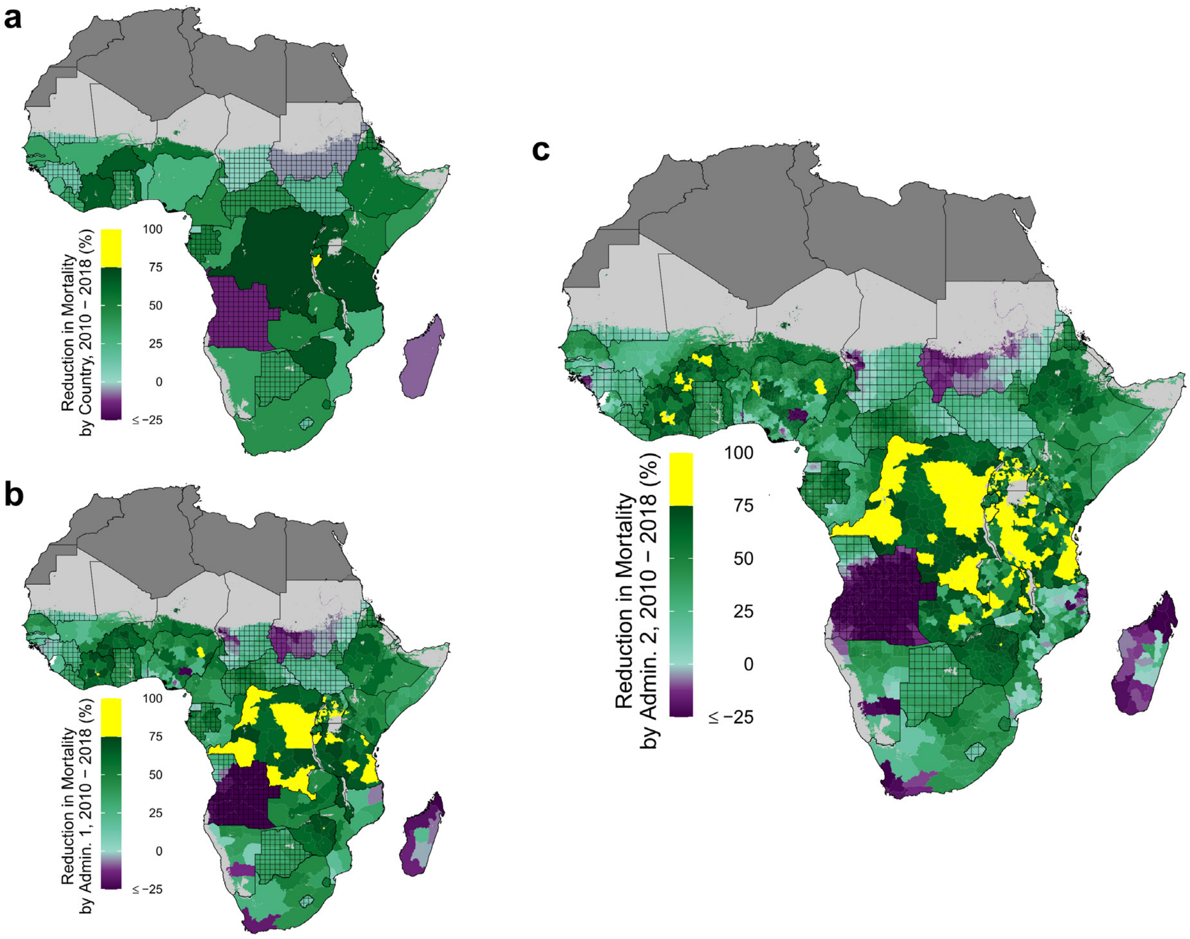

Appendix Figure 11. Percent reduction in HIV mortality rate from 2010 to 2018. Reduction in the HIV mortality rate (%) between 2010 and 2018 among individuals aged 15–49 by country (**a**), first-level administrative unit (**b**), and second-level administrative unit (**c**). Lakes and areas with fewer than ten people per 1 × 1 km and classified as barren or sparsely vegetated are colored light grey.[12,13,24–27] Areas in dark grey were not included in the analysis. Estimates in areas that are hatched are based on national, rather than subnational, ART coverage estimates only (see methods).

Appendix Figure 12: Posterior probability of a 75% or greater reduction in incident HIV cases from 2010 to 2018

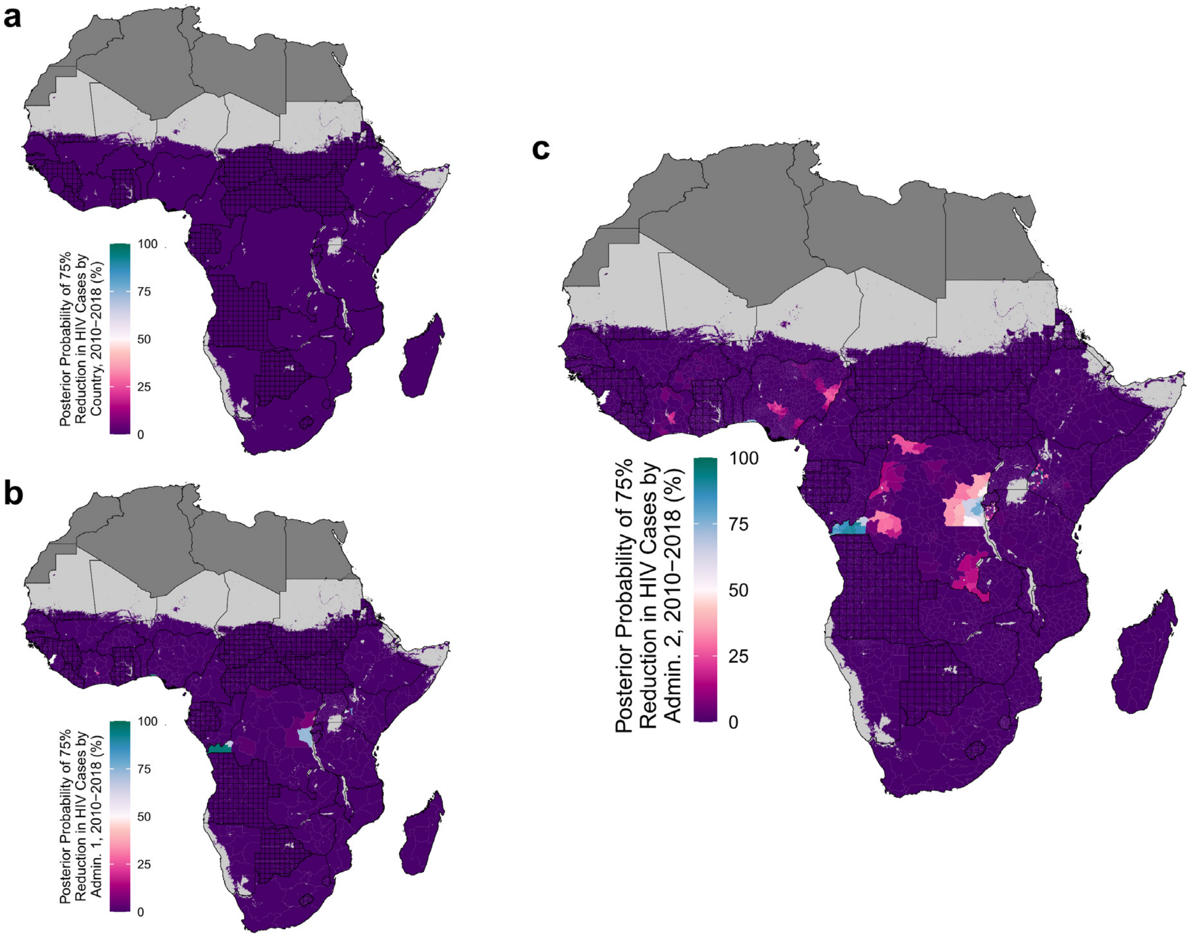

Appendix Figure 12. Posterior probability of a 75% or greater reduction in incident HIV cases from 2010 to 2018. The percent of model draws which met the UNAIDS target of a 75% reduction in incident HIV cases between 2010 and 2018 by country (**a**), first-level administrative unit (**b**), and second-level administrative unit (**c**). High posterior probability indicates a high degree of confidence that the reduction in new infections was at least 75%, while low posterior probability indicates a high degree of confidence that the reduction in incidence cases was less than 75%. Lakes and areas with fewer than ten people per 1 × 1 km and classified as barren or sparsely vegetated are colored light grey.[12,13,24–27] Areas in dark grey were not included in the analysis. Estimates in areas that are hatched are based on national, rather than subnational, ART coverage estimates only (see methods).

Appendix Figure 13: Posterior probability of an increase in incident HIV cases from 2010 to 2018

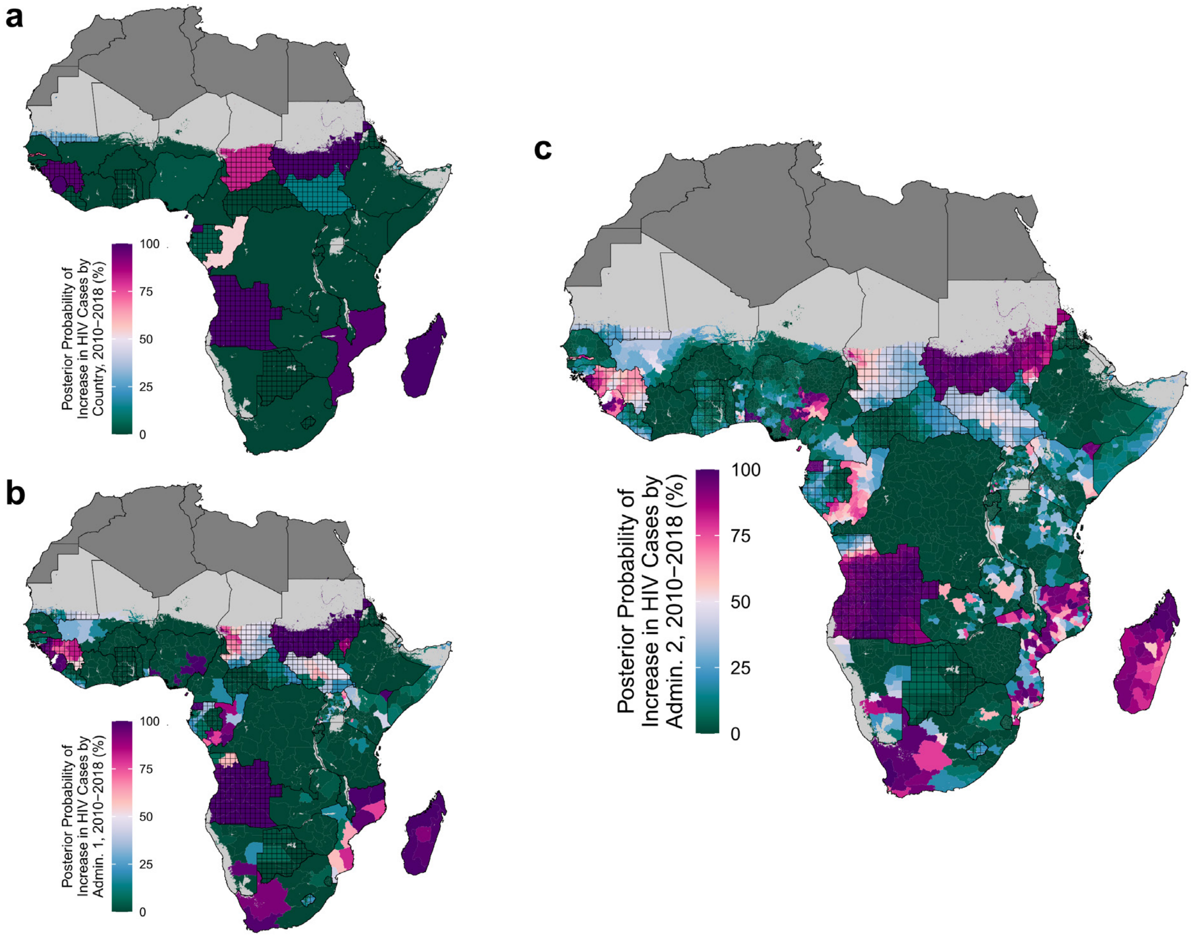

Appendix Figure 13. Posterior probability of an increase in incident HIV cases from 2010 to 2018. The percent of model draws which showed an increase in incident HIV cases between 2010 and 2018 by country (**a**), first-level administrative unit (**b**), and second-level administrative unit (**c**). High posterior probability indicates a high degree of confidence that the number of incidence cases increased, while low posterior probability indicates a high degree of confidence that the number of new infections declined. Lakes and areas with fewer than ten people per 1 × 1 km and classified as barren or sparsely vegetated are colored light grey.[12,13,24–27] Areas in dark grey were not included in the analysis. Estimates in areas that are hatched are based on national, rather than subnational, ART coverage estimates only (see methods).

Appendix Figure 14: Posterior probability of a 75% or greater reduction in HIV deaths from 2010 to 2018

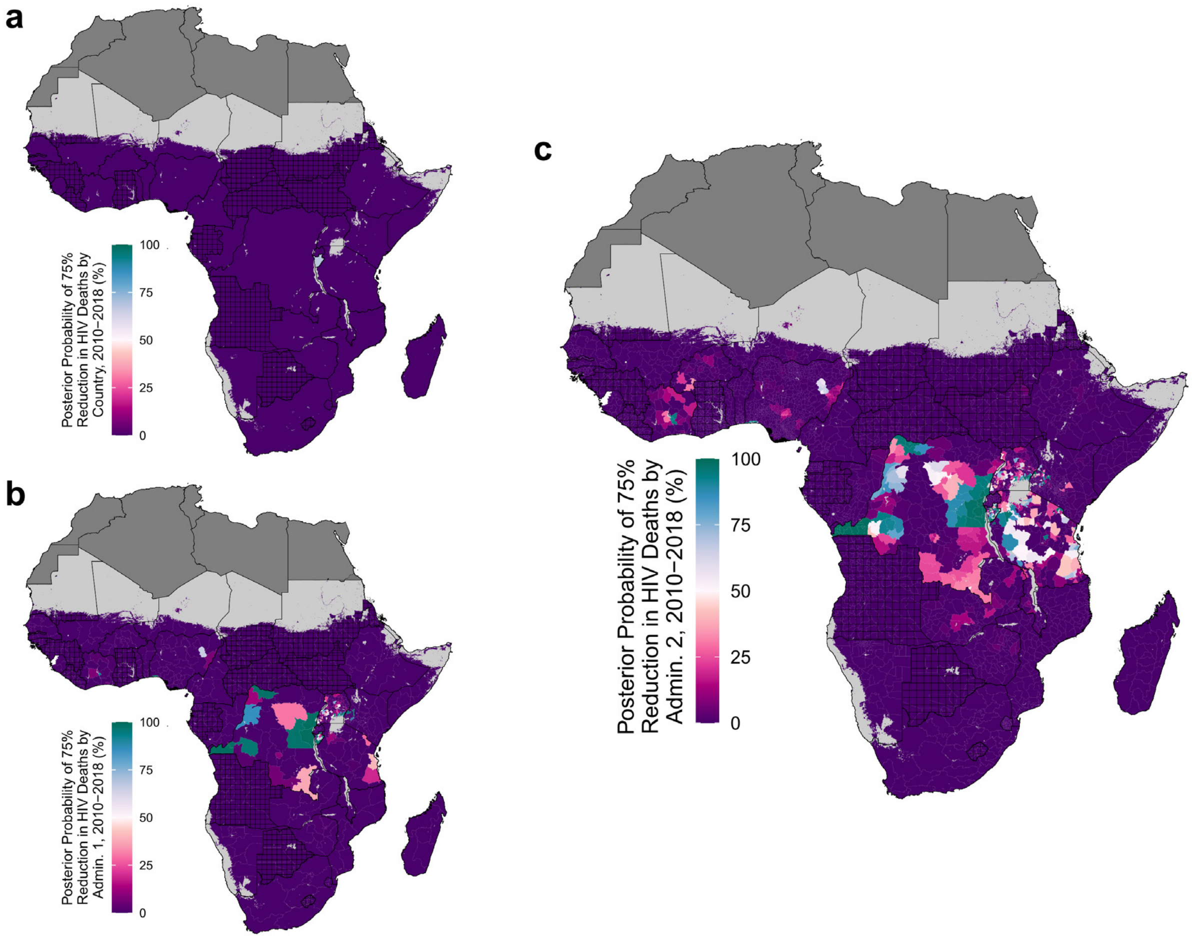

Appendix Figure 14. Posterior probability of a 75% or greater reduction in HIV deaths from 2010 to 2018. The percent of model draws which met the UNAIDS target of a 75% reduction in HIV deaths between 2010 and 2018 by country (**a**), first-level administrative unit (**b**), and second-level administrative unit (**c**). High posterior probability indicates a high degree of confidence that the reduction in deaths was at least 75%, while low posterior probability indicates a high degree of confidence that the reduction in deaths was less than 75%. Lakes and areas with fewer than ten people per 1 × 1 km and classified as barren or sparsely vegetated are colored light grey.[12,13,24–27] Areas in dark grey were not included in the analysis. Estimates in areas that are hatched are based on national, rather than subnational, ART coverage estimates only (see methods).

Appendix Figure 15: Posterior probability of an increase in HIV deaths from 2010 to 2018

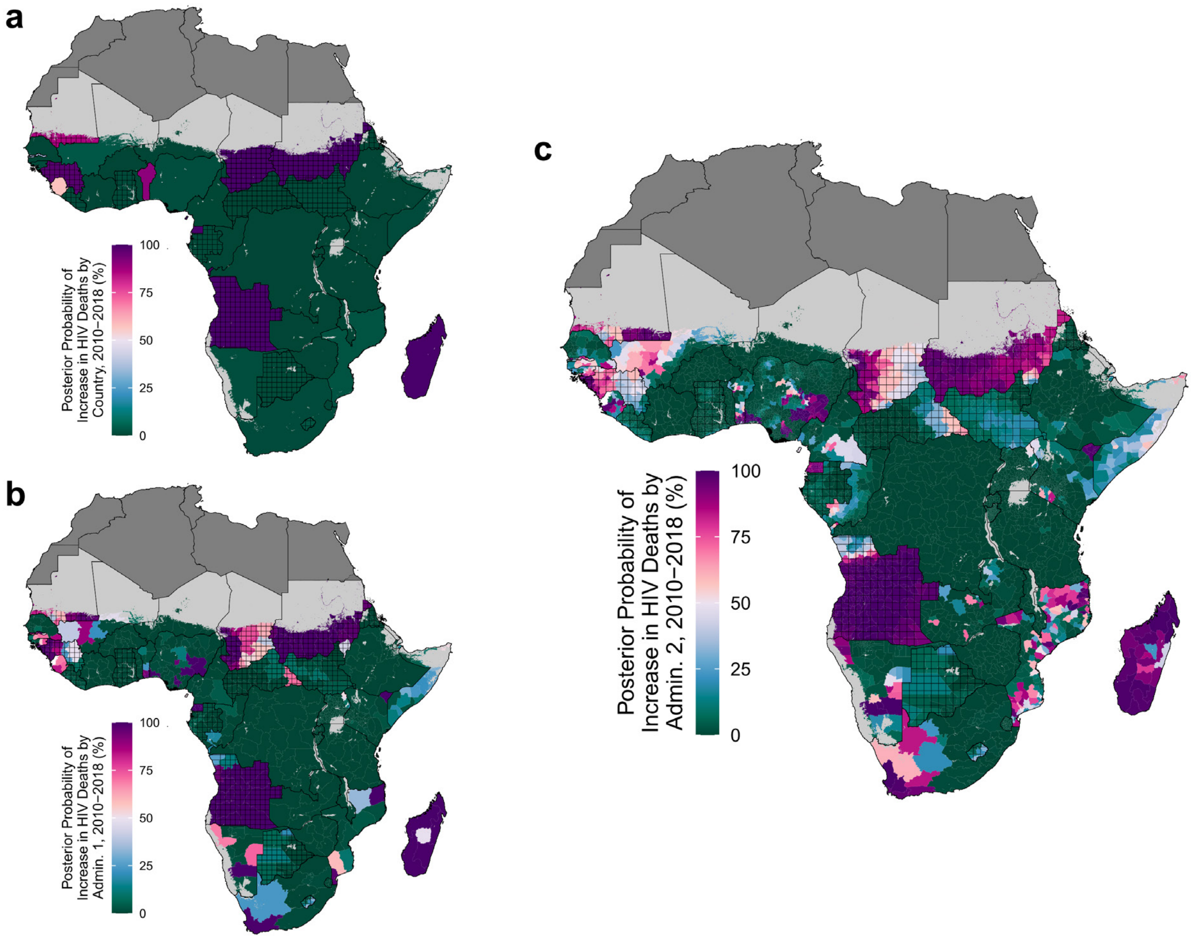

Appendix Figure 15. Posterior probability of an increase in HIV deaths from 2010 to 2018. The percent of model draws which showed an increase in HIV deaths between 2010 and 2018 by country (**a**), first-level administrative unit (**b**), and second-level administrative unit (**c**). High posterior probability indicates a high degree of confidence that the number of deaths increased, while low posterior probability indicates a high degree of confidence that the number of deaths declined. Lakes and areas with fewer than ten people per 1 × 1 km and classified as barren or sparsely vegetated are colored light grey.[12,13,24–27] Areas in dark grey were not included in the analysis. Estimates in areas that are hatched are based on national, rather than subnational, ART coverage estimates only (see methods).

Appendix Figure 16: Posterior probability that the ratio of incident HIV cases to people living with HIV was less than 0.03 in 2018

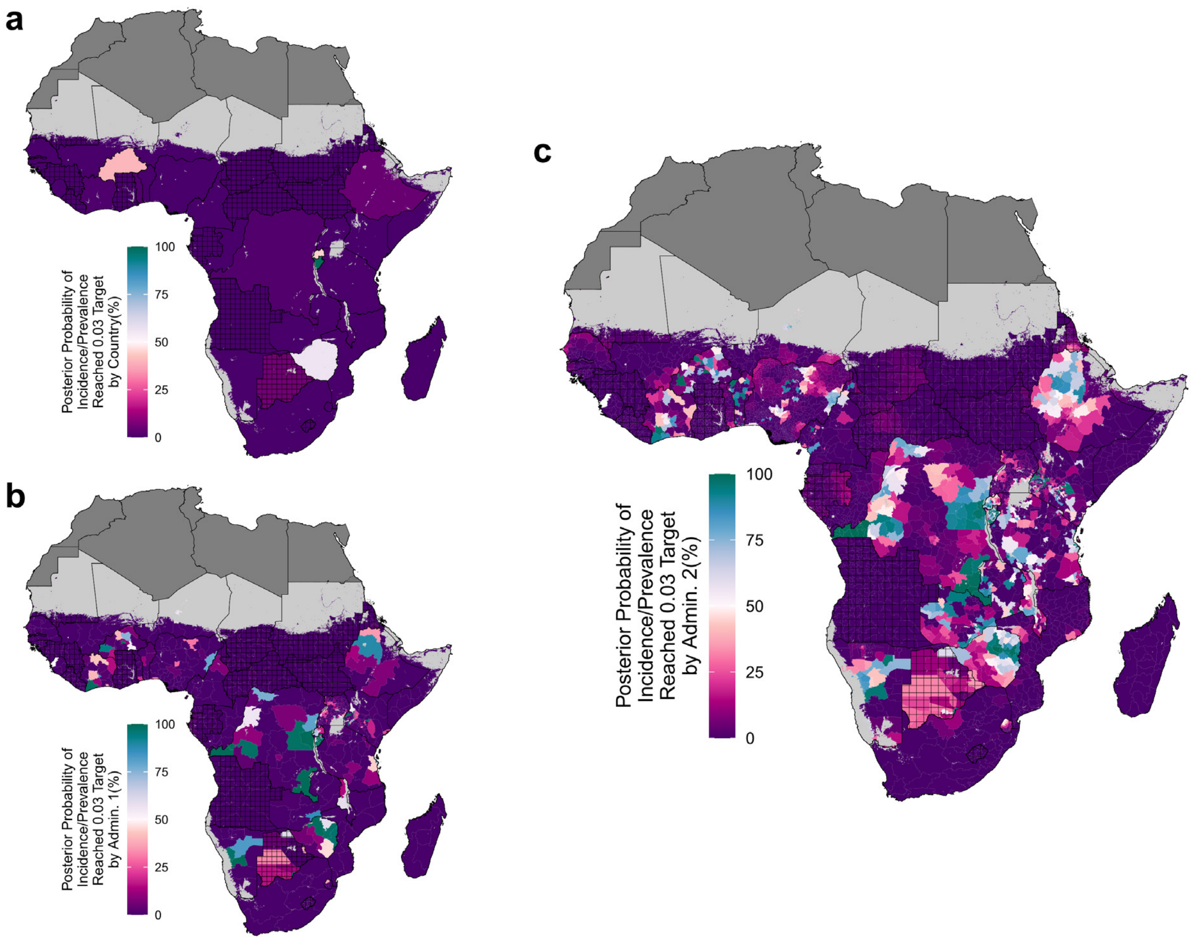

Appendix Figure 16. Posterior probability that the ratio of incident HIV cases to people living with HIV was less than 0.03 in 2018. The percent of model draws which met the goal of an incidence to prevalence ratio of less than 0.03 in 2018 by country (**a**), first-level administrative unit (**b**), and second-level administrative unit (**c**). High posterior probability indicates a high degree of confidence that the incidence to prevalence ratio was less than 0.03 while low posterior probability indicates a high degree of confidence that the incidence to prevalence ratio was greater than 0.03. Lakes and areas with fewer than ten people per 1 × 1 km and classified as barren or sparsely vegetated are colored light grey.[12,13,24–27] Areas in dark grey were not included in the analysis. Estimates in areas that are hatched are based on national, rather than subnational, ART coverage estimates only (see methods).

Appendix Tables

Appendix Table 1a: Impact of down sampling HIV mortality and CD4 progression parameters on mean estimate variability.

	Correlation		Mean Absolute Difference		Median Absolute Difference		90th Percentile of the Absolute Difference		Max Absolute Difference	
	1000 to 1000*	1000 to 100*	1000 to 1000*	1000 to 100*	1000 to 1000*	1000 to 100*	1000 to 1000*	1000 to 100*	1000 to 1000*	1000 to 100*
Incidence (cases / 100k pop)	0.999	0.999	5.15	5.83	2.26	3.11	14.02	15.07	58.33	90.24
Mortality (deaths / 100k pop)	0.999	0.999	1.90	2.98	1.16	1.87	4.72	7.07	20.74	50.07
Prevalence (% of population)	0.999	0.999	0.030	0.032	0.017	0.020	0.077	0.077	0.277	0.312

*Number of simulations being compared.

Appendix Table 1b: Impact of down sampling HIV mortality and CD4 progression parameters on uncertainty interval width variability.

	Correlation		Mean Absolute Difference		Median Absolute Difference		90th Percentile of the Absolute Difference		Max Absolute Difference	
	1000 to 1000*	1000 to 100*	1000 to 1000*	1000 to 100*	1000 to 1000*	1000 to 100*	1000 to 1000*	1000 to 100*	1000 to 1000*	1000 to 100*
Incidence (cases / 100k pop)	0.997	0.997	23.19	25.01	10.67	11.98	60.68	64.95	425.54	397.40
Mortality (deaths / 100k pop)	0.996	0.995	9.78	15.06	5.78	8.26	23.36	38.69	136.92	124.39
Prevalence (% of population)	0.996	0.996	0.121	0.121	0.072	0.073	0.291	0.295	1.313	1.265

*Number of simulations being compared.

Appendix Table 2: Edits to GADM shapes

Country Name	Action Taken
Benin	Split a second-level administrative unit into 2 pieces to better align with administrative data.
Burundi	Removed GADM second-level administrative units which represented only water in lakes. The area of the lakes was merged to adjacent land-based administrative units while avoiding changing the boundaries of first-level administrative units
Central African Republic	Replaced the first- and second-level administrative units with those published by the Malaria Atlas Project[28] because they better match published counts of subnational units.
Democratic Republic of the Congo	Edited second-level administrative unit locations to better align with population surfaces and satellite imagery.
Eritrea	Replaced the first- and second-level administrative units with those published by the Malaria Atlas Project[28] because they better match published counts of subnational units.
Gabon	Replaced the first- and second-level administrative units with those published by the Malaria Atlas Project[28] because they better match published counts of subnational units.
Ghana	Replaced the first- and second-level administrative units with those published by the Malaria Atlas Project[28] because they better match published counts of subnational units.
Kenya	Overlapping second-level administrative units identified and corrected, selected sub-districts remerged to better align with in-country district boundaries.
Malawi	Realigned the GADM definitions for first- and second-level administrative units to match in country documentation. Also edited the Likoma boundary to better represent the islands.
Mozambique	Corrected naming errors in GADM data
Niger	Replaced the first- and second-level administrative units with those published by the Malaria Atlas Project[28] because they better match published counts of subnational units.
Republic of the Congo	Replaced the first- and second-level administrative units with those published by the Malaria Atlas Project[28] because they better match published counts of subnational units.
Togo	Replaced the first- and second-level administrative units with those published by the Malaria Atlas Project[28] because they better match published counts of subnational units.
Uganda	Replaced the first- and second-level administrative unit boundaries with shapes verified by in-country collaborators.
United Republic of Tanzania	Removed GADM second-level administrative units which represented only water in lakes. The area of the lakes was merged to adjacent land-based administrative units while avoiding changing the boundaries of first-level administrative units
Zambia	Replaced the first- and second-level administrative units with those published by the Malaria Atlas Project[28] because they better match published counts of subnational units.

Appendix Table 3: Geographic administrative units

Country	Admin. 1 Count	Admin. 2 Count	Admin. 1 Name	Admin. 2 Name
Angola	18	163	Provinces	Municipalities
Benin	12	77	Departments	Communes
Botswana	16	30	Districts	Sub-districts
Burkina Faso	13	45	Regions	Provinces
Burundi	17	129	Provinces	Municipalities
Cameroon	10	58	Regions	Departments
Central African Republic	17	71	Prefectures	Sub-prefectures
Chad	23	53	Regions	Departments
Côte d'Ivoire	14	33	Districts	Territories
Democratic Republic of the Congo	26	239	Provinces	Territories
Djibouti	5	11	Regions	Communes
Equatorial Guinea	7	32	Provinces	Districts
Eritrea	6	58	Regions	Subregions
Eswatini	4	55	Regions	Tinkhundla
Ethiopia	11	78	Regions	Zones
Gabon	9	48	Provinces	Departments
Gambia	6	37	Regions	Districts
Ghana	10	216	Regions	Districts
Guinea	8	34	Regions	Prefectures
Guinea-Bissau	9	37	Regions	Sectors
Kenya	47	290	Counties	Sub-counties
Lesotho	10	10	Districts	Constituencies
Liberia	15	65	Counties	Districts
Madagascar	6	22	Provinces	Regions
Malawi	3	28	Districts	Constituencies
Mali	9	50	Regions	Cercles
Mauritania	13	44	Regions	Departments
Mozambique	11	129	Provinces	Districts
Namibia	13	107	Regions	Constituencies
Niger	8	64	Regions	Departments
Nigeria	37	769	States	Local Government Areas
Republic of Congo	12	89	Departments	Districts
Rwanda	5	30	Provinces	Districts
Senegal	14	45	Regions	Departments
Sierra Leone	4	14	Provinces	Districts
Somalia	18	74	Regions	Districts
South Africa	9	52	Provinces	Districts

Country	Admin. 1 Count	Admin. 2 Count	Admin. 1 Name	Admin. 2 Name
South Sudan	10	45	States	Counties
Sudan	18	79	States	Districts
Togo	5	30	Regions	Prefectures
Uganda	128	200	Districts	Counties
United Republic of Tanzania	30	169	Regions	Districts
Zambia	10	103	Provinces	Districts
Zimbabwe	10	60	Provinces	Districts

Appendix Table 4: HIV seroprevalence survey data

Country	Years	Name	Type	Geographic Detail	Citation	NID*
Angola	2015-16	Angola Demographic and Health Survey 2015-2016	Survey microdata	GPS	ICF International, Ministry of Health (Angola), National Institute of Statistics (Angola), United Nations Children's Fund (UNICEF). Angola Demographic and Health Survey 2015-2016. Fairfax, United States of America: ICF International, 2017.	218555
Benin	2006	Benin Demographic and Health Survey 2006	Survey report	Admin 1	Macro International, Inc, National Institute of Statistics and Economic Analysis (INSAE) (Benin), National Program Against AIDS (PNLS) (Benin). Benin Demographic and Health Survey 2006. Fairfax, United States of America: ICF International.	18959
Benin	2012	Benin Demographic and Health Survey 2011-2012	Survey report	Admin 1	ICF International, National Institute of Statistics and Economic Analysis (INSAE) (Benin), National Program Against AIDS (PNLS) (Benin). Benin Demographic and Health Survey 2011-2012. Fairfax, United States of America: ICF International, 2014.	79839
Benin, Cameroon, Kenya, Zambia	1997	Multicentre study on factors determining differences in rate of spread of HIV in sub Saharan Africa: methods and prevalence of HIV infection	Literature review	Admin 2	Buvé A, Caraël M, Hayes RJ, Auvert B, Ferry B, Robinson NJ, Anagonou S, Kanhonou L, Laourou M, Abega S, Akam E, Zekeng L, Chege J, Kahindo M, Rutenberg N, Kaona F, Musonda R, Sukwa T, Morison L, Weiss HA, Laga M. Multicentre study on factors determining differences in rate of spread of HIV in sub Saharan Africa: methods and prevalence of HIV infection. AIDS. 2001; 15: S5-14.	325201
Botswana	2004	Botswana AIDS Impact Survey 2004	Survey microdata	GPS	Central Statistics Office (Botswana). Botswana AIDS Impact Survey 2004.	22114

Country	Years	Name	Type	Geographic Detail	Citation	NID*
					Gaborone, Botswana: Statistics Botswana.	
Botswana	2008	Botswana AIDS Impact Survey 2008	Survey microdata	GPS	Central Statistics Office (Botswana), National AIDS Coordinating Agency (Botswana). Botswana AIDS Impact Survey 2008. Gaborone, Botswana: Statistics Botswana, 2015.	22116
Botswana	2013	Botswana AIDS Impact Survey 2013	Survey microdata	GPS	Ministry of Health (Botswana), National AIDS Coordinating Agency (Botswana), Statistics Botswana. Botswana AIDS Impact Survey 2013. Gaborone, Botswana: Statistics Botswana, 2015.	134753
Burkina Faso	2000	Epidemiology of HIV infection in urban Burkina Faso	Literature review	Admin 3	Lagarde E, Congo Z, Meda N, Baya B, Yaro S, Sangli G, Traoré Y, Van Renthergem H, Caraël M, Study Group on HIV Dynamic Among Young Adults in Burkina Faso. Epidemiology of HIV infection in urban Burkina Faso. Int J STD AIDS. 2004; 15(6): 395-402.	127572
Burkina Faso	2003	Burkina Faso Demographic and Health Survey 2003	Survey microdata	GPS	Macro International, Inc, National Institute of Statistics and Demography (Burkina Faso). Burkina Faso Demographic and Health Survey 2003. Fairfax, United States of America: ICF International.	19088
Burkina Faso	2010	Burkina Faso Demographic and Health Survey 2010-2011	Survey microdata	GPS	ICF Macro, Ministry of Health (Burkina Faso), National Institute of Statistics and Demography (Burkina Faso). Burkina Faso Demographic and Health Survey 2010-2011. Fairfax, United States of America: ICF International.	19133
Burundi	2002	Burundi National HIV Infection Seroprevalence Survey 2001	Survey report	Admin 1	Training Center for Medical and Infectious Diseases (CEFORMI) (Burundi). Burundi National HIV Infection Seroprevalence Survey 2001.	150378

Country	Years	Name	Type	Geographic Detail	Citation	NID*
Burundi	2007	Burundi Combined HIV/AIDS/STI Behavioral Surveillance and HIV/AIDS Seroprevalence Survey 2007	Survey report	Admin 1	National Council for the Fight Against AIDS (CNLS) (Burundi). Burundi Combined HIV/AIDS/STI Behavioral Surveillance and HIV/AIDS Seroprevalence Survey 2007.	335814
Burundi	2010-11	Burundi Demographic and Health Survey 2010-2011	Survey microdata	GPS	Burundi Institute of Statistics and Economic Studies, ICF International, Ministry of Public Health and the Fight Against AIDS (Burundi). Burundi Demographic and Health Survey 2010-2011. Fairfax, United States of America: ICF International, 2012.	30431
Burundi	2016-17	Burundi Demographic and Health Survey 2016-2017	Survey microdata	GPS	Burundi Institute of Statistics and Economic Studies, ICF International, Ministry of Public Health and the Fight Against AIDS (Burundi). Burundi Demographic and Health Survey 2016-2017. Fairfax, United States of America: ICF International, 2018.	286766
Cameroon	2004	Cameroon Demographic and Health Survey 2004	Survey microdata	GPS	Macro International, Inc, National Institute of Statistics (Cameroon). Cameroon Demographic and Health Survey 2004. Fairfax, United States of America: ICF International.	19211
Cameroon	2011	Cameroon Demographic and Health Survey 2011	Survey microdata	GPS	ICF International, Ministry of Economy, Planning and Regional Development (Cameroon), Ministry of Public Health (Cameroon), National Institute of Statistics (Cameroon), Pasteur Center of Cameroon. Cameroon Demographic and Health Survey 2011. Fairfax, United States of America: ICF International.	19274
Cameroon	2017	Cameroon Population-	Survey report	Admin >2	Ministry of Health, Cameroon, Centers for	358376

Country	Years	Name	Type	Geographic Detail	Citation	NID*
		based HIV Impact Assessment 2017-2018			Disease Control and Prevention (CDC), and ICAP at Columbia University. Cameroon Population-based HIV Impact Assessment (CAMPHIA) 2017-18: Summary Sheet. Yaoundé, Cameroon, Atlanta, Georgia and New York, New York, USA: Ministry of Health, CDC and ICAP. July 2018.	
Cape Verde	2005	Cape Verde Demographic and Health Survey 2005	Survey report	Admin 1	Macro International, Inc, Ministry of Health (Cape Verde), National Institute of Statistics (Cape Verde). Cape Verde Demographic and Health Survey 2005.	21442
Central African Republic	2006	Central African Republic Multiple Indicator Cluster Survey 2006	Survey report	Admin 1	United Nations Children's Fund (UNICEF). Central African Republic Multiple Indicator Cluster Survey 2006. New York, United States: United Nations Children's Fund (UNICEF).	2223
Central African Republic	2010	Central African Republic Multiple Indicator Cluster Survey 2010-2011	Survey report	Admin 1	Central African Institute of Statistics, Economic and Social Studies (ICASEES) (Central African Republic), ICF International. Central African Republic Multiple Indicator Cluster Survey 2010-2011. Fairfax, United States of America: ICF International, 2013.	82832
Chad	2005	Chad National HIV/AIDS Seroprevalence Survey 2005	Survey report	Admin >1	Conseil Santé (France), National Institute for Statistics, Economic and Demographic Studies (INSEED) (Chad). Chad National HIV/AIDS Seroprevalence Survey 2005.	2248
Chad	2014-15	Chad Demographic and Health Survey 2014-2015	Survey microdata	GPS	ICF International, National Institute for Statistics, Economic and Demographic Studies (INSEED) (Chad). Chad Demographic and Health Survey 2014-2015. Fairfax, United States of America: ICF International, 2016.	157025

Country	Years	Name	Type	Geographic Detail	Citation	NID*
Congo	2009	Congo AIDS Indicator Survey 2009	Survey microdata	Admin 1	ICF Macro, National Center for Statistics and Economic Studies (Congo, Rep.). Congo AIDS Indicator Survey 2009. Fairfax, United States of America: ICF International.	3133
Congo, Niger	2002-03	HIV and AIDS Surveillance Database, Version July 2017	Survey report	Admin 1	United States Census Bureau. HIV and AIDS Surveillance Database, Version July 2017. Washington, D.C., United States: United States Census Bureau, 2017.	313360
Côte d'Ivoire	2005	Côte d'Ivoire AIDS Indicator Survey 2005	Survey microdata	Admin >1	CDC Retro-CI, Ministry of the Fight Against AIDS (Côte d'Ivoire), National Institute of Statistics (Côte d'Ivoire), ORC Macro. Côte d'Ivoire AIDS Indicator Survey 2005. Fairfax, United States of America: ICF International.	56148
Côte d'Ivoire	2011-12	Côte d'Ivoire Demographic and Health Survey 2011-2012	Survey microdata	GPS	ICF International, Ministry of the Fight Against AIDS (Côte d'Ivoire), National Institute of Statistics (Côte d'Ivoire). Côte d'Ivoire Demographic and Health Survey 2011-2012. Fairfax, United States of America: ICF International.	18533
Côte d'Ivoire	2017	Côte d'Ivoire Population-based HIV Impact Assessment 2017-2018	Survey report	Admin 1	Ministry of Health and Public Hygiene, Côte d'Ivoire, Centers for Disease Control and Prevention (CDC), and ICAP at Columbia University. Côte d'Ivoire Population-based HIV Impact Assessment (CIPHIA) 2017-18: Summary Sheet. Abidjan, Côte d'Ivoire, Atlanta, Georgia and New York, New York, USA: Ministry of Health, CDC and ICAP. July 2018.	365441
Democratic Republic of the Congo	2007	Democratic Republic of the Congo Demographic and Health Survey 2007	Survey microdata	GPS	Macro International, Inc, Ministry of Planning (Congo, DR). Democratic Republic of the Congo Demographic and Health Survey 2007. Fairfax,	19381

Country	Years	Name	Type	Geographic Detail	Citation	NID*
					United States of America: ICF International.	
Democratic Republic of the Congo	2013-14	Democratic Republic of the Congo Demographic and Health Survey 2013-2014	Survey microdata	GPS	ICF International, Ministry of Planning and Monitoring Implementation of the Revolution of Modernity (Congo, DR), Ministry of Public Health (Congo, DR), National Institute of Statistics (Congo, DR). Democratic Republic of the Congo Demographic and Health Survey 2013-2014. Fairfax, United States of America: ICF International, 2014.	76878
Equatorial Guinea	2011	Equatorial Guinea Demographic and Health Survey 2011	Survey report	Admin 1	ICF International, Ministry of Health and Social Welfare (Equatorial Guinea), Ministry of Planning, Economic Development and Public Investment (Equatorial Guinea). Equatorial Guinea Demographic and Health Survey 2011. Fairfax, United States of America: ICF International, 2012.	76884
Eritrea	2010	Eritrea Population and Health Survey 2010	Survey report	Admin 1	Kenya Medical Research Institute (KEMRI), National Statistics Office (Eritrea), The Fafo Research Foundation. Eritrea Population and Health Survey 2010.	249999
Eswatini	2006-07	Swaziland Demographic and Health Survey 2006-2007	Survey microdata	GPS	Central Statistical Office (Swaziland), Macro International, Inc. Swaziland Demographic and Health Survey 2006-2007. Fairfax, United States of America: ICF International.	20829
Eswatini	2011	Swaziland HIV Incidence Measurement Survey (SHIMS): Descriptive Data Tables, August 2014	Survey report	Admin 1	Centers for Disease Control and Prevention (CDC), ICAP, Columbia University Mailman School of Public Health, Ministry of Health (Swaziland). Swaziland HIV Incidence Measurement Survey (SHIMS): Descriptive Data Tables, August 2014. New York, New York: ICAP,	334391

Country	Years	Name	Type	Geographic Detail	Citation	NID*
					Columbia University Mailman School of Public Health, 2014.	
Eswatini	2017	Swaziland HIV Incidence Measurement Survey 2 2016-2017	Survey microdata	Admin 1	Government of the Kingdom of Eswatini. Swaziland HIV Incidence Measurement Survey 2 (SHIMS2) 2016-2017. Final Report. Mbabane: Government of the Kingdom of Eswatini; April 2019.	415531
Ethiopia	2005	Ethiopia Demographic and Health Survey 2005	Survey microdata	GPS	Macro International, Inc, Population and Housing Census Commissions Office (PHCCO). Ethiopia Demographic and Health Survey 2005. Fairfax, United States of America: ICF International.	19557
Ethiopia	2011-12	Ethiopia Demographic and Health Survey 2010-2011	Survey microdata	GPS	Central Statistical Agency (Ethiopia), ICF Macro, Ministry of Health (Ethiopia). Ethiopia Demographic and Health Survey 2010-2011. Fairfax, United States of America: ICF International.	21301
Ethiopia	2016	Ethiopia Demographic and Health Survey 2016	Survey microdata	GPS	Central Statistical Agency (Ethiopia), ICF International. Ethiopia Demographic and Health Survey 2016. Fairfax, United States of America: ICF International, 2017.	218568
Gabon	2012	Gabon Demographic and Health Survey 2012	Survey microdata	GPS	General Directorate of Statistics (Gabon), ICF International, Ministry of Economy, Employment and Sustainable Development (Gabon), Ministry of Health (Gabon). Gabon Demographic and Health Survey 2012. Fairfax, United States of America: ICF International, 2013.	76706
Gambia	2013	Gambia Demographic and Health Survey 2013	Survey microdata	Admin 2	Gambia Bureau of Statistics (GBOS), ICF International, Ministry of Health and Social Welfare (The Gambia). Gambia Demographic and Health Survey 2013. Fairfax,	77384

Country	Years	Name	Type	Geographic Detail	Citation	NID*
					United States of America: ICF International, 2015.	
Ghana	2003	Ghana Demographic and Health Survey 2003	Survey microdata	GPS	Ghana Statistical Service, Macro International, Inc. Ghana Demographic and Health Survey 2003. Fairfax, United States of America: ICF International.	19627
Ghana	2014	Ghana Demographic and Health Survey 2014	Survey microdata	GPS	Ghana Health Service, Ghana Statistical Service, ICF International. Ghana Demographic and Health Survey 2014. Fairfax, United States of America: ICF International, 2016.	157027
Guinea	2005	Guinea Demographic and Health Survey 2005	Survey microdata	GPS	Macro International, Inc, National Statistics Directorate (Guinea). Guinea Demographic and Health Survey 2005. Fairfax, United States of America: ICF International.	19683
Guinea	2012	Guinea Demographic and Health Survey 2012	Survey microdata	GPS	ICF Macro, Ministry of Health and Public Hygiene (Guinea), National Institute of Statistics (Guinea). Guinea Demographic and Health Survey 2012. Fairfax, United States of America: ICF International.	69761
Guinea	2018	Guinea Demographic and Health Survey 2018	Survey microdata	GPS	Ministry of Health (Guinea), Ministry of Planning and Economic Development (Guinea), National Institute of Statistics (Guinea). Guinea Demographic and Health Survey 2018. Fairfax, United States of America: ICF International, 2019.	396957
Guinea-Bissau	1996-05	Changes in prevalence and incidence of HIV-1, HIV-2 and dual infections in urban areas of Bissau, Guinea-Bissau: is HIV-2 disappearing?	Literature review	Site	da Silva ZJ, Oliveira I, Andersen A, Dias F, Rodrigues A, Holmgren B, Andersson S, Aaby P. Changes in prevalence and incidence of HIV-1, HIV-2 and dual infections in urban areas of Bissau, Guinea-Bissau: is HIV-2 disappearing? AIDS. 2008; 22(10): 1195-202.	353416

Country	Years	Name	Type	Geographic Detail	Citation	NID*
Guinea-Bissau	1997-06	Two distinct epidemics: the rise of HIV-1 and decline of HIV-2 infection between 1990 and 2007 in rural Guinea-Bissau	Literature review	Admin 2	Tienen Cv, van der Loeff MS, Zaman SM, Vincent T, Sarge-Njie R, Peterson I, Leligdowicz A, Jaye A, Rowland-Jones S, Aaby P, Whittle H. Two distinct epidemics: the rise of HIV-1 and decline of HIV-2 infection between 1990 and 2007 in rural Guinea-Bissau. J Acquir Immune Defic Syndr. 2010; 53(5): 640-7.	353418
Guinea-Bissau	2010	Guinea-Bissau HIV Prevalence Survey 2010	Survey report	Admin 1	Institute of Hygiene and Tropical Medicine, Nova University of Lisbon (Portugal), National Institute of Public Health (INASA) (Guinea-Bissau). Guinea-Bissau HIV Prevalence Survey 2010.	340936
Kenya	2003	HIV prevalence and associated risk factors among individuals aged 13-34 years in Rural Western Kenya	Literature review	City	Amornkul PN, Vandenhoudt H, Nasokho P, Odhiambo F, Mwaengo D, Hightower A, Buvé A, Misore A, Vulule J, Vitek C, Glynn J, Greenberg A, Slutsker L, De Cock KM. HIV prevalence and associated risk factors among individuals aged 13-34 years in Rural Western Kenya. PLoS One. 2009; 4(7): e6470.	222209
Kenya	2003	Kenya Demographic and Health Survey 2003	Survey microdata	GPS	Centers for Disease Control and Prevention (CDC), Central Bureau of Statistics (Kenya), Macro International, Inc, Ministry of Health (Kenya), National Council for Population and Development (Kenya). Kenya Demographic and Health Survey 2003. Fairfax, United States of America: ICF International.	20145
Kenya	2006	Association of attitudes and beliefs towards antiretroviral therapy with	Literature review	Admin 2	Cohen CR, Montandon M, Carrico AW, Shiboski S, Bostrom A, Obure A, Kwena Z, Bailey RC, Nguti R, Bukusi EA. Association of attitudes and beliefs towards	353420

Country	Years	Name	Type	Geographic Detail	Citation	NID*
		HIV-seroprevalence in the general population of Kisumu, Kenya			antiretroviral therapy with HIV-seroprevalence in the general population of Kisumu, Kenya. PLoS One. 2009; 4(3): e4573.	
Kenya	2006	The effect of participant nonresponse on HIV prevalence estimates in a population-based survey in two informal settlements in Nairobi city	Literature review	Site	Ziraba AK, Madise NJ, Matilu M, Zulu E, Kebaso J, Khamadi S, Okoth V, Ezeh AC. The effect of participant nonresponse on HIV prevalence estimates in a population-based survey in two informal settlements in Nairobi city. Popul Health Metr. 2010; 8: 22.	353424
Kenya	2007	Kenya AIDS Indicator Survey 2007	Survey microdata	GPS	Centers for Disease Control and Prevention (CDC), Kenya Medical Research Institute (KEMRI), Kenya National Bureau of Statistics, Ministry of Public Health and Sanitation (Kenya), National AIDS Control Council (Kenya), National AIDS and STI Control Programme (NASCOP) (Kenya), National Coordinating Agency for Population and Development (Kenya), National Public Health Laboratory Services, Ministry of Public Health and Sanitation (Kenya), United States Agency for International Development (USAID). Kenya AIDS Indicator Survey 2007. Nairobi, Kenya: Kenya National Bureau of Statistics.	133219
Kenya	2008-09	Kenya Demographic and Health Survey 2008-2009	Survey microdata	GPS	ICF Macro, Kenya Medical Research Institute (KEMRI), Kenya National Bureau of Statistics, Ministry of Public Health and Sanitation (Kenya), National AIDS and STI Control Programme (NASCOP) (Kenya), National	21365

Country	Years	Name	Type	Geographic Detail	Citation	NID*
					Aids Control Council (NACC), National Coordinating Agency for Population and Development (Kenya). Kenya Demographic and Health Survey 2008-2009. Fairfax, United States of America: ICF International.	
Kenya	2012	Cascade of HIV care and population viral suppression in a high-burden region of Kenya	Literature review	Town	Maman D, Zeh C, Mukui I, Kirubi B, Masson S, Opolo V, Szumilin E, Riche B, Etard JF. Cascade of HIV care and population viral suppression in a high-burden region of Kenya. AIDS. 2015; 29(12): 1557-65.	353428
Kenya	2013	Kenya AIDS Indicator Survey 2012-2013	Survey microdata	GPS	Kenya National Bureau of Statistics, Ministry of Devolution and Planning (Kenya), Ministry of Health (Kenya), National AIDS and STI Control Programme (NASCOP) (Kenya). Kenya AIDS Indicator Survey 2012-2013. Nairobi, Kenya: Kenya National Bureau of Statistics.	133304
Kenya	2018	Kenya Population-based HIV Impact Assessment 2018-2019	Survey report	Admin 1	National AIDS and STI Control Programme (NASCOP), Preliminary KENPHIA 2018 Report. Nairobi: NASCOP; 2020.	436581
Kenya, Uganda	2013	Population levels and geographical distribution of HIV RNA in rural Ugandan and Kenyan communities, including serodiscordant couples: a cross-sectional analysis	Literature review	Town	Jain V, Petersen ML, Liegler T, Byonanebye DM, Kwarisiima D, Chamie G, Sang N, Black D, Clark TD, Ladai A, Plenty A, Kabami J, Ssemmondo E, Bukusi EA, Cohen CR, Charlebois ED, Kamya MR, Havlir DV, SEARCH Collaboration. Population levels and geographical distribution of HIV RNA in rural Ugandan and Kenyan communities, including serodiscordant couples: a cross-sectional analysis. Lancet HIV. 2017; 4(3): e122-33.	332689

Country	Years	Name	Type	Geographic Detail	Citation	NID*
Lesotho	2004-05	Lesotho Demographic and Health Survey 2004-2005	Survey microdata	GPS	Bureau of Statistics (Lesotho), Macro International, Inc, Ministry of Health and Social Welfare (Lesotho). Lesotho Demographic and Health Survey 2004-2005. Fairfax, United States of America: ICF International.	20167
Lesotho	2009-10	Lesotho Demographic and Health Survey 2009-2010	Survey microdata	GPS	ICF Macro, Ministry of Health and Social Welfare (Lesotho). Lesotho Demographic and Health Survey 2009-2010. Fairfax, United States of America: ICF International.	21382
Lesotho	2014	Lesotho Demographic and Health Survey 2014	Survey microdata	GPS	ICF International, Ministry of Health and Social Welfare (Lesotho). Lesotho Demographic and Health Survey 2014. Fairfax, United States of America: ICF International.	157058
Lesotho	2017	Lesotho Population-based HIV Impact Assessment 2016-2017	Survey report	Admin 1	Ministry of Health, Lesotho, Centers for Disease Control and Prevention (CDC), and ICAP at Columbia University. Lesotho Population-based HIV Impact Assessment (LePHIA) 2016-2017: Final Report. Maseru, Lesotho, Atlanta, Georgia, and New York, New York, USA: Ministry of Health, CDC, and ICAP, September 2019.	327582
Liberia	2006-07	Liberia Demographic and Health Survey 2006-2007	Survey microdata	GPS	Liberia Institute for Statistics and Geo-information Services (LISGIS), Macro International, Inc. Liberia Demographic and Health Survey 2006-2007. Fairfax, United States of America: ICF International.	20191
Liberia	2013	Liberia Demographic and Health Survey 2013	Survey microdata	GPS	ICF International, Liberia Institute for Statistics and Geo-information Services (LISGIS), National AIDS and STI Control Program (NACP), Ministry of Health and Social Welfare (Liberia). Liberia	77385

Country	Years	Name	Type	Geographic Detail	Citation	NID*
					Demographic and Health Survey 2013. Fairfax, United States of America: ICF International.	
Malawi	2004-05	Malawi Demographic and Health Survey 2004-2005	Survey microdata	GPS	Macro International, Inc, National Statistical Office of Malawi. Malawi Demographic and Health Survey 2004-2005. Fairfax, United States of America: ICF International.	20263
Malawi	2005-09	Underestimation of HIV prevalence in surveys when some people already know their status, and ways to reduce the bias	Literature review	Site	Floyd S, Molesworth A, Dube A, Crampin AC, Houben R, Chihana M, Price A, Kayuni N, Saul J, French N, Glynn JR. Underestimation of HIV prevalence in surveys when some people already know their status, and ways to reduce the bias. AIDS. 2013; 27(2): 233-42.	353430
Malawi	2006	Sexual network structure and the spread of HIV in Africa: evidence from Likoma Island, Malawi	Literature review	Village	Helleringer S, Kohler HP. Sexual network structure and the spread of HIV in Africa: evidence from Likoma Island, Malawi. AIDS. 2007; 21(17): 2323-32.	353434
Malawi	2010	Malawi Demographic and Health Survey 2010	Survey microdata	GPS	ICF Macro, National Statistical Office of Malawi. Malawi Demographic and Health Survey 2010. Fairfax, United States of America: ICF International.	21393
Malawi	2015-16	Malawi Demographic and Health Survey 2015-2016	Survey microdata	GPS	Emory University and Centers for Disease Control & Prevention Collaboration, ICF International, Ministry of Health (Malawi), National Statistical Office of Malawi. Malawi Demographic and Health Survey 2015-2016. Fairfax, United States of America: ICF International, 2017.	218581
Malawi	2015-16	Malawi Population-based HIV Impact	Survey microdata	Admin >1	Ministry of Health, Malawi, Centers for Disease Control and Prevention (CDC), and ICAP at Columbia University.	287629

Country	Years	Name	Type	Geographic Detail	Citation	NID*
		Assessment 2015-2016			Malawi Population-based HIV Impact Assessment (MPHIA) 2015-16: First Report. Lilongwe, Malawi, Atlanta, Georgia and New York, New York, USA: Ministry of Health, CDC and ICAP. December 2016.	
Mali	2001	Mali Demographic and Health Survey 2001	Survey microdata	Admin 1, GPS	Macro International, Inc, National Directorate of Statistics and Informatics (DNSI) (Mali), Planning and Statistics Unit, Ministry of Health (Mali). Mali Demographic and Health Survey 2001. Fairfax, United States of America: ICF International.	20315
Mali	2006	Mali Demographic and Health Survey 2006	Survey microdata	GPS	Macro International, Inc, Ministry of Health (Mali), National Directorate of Statistics and Informatics (DNSI) (Mali). Mali Demographic and Health Survey 2006. Fairfax, United States of America: ICF International.	20274
Mali	2012-13	Mali Demographic and Health Survey 2012-2013	Survey microdata	GPS	ICF International, INFO-STAT (Mali), Ministry of Health (Mali), National Institute of Statistics (INSTAT) (Mali), Planning and Statistics Unit, Ministry of Health (Mali). Mali Demographic and Health Survey 2012-2013. Fairfax, United States of America: ICF International, 2014.	77388
Mozambique	2009	Mozambique AIDS Indicator Survey 2009	Survey microdata	GPS	ICF Macro, Ministry of Health (Mozambique), National Institute of Statistics (INE) (Mozambique). Mozambique AIDS Indicator Survey 2009. Fairfax, United States of America: ICF International, 2010.	8906
Mozambique	2010-12	HIV Incidence and Spatial Clustering in a	Literature review	Site	Gonzalez R, Augusto OJ, Munguambe K, Pierrat C, Pedro EN, Sacoor C, De	353436

Country	Years	Name	Type	Geographic Detail	Citation	NID*
		Rural Area of Southern Mozambique			Lazzari E, Aponte JJ, Macete E, Alonso PL, Menendez C, Naniche D. HIV Incidence and Spatial Clustering in a Rural Area of Southern Mozambique. PLoS One. 2015; 10(7): e0132053.	
Mozambique	2015	Mozambique AIDS Indicator Survey 2015	Survey microdata	GPS	Centers for Disease Control and Prevention (CDC), ICF International, Ministry of Health (Mozambique), National Institute of Health (Mozambique), National Institute of Statistics (INE) (Mozambique). Mozambique AIDS Indicator Survey 2015. Fairfax, United States of America: ICF International, 2018.	157060
Namibia	2006-08	Namibia - Khomas Windhoek Okambilimbili HIV Endline Survey 2009	Survey microdata	GPS	Aulagnier M, Janssens W, De Beer I, van Rooy G, Gaeb E, Hesp C, van der Gaag J, Rinke de Wit TF. Namibia - Khomas Windhoek Okambilimbili HIV Endline Survey 2009.	425256
Namibia	2009	Incidence of HIV in Windhoek, Namibia: demographic and socio-economic associations	Literature review	Admin 2	Aulagnier M, Janssens W, De Beer I, van Rooy G, Gaeb E, Hesp C, van der Gaag J, Rinke de Wit TF. Incidence of HIV in Windhoek, Namibia: demographic and socio-economic associations. PLoS One. 2011; 6(10).	325204
Namibia	2013	Namibia Demographic and Health Survey 2013	Survey microdata	GPS	ICF International, Ministry of Health and Social Services (Namibia), Namibia Institute of Pathology, Namibia Statistics Agency. Namibia Demographic and Health Survey 2013. Fairfax, United States of America: ICF International.	150382
Namibia	2017	Namibia Population-based HIV Impact Assessment 2017	Survey report	Admin 1	Ministry of Health and Social Services (MoHSS), Namibia. Namibia Population-based HIV Impact Assessment (NAMPHIA) 2017: Final Report. Windhoek: MoHSS, Namibia; November, 2019.	359089

Country	Years	Name	Type	Geographic Detail	Citation	NID*
Niger	2006	Niger Demographic and Health Survey 2006	Survey microdata	Admin 1	Department of Statistics and National Accounts (Niger), Macro International, Inc. Niger Demographic and Health Survey 2006. Fairfax, United States of America: ICF International.	20499
Niger	2012	Niger Demographic and Health Survey 2012	Survey microdata	Admin 1	ICF International, Ministry of Public Health (Niger), National Institute of Statistics (Niger). Niger Demographic and Health Survey 2012. Fairfax, United States of America: ICF International.	74393
Nigeria	2018	Nigeria HIV/AIDS Indicator and Impact Survey 2018	Survey report	Admin 1	Federal Ministry of Health (Nigeria), Government of Nigeria, National Agency for the Control of AIDS (NACA) (Nigeria), University of Maryland. Nigeria HIV/AIDS Indicator and Impact Survey 2018.	399235
Rwanda	2005	Rwanda Demographic and Health Survey 2005	Survey microdata	GPS	Macro International, Inc, National Institute of Statistics of Rwanda. Rwanda Demographic and Health Survey 2005. Fairfax, United States of America: ICF International.	20740
Rwanda	2010-11	Rwanda Demographic and Health Survey 2010-2011	Survey microdata	GPS	ICF Macro, Ministry of Health (Rwanda), National Institute of Statistics of Rwanda. Rwanda Demographic and Health Survey 2010-2011. Fairfax, United States of America: ICF International.	56040
Rwanda	2013	Rwanda AIDS Indicator and HIV Incidence Survey 2013	Survey report	Admin 1	Ministry of Health (Rwanda), Rwanda Biomedical Center (RBC). Rwanda AIDS Indicator and HIV Incidence Survey 2013.	343466
Rwanda	2014-15	Rwanda Demographic and Health Survey 2014-2015	Survey microdata	GPS	ICF International, Ministry of Health (Rwanda), National Institute of Statistics of Rwanda. Rwanda Demographic and Health Survey 2014-2015. Fairfax,	157063

Country	Years	Name	Type	Geographic Detail	Citation	NID*
					United States of America: ICF International, 2016.	
Sao Tome and Principe	2008	Sao Tome and Principe Demographic and Health Survey 2008-2009	Survey microdata	Admin 2	ICF Macro, Ministry of Health (Sao Tome and Principe), National Institute of Statistics (Sao Tome and Principe). Sao Tome and Principe Demographic and Health Survey 2008-2009. Fairfax, United States of America: ICF International.	26866
Sao Tome and Principe	2014	Sao Tome and Principe Multiple Indicator Cluster Survey: Biomarker Data 2014	Survey report	Admin 2	Global Fund to Fight Aids Tuberculosis and Malaria (GFATM), ICF International, National Center for Endemic Diseases (CNE) (Sao Tome and Principe), National Institute of Statistics (Sao Tome and Principe), United Nations Children's Fund (UNICEF), United Nations Development Programme (UNDP). Sao Tome and Principe Multiple Indicator Cluster Survey: Biomarker Data 2014. Fairfax, United States: ICF International.	233903
Senegal	1997-98	Mobility and the spread of human immunodeficiency virus into rural areas of West Africa	Literature review	City	Lagarde E, Schim van der Loeff M, Enel C, Holmgren B, Dray-Spira R, Pison G, Piau JP, Delaunay V, M'Boup S, Ndoye I, Coeuret-Pellicer M, Whittle H, Aaby P, MECORA Group. Mobility and the spread of human immunodeficiency virus into rural areas of West Africa. Int J Epidemiol. 2003; 32(5): 744-52.	332686
Senegal	2005	Senegal Demographic and Health Survey 2005	Survey microdata	GPS	Ministry of Health and Prevention (Senegal), Research Center for Human Development (Senegal). Senegal Demographic and Health Survey 2005. Fairfax, United States of America: ICF International.	26855
Senegal	2010-11	Senegal Demographic and Health	Survey microdata	GPS	Center for Research in Human Development (CRDH), Cheikh Anta Diop	56063

Country	Years	Name	Type	Geographic Detail	Citation	NID*
		Survey 2010-2011			University, Hospital Aristide Le Dantec, ICF Macro, National Agency of Statistics and Demography (Senegal). Senegal Demographic and Health Survey 2010-2011. Fairfax, United States of America: ICF International.	
Senegal	2017	Senegal Continuous Demographic and Health Survey 2017	Survey microdata	Admin 1	ICF International, Ministry of Health and Social Action (Senegal), National Agency of Statistics and Demography (Senegal), Unit for the Fight Against Malnutrition (Senegal). Senegal Continuous Demographic and Health Survey 2017. Fairfax, United States of America: ICF International, 2018.	353526
Sierra Leone	2002	Sierra Leone HIV/AIDS Seroprevalence and Behavioral Risk Factor Survey 2002	Survey report	Admin >1, Admin >2	Centers for Disease Control and Prevention (CDC), Government of Sierra Leone, World Bank. Sierra Leone HIV/AIDS Seroprevalence and Behavioral Risk Factor Survey 2002.	356788
Sierra Leone	2005	Sierra Leone National Population-based HIV Seroprevalence Survey 2005	Survey report	Admin 2	Ministry of Health and Sanitation (Sierra Leone), National HIV/AIDS Secretariat (NAS) (Sierra Leone), Nimba Research and Consulting (NiRC) (Ghana), Statistics Sierra Leone. Sierra Leone National Population-Based HIV Seroprevalence Survey 2005.	325150
Sierra Leone	2008	Sierra Leone Demographic and Health Survey 2008	Survey microdata	GPS	Macro International, Inc, Statistics Sierra Leone. Sierra Leone Demographic and Health Survey 2008. Fairfax, United States of America: ICF International.	21258
Sierra Leone	2013	Sierra Leone Demographic and Health Survey 2013	Survey microdata	GPS	ICF International, Ministry of Health and Sanitation (Sierra Leone), Statistics Sierra Leone. Sierra Leone Demographic and Health Survey 2013. Fairfax, United	131467

Country	Years	Name	Type	Geographic Detail	Citation	NID*
					States of America: ICF International, 2014.	
South Africa	1998	Risk factors for HIV infection among women in Carletonville, South Africa: migration, demography and sexually transmitted diseases	Literature review	Township	Zuma K, Gouws E, Williams B, Lurie M. Risk factors for HIV infection among women in Carletonville, South Africa: migration, demography and sexually transmitted diseases. Int J STD AIDS. 2003; 14(12): 814-7.	353445
South Africa	2002	Can highly active antiretroviral therapy reduce the spread of HIV?: A study in a township of South Africa	Literature review	Admin 3	Auvert B, Males S, Puren A, Taljaard D, Carael M, Williams B. Can highly active antiretroviral therapy reduce the spread of HIV?: A study in a township of South Africa. J Acquir Immune Defic Syndr. 2004; 36(1): 613-21.	353457
South Africa	2002	South Africa HIV/AIDS Behavioral Risks, Sero-Status, and Mass Media Impact Survey 2002	Survey microdata	Admin 3	Center for AIDS Development, Research and Evaluation (CADRE) (South Africa), Department of Social Development (South Africa), Family Health International, Geospace International (South Africa), Human Sciences Research Council, Joint United Nations Program on HIV/AIDS (UNAIDS), Medical University of Southern Africa (MEDUNSA), National Agency for AIDS Research (ANRS) (France), National Health Laboratory Service (NHLS) (South Africa), South African Medical Research Council, University of Natal, Wits Health Consortium. South Africa HIV/AIDS Behavioral Risks, Sero-Status, and Mass Media Impact Survey 2002. Pretoria, South Africa: Human Sciences Research Council, 2011.	12102

Country	Years	Name	Type	Geographic Detail	Citation	NID*
South Africa	2004-16	South Africa - ACDIS Health and Demographic Surveillance System	Survey microdata	Admin <1	Africa Centre for Population Studies and Reproductive Health, INDEPTH, South African Medical Research Council, University of Kwazulu-Natal. South Africa - ACDIS Health and Demographic Surveillance System.	11780
South Africa	2005	South Africa National HIV Prevalence, Incidence, Behavior and Communication Survey 2004-2005	Survey microdata	Admin 3	Center for AIDS Development, Research and Evaluation (CADRE) (South Africa), Centers for Disease Control and Prevention (CDC), Global Clinical and Viral Laboratory (South Africa), Human Sciences Research Council, Maphume Research Services, National Institute for Communicable Diseases (South Africa). South Africa National HIV Prevalence, Incidence, Behavior and Communication Survey 2004-2005. Pretoria, South Africa: Human Sciences Research Council, 2011.	313074
South Africa	2008-09	South Africa National HIV Prevalence, Incidence, Behavior and Communication Survey 2008-2009	Survey microdata	Admin 5, GPS	Center for AIDS Development, Research and Evaluation (CADRE) (South Africa), Geospace International (South Africa), Global Clinical and Viral Laboratory (South Africa), Human Sciences Research Council, Maphume Research Services, National Institute for Communicable Diseases (South Africa), South African Medical Research Council. South Africa National HIV Prevalence, Incidence, Behavior and Communication Survey 2008-2009. Pretoria, South Africa: Human Sciences Research Council, 2014.	228102
South Africa	2010	Prevalence of HIV among	Literature review	Site	Gomez-Olive FX, Angotti N, Houle B, Klipstein-Grobusch	353443

Country	Years	Name	Type	Geographic Detail	Citation	NID*
		those 15 and older in rural South Africa			K, Kabudula C, Menken J, Williams J, Tollman S, Clark SJ. Prevalence of HIV among those 15 and older in rural South Africa. AIDS Care. 2013; 25(9): 1122-8.	
South Africa	2011-12	South Africa National HIV Prevalence, Incidence, and Behavior Survey 2011-2012	Survey microdata	Admin 5, GPS	Centers for Disease Control and Prevention (CDC), Global Clinical and Viral Laboratory (South Africa), Human Sciences Research Council, National Institute for Communicable Diseases (South Africa), South African Medical Research Council, University of Cape Town. South Africa National HIV Prevalence, Incidence, and Behavior Survey 2011-2012. Pretoria, South Africa: Human Sciences Research Council, 2016.	313076
South Africa	2012	Level of viral suppression and the cascade of HIV care in a South African semi-urban setting in 2012	Literature review	Admin 3	Jean K, Puren A, Cutler E, Singh B, Bouscaillou J, Rain-Taljaard R, Taljaard D, Gouws E, Lissouba P, Lewis DA, Peytavin G, Auvert B. Level of viral suppression and the cascade of HIV care in a South African semi-urban setting in 2012. AIDS. 2016; 30(13): 2107-16.	353455
South Africa	2013	Socio-economic and demographic factors related to HIV status in urban informal settlements in the Eastern Cape, South Africa	Literature review	Township	Steenkamp L, Venter D, Walsh C, Dana P. Socio-economic and demographic factors related to HIV status in urban informal settlements in the Eastern Cape, South Africa. Afr J AIDS Res. 2014; 13(3): 271-9.	353447
South Africa	2014	Attrition and Opportunities Along the HIV Care Continuum: Findings From a Population-based Sample,	Literature review	Admin 2	Lippman SA, Shade SB, El Ayadi AM, Gilvydis JM, Grignon JS, Liegler T, Morris J, Naidoo E, Prach LM, Puren A, Barnhart S. Attrition and Opportunities Along the HIV Care Continuum: Findings From a Population-Based	353451

Country	Years	Name	Type	Geographic Detail	Citation	NID*
		North West Province, South Africa			Sample, North West Province, South Africa. J Acquir Immune Defic Syndr. 2016; 73(1): 91-9.	
South Africa	2014	Transmission networks and risk of HIV infection in KwaZulu-Natal, South Africa: a community-wide phylogenetic study	Literature review	Site	de Oliveira T, Kharsany AB, Graf T, Cawood C, Khanyile D, Grobler A, Puren A, Madurai S, Baxter C, Karim QA, Karim SS. Transmission networks and risk of HIV infection in KwaZulu-Natal, South Africa: a community-wide phylogenetic study. Lancet HIV. 2017; 4(1): e41-e50.	353449
South Africa	2016	South Africa Demographic and Health Survey 2016	Survey microdata	GPS	Department of Health (South Africa), ICF International, South African Medical Research Council, Statistics South Africa. South Africa Demographic and Health Survey 2016. Fairfax, United States of America: ICF International, 2019.	157064
South Africa	2017	South Africa National HIV Prevalence, Incidence, Behavior and Communication Survey 2017	Survey report	Admin 1	Centers for Disease Control and Prevention (CDC), Human Sciences Research Council, National Institute for Communicable Diseases (South Africa), South African Medical Research Council. South Africa National HIV Prevalence, Incidence, Behavior and Communication Survey 2017.	357200
South Sudan	2002	HIV, syphilis, herpes simplex virus 2, and behavioral surveillance among conflict-affected populations in Yei and Rumbek, southern Sudan	Literature review	City	Kaiser R, Kedamo T, Lane J, Kessia G, Downing R, Handzel T, Marum E, Salama P, Mermin J, Brady W, Spiegel P. HIV, syphilis, herpes simplex virus 2, and behavioral surveillance among conflict-affected populations in Yei and Rumbek, southern Sudan. AIDS. 2006; 20(6): 942-4.	138559

Country	Years	Name	Type	Geographic Detail	Citation	NID*
Togo	2013-14	Togo Demographic and Health Survey 2013-2014	Survey microdata	GPS	Directorate General of Statistics and National Accounts (Togo), ICF International, Ministry of Health (Togo), Ministry of Planning, Development and Zoning (Togo). Togo Demographic and Health Survey 2013-2014. Fairfax, United States of America: ICF International, 2015.	77515
Uganda	1995-96	Seven-year trends in HIV-1 infection rates, and changes in sexual behaviour, among adults in rural Uganda	Literature review	Site	Kamali A, Carpenter LM, Whitworth JA, Pool R, Ruberantwari A, Ojwiya A. Seven-year trends in HIV-1 infection rates, and changes in sexual behaviour, among adults in rural Uganda. AIDS. 2000; 14(4): 427-34.	353502
Uganda	1997-04	HIV prevalence and incidence are no longer falling in southwest Uganda: evidence from a rural population cohort 1989-2005	Literature review	Site	Shafer LA, Biraro S, Nakiyingi-Miiro J, Kamali A, Ssematimba D, Ouma J, Ojwiya A, Hughes P, Van der Paal L, Whitworth J, Opio A, Grosskurth H. HIV prevalence and incidence are no longer falling in southwest Uganda: evidence from a rural population cohort 1989-2005. AIDS. 2008; 22(13): 1641-9.	353506
Uganda	2004	Sero-prevalence of herpes simplex type 2 virus (HSV-2) and HIV infection in Kampala, Uganda	Literature review	Admin 3	Nakku-Joloba E, Kambugu F, Wasubire J, Kimeze J, Salata R, Albert JM, Rimm A, Whalen C. Sero-prevalence of herpes simplex type 2 virus (HSV-2) and HIV infection in Kampala, Uganda. Afr Health Sci. 2014; 14(4): 782-9.	353493
Uganda	2004-05	Uganda AIDS Indicator Survey 2004-2005	Survey microdata	Admin 1	Division of Reproductive Health, Centers for Disease Control and Prevention (CDC), Ministry of Health (Uganda). Uganda AIDS Indicator Survey 2004-2005.	13084
Uganda	2008	High HIV Prevalence and Associated	Literature review	Admin 2	Rubaihayo J, Akib S, Mughusu E, Abaasa A. High HIV Prevalence and	353491

Country	Years	Name	Type	Geographic Detail	Citation	NID*
		Factors in a Remote Community in the Rwenzori Region of Western Uganda			Associated Factors in a Remote Community in the Rwenzori Region of Western Uganda. Infect Dis Rep. 2010; 2(2): e13.	
Uganda	2011	The Cango Lyec Project - Healing the Elephant : HIV related vulnerabilities of post-conflict affected populations aged 13-49 years living in three Mid-Northern Uganda districts	Literature review	Admin 2, Admin 3	Malamba SS, Muyinda H, Spittal PM, Ekwaru JP, Kiwanuka N, Ogwang MD, Odong P, Kitandwe PK, Katamba A, Jongbloed K, Sewankambo NK, Kinyanda E, Blair A, Schechter MT. The Cango Lyec Project - Healing the Elephant: HIV related vulnerabilities of post-conflict affected populations aged 13-49 years living in three Mid-Northern Uganda districts. BMC Infect Dis. 2016; 16(1): 690.	353487
Uganda	2011	Uganda AIDS Indicator Survey 2011	Survey microdata	GPS	Centers for Disease Control and Prevention (CDC), ICF Macro, Ministry of Health (Uganda), Uganda Bureau of Statistics, Uganda Virus Research Institute. Uganda AIDS Indicator Survey 2011. Fairfax, United States of America: ICF International.	55973
Uganda	2012	Heterogeneity of the HIV epidemic in agrarian, trading, and fishing communities in Rakai, Uganda: an observational epidemiological study	Literature review	Village	Chang LW, Grabowski MK, Ssekubugu R, Nalugoda F, Kigozi G, Nantume B, Lessler J, Moore SM, Quinn TC, Reynolds SJ, Gray RH, Serwadda D, Wawer MJ. Heterogeneity of the HIV epidemic in agrarian, trading, and fishing communities in Rakai, Uganda: an observational epidemiological study. Lancet HIV. 2016; 3(8): e388-e396.	353518
Uganda	2016	Uganda Population-based HIV Impact	Survey report	Admin 1	Ministry of Health, Uganda. Uganda Population-based HIV Impact Assessment (UPHIA) 2016-2017: Final	327593

Country	Years	Name	Type	Geographic Detail	Citation	NID*
		Assessment 2016-2017			Report. Kampala: Ministry of Health; July, 2019.	
Uganda, United Republic of Tanzania	2012	High prevalence of hypertension and of risk factors for non-communicable diseases (NCDs): a population based cross-sectional survey of NCDS and HIV infection in Northwestern Tanzania and Southern Uganda	Literature review	City	Kavishe B, Biraro S, Baisley K, Vanobberghen F, Kapiga S, Munderi P, Smeeth L, Peck R, Mghamba J, Mutungi G, Ikoona E, Levin J, Bou Monclús MA, Katende D, Kisanga E, Hayes R, Grosskurth H. High prevalence of hypertension and of risk factors for non-communicable diseases (NCDs): a population based cross-sectional survey of NCDS and HIV infection in Northwestern Tanzania and Southern Uganda. BMC Med. 2015; 13: 126.	247441
United Republic of Tanzania	1995	Assessment of behavioural risk factors associated with HIV infection among youth in Moshi rural district, Tanzania	Literature review	Admin 2	Tengia-Kessy A, Msamanga GI, Moshiro CS. Assessment of behavioural risk factors associated with HIV infection among youth in Moshi rural district, Tanzania. East Afr Med J. 1998; 75(9): 528-32.	353477
United Republic of Tanzania	1996	Decline in the prevalence of HIV-1 infection in young women in the Kagera region of Tanzania	Literature review	Admin 2	Kwesigabo G, Killewo J, Godoy C, Urassa W, Mbena E, Mhalu F, Biberfeld G, Wall S, Sandstrom A. Decline in the prevalence of HIV-1 infection in young women in the Kagera region of Tanzania. J Acquir Immune Defic Syndr Hum Retrovirol. 1998; 17(3): 262-8.	353469
United Republic of Tanzania	1996-03	HIV prevalence and incidence in rural Tanzania: results from 10 years of follow-up in an open-cohort study	Literature review	Admin 3	Wambura M, Urassa M, Isingo R, Ndege M, Marston M, Slaymaker E, Mngara J, Changalucha J, Boerma TJ, Zaba B. HIV prevalence and incidence in rural Tanzania: results from 10 years of follow-up in an open-cohort	353475

Country	Years	Name	Type	Geographic Detail	Citation	NID*
					study. J Acquir Immune Defic Syndr. 2007; 46(5): 616-23.	
United Republic of Tanzania	1997-05	Trends in HIV-1 prevalence and risk behaviours over 15 years in a rural population in Kilimanjaro region of Tanzania	Literature review	Village	Mmbaga EJ, Hussain A, Leyna GH, Holm-Hansen C, Mnyika KS, Sam NE, Klouman E, Klepp KI. Trends in HIV-1 prevalence and risk behaviours over 15 years in a rural population in Kilimanjaro region of Tanzania. AIDS Res Ther. 2007; 4: 23.	353479
United Republic of Tanzania	2003-04	Tanzania AIDS Indicator Survey 2003-2004	Survey microdata	GPS	National Bureau of Statistics (Tanzania), ORC Macro, Tanzania Commission for AIDS (TACAIDS). Tanzania AIDS Indicator Survey 2003-2004. Fairfax, United States of America: ICF International.	12630
United Republic of Tanzania	2007-08	Tanzania HIV/AIDS and Malaria Indicator Survey 2007-2008	Survey microdata	GPS	Macro International, Inc, National Bureau of Statistics (Tanzania), Office of the Chief Government Statistician (OCGS) (Zanzibar), Tanzania Commission for AIDS (TACAIDS), Zanzibar AIDS Commission (ZAC). Tanzania HIV/AIDS and Malaria Indicator Survey 2007-2008. Fairfax, United States of America: ICF International.	12644
United Republic of Tanzania	2010	Access to social capital and risk of HIV infection in Bukoba urban district, Kagera region, Tanzania	Literature review	Admin 2	Frumence G, Emmelin M, Eriksson M, Kwesigabo G, Killewo J, Moyo S, Nystrom L. Access to social capital and risk of HIV infection in Bukoba urban district, Kagera region, Tanzania. Arch Public Health. 2014; 72(1): 38.	353471
United Republic of Tanzania	2011-12	Tanzania AIDS Indicator Survey 2011-2012	Survey microdata	GPS	ICF International, National Bureau of Statistics (Tanzania), Office of the Chief Government Statistician (OCGS) (Zanzibar), Tanzania Commission for AIDS (TACAIDS), Zanzibar AIDS	77395

Country	Years	Name	Type	Geographic Detail	Citation	NID*
					Commission (ZAC). Tanzania AIDS Indicator Survey 2011-2012. Fairfax, United States of America: ICF International, 2013.	
United Republic of Tanzania	2017	Tanzania HIV Impact Survey 2016-2017	Survey microdata	Admin 1	Tanzania Commission for AIDS (TACAIDS), Zanzibar AIDS Commission (ZAC). Tanzania HIV Impact Survey(THIS) 2016-2017: Final Report. Dar es Salaam, Tanzania. December 2018.	327591
Zambia	1995-03	Steep HIV prevalence declines among young people in selected Zambian communities: population-based observations (1995-2003)	Literature review	Town	Michelo C, Sandoy IF, Dzekedzeke K, Siziya S, Fylkesnes K. Steep HIV prevalence declines among young people in selected Zambian communities: population-based observations (1995-2003). BMC Public Health. 2006; 6: 279.	353508
Zambia	1999	Susceptibility to intestinal infection and diarrhoea in Zambian adults in relation to HIV status and CD4 count	Literature review	Town	Kelly P, Todd J, Sianongo S, Mwansa J, Sinsungwe H, Katubulushi M, Farthing MJ, Feldman RA. Susceptibility to intestinal infection and diarrhoea in Zambian adults in relation to HIV status and CD4 count. BMC Gastroenterol. 2009; 9: 7.	353510
Zambia	2002	Zambia Demographic and Health Survey 2001-2002	Survey microdata	Admin 1	Central Board of Health (Zambia), Central Statistical Office (Zambia), Macro International, Inc. Zambia Demographic and Health Survey 2001-2002. Fairfax, United States of America: ICF International.	21102
Zambia	2007	Zambia Demographic and Health Survey 2007	Survey microdata	GPS	Central Statistical Office (Zambia), Macro International, Inc. Zambia Demographic and Health Survey 2007. Fairfax, United States of America: ICF International.	21117
Zambia	2013	The adult prevalence of	Literature review	Admin 1	Chanda-Kapata P, Kapata N, Klinkenberg E, William N,	353512

Country	Years	Name	Type	Geographic Detail	Citation	NID*
		HIV in Zambia: results from a population based mobile testing survey conducted in 2013-2014			Mazyanga L, Musukwa K, Kawesha EC, Masiye F, Mwaba P. The adult prevalence of HIV in Zambia: results from a population based mobile testing survey conducted in 2013-2014. AIDS Res Ther. 2016; 13: 4.	
Zambia	2013-14	Zambia Demographic and Health Survey 2013-2014	Survey microdata	GPS	Central Statistical Office (Zambia), ICF International, Ministry of Health (Zambia), Tropical Diseases Research Centre, University Teaching Hospital (Zambia), University of Zambia. Zambia Demographic and Health Survey 2013-2014. Fairfax, United States of America: ICF International.	77516
Zambia	2016	Zambia Population-based HIV Impact Assessment 2016	Survey microdata	Admin 2	Ministry of Health, Zambia, Centers for Disease Control and Prevention (CDC), and ICAP at Columbia University. Zambia Population-based HIV Impact Assessment (ZAMPHIA) 2015-16: First Report. Lusaka, Zambia, Atlanta, Georgia and New York, New York, USA: Ministry of Health, CDC and ICAP. December 2016.	287630
Zambia	2018	Zambia Demographic and Health Survey 2018-2019	Survey microdata	Admin 1	Central Statistical Office (Zambia), ICF International, Ministry of Health (Zambia), University Teaching Hospital (Zambia), University of Zambia. Zambia Demographic and Health Survey 2018-2019. Fairfax, United States of America: ICF International, 2020.	411301
Zimbabwe	2005	Zimbabwe - Chimanimani Behavioral Risks and HIV Serostatus Survey 2005	Survey report	Admin 2	Biomedical Research and Training Institute (Zimbabwe), Human Sciences Research Council, National Institute of Health Research, Ministry of Health and Child Welfare (NIHR) (Zimbabwe), Zimbabwe Central Statistics Office.	333662

Country	Years	Name	Type	Geographic Detail	Citation	NID*
					Zimbabwe - Chimanimani Behavioral Risks and HIV Serostatus Survey 2005.	
Zimbabwe	2005-06	Zimbabwe Demographic and Health Survey 2005-2006	Survey microdata	GPS	Central Statistical Office (Zimbabwe), Macro International, Inc. Zimbabwe Demographic and Health Survey 2005-2006. Fairfax, United States of America: ICF International.	21163
Zimbabwe	2010-11	Zimbabwe Demographic and Health Survey 2010-2011	Survey microdata	GPS	ICF Macro, Zimbabwe National Statistics Agency. Zimbabwe Demographic and Health Survey 2010-2011. Calverton, United States of America: ICF Macro, 2012.	55992
Zimbabwe	2015	Zimbabwe Demographic and Health Survey 2015	Survey microdata	GPS	ICF International, National Microbiology Reference Laboratory, Harare Central Hospital (NMRL) (Zimbabwe), Zimbabwe National Statistics Agency. Zimbabwe Demographic and Health Survey 2015. Fairfax, United States of America: ICF International, 2016.	157066
Zimbabwe	2016	Zimbabwe Population-based HIV Impact Assessment 2015-2016	Survey report	Admin 1	Ministry of Health and Child Care (MOHCC), Zimbabwe, Centers for Disease Control and Prevention (CDC), and ICAP at Columbia University. Zimbabwe Population-based HIV Impact Assessment (ZIMPHIA) 2015-16: First Report. Harare, Zimbabwe, Atlanta, Georgia and New York, New York, USA: Ministry of Health, CDC and ICAP. December 2016.	287631
Zimbabwe	2016	Zimbabwe Population-based HIV Impact Assessment 2015-2016	Survey report	Admin 1	Ministry of Health and Child Care (MOHCC), Zimbabwe, Centers for Disease Control and Prevention (CDC), and ICAP at Columbia University. Zimbabwe Population-based HIV Impact Assessment (ZIMPHIA) 2015-16: First Report. Harare, Zimbabwe, Atlanta, Georgia and New York, New York, USA:	287631

Country	Years	Name	Type	Geographic Detail	Citation	NID*
					Ministry of Health, CDC and ICAP. December 2016.	

*NID = Data source unique identifier in the Global Health Data Exchange (http://ghdx.healthdata.org/). Additional information about each data sources is available via the GHDx. NIDs can be entered in the search bar to retrieve the record for a particular source.

Appendix Table 5: ANC sentinel surveillance data

Country	Years	Source	Geographic Detail	Citation	NID*
Angola	1995-97, 1999, 2001-02	HIV Prevalence Among Women Attending Antenatal Care by Province 1985-2002	GPS	Angola Prevalence of HIV, Hepatitis B, and Syphilis, and HIV Prevalence Among Women Attending Antenatal Care, Personal Communication with K. Stanecki and D. Serrano. [Unpublished].	347096
Angola	2002	Presentation of HIV Surveillance Information and CAP Survey	GPS	Serrano D, National AIDS Control Program, Ministry of Health (PNLS) (Angola). Presentation of HIV Surveillance Information and CAP Survey.	347109
Angola	2004-05, 2007, 2009, 2011, 2013	UNAIDS Spectrum - National HIV Estimates 2019	GPS	Joint United Nations Program on HIV/AIDS (UNAIDS). UNAIDS Spectrum - National HIV Estimates 2019. Geneva, Switzerland: Joint United Nations Program on HIV/AIDS (UNAIDS), 2018.	415978
Benin	1995-17	UNAIDS Spectrum - National HIV Estimates 2019	GPS	Joint United Nations Program on HIV/AIDS (UNAIDS). UNAIDS Spectrum - National HIV Estimates 2019. Geneva, Switzerland: Joint United Nations Program on HIV/AIDS (UNAIDS), 2018.	415978
Benin	1997	Benin Surveillance of HIV/AIDS/MSM 1999	GPS	Ministry of Public Health (Benin), National Program Against AIDS (PNLS) (Benin). Benin Surveillance of HIV/AIDS/MSM 1999. Ministry of Public Health (Benin).	347119
Botswana	1995-03, 2005-07, 2009, 2011, 2015	UNAIDS Spectrum - National HIV Estimates 2019	Admin 2, GPS	Joint United Nations Program on HIV/AIDS (UNAIDS). UNAIDS Spectrum - National HIV Estimates 2019. Geneva, Switzerland: Joint United Nations Program on HIV/AIDS (UNAIDS), 2018.	415978
Botswana	1995-96, 2001-03	Botswana HIV Sentinel Survey 2003	Admin 2	Botswana AIDS/STD Unit, Botswana Harvard AIDS Institute Partnership (BHP), Centers for Disease Control and Prevention (CDC), Government of Botswana, National AIDS Coordinating Agency (Botswana), World Health Organization (WHO). Botswana HIV Sentinel Survey 2003.	347264
Botswana	1998, 2000	Botswana HIV Sentinel Survey 2000	Admin 2	Botswana AIDS/STD Unit, Centers for Disease Control and Prevention (CDC), Government of Botswana, National AIDS	347350

Country	Years	Source	Geographic Detail	Citation	NID*
				Coordinating Agency (Botswana), World Health Organization (WHO). Botswana HIV Sentinel Survey 2000.	
Botswana	2005	Botswana HIV Sentinel Survey 2005	Admin 2	Botswana Harvard AIDS Institute Partnership (BHP), Centers for Disease Control and Prevention (CDC), Department of AIDS Prevention and Care, Ministry of Health (Botswana), Joint United Nations Program on HIV/AIDS (UNAIDS), Ministry of Local Government (Botswana), National AIDS Coordinating Agency (Botswana), National Health Laboratory (NHL) (Botswana), World Health Organization (WHO). Botswana HIV Sentinel Survey 2005.	347307
Botswana	2007	Botswana HIV ANC Sentinel Survey 2007	Admin 2	African Comprehensive HIV/AIDS Partnerships (ACHAP), Botswana Harvard AIDS Institute Partnership (BHP), Centers for Disease Control and Prevention (CDC), Central Statistics Office (Botswana), Department of AIDS Prevention and Care, Ministry of Health (Botswana), Department of Local Government Service Management (DLGSM) (Botswana), Government of Botswana, Health Statistics Unit, Ministry of Health (Botswana), Joint United Nations Program on HIV/AIDS (UNAIDS), Ministry of Local Government (Botswana), National AIDS Coordinating Agency (Botswana), National Health Laboratory (NHL) (Botswana), World Health Organization (WHO). Botswana HIV ANC Sentinel Survey 2007.	347145
Burkina Faso	1995	Burkina Faso HIV Prevalence Data 1985-2002	GPS	Burkina Faso HIV Prevalence Data 1985-2002.	351969
Burkina Faso	1996-17	UNAIDS Spectrum - National HIV Estimates 2019	GPS	Joint United Nations Program on HIV/AIDS (UNAIDS). UNAIDS Spectrum - National HIV Estimates 2019. Geneva, Switzerland: Joint United Nations Program on HIV/AIDS (UNAIDS), 2018.	415978

Country	Years	Source	Geographic Detail	Citation	NID*
Burkina Faso	1997	Burkina Faso HIV/AIDS Epidemiological Surveillance Report 1997	GPS	Ministry of Health (Burkina Faso), National Committee Against AIDS and STDs (Burkina Faso). Burkina Faso HIV/AIDS Epidemiological Surveillance Report 1997. Burkina Faso: National Committee Against AIDS and STDs (Burkina Faso), 1998.	352076
Burundi	1995-98	Burundi Annual HIV/AIDS/STDs Epidemiological Surveillance Bulletin 2005	GPS	Ministry of Public Health (Burundi). Burundi Annual HIV/AIDS/STDs Epidemiological Surveillance Bulletin 2005. Bujumbura, Burundi: Ministry of Public Health (Burundi), 2005.	356867
Burundi	1999-10, 2015, 2017	UNAIDS Spectrum - National HIV Estimates 2019	GPS	Joint United Nations Program on HIV/AIDS (UNAIDS). UNAIDS Spectrum - National HIV Estimates 2019. Geneva, Switzerland: Joint United Nations Program on HIV/AIDS (UNAIDS), 2018.	415978
Burundi	2002	Burundi Annual HIV/AIDS/STIs Epidemiological Surveillance Report 2002	GPS	Ministry of Public Health (Burundi), National AIDS and STDs Program (Burundi). Burundi Annual HIV/AIDS/STIs Epidemiological Surveillance Report 2002. Burundi: National AIDS and STDs Program (Burundi), 2002.	352105
Cameroon	1995-98, 2000, 2002, 2007, 2009, 2012, 2016	UNAIDS Spectrum - National HIV Estimates 2019	GPS	Joint United Nations Program on HIV/AIDS (UNAIDS). UNAIDS Spectrum - National HIV Estimates 2019. Geneva, Switzerland: Joint United Nations Program on HIV/AIDS (UNAIDS), 2018.	415978
Cameroon	2002	Cameroon National HIV Serosurvey 2002	GPS	Ministry of Public Health (Cameroon), National AIDS Control Committee (Cameroon). Cameroon National HIV Serosurvey 2002.	352128
Cameroon	2016	UNAIDS Spectrum - National HIV Estimates 2017	GPS	Joint United Nations Program on HIV/AIDS (UNAIDS). UNAIDS Spectrum - National HIV Estimates 2017. Geneva, Switzerland: Joint United Nations Program on HIV/AIDS (UNAIDS), 2017.	317607
Cape Verde	1995-96	Cape Verde Sentinel Surveillance 1989-1997	Admin 2	National Program to Fight AIDS (PNLS) (Cape Verde). Cape Verde Sentinel Surveillance 1989-1997.	354752

Country	Years	Source	Geographic Detail	Citation	NID*
Cape Verde	1995-97, 2001-02, 2006-08	UNAIDS Spectrum - National HIV Estimates 2018	Admin 2	Joint United Nations Program on HIV/AIDS (UNAIDS). UNAIDS Spectrum - National HIV Estimates 2018. Geneva, Switzerland: Joint United Nations Program on HIV/AIDS (UNAIDS), 2018.	365412
Central African Republic	1995-00, 2002, 2006, 2011, 2015	UNAIDS Spectrum - National HIV Estimates 2019	GPS	Joint United Nations Program on HIV/AIDS (UNAIDS). UNAIDS Spectrum - National HIV Estimates 2019. Geneva, Switzerland: Joint United Nations Program on HIV/AIDS (UNAIDS), 2018.	415978
Chad	1999	Chad HIV Prevalence Among Women in ANC Per Sentinel Site 1999	GPS	Chad HIV prevalence among women in ANC per Sentinel SIte 1999.	356792
Chad	1999-00, 2002-03	UNAIDS Spectrum - National HIV Estimates 2016	GPS	Joint United Nations Program on HIV/AIDS (UNAIDS). UNAIDS Spectrum - National HIV Estimates 2016. Geneva, Switzerland: Joint United Nations Program on HIV/AIDS (UNAIDS).	306517
Chad	2000	Chad HIV Prevalence Among Women in ANC Per Sentinel Site 2000	GPS	Ministry of Public Health (Chad). Chad HIV Prevalence Among Women in ANC Per Sentinel Site 2000.	356793
Chad	2002	Chad Annual Report of Epidemiology Service Activities 2002, Epidemiological Surveillance of Second Generation HIV	GPS	Ministry of Public Health (Chad), National AIDS/STI Program (PNLS) (Chad). Chad Annual Report of Epidemiology Service Activities 2002, Epidemiological Surveillance of Second Generation HIV. N'Djamena, Chad: Ministry of Public Health (Chad), 2003.	354940
Chad	2002-03, 2009-11, 2013-14	UNAIDS Spectrum - National HIV Estimates 2019	GPS	Joint United Nations Program on HIV/AIDS (UNAIDS). UNAIDS Spectrum - National HIV Estimates 2019. Geneva, Switzerland: Joint United Nations Program on HIV/AIDS (UNAIDS), 2018.	415978
Chad	2002-03, 2011, 2013-14	UNAIDS Spectrum - National HIV Estimates 2018	GPS	Joint United Nations Program on HIV/AIDS (UNAIDS). UNAIDS Spectrum - National HIV Estimates 2018. Geneva, Switzerland: Joint United Nations Program on HIV/AIDS (UNAIDS), 2018.	365412

Country	Years	Source	Geographic Detail	Citation	NID*
Comoros	2007-08, 2011-16, 2018	UNAIDS Spectrum - National HIV Estimates 2019	Admin 1	Joint United Nations Program on HIV/AIDS (UNAIDS). UNAIDS Spectrum - National HIV Estimates 2019. Geneva, Switzerland: Joint United Nations Program on HIV/AIDS (UNAIDS), 2018.	415978
Congo	2002, 2005-06, 2011	UNAIDS Spectrum - National HIV Estimates 2019	Admin 1, Admin 2	Joint United Nations Program on HIV/AIDS (UNAIDS). UNAIDS Spectrum - National HIV Estimates 2019. Geneva, Switzerland: Joint United Nations Program on HIV/AIDS (UNAIDS), 2018.	415978
Côte d'Ivoire	1997-98, 2000-02, 2004-05, 2008, 2017	UNAIDS Spectrum - National HIV Estimates 2018	GPS	Joint United Nations Program on HIV/AIDS (UNAIDS). UNAIDS Spectrum - National HIV Estimates 2018. Geneva, Switzerland: Joint United Nations Program on HIV/AIDS (UNAIDS), 2018.	365412
Côte d'Ivoire	1997-98, 2000-02, 2004-05, 2008, 2017-18	UNAIDS Spectrum - National HIV Estimates 2019	GPS	Joint United Nations Program on HIV/AIDS (UNAIDS). UNAIDS Spectrum - National HIV Estimates 2019. Geneva, Switzerland: Joint United Nations Program on HIV/AIDS (UNAIDS), 2018.	415978
Côte d'Ivoire	2001	UNAIDS Spectrum - National HIV Estimates 2017	GPS	Joint United Nations Program on HIV/AIDS (UNAIDS). UNAIDS Spectrum - National HIV Estimates 2017. Geneva, Switzerland: Joint United Nations Program on HIV/AIDS (UNAIDS), 2017.	317607
Côte d'Ivoire	2005, 2008	UNAIDS Spectrum - National HIV Estimates 2016	GPS	Joint United Nations Program on HIV/AIDS (UNAIDS). UNAIDS Spectrum - National HIV Estimates 2016. Geneva, Switzerland: Joint United Nations Program on HIV/AIDS (UNAIDS).	306517
Democratic Republic of the Congo	1995-00, 2003-09, 2011-13, 2015, 2017	UNAIDS Spectrum - National HIV Estimates 2019	GPS	Joint United Nations Program on HIV/AIDS (UNAIDS). UNAIDS Spectrum - National HIV Estimates 2019. Geneva, Switzerland: Joint United Nations Program on HIV/AIDS (UNAIDS), 2018.	415978
Democratic Republic of the Congo	1997	Democratic Republic of the Congo AIDS/STD Notifications 1998	GPS	Ministry of Public Health (Congo, DR), National AIDS and STD Program (Congo, DR). Democratic Republic of the Congo AIDS/STD Notifications 1998. Kinshasa, Democratic Republic of the Congo: Ministry of Public Health (Congo, DR), 1999.	355211

Country	Years	Source	Geographic Detail	Citation	NID*
Djibouti	2002, 2006-10, 2013-14	UNAIDS Spectrum - National HIV Estimates 2019	GPS	Joint United Nations Program on HIV/AIDS (UNAIDS). UNAIDS Spectrum - National HIV Estimates 2019. Geneva, Switzerland: Joint United Nations Program on HIV/AIDS (UNAIDS), 2018.	415978
Equatorial Guinea	2008	Equatorial Guinea HIV Seroprevalence Survey 2008	Admin 1	Castroverde Laboratory (Equatorial Guinea), Institute of Health Carlos III (Spain), Ministry of Science and Innovation (Spain), Reference Center for Endemic Control (CRCE) (Equatorial Guinea), Spanish Agency for International Development Cooperation (AECID). Equatorial Guinea HIV Seroprevalence Survey 2008.	355263
Eritrea	1999, 2003	Eritrea HIV Sentinel Surveillance Survey 2003	GPS	Ministry of Health (Eritrea). Eritrea HIV Sentinel Surveillance Survey 2003.	355283
Eritrea	1999, 2003, 2005, 2007, 2009, 2011-13, 2015, 2017	UNAIDS Spectrum - National HIV Estimates 2019	GPS	Joint United Nations Program on HIV/AIDS (UNAIDS). UNAIDS Spectrum - National HIV Estimates 2019. Geneva, Switzerland: Joint United Nations Program on HIV/AIDS (UNAIDS), 2018.	415978
Eritrea	2003	Eritrea HIV Prevalence by Health Unit, Site, and Zone 1999-2003	GPS	Ministry of Health (Eritrea). Eritrea HIV Prevalence by Health Unit, Site, and Zone 1999-2003.	355296
Eritrea	2003, 2005, 2007	Eritrea HIV and Syphilis Sentinel Surveillance Survey in ANC Attendee Women 2007	GPS	Ministry of Health (Eritrea). Eritrea HIV and Syphilis Sentinel Surveillance Survey in ANC Attendee Women 2007.	143203
Eritrea	2003, 2005, 2007, 2009, 2011, 2013, 2015, 2018	UNAIDS Spectrum - National HIV Estimates 2018	GPS	Joint United Nations Program on HIV/AIDS (UNAIDS). UNAIDS Spectrum - National HIV Estimates 2018. Geneva, Switzerland: Joint United Nations Program on HIV/AIDS (UNAIDS), 2018.	365412

Country	Years	Source	Geographic Detail	Citation	NID*
Eswatini	1996, 1998, 2000, 2002, 2004, 2006, 2008, 2010	UNAIDS Spectrum - National HIV Estimates 2018	Admin 1, GPS	Joint United Nations Program on HIV/AIDS (UNAIDS). UNAIDS Spectrum - National HIV Estimates 2018. Geneva, Switzerland: Joint United Nations Program on HIV/AIDS (UNAIDS), 2018.	365412
Eswatini	1998	Swaziland HIV Sentinel Surveillance Survey 1998	Admin 1	Fogarty International Center, National Institutes of Health (NIH), Swaziland National AIDS Programme (SNAP), United Nations Development Programme (UNDP), United Nations Population Fund (UNFPA), World Health Organization (WHO). Swaziland HIV Sentinel Surveillance Survey 1998.	356634
Ethiopia	1997-99, 2001-03, 2005, 2007, 2009, 2012, 2014, 2016	UNAIDS Spectrum - National HIV Estimates 2018	GPS	Joint United Nations Program on HIV/AIDS (UNAIDS). UNAIDS Spectrum - National HIV Estimates 2018. Geneva, Switzerland: Joint United Nations Program on HIV/AIDS (UNAIDS), 2018.	365412
Ethiopia	1999, 2001-03	AIDS in Ethiopia: Fifth Report	GPS	Centers for Disease Control and Prevention (CDC), Ministry of Health (Ethiopia). AIDS in Ethiopia: Fifth Report. Addis Ababa, Ethiopia: Ministry of Health (Ethiopia), 2004.	316592
Ethiopia	2005	AIDS in Ethiopia: Sixth Report	GPS	Federal HIV/AIDS Prevention and Control Office (Ethiopia), Ministry of Health (Ethiopia), President's Emergency Plan for AIDS Relief (PEPFAR). AIDS in Ethiopia: Sixth Report. Addis Ababa, Ethiopia: Ministry of Health (Ethiopia).	143235
Ethiopia	2007, 2009	Ethiopia Antenatal Care Sentinel HIV Surveillance 2009	GPS	Centers for Disease Control and Prevention (CDC), Ethiopian Health and Nutrition Research Center (EHNRI). Ethiopia Antenatal Care Sentinel HIV Surveillance 2009.	355394
Gabon	1995, 2001-03, 2007, 2009	UNAIDS Spectrum - National HIV Estimates 2019	GPS	Joint United Nations Program on HIV/AIDS (UNAIDS). UNAIDS Spectrum - National HIV Estimates 2019. Geneva, Switzerland: Joint	415978

Country	Years	Source	Geographic Detail	Citation	NID*
				United Nations Program on HIV/AIDS (UNAIDS), 2018.	
Gabon	2001	Gabon HIV/AIDS Incidence, Prevalence, and Mortality 2001-2003	GPS	National AIDS Control Program (Gabon). Gabon HIV/AIDS Incidence, Prevalence, and Mortality 2001-2003. 2003.	355441
Gambia	1995, 2001-08, 2011-12, 2014, 2016-17	UNAIDS Spectrum - National HIV Estimates 2019	GPS	Joint United Nations Program on HIV/AIDS (UNAIDS). UNAIDS Spectrum - National HIV Estimates 2019. Geneva, Switzerland: Joint United Nations Program on HIV/AIDS (UNAIDS), 2018.	415978
Ghana	1995	Ghana HIV Sentinel Survey 1995	GPS	German Technical Cooperation Agency (GTZ), Ministry of Health (Ghana), National Public Health and Reference Laboratory (NHPRL)(Ghana), United States Agency for International Development (USAID), World Health Organization (WHO). Ghana HIV Sentinel Survey 1995.	355503
Ghana	1995-18	UNAIDS Spectrum - National HIV Estimates 2019	GPS	Joint United Nations Program on HIV/AIDS (UNAIDS). UNAIDS Spectrum - National HIV Estimates 2019. Geneva, Switzerland: Joint United Nations Program on HIV/AIDS (UNAIDS), 2018.	415978
Ghana	1996	Ghana HIV Sentinel Survey 1996	GPS	Ministry of Health (Ghana), National Public Health and Reference Laboratory (NHPRL)(Ghana), United States Agency for International Development (USAID). Ghana HIV Sentinel Survey 1996.	355500
Ghana	1997-98	Ghana HIV/STD Sentinel Survey 1997	GPS	Ministry of Health (Ghana), National AIDS Control Program (NACP) (Ghana), National Public Health and Reference Laboratory (NHPRL)(Ghana), United States Agency for International Development (USAID), World Health Organization (WHO). Ghana HIV/STD Sentinel Survey 1997.	355498
Ghana	1999	Ghana HIV Prevalence by Sentinel Surveillance Site 1999	GPS	Ghana HIV Prevalence by Sentinel Surveillance Site 1999.	356790

Country	Years	Source	Geographic Detail	Citation	NID*
Ghana	2000	Ghana HIV Sentinel Survey 2000	GPS	Ministry of Health (Ghana), National AIDS Control Program (NACP) (Ghana), National Public Health and Reference Laboratory (NHPRL) (Ghana). Ghana HIV Sentinel Survey 2000.	355496
Ghana	2001	Ghana HIV Sentinel Survey 2001	GPS	Ministry of Health (Ghana), National AIDS Control Program (NACP) (Ghana), National Public Health and Reference Laboratory (NHPRL) (Ghana). Ghana HIV Sentinel Survey 2001.	355494
Ghana	2003	Ghana HIV Sentinel Survey 2003	GPS	Ghana Health Service, National AIDS Control Program (NACP) (Ghana), National Public Health and Reference Laboratory (NHPRL) (Ghana), Noguchi Memorial Institute for Medical Research (NMIMR) (Ghana). Ghana HIV Sentinel Survey 2003.	355475
Ghana	2005	Ghana HIV Sentinel Survey 2005	GPS	Ghana Health Service, National AIDS Control Program (NACP) (Ghana), National Public Health and Reference Laboratory (NHPRL) (Ghana), Noguchi Memorial Institute for Medical Research (NMIMR) (Ghana). Ghana HIV Sentinel Survey 2005.	355473
Ghana	2006	Ghana HIV Sentinel Survey 2006	GPS	Ghana Health Service, National AIDS Control Program (NACP) (Ghana), National Public Health and Reference Laboratory (NHPRL) (Ghana), Noguchi Memorial Institute for Medical Research (NMIMR) (Ghana). Ghana HIV Sentinel Survey 2006.	355458
Guinea	1995-96, 2001, 2004, 2008, 2015, 2018	UNAIDS Spectrum - National HIV Estimates 2019	GPS	Joint United Nations Program on HIV/AIDS (UNAIDS). UNAIDS Spectrum - National HIV Estimates 2019. Geneva, Switzerland: Joint United Nations Program on HIV/AIDS (UNAIDS), 2018.	415978
Guinea	2015	UNAIDS Spectrum - National HIV Estimates 2016	GPS	Joint United Nations Program on HIV/AIDS (UNAIDS). UNAIDS Spectrum - National HIV Estimates 2016. Geneva, Switzerland: Joint United Nations Program on HIV/AIDS (UNAIDS).	306517

Country	Years	Source	Geographic Detail	Citation	NID*
Guinea-Bissau	1995-96, 1998-05, 2010, 2014	UNAIDS Spectrum - National HIV Estimates 2019	Admin 2	Joint United Nations Program on HIV/AIDS (UNAIDS). UNAIDS Spectrum - National HIV Estimates 2019. Geneva, Switzerland: Joint United Nations Program on HIV/AIDS (UNAIDS), 2018.	415978
Kenya	1995	Estimating National HIV Prevalence in Kenya from Sentinel Surveillance Data	GPS	National AIDS and STI Control Program (Kenya). Estimating National HIV Prevalence in Kenya from Sentinel Surveillance Data. Nairobi, Kenya: National AIDS and STI Control Program (Kenya), 1999.	355617
Kenya	1995-11	UNAIDS Spectrum - National HIV Estimates 2018	GPS	Joint United Nations Program on HIV/AIDS (UNAIDS). UNAIDS Spectrum - National HIV Estimates 2018. Geneva, Switzerland: Joint United Nations Program on HIV/AIDS (UNAIDS), 2018.	365412
Kenya	1995-96, 1999	AIDS in Kenya 2001	GPS	Ministry of Health (Kenya), National AIDS Control Council (Kenya). AIDS in Kenya 2001. Nairobi, Kenya: Ministry of Health (Kenya), 2001.	355597
Kenya	2010	Kenya ANC HIV Sentinel Survey 2010	GPS	Kenya Medical Research Institute (KEMRI), National AIDS and STI Control Programme (NASCOP) (Kenya), National HIV Reference Laboratory (NHRL) (Kenya), National Public Health Laboratory Services, Ministry of Public Health and Sanitation (Kenya), World Health Organization (WHO). Kenya ANC HIV Sentinel Survey 2010.	355605
Kenya	2011	Kenya ANC HIV Sentinel Survey 2011	GPS	Kenya Medical Research Institute (KEMRI), National AIDS and STI Control Programme (NASCOP) (Kenya), National HIV Reference Laboratory (NHRL) (Kenya), National Public Health Laboratory Services, Ministry of Public Health and Sanitation (Kenya), World Health Organization (WHO). Kenya ANC HIV Sentinel Survey 2011.	355607
Lesotho	1996, 2000, 2003, 2005,	UNAIDS Spectrum - National HIV Estimates 2019	GPS	Joint United Nations Program on HIV/AIDS (UNAIDS). UNAIDS Spectrum - National HIV Estimates 2019. Geneva, Switzerland: Joint	415978

Country	Years	Source	Geographic Detail	Citation	NID*
	2009, 2011, 2013, 2016, 2018			United Nations Program on HIV/AIDS (UNAIDS), 2018.	
Liberia	2006-08, 2011, 2013, 2017	UNAIDS Spectrum - National HIV Estimates 2019	GPS	Joint United Nations Program on HIV/AIDS (UNAIDS). UNAIDS Spectrum - National HIV Estimates 2019. Geneva, Switzerland: Joint United Nations Program on HIV/AIDS (UNAIDS), 2018.	415978
Liberia	2007	UNAIDS Spectrum - National HIV Estimates 2016	GPS	Joint United Nations Program on HIV/AIDS (UNAIDS). UNAIDS Spectrum - National HIV Estimates 2016. Geneva, Switzerland: Joint United Nations Program on HIV/AIDS (UNAIDS).	306517
Liberia	2007-08	Liberia HIV Sentinel Survey 2008	GPS	Global Fund to Fight Aids Tuberculosis and Malaria (GFATM), National AIDS and STI Control Program (NACP), Ministry of Health and Social Welfare (Liberia), National Public Health Reference Laboratory (Liberia), United Nations Development Programme (UNDP), World Health Organization (WHO). Liberia HIV Sentinel Survey 2008.	355669
Madagascar	1995-96, 2003	Madagascar Towards Knowledge of its Epidemic: Analysis of the Epidemiological Profile and Determinants of the HIV Infection	GPS	Executive Secretariat of the National Committee for the Fight against HIV/AIDS (SE CNLS) (Madagascar), Joint United Nations Program on HIV/AIDS (UNAIDS), United States Agency for International Development (USAID), World Bank, World Health Organization (WHO). Madagascar Towards Knowledge of its Epidemic: Analysis of the Epidemiological Profile and Determinants of the HIV Infection. Antananarivo, Madagascar: Executive Secretariat of the National Committee for the Fight against HIV/AIDS (SE CNLS) (Madagascar), 2008.	355675
Madagascar	2005	Madagascar Biological Surveillance Survey 2005	GPS	Centers for Disease Control and Prevention (CDC), French Cooperation, Joint United Nations Program on HIV/AIDS (UNAIDS),	355671

Country	Years	Source	Geographic Detail	Citation	NID*
				Ministry of Health and Family Planning (Madagascar), National Institute of Public and Community Health (INSPC) (Madagascar), Pasteur Institute of Madagascar (IPM), United Nations Children's Fund (UNICEF), United States Agency for International Development (USAID), World Health Organization (WHO). Madagascar Biological Surveillance Survey 2005.	
Madagascar	2005, 2007, 2010-18	UNAIDS Spectrum - National HIV Estimates 2019	GPS	Joint United Nations Program on HIV/AIDS (UNAIDS). UNAIDS Spectrum - National HIV Estimates 2019. Geneva, Switzerland: Joint United Nations Program on HIV/AIDS (UNAIDS), 2018.	415978
Malawi	1995-96, 1998-99, 2003	Malawi HIV and Syphilis Sentinel Surveillance of Antenatal Clinic Attendees 2003	GPS	Ministry of Health (Malawi), National AIDS Commission (Malawi). Malawi HIV and Syphilis Sentinel Surveillance of Antenatal Clinic Attendees 2003.	157140
Malawi	1995-99, 2001, 2003, 2005, 2007, 2010	UNAIDS Spectrum - National HIV Estimates 2019	GPS	Joint United Nations Program on HIV/AIDS (UNAIDS). UNAIDS Spectrum - National HIV Estimates 2019. Geneva, Switzerland: Joint United Nations Program on HIV/AIDS (UNAIDS), 2018.	415978
Malawi	1997	Malawi HIV/Syphilis Seroprevalence Survey of Antenatal Clinic Attendees 1997-1998	GPS	Community Health Sciences Unit (Malawi), National AIDS Control Program (Malawi). Malawi HIV/Syphilis Seroprevalence Survey of Antenatal Clinic Attendees 1997-1998.	355681
Malawi	1999	Malawi HIV/Syphilis Seroprevalence Survey of Antenatal Clinic Attendees 1999-2000	GPS	Community Health Sciences Unit (Malawi), National AIDS Control Program (Malawi), University of North Carolina. Malawi HIV/Syphilis Seroprevalence Survey of Antenatal Clinic Attendees 1999-2000.	355679
Malawi	2001	HIV/AIDS in Malawi: 2003 Estimates and Implications	GPS	Ministry of Health and Population (Malawi), National AIDS Commission (Malawi). HIV/AIDS in Malawi: 2003 Estimates and Implications. Lilongwe, Malawi: National AIDS Commission (Malawi).	355677

Country	Years	Source	Geographic Detail	Citation	NID*
Malawi	2007	Malawi HIV and Syphilis Sero-Survey of Antenatal Clinic Attendees 2007	GPS	Ministry of Health (Malawi), National AIDS Commission (Malawi). Malawi HIV and Syphilis Sero-Survey of Antenatal Clinic Attendees 2007.	157132
Mali	1997, 1999	Epidemiological Surveillance of HIV in Mali 1985-2004	GPS	National AIDS Control Program (Mali). Epidemiological Surveillance of HIV in Mali 1985-2004. Bamako, Mali: Ministry of Health (Mali).	355683
Mali	2002-03, 2005, 2007, 2009, 2012	UNAIDS Spectrum - National HIV Estimates 2019	GPS	Joint United Nations Program on HIV/AIDS (UNAIDS). UNAIDS Spectrum - National HIV Estimates 2019. Geneva, Switzerland: Joint United Nations Program on HIV/AIDS (UNAIDS), 2018.	415978
Mali	2007	Mali HIV and Syphilis Sentinel Surveillance Survey 2007	GPS	Centers for Disease Control and Prevention (CDC), INFO-STAT (Mali), Ministry of Health (Mali), National Institute of Public Health Research (INRSP) (Mali), Sectoral Committee for the Fight Against AIDS (CSLS) (Mali). Mali HIV and Syphilis Sentinel Surveillance Survey 2007.	355931
Mauritania	2001, 2003, 2005, 2007, 2009	UNAIDS Spectrum - National HIV Estimates 2019	GPS	Joint United Nations Program on HIV/AIDS (UNAIDS). UNAIDS Spectrum - National HIV Estimates 2019. Geneva, Switzerland: Joint United Nations Program on HIV/AIDS (UNAIDS), 2018.	415978
Mauritania	2001, 2003, 2007	Mauritania HIV Sentinel Surveillance Survey 2007	GPS	Joint United Nations Program on HIV/AIDS (UNAIDS), Ministry of Health and Social Affairs (Mauritania), National Executive Secretariat for AIDS Control (SENLS) (Mauritania), National Institute of Public Health Research (INRSP) (Mauritania), United Nations Children's Fund (UNICEF). Mauritania HIV Sentinel Surveillance Survey 2007.	356151
Mozambique	1996, 1998, 2000-02, 2004, 2007, 2009, 2011	UNAIDS Spectrum - National HIV Estimates 2019	GPS	Joint United Nations Program on HIV/AIDS (UNAIDS). UNAIDS Spectrum - National HIV Estimates 2019. Geneva, Switzerland: Joint United Nations Program on HIV/AIDS (UNAIDS), 2018.	415978

Country	Years	Source	Geographic Detail	Citation	NID*
Mozambique	2000	Mozambique Results of Epidemiological Surveillance - Sentinel Posts 2000	GPS	Ministry of Health (Mozambique). Mozambique Results of Epidemiological Surveillance - Sentinel Posts 2000.	356796
Namibia	1996, 1998, 2000, 2002, 2004, 2006, 2008, 2010, 2012, 2014, 2016-17	UNAIDS Spectrum - National HIV Estimates 2019	Admin 2, GPS	Joint United Nations Program on HIV/AIDS (UNAIDS). UNAIDS Spectrum - National HIV Estimates 2019. Geneva, Switzerland: Joint United Nations Program on HIV/AIDS (UNAIDS), 2018.	415978
Namibia	1996-98	Mission Report, Dr. Emil Asamoah-odei, EPS/AFRO Windhoek, Namibia 17-20 July 2000	Admin 2, GPS	Mission Report, Dr. Emil Asamoah-odei, EPS/AFRO Windhoek, Namibia 17-20 July 2000.	356161
Namibia	1997	Namibia Epidemiological Report on HIV/AIDS 1999	Admin 2	National AIDS Coordination Program (Namibia). Namibia Epidemiological Report on HIV/AIDS 1999. Namibia: National AIDS Coordination Program (Namibia), 2000.	356157
Namibia	1998, 2000, 2002, 2004, 2006, 2008, 2010, 2012, 2014, 2016	UNAIDS Spectrum - National HIV Estimates 2017	GPS, Admin 2	Joint United Nations Program on HIV/AIDS (UNAIDS). UNAIDS Spectrum - National HIV Estimates 2017. Geneva, Switzerland: Joint United Nations Program on HIV/AIDS (UNAIDS), 2017.	317607
Namibia	2000	Namibia Epidemiological Report on HIV/AIDS for the Year 2000	Admin 2	National AIDS Coordination Program (Namibia). Namibia Epidemiological Report on HIV/AIDS for the Year 2000. Namibia: National AIDS Coordination Program (Namibia), 2001.	356159
Namibia	2002	Namibia National HIV Sentinel Survey 2002	Admin 2, GPS	Ministry of Health and Social Services (Namibia), Namibia Institute of Pathology, National	356248

Country	Years	Source	Geographic Detail	Citation	NID*
				AIDS Coordination Program (Namibia). Namibia National HIV Sentinel Survey 2002.	
Namibia	2014, 2016	UNAIDS Spectrum - National HIV Estimates 2018	GPS, Admin 2	Joint United Nations Program on HIV/AIDS (UNAIDS). UNAIDS Spectrum - National HIV Estimates 2018. Geneva, Switzerland: Joint United Nations Program on HIV/AIDS (UNAIDS), 2018.	365412
Niger	1995, 1997, 1999-00	Niger HIV Epidemiological Surveillance Report 2001	GPS	National Program for the Fight Against AIDS/STDs (PNLS/MST) (Niger). Niger HIV Epidemiological Surveillance Report 2001.	356296
Niger	1995, 1999-00, 2009, 2012, 2014	UNAIDS Spectrum - National HIV Estimates 2019	GPS	Joint United Nations Program on HIV/AIDS (UNAIDS). UNAIDS Spectrum - National HIV Estimates 2019. Geneva, Switzerland: Joint United Nations Program on HIV/AIDS (UNAIDS), 2018.	415978
Nigeria	1995	Nigeria Sentinel Surveillance Survey 1995-1996	GPS	World Health Organization (WHO). Nigeria Sentinel Surveillance Survey 1995-1996.	356285
Nigeria	1995, 1999-03, 2005, 2008, 2010, 2014, 2017	UNAIDS Spectrum - National HIV Estimates 2019	GPS	Joint United Nations Program on HIV/AIDS (UNAIDS). UNAIDS Spectrum - National HIV Estimates 2019. Geneva, Switzerland: Joint United Nations Program on HIV/AIDS (UNAIDS), 2018.	415978
Nigeria	1999	Nigeria HIV/Syphilis Sentinel Seroprevalence Survey 1999	GPS	Department for International Development (DFiD) (United Kingdom), Family Health International, Federal Ministry of Health (Nigeria), Petroleum Trust Fund (PTF) (Nigeria), United Nations Children's Fund (UNICEF), United States Agency for International Development (USAID), University College Hospital, Ibadan, University of Jos, World Health Organization (WHO). Nigeria HIV/Syphilis Sentinel Seroprevalence Survey 1999.	356272
Nigeria	2003	Nigeria National HIV Seroprevalence Sentinel Survey 2003	GPS	Federal Ministry of Health (Nigeria). Nigeria National HIV Seroprevalence Sentinel Survey 2003.	152886

Country	Years	Source	Geographic Detail	Citation	NID*
Nigeria	2003, 2005, 2008, 2010, 2014	UNAIDS Spectrum - National HIV Estimates 2018	GPS	Joint United Nations Program on HIV/AIDS (UNAIDS). UNAIDS Spectrum - National HIV Estimates 2018. Geneva, Switzerland: Joint United Nations Program on HIV/AIDS (UNAIDS), 2018.	365412
Nigeria	2005	Nigeria National HIV/Syphilis Seroprevalence Sentinel Survey 2005	GPS	Federal Ministry of Health (Nigeria). Nigeria National HIV/Syphilis Seroprevalence Sentinel Survey 2005.	138348
Nigeria	2005, 2008, 2010	Nigeria National HIV Seroprevalence Sentinel Survey 2010	GPS	Federal Ministry of Health (Nigeria). Nigeria National HIV Seroprevalence Sentinel Survey 2010.	152887
Rwanda	1996, 1998, 2002-03, 2005, 2007, 2011, 2013	UNAIDS Spectrum - National HIV Estimates 2016	GPS	Joint United Nations Program on HIV/AIDS (UNAIDS). UNAIDS Spectrum - National HIV Estimates 2016. Geneva, Switzerland: Joint United Nations Program on HIV/AIDS (UNAIDS).	306517
Rwanda	1996, 1998, 2002-03, 2005, 2007, 2011, 2013	UNAIDS Spectrum - National HIV Estimates 2019	GPS	Joint United Nations Program on HIV/AIDS (UNAIDS). UNAIDS Spectrum - National HIV Estimates 2019. Geneva, Switzerland: Joint United Nations Program on HIV/AIDS (UNAIDS), 2018.	415978
Rwanda	1998, 2002	Rwanda HIV Sentinel Surveillance Survey 2002	GPS	Centers for Disease Control and Prevention (CDC), IMPACT Rwanda, Ministry of Health (Rwanda). Rwanda HIV Sentinel Surveillance Survey 2002.	356349
Senegal	1995-01	Senegal HIV Prevalence in Men and Pregnant Women by Region 1989-2002	Admin 1, GPS	Ministry of Health and Prevention (Senegal). Senegal HIV Prevalence in Men and Pregnant Women by Region 1989-2002.	356379
Senegal	1995-07, 2009, 2011, 2014, 2018	UNAIDS Spectrum - National HIV Estimates 2019	Admin 1, GPS	Joint United Nations Program on HIV/AIDS (UNAIDS). UNAIDS Spectrum - National HIV Estimates 2019. Geneva, Switzerland: Joint United Nations Program on HIV/AIDS (UNAIDS), 2018.	415978
Senegal	2002	Senegal Sero-Epidemiological Bulletin on HIV	Admin 1, GPS	Ministry of Health and Prevention (Senegal), National Council for the Fight Against AIDS (Senegal).	356380

Country	Years	Source	Geographic Detail	Citation	NID*
		Surveillance No. 10, July 2003		Senegal Sero-Epidemiological Bulletin on HIV Surveillance No. 10, July 2003. Dakar, Senegal: National Council for the Fight Against AIDS (Senegal), 2003.	
Senegal	2005-07	Senegal Epidemiological Bulletin on Sentinel Surveillance of HIV and Syphilis in Pregnant Women No. 13, November 2008	Admin 1	Ministry of Health and Prevention (Senegal), National Council for the Fight Against AIDS (Senegal). Senegal Epidemiological Bulletin on Sentinel Surveillance of HIV and Syphilis in Pregnant Women No. 13, November 2008. Dakar, Senegal: National Council for the Fight Against AIDS (Senegal), 2008.	356395
Sierra Leone	2003, 2006-08, 2010, 2018	UNAIDS Spectrum - National HIV Estimates 2019	GPS	Joint United Nations Program on HIV/AIDS (UNAIDS). UNAIDS Spectrum - National HIV Estimates 2019. Geneva, Switzerland: Joint United Nations Program on HIV/AIDS (UNAIDS), 2018.	415978
Sierra Leone	2006-07	UNAIDS Spectrum - National HIV Estimates 2018	GPS	Joint United Nations Program on HIV/AIDS (UNAIDS). UNAIDS Spectrum - National HIV Estimates 2018. Geneva, Switzerland: Joint United Nations Program on HIV/AIDS (UNAIDS), 2018.	365412
Sierra Leone	2015	UNAIDS Spectrum - National HIV Estimates 2016	GPS	Joint United Nations Program on HIV/AIDS (UNAIDS). UNAIDS Spectrum - National HIV Estimates 2016. Geneva, Switzerland: Joint United Nations Program on HIV/AIDS (UNAIDS).	306517
Somalia	1997, 1999, 2004, 2007, 2010-12, 2014, 2016	UNAIDS Spectrum - National HIV Estimates 2019	GPS	Joint United Nations Program on HIV/AIDS (UNAIDS). UNAIDS Spectrum - National HIV Estimates 2019. Geneva, Switzerland: Joint United Nations Program on HIV/AIDS (UNAIDS), 2018.	415978
South Africa	1995-14	UNAIDS Spectrum - National HIV Estimates 2017	Admin 1, Admin 2, Admin 3	Joint United Nations Program on HIV/AIDS (UNAIDS). UNAIDS Spectrum - National HIV Estimates 2017. Geneva, Switzerland: Joint United Nations Program on HIV/AIDS (UNAIDS), 2017.	317607
South Africa	2006-08	South Africa National Antenatal Sentinel HIV and	Admin 2, Admin 3	Department of Health (South Africa). South Africa National Antenatal Sentinel HIV and Syphilis Prevalence Survey 2008.	356401

Country	Years	Source	Geographic Detail	Citation	NID*
		Syphilis Prevalence Survey 2008			
South Sudan	2007, 2009, 2012	UNAIDS Spectrum - National HIV Estimates 2016	GPS	Joint United Nations Program on HIV/AIDS (UNAIDS). UNAIDS Spectrum - National HIV Estimates 2016. Geneva, Switzerland: Joint United Nations Program on HIV/AIDS (UNAIDS).	306517
South Sudan	2007, 2009, 2012, 2017	UNAIDS Spectrum - National HIV Estimates 2019	GPS	Joint United Nations Program on HIV/AIDS (UNAIDS). UNAIDS Spectrum - National HIV Estimates 2019. Geneva, Switzerland: Joint United Nations Program on HIV/AIDS (UNAIDS), 2018.	415978
Sudan	2004-05, 2007, 2009-10	UNAIDS Spectrum - National HIV Estimates 2019	GPS	Joint United Nations Program on HIV/AIDS (UNAIDS). UNAIDS Spectrum - National HIV Estimates 2019. Geneva, Switzerland: Joint United Nations Program on HIV/AIDS (UNAIDS), 2018.	415978
Togo	1995-97, 2001, 2003-04, 2006-11, 2014, 2016	UNAIDS Spectrum - National HIV Estimates 2019	GPS	Joint United Nations Program on HIV/AIDS (UNAIDS). UNAIDS Spectrum - National HIV Estimates 2019. Geneva, Switzerland: Joint United Nations Program on HIV/AIDS (UNAIDS), 2018.	415978
Togo	2011	Togo National HIV and Syphilis Sentinel Surveillance Survey 2011-2012	GPS	Directorate General of Health (DGS) (Togo), Joint United Nations Program on HIV/AIDS (UNAIDS), National Program for the Fight Against HIV/AIDS (PNLS) (Togo), World Health Organization (WHO). Togo National HIV and Syphilis Sentinel Surveillance Survey 2011-2012.	356774
Uganda	1995-05, 2007, 2009-12, 2014, 2016, 2018	UNAIDS Spectrum - National HIV Estimates 2019	GPS	Joint United Nations Program on HIV/AIDS (UNAIDS). UNAIDS Spectrum - National HIV Estimates 2019. Geneva, Switzerland: Joint United Nations Program on HIV/AIDS (UNAIDS), 2018.	415978
Uganda	1996-01, 2003-07, 2009	Uganda HIV/AIDS Epidemiological Surveillance Report 2010	GPS	Centers for Disease Control and Prevention (CDC), Medical Research Council (Uganda), Ministry of Health (Uganda), National Institute of Health (Italy), Rakai Health Sciences Program (Uganda), STD/AIDS Control Program, Ministry of Health	321955

Country	Years	Source	Geographic Detail	Citation	NID*
				(Uganda), Uganda Virus Research Institute. Uganda HIV/AIDS Epidemiological Surveillance Report 2010. Uganda: STD/AIDS Control Program, Ministry of Health (Uganda), 2010.	
Uganda	2005-07	Uganda HIV/AIDS Epidemiological Surveillance Report 2005-2007	GPS	Centers for Disease Control and Prevention (CDC), Joint United Nations Program on HIV/AIDS (UNAIDS), Medical Research Council (Uganda), Ministry of Health (Uganda), National Institute of Health (Italy), Rakai Health Sciences Program (Uganda), STD/AIDS Control Program, Ministry of Health (Uganda), Uganda AIDS Commission, Uganda Virus Research Institute, United States Agency for International Development (USAID), World Health Organization (WHO). Uganda HIV/AIDS Epidemiological Surveillance Report 2005-2007. Uganda: STD/AIDS Control Program, Ministry of Health (Uganda), 2009.	356783
United Republic of Tanzania	1995-00, 2005, 2008-09, 2011, 2017	UNAIDS Spectrum - National HIV Estimates 2018	GPS	Joint United Nations Program on HIV/AIDS (UNAIDS). UNAIDS Spectrum - National HIV Estimates 2018. Geneva, Switzerland: Joint United Nations Program on HIV/AIDS (UNAIDS), 2018.	365412
United Republic of Tanzania	1995-03, 2005, 2008, 2010-11, 2014, 2017-18	UNAIDS Spectrum - National HIV Estimates 2019	GPS	Joint United Nations Program on HIV/AIDS (UNAIDS). UNAIDS Spectrum - National HIV Estimates 2019. Geneva, Switzerland: Joint United Nations Program on HIV/AIDS (UNAIDS), 2018.	415978
United Republic of Tanzania	1996, 1999-01, 2003, 2005, 2008, 2011	UNAIDS Spectrum - National HIV Estimates 2017	GPS	Joint United Nations Program on HIV/AIDS (UNAIDS). UNAIDS Spectrum - National HIV Estimates 2017. Geneva, Switzerland: Joint United Nations Program on HIV/AIDS (UNAIDS), 2017.	317607
United Republic of Tanzania	1999	Tanzania HIV Prevalence by Region, Antenatal Clinic and Age Group 1999	GPS	Tanzania HIV Prevalence by Region, Antenatal Clinic and Age Group 1999.	356682

Country	Years	Source	Geographic Detail	Citation	NID*
Zambia	1998, 2002, 2004, 2006, 2008, 2011, 2018	UNAIDS Spectrum - National HIV Estimates 2019	GPS	Joint United Nations Program on HIV/AIDS (UNAIDS). UNAIDS Spectrum - National HIV Estimates 2019. Geneva, Switzerland: Joint United Nations Program on HIV/AIDS (UNAIDS), 2018.	415978
Zimbabwe	1995, 1997	UNAIDS Spectrum - National HIV Estimates 2016	GPS	Joint United Nations Program on HIV/AIDS (UNAIDS). UNAIDS Spectrum - National HIV Estimates 2016. Geneva, Switzerland: Joint United Nations Program on HIV/AIDS (UNAIDS).	306517
Zimbabwe	1995-97, 1999-02, 2004, 2006, 2009, 2012, 2016	UNAIDS Spectrum - National HIV Estimates 2019	GPS	Joint United Nations Program on HIV/AIDS (UNAIDS). UNAIDS Spectrum - National HIV Estimates 2019. Geneva, Switzerland: Joint United Nations Program on HIV/AIDS (UNAIDS), 2018.	415978

*NID = Data source unique identifier in the Global Health Data Exchange (http://ghdx.healthdata.org/). Additional information about each data sources is available via the GHDx. NIDs can be entered in the search bar to retrieve the record for a particular source.

Appendix Table 6: HIV covariate survey data

Country	Years	Name	Type	Geographic Detail	Male Circumcision	STI Symptoms	Had Intercourse (Women)	Partner Away	Condom Last Time	Multiple Partners in Year (Men)	Multiple Partners in Year (Women)	In Union	Citation	NID*
Angola	2001	Angola Multiple Indicator Cluster Survey 2001	Survey microdata	Admin 1								X	National Institute of Statistics (Angola), United Nations Children's Fund (UNICEF). Angola Multiple Indicator Cluster Survey 2001. New York, United States of America: United Nations Children's Fund (UNICEF).	687
Angola	2006	Angola Core Welfare Indicators Questionnaire Survey 2005	Survey microdata	Admin 1								X	National Institute of Statistics (Angola). Angola Core Welfare Indicators Questionnaire Survey 2005. Luanda, Angola: National Institute of Statistics (Angola).	151566
Angola	2011	Angola Core Welfare Indicators Questionnaire Survey 2011	Survey microdata	Admin 1								X	Ministry of Planning and Territorial Development (Angola), National Institute of Statistics (Angola). Angola Core Welfare Indicators Questionnaire Survey 2011. Luanda, Angola: National Institute of Statistics (Angola).	151568

Country	Years	Name	Type	Geographic Detail	Male Circumcision	STI Symptoms	Had Intercourse (Women)	Partner Away	Condom Last Time	Multiple Partners in Year (Men)	Multiple Partners in Year (Women)	In Union	Citation	NID*
Angola	2015-16	Angola Demographic and Health Survey 2015-2016	Survey microdata	GPS	X	X	X	X	X	X	X	X	ICF International, Ministry of Health (Angola), National Institute of Statistics (Angola), United Nations Children's Fund (UNICEF). Angola Demographic and Health Survey 2015-2016. Fairfax, United States of America: ICF International, 2017.	218555
Benin	2001	Benin Demographic and Health Survey 2001	Survey microdata	GPS			X	X			X		National Institute of Statistics and Economic Analysis (INSAE) (Benin), ORC Macro. Benin Demographic and Health Survey 2001. Fairfax, United States of America: ICF International.	18950
Benin	2006	Benin Demographic and Health Survey 2006	Survey microdata	Admin 1	X	X	X	X	X	X		X	Macro International, Inc, National Institute of Statistics and Economic Analysis (INSAE) (Benin), National Program Against AIDS (PNLS) (Benin). Benin Demographic and Health Survey 2006. Fairfax, United States of America: ICF International.	18959
Benin	2011-12	Benin Demographic and Health	Survey microdata	GPS	X	X	X	X	X	X	X	X	ICF International, National Institute of Statistics and Economic Analysis (INSAE) (Benin), National Program Against AIDS (PNLS) (Benin). Benin Demographic and Health Survey	79839

Country	Years	Name	Type	Geographic Detail	Male Circumcision	STI Symptoms	Had Intercourse (Women)	Partner Away	Condom Last Time	Multiple Partners in Year (Men)	Multiple Partners in Year (Women)	In Union	Citation	NID*
		Survey 2011-2012											2011-2012. Fairfax, United States of America: ICF International, 2014.	
Benin	2012	Benin Integrated Modular Survey on Household Living Conditions 2011-2012	Survey microdata	Admin 1								X	Danish International Development Agency (DANIDA), German Society for International Cooperation (GIZ), National Institute of Statistics and Economic Analysis (INSAE) (Benin). Benin Integrated Modular Survey on Household Living Conditions 2011-2012. Cotonou, Benin: National Institute of Statistics and Economic Analysis (INSAE) (Benin).	151805
Benin	2014	Benin Multiple Indicator Cluster Survey 2014	Survey microdata	Admin 1	X		X		X	X	X	X	National Institute of Statistics and Economic Analysis (INSAE) (Benin), United Nations Children's Fund (UNICEF). Benin Multiple Indicator Cluster Survey 2014. New York, United States of America: United Nations Children's Fund (UNICEF), 2017.	206075
Benin	2017-18	Benin Demographic and Health	Survey microdata	GPS	X	X	X	X	X	X	X	X	Hubert Koutoukou Maga National University Hospital Center (CNHU-HKM) (Benin), ICF International, National Institute of Statistics and Economic Analysis (INSAE) (Benin),	218565

Country	Years	Name	Type	Geographic Detail	Male Circumcision	STI Symptoms	Had Intercourse (Women)	Partner Away	Condom Last Time	Multiple Partners in Year (Men)	Multiple Partners in Year (Women)	In Union	Citation	NID*
		Survey 2017-2018											National Malaria Control Program, Ministry of Health (Benin), Permanent Secretariat of the Food Council and Nutrition (SP-CAN) (Benin). Benin Demographic and Health Survey 2017-2018. Fairfax, United States of America: ICF International, 2018.	
Botswana	2000	Botswana Multiple Indicator Cluster Survey 2000	Survey microdata	Admin 1								X	Central Statistics Office (Botswana), United Nations Children's Fund (UNICEF). Botswana Multiple Indicator Cluster Survey 2000. New York, United States of America: United Nations Children's Fund (UNICEF), 2015.	1404
Botswana	2001	Botswana AIDS Impact Survey 2001	Survey microdata	Admin 2, Admin <1	X	X	X		X			X	Central Statistics Office (Botswana), Ministry of Health (Botswana). Botswana AIDS Impact Survey 2001. Gaborone, Botswana: Statistics Botswana.	22112
Botswana	2004	Botswana AIDS Impact Survey 2004	Survey microdata	GPS	X	X	X		X	X	X	X	Central Statistics Office (Botswana). Botswana AIDS Impact Survey 2004. Gaborone, Botswana: Statistics Botswana.	22114

Country	Years	Name	Type	Geographic Detail	Male Circumcision	STI Symptoms	Had Intercourse (Women)	Partner Away	Condom Last Time	Multiple Partners in Year (Men)	Multiple Partners in Year (Women)	In Union	Citation	NID*
Botswana	2007-08	Botswana Family Health Survey 2007-2008	Survey microdata	Admin 3, GPS			X					X	Central Statistics Office (Botswana). Botswana Family Health Survey 2007-2008. Gaborone, Botswana: Central Statistics Office (Botswana), 2009.	22125
Botswana	2008	Botswana AIDS Impact Survey 2008	Survey microdata	GPS	X	X	X	X	X	X	X	X	Central Statistics Office (Botswana), National AIDS Coordinating Agency (Botswana). Botswana AIDS Impact Survey 2008. Gaborone, Botswana: Statistics Botswana, 2015.	22116
Botswana	2010	Botswana Core Welfare Indicators Survey 2009-2010	Survey microdata	Admin 3, GPS								X	Central Statistics Office (Botswana). Botswana Core Welfare Indicators Survey 2009-2010.	151536
Botswana	2013	Botswana AIDS Impact Survey 2013	Survey microdata	GPS	X	X	X		X	X	X	X	Ministry of Health (Botswana), National AIDS Coordinating Agency (Botswana), Statistics Botswana. Botswana AIDS Impact Survey 2013. Gaborone, Botswana: Statistics Botswana, 2015.	134753
Burkina Faso	2003	Burkina Faso Core Welfare	Survey microdata	Admin 3, GPS								X	National Institute of Statistics and Demography (Burkina Faso), World Bank. Burkina Faso Core Welfare	1855

Country	Years	Name	Type	Geographic Detail	Male Circumcision	STI Symptoms	Had Intercourse (Women)	Partner Away	Condom Last Time	Multiple Partners in Year (Men)	Multiple Partners in Year (Women)	In Union	Citation	NID*
		Indicators Questionnaire Survey 2003											Indicators Questionnaire Survey 2003. Ouagadougou, Burkina Faso: National Institute of Statistics and Demography (Burkina Faso).	
Burkina Faso	2003	Burkina Faso Demographic and Health Survey 2003	Survey microdata	GPS	X	X	X	X	X	X	X	X	Macro International, Inc, National Institute of Statistics and Demography (Burkina Faso). Burkina Faso Demographic and Health Survey 2003. Fairfax, United States of America: ICF International.	19088
Burkina Faso	2006	Burkina Faso Multiple Indicator Cluster Survey 2006	Survey microdata	GPS			X				X		National Institute of Statistics and Demography (Burkina Faso), United Nations Children's Fund (UNICEF). Burkina Faso Multiple Indicator Cluster Survey 2006. New York, United States of America: United Nations Children's Fund (UNICEF).	1927
Burkina Faso	2007	Burkina Faso Core Welfare Indicators Questionnaire Survey 2007	Survey microdata	Admin 3, GPS								X	National Institute of Statistics and Demography (INSD). Burkina Faso Core Welfare Indicators Questionnaire Survey 2007. Ouagadougou, Burkina Faso: National Institute of Statistics and Demography (INSD), 2008.	18499

Country	Years	Name	Type	Geographic Detail	Male Circumcision	STI Symptoms	Had Intercourse (Women)	Partner Away	Condom Last Time	Multiple Partners in Year (Men)	Multiple Partners in Year (Women)	In Union	Citation	NID*
Burkina Faso	2010	Burkina Faso Demographic and Health Survey 2010-2011	Survey microdata	GPS	X	X	X	X	X	X	X	X	ICF Macro, Ministry of Health (Burkina Faso), National Institute of Statistics and Demography (Burkina Faso). Burkina Faso Demographic and Health Survey 2010-2011. Fairfax, United States of America: ICF International.	19133
Burkina Faso	2014	Burkina Faso Malaria Indicator Survey 2014	Survey microdata	GPS								X	ICF International, National Center for Research and Training on Malaria (CNRFP) (Burkina Faso), National Institute of Statistics and Demography (Burkina Faso), National Program for the Fight Against Malaria (PNLP) (Burkina Faso). Burkina Faso Malaria Indicator Survey 2014. Fairfax, United States of America: ICF International, 2015.	188785
Burkina Faso	2014	Burkina Faso Performance Monitoring and Accountability 2020	Survey microdata	Admin 1			X	X				X	Burkina Faso Institut national de la statistique et de la Démographie (National Institute of Statistics and Demography) and The Bill & Melinda Gates Institute for Population and Reproductive Health at The Johns Hopkins Bloomberg School of Public Health. Performance Monitoring and	257044

Country	Years	Name	Type	Geographic Detail	Male Circumcision	STI Symptoms	Had Intercourse (Women)	Partner Away	Condom Last Time	Multiple Partners in Year (Men)	Multiple Partners in Year (Women)	In Union	Citation	NID*
		Survey, Round 1 2014											Accountability 2020 (PMA2020) Survey round 1, PMA2014/Burkina Faso-R1. 2014. Ouagadougou, Burkina Faso and Baltimore, Maryland, USA.	
Burkina Faso	2015	Burkina Faso Performance Monitoring and Accountability 2020 Survey, Round 2 2015	Survey microdata	Admin 1			X	X				X	Burkina Faso Institut national de la statistique et de la Démographie (National Institute of Statistics and Demography), and The Bill & Melinda Gates Institute for Population and Reproductive Health at The Johns Hopkins Bloomberg School of Public Health. Performance Monitoring and Accountability 2020 (PMA2020) Survey round 2, PMA2015/Burkina Faso-R2. 2015. Ouagadougou, Burkina Faso and Baltimore, Maryland, USA.	257045
Burkina Faso	2016	Burkina Faso Performance Monitoring and Accountabi	Survey microdata	Admin 1			X	X				X	Burkina Faso Institut national de la statistique et de la Démographie (National Institute of Statistics and Demography), and The Bill & Melinda Gates Institute for Population and Reproductive Health at The Johns Hopkins Bloomberg School of Public	285993

Country	Years	Name	Type	Geographic Detail	Male Circumcision	STI Symptoms	Had Intercourse (Women)	Partner Away	Condom Last Time	Multiple Partners in Year (Men)	Multiple Partners in Year (Women)	In Union	Citation	NID*
		lity 2020 Survey, Round 3 2016											Health. Performance Monitoring and Accountability 2020 (PMA2020) Survey round 3, PMA2016/Burkina Faso-R3. 2016. Ouagadougou, Burkina Faso and Baltimore, Maryland, USA.	
Burkina Faso	2016	Burkina Faso Performan ce Monitoring and Accountabi lity 2020 Survey, Round 4 2016-2017	Survey microdata	Admin 1			X	X				X	Burkina Faso Institut national de la statistique et de la Démographie (National Institute of Statistics and Demography), and The Bill & Melinda Gates Institute for Population and Reproductive Health at The Johns Hopkins Bloomberg School of Public Health. Performance Monitoring and Accountability 2020 (PMA2020) Survey round 4, PMA2016/Burkina Faso-R4. 2016. Ouagadougou, Burkina Faso and Baltimore, Maryland, USA?.	307751
Burkina Faso	2018	Burkina Faso Performan ce Monitoring and	Survey microdata	Admin 1			X					X	Burkina Faso Institut national de la statistique et de la Démographie (National Institute of Statistics and Demography), and The Bill & Melinda Gates Institute for Population and Reproductive Health at The Johns	407829

Country	Years	Name	Type	Geographic Detail	Male Circumcision	STI Symptoms	Had Intercourse (Women)	Partner Away	Condom Last Time	Multiple Partners in Year (Men)	Multiple Partners in Year (Women)	In Union	Citation	NID*
		Accountability 2020 Survey, Round 6 2018-2019											Hopkins Bloomberg School of Public Health. Performance Monitoring and Accountability 2020 (PMA2020) Survey Round 6, PMA2018/Burkina Faso-R6. 2018. Ouagadougou, Burkina Faso and Baltimore, Maryland, USA?.	
Burundi	2000	Burundi Multiple Indicator Cluster Survey 2000	Survey microdata	Admin 1								X	Burundi Institute of Statistics and Economic Studies, United Nations Children's Fund (UNICEF). Burundi Multiple Indicator Cluster Survey 2000. New York, United States: United Nations Children's Fund (UNICEF).	1994
Burundi	2005	Burundi Multiple Indicator Cluster Survey 2005	Survey microdata	Admin 3			X		X	X			United Nations Children's Fund (UNICEF), Burundi Institute of Statistics and Economic Studies, United Nations Population Fund (UNFPA). Burundi Multiple Indicator Cluster Survey 2005. New York, United States: United Nations Children's Fund (UNICEF).	1981
Burundi	2010-11	Burundi Demographic and	Survey microdata	GPS	X	X	X		X	X	X	X	Burundi Institute of Statistics and Economic Studies, ICF International, Ministry of Public Health and the	30431

Country	Years	Name	Type	Geographic Detail	Male Circumcision	STI Symptoms	Had Intercourse (Women)	Partner Away	Condom Last Time	Multiple Partners in Year (Men)	Multiple Partners in Year (Women)	In Union	Citation	NID*
		Health Survey 2010-2011											Fight Against AIDS (Burundi). Burundi Demographic and Health Survey 2010-2011. Fairfax, United States of America: ICF International, 2012.	
Burundi	2016-17	Burundi Demographic and Health Survey 2016-2017	Survey microdata	GPS	X	X	X		X	X	X	X	Burundi Institute of Statistics and Economic Studies, ICF International, Ministry of Public Health and the Fight Against AIDS (Burundi). Burundi Demographic and Health Survey 2016-2017. Fairfax, United States of America: ICF International, 2018.	286766
Cameroon	2000	Cameroon Multiple Indicator Cluster Survey 2000	Survey microdata	Admin 1								X	Directorate of Statistics and National Accounts, Ministry of Economics and Finance (Cameroon), United Nations Children's Fund (UNICEF). Cameroon Multiple Indicator Cluster Survey 2000. New York, United States of America: United Nations Children's Fund (UNICEF).	2053
Cameroon	2001	Cameroon Household Survey 2001	Survey microdata	Admin 1, Admin 7								X	National Institute of Statistics (Cameroon), Directorate of Statistics and National Accounts, Ministry of Economics and Finance (Cameroon), AFRISTAT. Cameroon Household Survey 2001. Yaounde, Cameroon:	2039

Country	Years	Name	Type	Geographic Detail	Male Circumcision	STI Symptoms	Had Intercourse (Women)	Partner Away	Condom Last Time	Multiple Partners in Year (Men)	Multiple Partners in Year (Women)	In Union	Citation	NID*
													National Institute of Statistics (Cameroon).	
Cameroon	2004	Cameroon Demographic and Health Survey 2004	Survey microdata	GPS	X	X	X	X	X	X	X	X	Macro International, Inc, National Institute of Statistics (Cameroon). Cameroon Demographic and Health Survey 2004. Fairfax, United States of America: ICF International.	19211
Cameroon	2006	Cameroon Multiple Indicator Cluster Survey 2006	Survey microdata	Admin 1, Admin 3							X	X	United Nations Children's Fund (UNICEF), National Institute of Statistics (Cameroon). Cameroon Multiple Indicator Cluster Survey 2006. New York, United States: United Nations Children's Fund (UNICEF).	2063
Cameroon	2011	Cameroon Demographic and Health Survey 2011	Survey microdata	GPS	X	X	X	X	X	X	X	X	ICF International, Ministry of Economy, Planning and Regional Development (Cameroon), Ministry of Public Health (Cameroon), National Institute of Statistics (Cameroon), Pasteur Center of Cameroon. Cameroon Demographic and Health Survey 2011. Fairfax, United States of America: ICF International.	19274

Country	Years	Name	Type	Geographic Detail	Male Circumcision	STI Symptoms	Had Intercourse (Women)	Partner Away	Condom Last Time	Multiple Partners in Year (Men)	Multiple Partners in Year (Women)	In Union	Citation	NID*
Cameroon	2014	Cameroon Multiple Indicator Cluster Survey 2014	Survey microdata	Admin 1, Admin 3			X		X	X	X	X	Ministry of Public Health (Cameroon), National Institute of Statistics (Cameroon), United Nations Children's Fund (UNICEF). Cameroon Multiple Indicator Cluster Survey 2014. New York, United States of America: United Nations Children's Fund (UNICEF), 2017.	244455
Central African Republic	2006	Central African Republic Multiple Indicator Cluster Survey 2006	Survey microdata	Admin 1	X		X		X	X	X	X	United Nations Children's Fund (UNICEF). Central African Republic Multiple Indicator Cluster Survey 2006. New York, United States: United Nations Children's Fund (UNICEF).	2223
Central African Republic	2010	Central African Republic Multiple Indicator Cluster Survey 2010-2011	Survey microdata	Admin 1			X		X	X	X	X	Central African Institute of Statistics, Economic and Social Studies (ICASEES) (Central African Republic), ICF International. Central African Republic Multiple Indicator Cluster Survey 2010-2011. Fairfax, United States of America: ICF International, 2013.	82832

Country	Years	Name	Type	Geographic Detail	Male Circumcision	STI Symptoms	Had Intercourse (Women)	Partner Away	Condom Last Time	Multiple Partners in Year (Men)	Multiple Partners in Year (Women)	In Union	Citation	NID*
Chad	2000	Chad Multiple Indicator Cluster Survey 2000	Survey microdata	Admin 1								X	United Nations Children's Fund (UNICEF), Census Bureau (Chad), National Institute of Statistical, Economic and Demographic Studies (Chad). Chad Multiple Indicator Cluster Survey 2000. New York, United States: United Nations Children's Fund (UNICEF).	2244
Chad	2004	Chad Demographic and Health Survey 2004	Survey microdata	Admin 1	X	X	X	X	X	X	X	X	Macro International, Inc, National Institute for Statistics, Economic and Demographic Studies (INSEED) (Chad). Chad Demographic and Health Survey 2004. Fairfax, United States of America: ICF International.	19315
Chad	2010	Chad Multiple Indicator Cluster Survey 2010	Survey microdata	Admin 2			X				X		Ministry of Planning, Economy, and International Cooperation (Chad), National Institute for Statistics, Economic and Demographic Studies (INSEED) (Chad), United Nations Children's Fund (UNICEF). Chad Multiple Indicator Cluster Survey 2010. New York, United States of America: United Nations Children's Fund (UNICEF), 2014.	76701

Country	Years	Name	Type	Geographic Detail	Male Circumcision	STI Symptoms	Had Intercourse (Women)	Partner Away	Condom Last Time	Multiple Partners in Year (Men)	Multiple Partners in Year (Women)	In Union	Citation	NID*
Chad	2014-15	Chad Demographic and Health Survey 2014-2015	Survey microdata	GPS	X	X	X	X	X	X	X	X	ICF International, National Institute for Statistics, Economic and Demographic Studies (INSEED) (Chad). Chad Demographic and Health Survey 2014-2015. Fairfax, United States of America: ICF International, 2016.	157025
Comoros	2000	Comoros Multiple Indicator Cluster Survey 2000	Survey microdata	Admin 1								X	United Nations Development Programme (UNDP), United Nations Children's Fund (UNICEF). Comoros Multiple Indicator Cluster Survey 2000. New York, United States: United Nations Children's Fund (UNICEF).	3114
Comoros	2012	Comoros Demographic and Health Survey 2012-2013	Survey microdata	GPS	X	X	X	X	X	X	X	X	General Directorate of Statistics and Forecasting (Comoros), ICF International. Comoros Demographic and Health Survey 2012-2013. Fairfax, United States of America: ICF International.	76850
Congo	2005	Congo Demographic and Health	Survey microdata	Admin 1		X	X	X	X	X	X	X	Macro International, Inc, National Center for Statistics and Economic Studies (Congo, Rep.). Congo Demographic and Health Survey	19391

Country	Years	Name	Type	Geographic Detail	Male Circumcision	STI Symptoms	Had Intercourse (Women)	Partner Away	Condom Last Time	Multiple Partners in Year (Men)	Multiple Partners in Year (Women)	In Union	Citation	NID*
		Survey 2005											2005. Fairfax, United States of America: ICF International.	
Congo	2009	Congo AIDS Indicator Survey 2009	Survey microdata	Admin 1	X	X	X	X	X	X	X	X	ICF Macro, National Center for Statistics and Economic Studies (Congo, Rep.). Congo AIDS Indicator Survey 2009. Fairfax, United States of America: ICF International.	3133
Congo	2011	Congo Demographic and Health Survey 2011-2012	Survey microdata	Admin 1	X	X	X	X	X	X	X	X	ICF International, Ministry of Health (Congo, Rep.), National Center for Statistics and Economic Studies (Congo, Rep.). Congo Demographic and Health Survey 2011-2012. Fairfax, United States of America: ICF International.	56151
Congo	2014-15	Congo Multiple Indicator Cluster Survey 2014-2015	Survey microdata	Admin 1	X								National Institute of Statistics (INS) (Congo, Rep.), United Nations Children's Fund (UNICEF). Congo Multiple Indicator Cluster Survey 2014-2015. New York, United States of America: United Nations Children's Fund (UNICEF), 2018.	234733
Côte d'Ivoire	2000	Côte d'Ivoire Multiple Indicator	Survey microdata	Admin >1								X	National School for Statistics and Economics Applied (ENSEA), United Nations Children's Fund (UNICEF), United Nations Educational, Scientific	26444

Country	Years	Name	Type	Geographic Detail	Male Circumcision	STI Symptoms	Had Intercourse (Women)	Partner Away	Condom Last Time	Multiple Partners in Year (Men)	Multiple Partners in Year (Women)	In Union	Citation	NID*
		Cluster Survey 2000											and Cultural Organization (UNESCO). Côte d'Ivoire Multiple Indicator Cluster Survey 2000. New York, United States of America: United Nations Children's Fund (UNICEF).	
Côte d'Ivoire	2005	Côte d'Ivoire AIDS Indicator Survey 2005	Survey microdata	Admin >1	X	X	X	X	X	X	X	X	CDC Retro-CI, Ministry of the Fight Against AIDS (Côte d'Ivoire), National Institute of Statistics (Côte d'Ivoire), ORC Macro. Côte d'Ivoire AIDS Indicator Survey 2005. Fairfax, United States of America: ICF International.	56148
Côte d'Ivoire	2011-12	Côte d'Ivoire Demographic and Health Survey 2011-2012	Survey microdata	GPS	X	X	X	X	X	X	X	X	ICF International, Ministry of the Fight Against AIDS (Côte d'Ivoire), National Institute of Statistics (Côte d'Ivoire). Côte d'Ivoire Demographic and Health Survey 2011-2012. Fairfax, United States of America: ICF International.	18533
Côte d'Ivoire	2016	Côte d'Ivoire Multiple Indicator Cluster Survey 2016	Survey microdata	Admin >1			X		X	X	X	X	National Institute of Statistics (Côte d'Ivoire), United Nations Children's Fund (UNICEF). Côte d'Ivoire Multiple Indicator Cluster Survey 2016. New York, United States of America: United Nations Children's Fund (UNICEF), 2018.	218611

Country	Years	Name	Type	Geographic Detail	Male Circumcision	STI Symptoms	Had Intercourse (Women)	Partner Away	Condom Last Time	Multiple Partners in Year (Men)	Multiple Partners in Year (Women)	In Union	Citation	NID*
Côte d'Ivoire	2018	Côte d'Ivoire Performance Monitoring and Accountability 2020 Survey, Round 2 2018	Survey microdata	Admin 2			X					X	Institut National de la Statistique de la Côte d'Ivoire (INS-Côte d'Ivoire), La Direction de Coordination du Programme National de Santé de la Mère et de l'Enfant (DC-PNSME), and The Bill & Melinda Gates Institute for Population and Reproductive Health at The Johns Hopkins Bloomberg School of Public Health. Performance Monitoring and Accountability 2020 (PMA2020) Survey round 2, PMA2018/Cote d'Ivoire-R2. 2018. Abidjan, Cote d'Ivoire and Baltimore, Maryland, USA.	398717
Democratic Republic of the Congo	2001	Democratic Republic of the Congo Multiple Indicator Cluster Survey 2001	Survey microdata	Admin 1			X					X	Ministry of Planning and Reconstruction (Congo, DR), United Nations Children's Fund (UNICEF). Congo, DR Multiple Indicator Cluster Survey 2001. New York, United States: United Nations Children's Fund (UNICEF).	3161
Democratic Republic	2007	Democratic Republic of the Congo	Survey microdata	GPS	X	X	X	X	X	X	X	X	Macro International, Inc, Ministry of Planning (Congo, DR). Democratic Republic of the Congo Demographic	19381

Country	Years	Name	Type	Geographic Detail	Male Circumcision	STI Symptoms	Had Intercourse (Women)	Partner Away	Condom Last Time	Multiple Partners in Year (Men)	Multiple Partners in Year (Women)	In Union	Citation	NID*
of the Congo		Demographic and Health Survey 2007											and Health Survey 2007. Fairfax, United States of America: ICF International.	
Democratic Republic of the Congo	2010	Democratic Republic of the Congo Multiple Indicator Cluster Survey 2010	Survey microdata	GPS			X				X		National Statistical Institute (Congo, DR), Ministry of Planning (Congo, DR), United Nations Children's Fund (UNICEF). Congo, DR Multiple Indicator Cluster Survey 2010. New York, United States: United Nations Children's Fund (UNICEF).	26998
Democratic Republic of the Congo	2013	Democratic Republic of the Congo - Kinshasa Performance Monitoring and Accountability 2020 Survey,	Survey microdata	Admin 1			X	X					Tulane University School of Public Health, University of Kinshasa School of Public Health and The Bill & Melinda Gates Institute for Population and Reproductive Health at The Johns Hopkins Bloomberg School of Public Health. Performance Monitoring and Accountability 2020 (PMA2020) Survey round 1, PMA2013/DRC-R1 (Kinshasa). 2013. DRC and Baltimore, Maryland, USA.	257822

Country	Years	Name	Type	Geographic Detail	Male Circumcision	STI Symptoms	Had Intercourse (Women)	Partner Away	Condom Last Time	Multiple Partners in Year (Men)	Multiple Partners in Year (Women)	In Union	Citation	NID*
		Round 1 2013-2014												
Democratic Republic of the Congo	2013-14	Democratic Republic of the Congo Demographic and Health Survey 2013-2014	Survey microdata	GPS		X	X	X	X	X	X	X	ICF International, Ministry of Planning and Monitoring Implementation of the Revolution of Modernity (Congo, DR), Ministry of Public Health (Congo, DR), National Institute of Statistics (Congo, DR). Democratic Republic of the Congo Demographic and Health Survey 2013-2014. Fairfax, United States of America: ICF International, 2014.	76878
Democratic Republic of the Congo	2014	Democratic Republic of the Congo - Kinshasa Performance Monitoring and Accountability 2020 Survey, Round 2 2014	Survey microdata	GPS			X	X					Tulane University School of Public Health, University of Kinshasa School of Public Health and The Bill & Melinda Gates Institute for Population and Reproductive Health at The Johns Hopkins Bloomberg School of Public Health. Performance Monitoring and Accountability 2020 (PMA2020) Survey round 2, PMA2014/DRC-R2 (Kinshasa). 2014. Kinshasa, DRC and Baltimore, Maryland, USA.	257823

Country	Years	Name	Type	Geographic Detail	Male Circumcision	STI Symptoms	Had Intercourse (Women)	Partner Away	Condom Last Time	Multiple Partners in Year (Men)	Multiple Partners in Year (Women)	In Union	Citation	NID*
Democratic Republic of the Congo	2015	Democratic Republic of the Congo - Kinshasa Performance Monitoring and Accountability 2020 Survey, Round 3 2015	Survey microdata	GPS			X	X					Tulane University School of Public Health, University of Kinshasa School of Public Health and The Bill & Melinda Gates Institute for Population and Reproductive Health at The Johns Hopkins Bloomberg School of Public Health. Performance Monitoring and Accountability 2020 (PMA2020) Survey round 3, PMA2015/DRC-R3 (Kinshasa). 2015. Kinshasa, DRC and Baltimore, Maryland, USA.	257826
Democratic Republic of the Congo	2015	Democratic Republic of the Congo - Kinshasa and Kongo Central Performance Monitoring and Accountability 2020	Survey microdata	GPS			X	X					Tulane University School of Public Health, University of Kinshasa School of Public Health and The Bill & Melinda Gates Institute for Population and Reproductive Health at The Johns Hopkins Bloomberg School of Public Health. Performance Monitoring and Accountability 2020 (PMA2020) Survey round 4, PMA2015/DRC-R4 (Kinshasa & Kongo Central). 2015. Kinshasa, DRC and Baltimore, Maryland, USA.	286019

Country	Years	Name	Type	Geographic Detail	Male Circumcision	STI Symptoms	Had Intercourse (Women)	Partner Away	Condom Last Time	Multiple Partners in Year (Men)	Multiple Partners in Year (Women)	In Union	Citation	NID*
		Survey, Round 4 2015-2016												
Democratic Republic of the Congo	2016	Democratic Republic of the Congo - Kinshasa and Kongo Central Performance Monitoring and Accountability 2020 Survey, Round 5 2016	Survey microdata	GPS			X	X					Tulane University School of Public Health, University of Kinshasa School of Public Health and The Bill & Melinda Gates Institute for Population and Reproductive Health at The Johns Hopkins Bloomberg School of Public Health. Performance Monitoring and Accountability 2020 (PMA2020) Survey round 5, PMA2016/DRC-R5 (Kinshasa & Kongo Central). 2016. Kinshasa, DRC and Baltimore, Maryland, USA.	286054
Equatorial Guinea	2000	Equatorial Guinea Multiple Indicator Cluster Survey 2000	Survey microdata	Admin 1								X	Ministry of Planning, Economic Development and Public Investment (Equatorial Guinea), United Nations Children's Fund (UNICEF). Equatorial Guinea Multiple Indicator Cluster Survey 2000. New York, United	3655

Country	Years	Name	Type	Geographic Detail	Male Circumcision	STI Symptoms	Had Intercourse (Women)	Partner Away	Condom Last Time	Multiple Partners in Year (Men)	Multiple Partners in Year (Women)	In Union	Citation	NID*
													States of America: United Nations Children's Fund (UNICEF).	
Eritrea	2002	Eritrea Demographic and Health Survey 2002	Survey microdata	Admin 1			X	X				X	Macro International, Inc, National Statistics and Evaluation Office (Eritrea). Eritrea Demographic and Health Survey 2002. Fairfax, United States of America: ICF International.	19539
Eritrea	2010	Eritrea Population and Health Survey 2010	Survey report	Admin 1	X								Kenya Medical Research Institute (KEMRI), National Statistics Office (Eritrea), The Fafo Research Foundation. Eritrea Population and Health Survey 2010.	249999
Eswatini	2000	Swaziland Multiple Indicator Cluster Survey 2000	Survey microdata	Admin 1								X	Central Statistical Office (Swaziland), United Nations Children's Fund (UNICEF). Swaziland Multiple Indicator Cluster Survey 2000. New York, United States of America: United Nations Children's Fund (UNICEF).	12320
Eswatini	2006-07	Swaziland Demographic and Health	Survey microdata	GPS	X	X	X	X	X	X	X	X	Central Statistical Office (Swaziland), Macro International, Inc. Swaziland Demographic and Health Survey 2006-2007. Fairfax, United States of America: ICF International.	20829

Country	Years	Name	Type	Geographic Detail	Male Circumcision	STI Symptoms	Had Intercourse (Women)	Partner Away	Condom Last Time	Multiple Partners in Year (Men)	Multiple Partners in Year (Women)	In Union	Citation	NID*
		Survey 2006-2007												
Eswatini	2010	Swaziland Multiple Indicator Cluster Survey 2010	Survey microdata	Admin 1	X	X	X		X	X	X	X	Central Statistical Office (Swaziland), United Nations Children's Fund (UNICEF). Swaziland Multiple Indicator Cluster Survey 2010. New York, United States of America: United Nations Children's Fund (UNICEF).	30325
Eswatini	2014	Swaziland Multiple Indicator Cluster Survey 2014	Survey microdata	Admin 1	X		X		X	X	X	X	Central Statistical Office (Swaziland), United Nations Children's Fund (UNICEF), United Nations Educational, Scientific and Cultural Organization (UNESCO), United Nations Population Fund (UNFPA). Swaziland Multiple Indicator Cluster Survey 2014. New York, United States of America: United Nations Children's Fund (UNICEF), 2016.	200707
Eswatini	2017	Swaziland HIV Incidence Measurement Survey	Survey microdata	Admin 1	X		X		X	X	X	X	Centers for Disease Control and Prevention (CDC), Central Statistical Office (Swaziland), Environmental Health Department, Ministry of Health (Swaziland), Health Promotion Unit (Swaziland), ICAP, Columbia	415531

Country	Years	Name	Type	Geographic Detail	Male Circumcision	STI Symptoms	Had Intercourse (Women)	Partner Away	Condom Last Time	Multiple Partners in Year (Men)	Multiple Partners in Year (Women)	In Union	Citation	NID*
		2 2016-2017											University Mailman School of Public Health, Ministry of Health (Swaziland), National Emergency Response Council on HIV and AIDS (NERCHA) (Swaziland), Swaziland Health Laboratory Services, Ministry of Health (SHLS), Swaziland Health Research (SHR), Swaziland National AIDS Programme (SNAP). Swaziland HIV Incidence Measurement Survey 2 2016-2017.	
Ethiopia	2000	Ethiopia Demographic and Health Survey 2000	Survey microdata	GPS			X	X	X	X	X	X	Central Statistical Agency (Ethiopia), ORC Macro. Ethiopia Demographic and Health Survey 2000. Calverton, United States of America: ORC Macro, 2001.	19571
Ethiopia	2004	Ethiopia Rural Socioeconomic Survey 2011-2012	Survey microdata	GPS								X	Central Statistical Agency (Ethiopia), World Bank. Ethiopia Rural Socioeconomic Survey 2011-2012. Washington DC, United States of America: World Bank.	93848
Ethiopia	2005	Ethiopia Demographic and	Survey microdata	GPS	X	X	X	X	X	X	X	X	Macro International, Inc, Population and Housing Census Commissions Office (PHCCO). Ethiopia	19557

Country	Years	Name	Type	Geographic Detail	Male Circumcision	STI Symptoms	Had Intercourse (Women)	Partner Away	Condom Last Time	Multiple Partners in Year (Men)	Multiple Partners in Year (Women)	In Union	Citation	NID*
		Health Survey 2005											Demographic and Health Survey 2005. Fairfax, United States of America: ICF International.	
Ethiopia	2006-07	Ethiopia Socioeconomic Survey 2013-2014	Survey microdata	GPS								X	Central Statistical Agency (Ethiopia), World Bank. Ethiopia Socioeconomic Survey 2013-2014. Washington DC, United States of America: World Bank, 2015.	235215
Ethiopia	2008	Ethiopia Socioeconomic Survey 2015-2016	Survey microdata	GPS								X	Central Statistical Agency (Ethiopia), World Bank. Ethiopia Socioeconomic Survey 2015-2016. Washington DC, United States of America: World Bank, 2015.	286657
Ethiopia	2010-11	Ethiopia Demographic and Health Survey 2010-2011	Survey microdata	GPS	X	X	X	X	X	X	X	X	Central Statistical Agency (Ethiopia), ICF Macro, Ministry of Health (Ethiopia). Ethiopia Demographic and Health Survey 2010-2011. Fairfax, United States of America: ICF International.	21301
Ethiopia	2014	Ethiopia Performance Monitoring and Accountabi	Survey microdata	Admin 1			X	X				X	Addis Ababa University School of Public Health and The Bill & Melinda Gates Institute for Population and Reproductive Health at The Johns Hopkins Bloomberg School of Public Health. Performance Monitoring and	153503

Country	Years	Name	Type	Geographic Detail	Male Circumcision	STI Symptoms	Had Intercourse (Women)	Partner Away	Condom Last Time	Multiple Partners in Year (Men)	Multiple Partners in Year (Women)	In Union	Citation	NID*
		lity 2020 Survey, Round 1 2014											Accountability 2020 (PMA2020) Survey round 1, PMA2014/Ethiopia-R1. 2014. Ethiopia and Baltimore, Maryland, USA.	
Ethiopia	2014	Ethiopia Performance Monitoring and Accountability 2020 Survey, Round 2 2014	Survey microdata	Admin 1			X	X				X	Addis Ababa University School of Public Health and The Bill & Melinda Gates Institute for Population and Reproductive Health at The Johns Hopkins Bloomberg School of Public Health. Performance Monitoring and Accountability 2020 (PMA2020) Survey round 2, PMA2014/Ethiopia-R2. 2014. Ethiopia and Baltimore, Maryland, USA.	256175
Ethiopia	2015	Ethiopia Performance Monitoring and Accountability 2020 Survey, Round 3 2015	Survey microdata	Admin 1			X	X				X	Addis Ababa University School of Public Health and The Bill & Melinda Gates Institute for Population and Reproductive Health at The Johns Hopkins Bloomberg School of Public Health. Performance Monitoring and Accountability 2020 (PMA2020) Survey round 3, PMA2015/Ethiopia-R3. 2015. Ethiopia and Baltimore, Maryland, USA.	256176

Country	Years	Name	Type	Geographic Detail	Male Circumcision	STI Symptoms	Had Intercourse (Women)	Partner Away	Condom Last Time	Multiple Partners in Year (Men)	Multiple Partners in Year (Women)	In Union	Citation	NID*
Ethiopia	2016	Ethiopia Demographic and Health Survey 2016	Survey microdata	GPS	X	X	X	X	X	X	X	X	Central Statistical Agency (Ethiopia), ICF International. Ethiopia Demographic and Health Survey 2016. Fairfax, United States of America: ICF International, 2017.	218568
Ethiopia	2016	Ethiopia Performance Monitoring and Accountability 2020 Survey, Round 4 2016	Survey microdata	Admin 1			X	X				X	Addis Ababa University School of Public Health and The Bill & Melinda Gates Institute for Population and Reproductive Health at The Johns Hopkins Bloomberg School of Public Health. Performance Monitoring and Accountability 2020 (PMA2020) Survey round 4, PMA2016/Ethiopia-R4. 2016. Ethiopia and Baltimore, Maryland, USA.PMA2020) Survey round 3, PMA2016/Burkina Faso-R3. 2016. Ouagadougou, Burkina Faso and Baltimore, Maryland, USA.	285891
Ethiopia	2017	Ethiopia Performance Monitoring and Accountabi	Survey microdata	Admin 1			X	X				X	Addis Ababa University School of Public Health and The Bill & Melinda Gates Institute for Population and Reproductive Health at The Johns Hopkins Bloomberg School of Public Health. Performance Monitoring and	347050

Country	Years	Name	Type	Geographic Detail	Male Circumcision	STI Symptoms	Had Intercourse (Women)	Partner Away	Condom Last Time	Multiple Partners in Year (Men)	Multiple Partners in Year (Women)	In Union	Citation	NID*
		lity 2020 Survey, Round 5 2017											Accountability 2020 (PMA2020) Survey round 5, PMA2017/Ethiopia-R5. 2017. Ethiopia and Baltimore, Maryland, USA.	
Ethiopia	2018	Ethiopia Performance Monitoring and Accountability 2020 Survey, Round 6 2018	Survey microdata	GPS			X					X	Addis Ababa University School of Public Health and The Bill & Melinda Gates Institute for Population and Reproductive Health at The Johns Hopkins Bloomberg School of Public Health. Performance Monitoring and Accountability 2020 (PMA2020) Survey round 6, PMA2018/Ethiopia-R6. 2018. Ethiopia and Baltimore, Maryland, USA.	398720
Gabon	2000	Gabon Demographic and Health Survey 2000-2001	Survey microdata	Admin 2			X	X	X			X	General Directorate of Statistics and Economic Studies (Gabon), Macro International, Inc. Gabon Demographic and Health Survey 2000-2001. Fairfax, United States of America: ICF International.	19579
Gabon	2012	Gabon Demographic and Health	Survey microdata	GPS	X	X	X	X	X	X	X	X	General Directorate of Statistics (Gabon), ICF International, Ministry of Economy, Employment and Sustainable Development (Gabon), Ministry of Health (Gabon). Gabon	76706

Country	Years	Name	Type	Geographic Detail	Male Circumcision	STI Symptoms	Had Intercourse (Women)	Partner Away	Condom Last Time	Multiple Partners in Year (Men)	Multiple Partners in Year (Women)	In Union	Citation	NID*
		Survey 2012											Demographic and Health Survey 2012. Fairfax, United States of America: ICF International, 2013.	
Gambia	2000	Gambia Multiple Indicator Cluster Survey 2000	Survey microdata	Admin 1								X	Central Statistics Department (Gambia), United Nations Children's Fund (UNICEF). Gambia Multiple Indicator Cluster Survey 2000. New York, United States: United Nations Children's Fund (UNICEF).	3922
Gambia	2010	Gambia Multiple Indicator Cluster Survey 2010	Survey microdata	Admin 1			X				X		Gambia Bureau of Statistics (GBOS), United Nations Children's Fund (UNICEF). Gambia Multiple Indicator Cluster Survey 2010. New York, United States of America: United Nations Children's Fund (UNICEF), 2018.	91506
Gambia	2013	Gambia Demographic and Health Survey 2013	Survey microdata	Admin 2		X	X	X	X	X	X	X	Gambia Bureau of Statistics (GBOS), ICF International, Ministry of Health and Social Welfare (The Gambia). Gambia Demographic and Health Survey 2013. Fairfax, United States of America: ICF International, 2015.	77384
Gambia	2018	Gambia Multiple Indicator	Survey microdata	Admin 1			X		X			X	Gambia Bureau of Statistics (GBOS), United Nations Children's Fund (UNICEF). Gambia Multiple Indicator	424884

Country	Years	Name	Type	Geographic Detail	Male Circumcision	STI Symptoms	Had Intercourse (Women)	Partner Away	Condom Last Time	Multiple Partners in Year (Men)	Multiple Partners in Year (Women)	In Union	Citation	NID*
		Cluster Survey 2018											Cluster Survey 2018. New York, United States of America: United Nations Children's Fund (UNICEF), 2019.	
Ghana	2003	Ghana Demographic and Health Survey 2003	Survey microdata	GPS	X	X	X	X	X	X	X	X	Ghana Statistical Service, Macro International, Inc. Ghana Demographic and Health Survey 2003. Fairfax, United States of America: ICF International.	19627
Ghana	2006	Ghana Multiple Indicator Cluster Survey 2006	Survey microdata	Admin 1			X		X	X	X	X	Ministry of Health (MOH) (Ghana), Ghana Statistical Service and United Nations Children's Fund (UNICEF). Ghana Multiple Indicator Cluster Survey 2006. New York, United States: United Nations Children's Fund (UNICEF).	4694
Ghana	2008	Ghana Demographic and Health Survey 2008	Survey microdata	GPS	X	X	X	X	X	X	X	X	Ghana Statistical Service, Macro International, Inc, Ministry of Health (Ghana). Ghana Demographic and Health Survey 2008. Fairfax, United States of America: ICF International.	21188
Ghana	2010	Ghana - Accra	Survey microdata	GPS			X		X	X	X	X	Institute of Statistical, Social and Economic Research, University of	56241

Country	Years	Name	Type	Geographic Detail	Male Circumcision	STI Symptoms	Had Intercourse (Women)	Partner Away	Condom Last Time	Multiple Partners in Year (Men)	Multiple Partners in Year (Women)	In Union	Citation	NID*
		Multiple Indicator Cluster Survey 2010-2011											Ghana, United Nations Children's Fund (UNICEF). Ghana - Accra Multiple Indicator Cluster Survey 2010-2011. New York, United States of America: United Nations Children's Fund (UNICEF), 2014.	
Ghana	2011	Ghana Multiple Indicator Cluster Survey 2011	Survey microdata	GPS			X		X	X	X	X	Centers for Disease Control and Prevention (CDC), Ghana Statistical Service, Government of Japan, ICF Macro, Ministry of Health (Ghana), Navrongo Health Research Centre, United Nations Children's Fund (UNICEF), United Nations Population Fund (UNFPA), United States Agency for International Development (USAID). Ghana Multiple Indicator Cluster Survey 2011. New York, United States of America: United Nations Children's Fund (UNICEF), 2013.	63993
Ghana	2013	Ghana Living Standards Measurem	Survey microdata	Admin 1								X	Ghana Statistical Service, World Bank. Ghana Living Standards Measurement Survey 2012-2013. Accra, Ghana: Ghana Statistical Service.	165101

Country	Years	Name	Type	Geographic Detail	Male Circumcision	STI Symptoms	Had Intercourse (Women)	Partner Away	Condom Last Time	Multiple Partners in Year (Men)	Multiple Partners in Year (Women)	In Union	Citation	NID*
		ent Survey 2012-2013												
Ghana	2013	Ghana Performance Monitoring and Accountability 2020 Survey, Round 1 2013	Survey microdata	Admin 1			X	X				X	Kwame Nkrumah University of Science & Technology School of Medicine and The Bill & Melinda Gates Institute for Population and Reproductive Health at The Johns Hopkins Bloomberg School of Public Health. Performance Monitoring and Accountability 2020 (PMA2020) Survey round 1, PMA2013/Ghana-R1. 2013. Ghana and Baltimore, Maryland, USA.	197904
Ghana	2014	Ghana Demographic and Health Survey 2014	Survey microdata	GPS	X	X	X	X	X	X	X	X	Ghana Health Service, Ghana Statistical Service, ICF International. Ghana Demographic and Health Survey 2014. Fairfax, United States of America: ICF International, 2016.	157027
Ghana	2014	Ghana Performance Monitoring and Accountabi	Survey microdata	Admin 1			X	X				X	Kwame Nkrumah University of Science & Technology School of Medicine and The Bill & Melinda Gates Institute for Population and Reproductive Health at The Johns Hopkins Bloomberg School of Public	256241

Country	Years	Name	Type	Geographic Detail	Male Circumcision	STI Symptoms	Had Intercourse (Women)	Partner Away	Condom Last Time	Multiple Partners in Year (Men)	Multiple Partners in Year (Women)	In Union	Citation	NID*
		lity 2020 Survey, Round 2 2014											Health. Performance Monitoring and Accountability 2020 (PMA2020) Survey round 2, PMA2014/Ghana-R2. 2014. Ghana and Baltimore, Maryland, USA.	
Ghana	2014	Ghana Performance Monitoring and Accountability 2020 Survey, Round 3 2014	Survey microdata	Admin 1			X	X				X	Kwame Nkrumah University of Science & Technology School of Medicine and The Bill & Melinda Gates Institute for Population and Reproductive Health at The Johns Hopkins Bloomberg School of Public Health. Performance Monitoring and Accountability 2020 (PMA2020) Survey round 3, PMA2014/Ghana-R3. 2014. Ghana and Baltimore, Maryland, USA.	256243
Ghana	2015	Ghana Performance Monitoring and Accountability 2020 Survey,	Survey microdata	Admin 1			X	X				X	Bill and Melinda Gates Institute for Population and Reproductive Health, Johns Hopkins Bloomberg School of Public Health, Ghana Health Service, Ghana Statistical Service, Kwame Nkrumah University of Science and Technology (KNUST), University for Development Studies (Ghana). Ghana Performance Monitoring and	256244

Country	Years	Name	Type	Geographic Detail	Male Circumcision	STI Symptoms	Had Intercourse (Women)	Partner Away	Condom Last Time	Multiple Partners in Year (Men)	Multiple Partners in Year (Women)	In Union	Citation	NID*
		Round 4 2015											Accountability 2020 Survey, Round 4 2015. Baltimore, United States of America: Bill and Melinda Gates Institute for Population and Reproductive Health, Johns Hopkins Bloomberg School of Public Health.	
Ghana	2016	Ghana Performance Monitoring and Accountability 2020 Survey, Round 5 2016	Survey microdata	Admin 1			X	X				X	Kwame Nkrumah University of Science & Technology School of Medicine and The Bill & Melinda Gates Institute for Population and Reproductive Health at The Johns Hopkins Bloomberg School of Public Health. Performance Monitoring and Accountability 2020 (PMA2020) Survey round 5, PMA2016/Ghana-R5. 2016. Ghana and Baltimore, Maryland, USA.	286146
Ghana	2017	Ghana Special Demographic and Health Survey 2017	Survey microdata	GPS				X					Ghana Health Service, Ghana Statistical Service, ICF International. Ghana Special Demographic and Health Survey 2017. Fairfax, United States of America: ICF International, 2018.	218572

Country	Years	Name	Type	Geographic Detail	Male Circumcision	STI Symptoms	Had Intercourse (Women)	Partner Away	Condom Last Time	Multiple Partners in Year (Men)	Multiple Partners in Year (Women)	In Union	Citation	NID*
Guinea	2005	Guinea Demographic and Health Survey 2005	Survey microdata	GPS	X	X	X	X	X	X	X	X	Macro International, Inc, National Statistics Directorate (Guinea). Guinea Demographic and Health Survey 2005. Fairfax, United States of America: ICF International.	19683
Guinea	2012	Guinea Demographic and Health Survey 2012	Survey microdata	GPS		X	X	X	X	X	X	X	ICF Macro, Ministry of Health and Public Hygiene (Guinea), National Institute of Statistics (Guinea). Guinea Demographic and Health Survey 2012. Fairfax, United States of America: ICF International.	69761
Guinea	2016	Guinea Multiple Indicator Cluster Survey 2016	Survey microdata	Admin 1			X				X		National Institute of Public Health (NPHI) (Guinea), National Institute of Statistics (Guinea), National Malaria Control Program (Guinea), United Nations Children's Fund (UNICEF). Guinea Multiple Indicator Cluster Survey 2016. New York, United States of America: United Nations Children's Fund (UNICEF), 2018.	303458
Guinea	2018	Guinea Demographic and Health	Survey microdata	GPS		X	X	X	X	X	X	X	Ministry of Health (Guinea), Ministry of Planning and Economic Development (Guinea), National Institute of Statistics (Guinea).	396957

Country	Years	Name	Type	Geographic Detail	Male Circumcision	STI Symptoms	Had Intercourse (Women)	Partner Away	Condom Last Time	Multiple Partners in Year (Men)	Multiple Partners in Year (Women)	In Union	Citation	NID*
		Survey 2018											Guinea Demographic and Health Survey 2018. Fairfax, United States of America: ICF International, 2019.	
Guinea-Bissau	2006	Guinea-Bissau Multiple Indicator Cluster Survey 2006	Survey microdata	Admin 1			X				X		United Nations Children's Fund (UNICEF), Government of Guinea-Bissau. Guinea-Bissau Multiple Indicator Cluster Survey 2006. New York, United States: United Nations Children's Fund (UNICEF).	4818
Guinea-Bissau	2014	Guinea-Bissau Multiple Indicator Cluster Survey 2014	Survey microdata	Admin 1	X		X		X	X	X	X	National Statistics Institute (Guinea-Bissau), United Nations Children's Fund (UNICEF). Guinea-Bissau Multiple Indicator Cluster Survey 2014. New York, United States of America: United Nations Children's Fund (UNICEF), 2016.	174049
Kenya	2000	Kenya Multiple Indicator Cluster Survey 2000	Survey microdata	GPS								X	Central Bureau of Statistics (Kenya), United Nations Children's Fund (UNICEF). Kenya Multiple Indicator Cluster Survey 2000. New York, United States: United Nations Children's Fund (UNICEF).	7387
Kenya	2003	Kenya Demograp	Survey microdata	GPS	X	X	X	X	X	X	X	X	Centers for Disease Control and Prevention (CDC), Central Bureau of	20145

Country	Years	Name	Type	Geographic Detail	Male Circumcision	STI Symptoms	Had Intercourse (Women)	Partner Away	Condom Last Time	Multiple Partners in Year (Men)	Multiple Partners in Year (Women)	In Union	Citation	NID*
		hic and Health Survey 2003											Statistics (Kenya), Macro International, Inc, Ministry of Health (Kenya), National Council for Population and Development (Kenya). Kenya Demographic and Health Survey 2003. Fairfax, United States of America: ICF International.	
Kenya	2007	Kenya AIDS Indicator Survey 2007	Survey microdata	GPS	X		X	X	X	X	X	X	Centers for Disease Control and Prevention (CDC), Kenya Medical Research Institute (KEMRI), Kenya National Bureau of Statistics, Ministry of Public Health and Sanitation (Kenya), National AIDS Control Council (Kenya), National AIDS and STI Control Programme (NASCOP) (Kenya), National Coordinating Agency for Population and Development (Kenya), National Public Health Laboratory Services, Ministry of Public Health and Sanitation (Kenya), United States Agency for International Development (USAID). Kenya AIDS Indicator Survey 2007. Nairobi,	133219

Country	Years	Name	Type	Geographic Detail	Male Circumcision	STI Symptoms	Had Intercourse (Women)	Partner Away	Condom Last Time	Multiple Partners in Year (Men)	Multiple Partners in Year (Women)	In Union	Citation	NID*
													Kenya: Kenya National Bureau of Statistics.	
Kenya	2007	Kenya Household Health Expenditure and Utilization Survey 2007	Survey microdata	Admin 2								X	Abt Associates Inc., Kenya National Bureau of Statistics, Ministry of Health (Kenya). Kenya Household Health Expenditure and Utilization Survey 2007. Nairobi, Kenya: Kenya National Bureau of Statistics.	157635
Kenya	2008-09	Kenya Demographic and Health Survey 2008-2009	Survey microdata	GPS	X	X	X	X	X	X	X	X	ICF Macro, Kenya Medical Research Institute (KEMRI), Kenya National Bureau of Statistics, Ministry of Public Health and Sanitation (Kenya), National AIDS and STI Control Programme (NASCOP) (Kenya), National Aids Control Council (NACC), National Coordinating Agency for Population and Development (Kenya). Kenya Demographic and Health Survey 2008-2009. Fairfax, United States of America: ICF International.	21365
Kenya	2009	Kenya - Coast	Survey microdata	Admin 3			X				X		Kenya National Bureau of Statistics, United Nations Children's Fund	56420

Country	Years	Name	Type	Geographic Detail	Male Circumcision	STI Symptoms	Had Intercourse (Women)	Partner Away	Condom Last Time	Multiple Partners in Year (Men)	Multiple Partners in Year (Women)	In Union	Citation	NID*
		Multiple Indicator Cluster Survey 2009											(UNICEF). Kenya - Coast Multiple Indicator Cluster Survey 2009. New York, United States of America: United Nations Children's Fund (UNICEF), 2014.	
Kenya	2011	Kenya - Nyanza Province Multiple Indicator Cluster Survey 2011	Survey microdata	GPS			X				X		Kenya National Bureau of Statistics, United Nations Children's Fund (UNICEF). Kenya - Nyanza Province Multiple Indicator Cluster Survey 2011. Nairobi, Kenya: Kenya National Bureau of Statistics.	135416
Kenya	2013	Kenya AIDS Indicator Survey 2012-2013	Survey microdata	GPS	X	X	X	X		X	X	X	Kenya National Bureau of Statistics, Ministry of Devolution and Planning (Kenya), Ministry of Health (Kenya), National AIDS and STI Control Programme (NASCOP) (Kenya). Kenya AIDS Indicator Survey 2012-2013. Nairobi, Kenya: Kenya National Bureau of Statistics.	133304
Kenya	2013-14	Kenya - Bungoma County Multiple	Survey microdata	GPS			X						Kenya National Bureau of Statistics, Population Studies and Research Institute, University of Nairobi (Kenya), United Nations Children's	203654

Country	Years	Name	Type	Geographic Detail	Male Circumcision	STI Symptoms	Had Intercourse (Women)	Partner Away	Condom Last Time	Multiple Partners in Year (Men)	Multiple Partners in Year (Women)	In Union	Citation	NID*
		Indicator Survey 2013-2014											Fund (UNICEF). Kenya - Bungoma County Multiple Indicator Survey 2013-2014. New York, United States of America: United Nations Children's Fund (UNICEF), 2015.	
Kenya	2013-14	Kenya - Kakamega County Multiple Indicator Survey 2013-2014	Survey microdata	GPS			X				X		Kenya National Bureau of Statistics, Population Studies and Research Institute, University of Nairobi (Kenya), United Nations Children's Fund (UNICEF). Kenya - Kakamega County Multiple Indicator Survey 2013-2014. New York, United States of America: United Nations Children's Fund (UNICEF), 2015.	203663
Kenya	2013-14	Kenya - Turkana County Multiple Indicator Survey 2013-2014	Survey microdata	GPS			X				X		Kenya National Bureau of Statistics, Population Studies and Research Institute, University of Nairobi (Kenya), United Nations Children's Fund (UNICEF). Kenya - Turkana County Multiple Indicator Survey 2013-2014. New York, United States of America: United Nations Children's Fund (UNICEF), 2015.	203664
Kenya	2014	Kenya Demograp	Survey microdata	GPS	X	X	X	X	X	X	X	X	ICF International, Kenya Medical Research Institute (KEMRI), Kenya	157057

Country	Years	Name	Type	Geographic Detail	Male Circumcision	STI Symptoms	Had Intercourse (Women)	Partner Away	Condom Last Time	Multiple Partners in Year (Men)	Multiple Partners in Year (Women)	In Union	Citation	NID*
		hic and Health Survey 2014											National Bureau of Statistics, Ministry of Health (Kenya), National AIDS Control Council (Kenya), National Council for Population and Development (Kenya). Kenya Demographic and Health Survey 2014. Fairfax, United States of America: ICF International.	
Kenya	2014	Kenya Performance Monitoring and Accountability 2020 Survey, Round 1 2014	Survey microdata	Admin 2			X	X				X	International Centre for Reproductive Health Kenya (ICRHK) and The Bill & Melinda Gates Institute for Population and Reproductive Health at The Johns Hopkins Bloomberg School of Public Health. Performance Monitoring and Accountability 2020 (PMA2020) Survey round 1, PMA2014/Kenya-R1. 2014. Kenya and Baltimore, Maryland, USA.	197910
Kenya	2014	Kenya Performance Monitoring and Accountabi	Survey microdata	Admin 2			X	X				X	Bill and Melinda Gates Institute for Population and Reproductive Health, Johns Hopkins Bloomberg School of Public Health, International Center for Reproductive Health (Kenya), Kenya National Bureau of Statistics,	256338

Country	Years	Name	Type	Geographic Detail	Male Circumcision	STI Symptoms	Had Intercourse (Women)	Partner Away	Condom Last Time	Multiple Partners in Year (Men)	Multiple Partners in Year (Women)	In Union	Citation	NID*
		lity 2020 Survey, Round 2 2014											Ministry of Health (Kenya), National Council for Population and Development (Kenya). Kenya Performance Monitoring and Accountability 2020 Survey, Round 2 2014. Baltimore, United States of America: Johns Hopkins Bloomberg School of Public Health.	
Kenya	2015	Kenya Performance Monitoring and Accountability 2020 Survey, Round 3 2015	Survey microdata	Admin 2			X	X				X	International Centre for Reproductive Health Kenya (ICRHK) and The Bill & Melinda Gates Institute for Population and Reproductive Health at The Johns Hopkins Bloomberg School of Public Health. Performance Monitoring and Accountability 2020 (PMA2020) Survey round 3, PMA2015/Kenya-R3. 2015. Kenya and Baltimore, Maryland, USA.	256365
Kenya	2015	Kenya Performance Monitoring and Accountabi	Survey microdata	Admin 2			X	X				X	International Centre for Reproductive Health Kenya (ICRHK) and The Bill & Melinda Gates Institute for Population and Reproductive Health at The Johns Hopkins Bloomberg School of Public	256366

Country	Years	Name	Type	Geographic Detail	Male Circumcision	STI Symptoms	Had Intercourse (Women)	Partner Away	Condom Last Time	Multiple Partners in Year (Men)	Multiple Partners in Year (Women)	In Union	Citation	NID*
		lity 2020 Survey, Round 4 2015											Health. Performance Monitoring and Accountability 2020 (PMA2020) Survey round 4, PMA2015/Kenya-R4. 2015. Kenya and Baltimore, Maryland, USA.	
Kenya	2016	Kenya Performan ce Monitoring and Accountabi lity 2020 Survey, Round 5 2016	Survey microdata	Admin 2			X	X				X	International Centre for Reproductive Health Kenya (ICRHK) and The Bill & Melinda Gates Institute for Population and Reproductive Health at The Johns Hopkins Bloomberg School of Public Health. Performance Monitoring and Accountability 2020 (PMA2020) Survey round 5, PMA2016/Kenya-R5. 2016. Kenya and Baltimore, Maryland, USA.	347047
Kenya	2017	Kenya Performan ce Monitoring and Accountabi lity 2020 Survey,	Survey microdata	Admin 1			X						International Centre for Reproductive Health Kenya (ICRHK) and The Bill & Melinda Gates Institute for Population and Reproductive Health at The Johns Hopkins Bloomberg School of Public Health. Performance Monitoring and Accountability 2020 (PMA2020) Survey round 5, PMA2016/Kenya-R5.	374949

Country	Years	Name	Type	Geographic Detail	Male Circumcision	STI Symptoms	Had Intercourse (Women)	Partner Away	Condom Last Time	Multiple Partners in Year (Men)	Multiple Partners in Year (Women)	In Union	Citation	NID*
		Round 6 2017											2016. Kenya and Baltimore, Maryland, USA.	
Kenya	2018	Kenya Performance Monitoring and Accountability 2020 Survey, Round 7 2018	Survey microdata	Admin 1			X					X	International Centre for Reproductive Health Kenya (ICRHK) and The Bill & Melinda Gates Institute for Population and Reproductive Health at The Johns Hopkins Bloomberg School of Public Health. Performance Monitoring and Accountability 2020 (PMA2020) Survey Round 7, PMA2018/Kenya-R7. 2018. Kenya and Baltimore, Maryland, USA.	407837
Lesotho	2000	Lesotho Multiple Indicator Cluster Survey 2000	Survey microdata	Admin 1								X	Bureau of Statistics (Lesotho), United Nations Children's Fund (UNICEF). Lesotho Multiple Indicator Cluster Survey 2000. New York, United States of America: United Nations Children's Fund (UNICEF).	7721
Lesotho	2004-05	Lesotho Demographic and Health Survey 2004-2005	Survey microdata	GPS	X	X	X	X		X	X	X	Bureau of Statistics (Lesotho), Macro International, Inc, Ministry of Health and Social Welfare (Lesotho). Lesotho Demographic and Health Survey 2004-2005. Fairfax, United States of America: ICF International.	20167

Country	Years	Name	Type	Geographic Detail	Male Circumcision	STI Symptoms	Had Intercourse (Women)	Partner Away	Condom Last Time	Multiple Partners in Year (Men)	Multiple Partners in Year (Women)	In Union	Citation	NID*
Lesotho	2009-10	Lesotho Demographic and Health Survey 2009-2010	Survey microdata	GPS	X	X	X	X	X	X	X	X	ICF Macro, Ministry of Health and Social Welfare (Lesotho). Lesotho Demographic and Health Survey 2009-2010. Fairfax, United States of America: ICF International.	21382
Lesotho	2014	Lesotho Demographic and Health Survey 2014	Survey microdata	GPS	X	X	X	X	X	X	X	X	ICF International, Ministry of Health and Social Welfare (Lesotho). Lesotho Demographic and Health Survey 2014. Fairfax, United States of America: ICF International.	157058
Lesotho	2018	Lesotho Multiple Indicator Cluster Survey 2018	Survey microdata	Admin 1								X	Bureau of Statistics (Lesotho), United Nations Children's Fund (UNICEF). Lesotho Multiple Indicator Cluster Survey 2018. New York, United States of America: United Nations Children's Fund (UNICEF), 2019.	427778
Liberia	2006-07	Liberia Demographic and Health Survey 2006-2007	Survey microdata	GPS	X	X	X	X	X	X	X	X	Liberia Institute for Statistics and Geo-information Services (LISGIS), Macro International, Inc. Liberia Demographic and Health Survey 2006-2007. Fairfax, United States of America: ICF International.	20191

Country	Years	Name	Type	Geographic Detail	Male Circumcision	STI Symptoms	Had Intercourse (Women)	Partner Away	Condom Last Time	Multiple Partners in Year (Men)	Multiple Partners in Year (Women)	In Union	Citation	NID*
Liberia	2013	Liberia Demographic and Health Survey 2013	Survey microdata	GPS	X	X	X	X	X	X	X	X	ICF International, Liberia Institute for Statistics and Geo-information Services (LISGIS), National AIDS and STI Control Program (NACP), Ministry of Health and Social Welfare (Liberia). Liberia Demographic and Health Survey 2013. Fairfax, United States of America: ICF International.	77385
Madagascar	2000	Madagascar Multiple Indicator Cluster Survey 2000	Survey microdata	Admin 1			X						National Institute of Statistics (Madagascar), United Nations Children's Fund (UNICEF). Madagascar Multiple Indicator Cluster Survey 2000. New York, United States of America: United Nations Children's Fund (UNICEF).	27020
Madagascar	2003-04	Madagascar Demographic and Health Survey 2003-2004	Survey microdata	Admin 2	X	X	X	X	X	X	X	X	Macro International, Inc, National Institute of Statistics (Madagascar). Madagascar Demographic and Health Survey 2003-2004. Fairfax, United States of America: ICF International.	20223
Madagascar	2008-09	Madagascar Demograp	Survey microdata	GPS	X	X	X	X	X	X	X	X	ICF Macro, National Institute of Statistics (Madagascar). Madagascar Demographic and Health Survey	21409

Country	Years	Name	Type	Geographic Detail	Male Circumcision	STI Symptoms	Had Intercourse (Women)	Partner Away	Condom Last Time	Multiple Partners in Year (Men)	Multiple Partners in Year (Women)	In Union	Citation	NID*
		hic and Health Survey 2008-2009											2008-2009. Fairfax, United States of America: ICF International.	
Madagascar	2012	Madagascar - South Multiple Indicator Cluster Survey 2012	Survey microdata	GPS			X				X		National Institute of Statistics (Madagascar), United Nations Children's Fund (UNICEF). Madagascar - South Multiple Indicator Cluster Survey 2012. New York, United States of America: United Nations Children's Fund (UNICEF), 2015.	125594
Madagascar	2018	Madagascar Multiple Indicator Cluster Survey 2018	Survey microdata	GPS			X		X			X	National Institute of Statistics (Madagascar), United Nations Children's Fund (UNICEF). Madagascar Multiple Indicator Cluster Survey 2018. 2019.	399853
Malawi	2000	Malawi Demographic and Health Survey 2000	Survey microdata	GPS		X	X	X	X	X	X	X	Macro International, Inc, National Statistical Office of Malawi. Malawi Demographic and Health Survey 2000. Fairfax, United States of America: ICF International.	20252

Country	Years	Name	Type	Geographic Detail	Male Circumcision	STI Symptoms	Had Intercourse (Women)	Partner Away	Condom Last Time	Multiple Partners in Year (Men)	Multiple Partners in Year (Women)	In Union	Citation	NID*
Malawi	2004-05	Malawi Demographic and Health Survey 2004-2005	Survey microdata	GPS	X	X	X	X	X	X	X	X	Macro International, Inc, National Statistical Office of Malawi. Malawi Demographic and Health Survey 2004-2005. Fairfax, United States of America: ICF International.	20263
Malawi	2006	Malawi Multiple Indicator Cluster Survey 2006	Survey microdata	Admin 2			X		X	X	X	X	United Nations Children's Fund (UNICEF), National Statistics Office (Malawi). Malawi Multiple Indicator Cluster Survey 2006. New York, United States: United Nations Children's Fund (UNICEF).	7919
Malawi	2010	Malawi Demographic and Health Survey 2010	Survey microdata	GPS	X	X	X	X	X	X	X	X	ICF Macro, National Statistical Office of Malawi. Malawi Demographic and Health Survey 2010. Fairfax, United States of America: ICF International.	21393
Malawi	2013-14	Malawi Multiple Indicator Cluster Survey 2013-2014	Survey microdata	Admin 2	X		X		X	X	X	X	National Statistical Office of Malawi, United Nations Children's Fund (UNICEF). Malawi Multiple Indicator Cluster Survey 2013-2014. New York, United States of America: United Nations Children's Fund (UNICEF), 2015.	161662

Country	Years	Name	Type	Geographic Detail	Male Circumcision	STI Symptoms	Had Intercourse (Women)	Partner Away	Condom Last Time	Multiple Partners in Year (Men)	Multiple Partners in Year (Women)	In Union	Citation	NID*
Malawi	2015-16	Malawi Demographic and Health Survey 2015-2016	Survey microdata	GPS	X	X	X	X	X	X	X	X	Emory University and Centers for Disease Control & Prevention Collaboration, ICF International, Ministry of Health (Malawi), National Statistical Office of Malawi. Malawi Demographic and Health Survey 2015-2016. Fairfax, United States of America: ICF International, 2017.	218581
Malawi	2015-16	Malawi Population-based HIV Impact Assessment 2015-2016	Survey microdata	Admin >1	X	X	X	X	X	X	X	X	Ministry of Health, Malawi, Centers for Disease Control and Prevention (CDC), and ICAP at Columbia University. Malawi Population-based HIV Impact Assessment (MPHIA) 2015-16: First Report. Lilongwe, Malawi, Atlanta, Georgia and New York, New York, USA: Ministry of Health, CDC and ICAP. December 2016.	287629
Malawi	2016-17	Malawi Integrated Household Survey 2016-2017	Survey microdata	Admin 2								X	National Statistical Office. Malawi - Fourth Integrated Household Survey 2016-2017, Ref. MWI_2016_IHS-IV_v02_M. Dataset downloaded from [http://microdata.worldbank.org/index.php/catalog/2936] on [December 18, 2017].	327852

Country	Years	Name	Type	Geographic Detail	Male Circumcision	STI Symptoms	Had Intercourse (Women)	Partner Away	Condom Last Time	Multiple Partners in Year (Men)	Multiple Partners in Year (Women)	In Union	Citation	NID*
Mali	2001	Mali Demographic and Health Survey 2001	Survey microdata	GPS		X	X	X	X	X	X	X	Macro International, Inc, National Directorate of Statistics and Informatics (DNSI) (Mali), Planning and Statistics Unit, Ministry of Health (Mali). Mali Demographic and Health Survey 2001. Fairfax, United States of America: ICF International.	20315
Mali	2006	Mali Demographic and Health Survey 2006	Survey microdata	GPS	X	X	X	X	X	X	X	X	Macro International, Inc, Ministry of Health (Mali), National Directorate of Statistics and Informatics (DNSI) (Mali). Mali Demographic and Health Survey 2006. Fairfax, United States of America: ICF International.	20274
Mali	2009-10	Mali Multiple Indicator Cluster Survey 2009-2010	Survey microdata	Admin 2			X				X		Ministry of Health (Mali), National Institute of Statistics (INSTAT) (Mali), United Nations Children's Fund (UNICEF). Mali Multiple Indicator Cluster Survey 2009-2010. New York, United States of America: United Nations Children's Fund (UNICEF), 2017.	270627
Mali	2012-13	Mali Demographic and Health	Survey microdata	GPS	X	X	X	X	X	X	X	X	ICF International, INFO-STAT (Mali), Ministry of Health (Mali), National Institute of Statistics (INSTAT) (Mali), Planning and Statistics Unit, Ministry	77388

Country	Years	Name	Type	Geographic Detail	Male Circumcision	STI Symptoms	Had Intercourse (Women)	Partner Away	Condom Last Time	Multiple Partners in Year (Men)	Multiple Partners in Year (Women)	In Union	Citation	NID*
		Survey 2012-2013											of Health (Mali). Mali Demographic and Health Survey 2012-2013. Fairfax, United States of America: ICF International, 2014.	
Mali	2015	Mali Malaria Indicator Survey 2015	Survey microdata	GPS								X	ICF International, INFO-STAT (Mali), Ministry of Health and Public Hygiene (Mali), National Institute of Public Health Research (INRSP) (Mali), National Institute of Statistics (INSTAT) (Mali), National Program for the Fight Against Malaria (Mali). Mali Malaria Indicator Survey 2015. Fairfax, United States of America: ICF International, 2016.	218587
Mali	2015	Mali Multiple Indicator Cluster Survey 2015	Survey microdata	Admin 1			X		X	X	X	X	Ministry of Health (Mali), Ministry of Planning (Mali), National Institute of Statistics (INSTAT) (Mali), United Nations Children's Fund (UNICEF). Mali Multiple Indicator Cluster Survey 2015. New York, United States of America: United Nations Children's Fund (UNICEF), 2017.	248224
Mali	2018	Mali Demographic and	Survey microdata	GPS	X	X	X	X	X	X	X	X	ICF International, National Institute of Statistics (INSTAT) (Mali). Mali Demographic and Health Survey	398033

Country	Years	Name	Type	Geographic Detail	Male Circumcision	STI Symptoms	Had Intercourse (Women)	Partner Away	Condom Last Time	Multiple Partners in Year (Men)	Multiple Partners in Year (Women)	In Union	Citation	NID*
		Health Survey 2018											2018. Fairfax, United States of America: ICF International, 2019.	
Mauritania	2000-01	Mauritania Demographic and Health Survey 2000-2001	Survey microdata	Admin 1			X	X		X		X	Macro International, Inc, National Office of Statistics (Mauritania). Mauritania Demographic and Health Survey 2000-2001. Fairfax, United States of America: ICF International.	20322
Mauritania	2011	Mauritania Multiple Indicator Cluster Survey 2011	Survey microdata	Admin 3								X	National Office of Statistics (Mauritania), United Nations Children's Fund (UNICEF). Mauritania Multiple Indicator Cluster Survey 2011. New York, United States of America: United Nations Children's Fund (UNICEF), 2015.	152783
Mauritania	2015	Mauritania Multiple Indicator Cluster Survey 2015	Survey microdata	Admin 1								X	National Office of Statistics (Mauritania), United Nations Children's Fund (UNICEF). Mauritania Multiple Indicator Cluster Survey 2015. New York, United States of America: United Nations Children's Fund (UNICEF), 2018.	267343
Mozambique	2001	Mozambique Young	Survey microdata	Admin 1			X						Mozambique National Institute of Statistics, Division of Reproductive	27519

Country	Years	Name	Type	Geographic Detail	Male Circumcision	STI Symptoms	Had Intercourse (Women)	Partner Away	Condom Last Time	Multiple Partners in Year (Men)	Multiple Partners in Year (Women)	In Union	Citation	NID*
		Adult Reproductive Health Survey 2001											Health-Centers for Disease Control and Prevention (CDC). (2003) Mozambique Young Adult Reproductive Health Survey 2001. Atlanta, United States: Centers for Disease Control and Prevention (CDC).	
Mozambique	2003	Mozambique Demographic and Health Survey 2003-2004	Survey microdata	Admin 1	X	X	X	X	X	X	X	X	Macro International, Inc, National Institute of Statistics (INE) (Mozambique). Mozambique Demographic and Health Survey 2003-2004. Fairfax, United States of America: ICF International.	20394
Mozambique	2008	Mozambique Multiple Indicator Cluster Survey 2008-2009	Survey microdata	Admin 5, GPS			X						United Nations Children's Fund (UNICEF), National Statistics Institute (Mozambique). Mozambique Multiple Indicator Cluster Survey 2008-2009. New York, United States: United Nations Children's Fund (UNICEF).	27031
Mozambique	2009	Mozambique AIDS Indicator	Survey microdata	GPS	X	X	X	X	X	X	X	X	ICF Macro, Ministry of Health (Mozambique), National Institute of Statistics (INE) (Mozambique). Mozambique AIDS Indicator Survey	8906

Country	Years	Name	Type	Geographic Detail	Male Circumcision	STI Symptoms	Had Intercourse (Women)	Partner Away	Condom Last Time	Multiple Partners in Year (Men)	Multiple Partners in Year (Women)	In Union	Citation	NID*
		Survey 2009											2009. Fairfax, United States of America: ICF International, 2010.	
Mozambique	2011	Mozambique Demographic and Health Survey 2011	Survey microdata	GPS	X	X	X	X	X	X	X	X	ICF Macro, Manhica Health Research Center (CISM), Ministry of Health (Mozambique), National Institute of Statistics (INE) (Mozambique). Mozambique Demographic and Health Survey 2011. Fairfax, United States of America: ICF International.	55975
Mozambique	2015	Mozambique AIDS Indicator Survey 2015	Survey microdata	GPS	X	X	X	X	X	X	X	X	Centers for Disease Control and Prevention (CDC), ICF International, Ministry of Health (Mozambique), National Institute of Health (Mozambique), National Institute of Statistics (INE) (Mozambique). Mozambique AIDS Indicator Survey 2015. Fairfax, United States of America: ICF International, 2018.	157060
Namibia	2000	Namibia Demographic and Health Survey 2000	Survey microdata	GPS			X	X	X	X	X	X	Macro International, Inc, Ministry of Health and Social Services (Namibia), National Planning Commission (Namibia). Namibia Demographic and Health Survey 2000. Fairfax, United States of America: ICF International.	20417

Country	Years	Name	Type	Geographic Detail	Male Circumcision	STI Symptoms	Had Intercourse (Women)	Partner Away	Condom Last Time	Multiple Partners in Year (Men)	Multiple Partners in Year (Women)	In Union	Citation	NID*
Namibia	2006-07	Namibia Demographic and Health Survey 2006-2007	Survey microdata	GPS	X	X	X	X	X	X	X	X	Macro International, Inc, Ministry of Health and Social Services (Namibia). Namibia Demographic and Health Survey 2006-2007. Fairfax, United States of America: ICF International.	20428
Namibia	2013	Namibia Demographic and Health Survey 2013	Survey microdata	GPS	X	X	X	X	X	X	X	X	ICF International, Ministry of Health and Social Services (Namibia), Namibia Institute of Pathology, Namibia Statistics Agency. Namibia Demographic and Health Survey 2013. Fairfax, United States of America: ICF International.	150382
Niger	2000	Niger Multiple Indicator Cluster Survey 2000	Survey microdata	Admin 1								X	Government of Niger, Macro International, Inc, United Nations Children's Fund (UNICEF). Niger Multiple Indicator Cluster Survey 2000. New York, United States of America: United Nations Children's Fund (UNICEF).	9439
Niger	2006	Niger Demographic and Health	Survey microdata	Admin 1	X	X	X	X	X	X	X	X	Department of Statistics and National Accounts (Niger), Macro International, Inc. Niger Demographic and Health Survey 2006. Fairfax,	20499

Country	Years	Name	Type	Geographic Detail	Male Circumcision	STI Symptoms	Had Intercourse (Women)	Partner Away	Condom Last Time	Multiple Partners in Year (Men)	Multiple Partners in Year (Women)	In Union	Citation	NID*
		Survey 2006											United States of America: ICF International.	
Niger	2012	Niger Demographic and Health Survey 2012	Survey microdata	Admin 1		X	X	X	X	X	X	X	ICF International, Ministry of Public Health (Niger), National Institute of Statistics (Niger). Niger Demographic and Health Survey 2012. Fairfax, United States of America: ICF International.	74393
Niger	2015	Niger - Niamey Performance Monitoring and Accountability 2020 Survey, Round 1 2015	Survey microdata	Admin 1			X	X				X	Niger/Niamey Institut National de la Statistique (National Institute of Statistics) and The Bill & Melinda Gates Institute for Population and Reproductive Health at The Johns Hopkins Bloomberg School of Public Health. Performance Monitoring and Accountability 2020 (PMA2020) Survey round 1, PMA2015/Niger-R1 (Niamey). 2015. Niamey, Niger and Baltimore, Maryland, USA.	256177
Niger	2016	Niger Performance Monitoring and Accountabi	Survey microdata	Admin 1			X	X				X	Niger/Niamey Institut National de la Statistique (National Institute of Statistics) and The Bill & Melinda Gates Institute for Population and Reproductive Health at The Johns Hopkins Bloomberg School of Public	286052

Country	Years	Name	Type	Geographic Detail	Male Circumcision	STI Symptoms	Had Intercourse (Women)	Partner Away	Condom Last Time	Multiple Partners in Year (Men)	Multiple Partners in Year (Women)	In Union	Citation	NID*
		lity 2020 Survey, Round 2 2016											Health. Performance Monitoring and Accountability 2020 (PMA2020) Survey round 2, PMA2016/Niger-R2 (National). 2016. Niamey, Niger and Baltimore, Maryland, USA.	
Niger	2018	Niger - Niamey Performan ce Monitoring and Accountabi lity 2020 Survey, Round 5 2018	Survey microdata	GPS			X					X	Niger/Niamey Institut National de la Statistique (National Institute of Statistics) and The Bill & Melinda Gates Institute for Population and Reproductive Health at The Johns Hopkins Bloomberg School of Public Health. Performance Monitoring and Accountability 2020 (PMA2020) Survey round 5, PMA2018/Niger-R5 (Niamey). 2018. Niamey, Niger and Baltimore, Maryland, USA.	398719
Nigeria	2003	Nigeria Demograp hic and Health Survey 2003	Survey microdata	GPS		X	X	X	X	X	X	X	Department for International Development (DFiD) (United Kingdom), National Population Commission of Nigeria, ORC Macro, United Nations Children's Fund (UNICEF), United Nations Population Fund (UNFPA). Nigeria Demographic and Health Survey 2003. Fairfax,	20567

Country	Years	Name	Type	Geographic Detail	Male Circumcision	STI Symptoms	Had Intercourse (Women)	Partner Away	Condom Last Time	Multiple Partners in Year (Men)	Multiple Partners in Year (Women)	In Union	Citation	NID*
													United States of America: ICF International.	
Nigeria	2004	Nigeria Living Standards Survey 2003-2004	Survey microdata	Admin 1								X	Federal Office of Statistics (Nigeria). Nigeria Living Standards Survey 2003-2004.	25006
Nigeria	2006	Nigeria Core Welfare Indicators Questionnaire Survey 2006	Survey microdata	Admin 1	X							X	National Bureau of Statistics (Nigeria). Nigeria Core Welfare Indicators Questionnaire Survey 2006. Abuja, Nigeria: National Bureau of Statistics (Nigeria).	9522
Nigeria	2007	Nigeria Multiple Indicator Cluster Survey 2007	Survey microdata	Admin 1			X				X		United Nations Children's Fund (UNICEF), National Bureau of Statistics (Nigeria). Nigeria Multiple Indicator Cluster Survey 2007. New York, United States: United Nations Children's Fund (UNICEF).	9516
Nigeria	2007	Nigeria National HIV/AIDS and Reproducti	Survey microdata	Admin 1		X	X			X	X	X	Federal Ministry of Health (Nigeria), National Agency for the Control of AIDS (NACA) (Nigeria), National Bureau of Statistics (Nigeria), National Population Commission	325046

Country	Years	Name	Type	Geographic Detail	Male Circumcision	STI Symptoms	Had Intercourse (Women)	Partner Away	Condom Last Time	Multiple Partners in Year (Men)	Multiple Partners in Year (Women)	In Union	Citation	NID*
		ve Health Survey 2007											(NPC), Nigerian Institute of Medical Research, Society for Family Health (Nigeria), University College Hospital, Ibadan. Nigeria National HIV/AIDS and Reproductive Health Survey 2007.	
Nigeria	2008	Nigeria Demographic and Health Survey 2008	Survey microdata	GPS	X	X	X	X	X	X	X	X	Macro International, Inc, National Population Commission of Nigeria. Nigeria Demographic and Health Survey 2008. Fairfax, United States of America: ICF International, 2009.	21433
Nigeria	2008	Nigeria General Household Survey 2008 - IPUMS	Survey microdata	Admin 1								X	National Bureau of Statistics (Nigeria), Minnesota Population Center. Nigeria General Household Survey 2008 from the Integrated Public Use Microdata Series, International: [Machine-readable database]. Minneapolis: University of Minnesota.	151313
Nigeria	2009	Nigeria General Household Survey	Survey microdata	Admin 1								X	National Bureau of Statistics (Nigeria), Minnesota Population Center. Nigeria General Household Survey 2009 from the Integrated Public Use Microdata Series,	151314

Country	Years	Name	Type	Geographic Detail	Male Circumcision	STI Symptoms	Had Intercourse (Women)	Partner Away	Condom Last Time	Multiple Partners in Year (Men)	Multiple Partners in Year (Women)	In Union	Citation	NID*
		2009 - IPUMS											International: [Machine-readable database]. Minneapolis: University of Minnesota.	
Nigeria	2009	Nigeria Living Standards Survey 2008-2010	Survey microdata	Admin 1, Admin 2								X	National Bureau of Statistics (Nigeria). Nigeria Living Standards Survey 2008-2010. Abuja, Nigeria: National Bureau of Statistics (Nigeria).	151719
Nigeria	2010	Nigeria General Household Survey 2010 - IPUMS	Survey microdata	Admin 2								X	National Bureau of Statistics (Nigeria), Minnesota Population Center. Nigeria General Household Survey 2010 from the Integrated Public Use Microdata Series, International: [Machine-readable database]. Minneapolis: University of Minnesota.	151315
Nigeria	2011	Nigeria General Household Survey 2010-2011 - IPUMS	Survey microdata	Admin 1								X	National Bureau of Statistics (Nigeria), Minnesota Population Center. Nigeria General Household Survey 2010-2011 from the Integrated Public Use Microdata Series, International: [Machine-readable database]. Minneapolis: University of Minnesota.	151317

Country	Years	Name	Type	Geographic Detail	Male Circumcision	STI Symptoms	Had Intercourse (Women)	Partner Away	Condom Last Time	Multiple Partners in Year (Men)	Multiple Partners in Year (Women)	In Union	Citation	NID*
Nigeria	2011	Nigeria Multiple Indicator Cluster Survey 2011	Survey microdata	Admin 1			X				X		National Bureau of Statistics (Nigeria), United Nations Children's Fund (UNICEF). Nigeria Multiple Indicator Cluster Survey 2011. New York, United States of America: United Nations Children's Fund (UNICEF), 2013.	76703
Nigeria	2012	Nigeria National HIV/AIDS and Reproductive Health Survey 2012	Survey microdata	Admin 2		X	X			X	X	X	Expanded Social Marketing Project in Nigeria (ESMPIN), Federal Ministry of Health (Nigeria), Joint United Nations Program on HIV/AIDS (UNAIDS), National Population Commission (NPC), Society for Family Health (Nigeria), University College Hospital, Ibadan, World Health Organization (WHO). Nigeria National HIV/AIDS and Reproductive Health Survey 2012.	324443
Nigeria	2013	Nigeria Demographic and Health Survey 2013	Survey microdata	GPS	X	X	X	X	X	X	X	X	ICF International, National Population Commission of Nigeria. Nigeria Demographic and Health Survey 2013. Fairfax, United States of America: ICF International.	77390

Country	Years	Name	Type	Geographic Detail	Male Circumcision	STI Symptoms	Had Intercourse (Women)	Partner Away	Condom Last Time	Multiple Partners in Year (Men)	Multiple Partners in Year (Women)	In Union	Citation	NID*
Nigeria	2014	Nigeria - Kaduna and Lagos Performance Monitoring and Accountability 2020 Survey, Round 1 2014	Survey microdata	Admin 1			X	X				X	Centre for Population and Reproductive Health (CPRH), University of Ibadan; Centre for Research, Evaluation Resources and Development (CRERD); Population and Reproductive Health Program (PRHP), Obafemi Awolowo University (OAU); Bayero University Kano (BUK); and The Bill & Melinda Gates Institute for Population and Reproductive Health at The Johns Hopkins Bloomberg School of Public Health. Performance Monitoring and Accountability 2020 (PMA2020) Survey round 1, PMA2014/Nigeria-R1 (Kaduna & Lagos). 2014. Nigeria and Baltimore, Maryland, USA.	256263
Nigeria	2015	Nigeria - Kaduna and Lagos Performance Monitoring and Accountabi	Survey microdata	Admin 1			X	X				X	Centre for Research, Evaluation Resources and Development (CRERD), Bayero University Kano (BUK), and The Bill & Melinda Gates Institute for Population and Reproductive Health at The Johns Hopkins Bloomberg School of Public Health. Performance Monitoring and	256268

Country	Years	Name	Type	Geographic Detail	Male Circumcision	STI Symptoms	Had Intercourse (Women)	Partner Away	Condom Last Time	Multiple Partners in Year (Men)	Multiple Partners in Year (Women)	In Union	Citation	NID*
		lity 2020 Survey, Round 2 2015											Accountability 2020 (PMA2020) Survey round 2, PMA2015/Nigeria-R2 (Kaduna & Lagos). 2015. Nigeria and Baltimore, Maryland, USA	
Nigeria	2016	Nigeria Performance Monitoring and Accountability 2020 Survey, Round 3 2016	Survey microdata	Admin 1			X					X	Centre for Research, Evaluation Resources and Development (CRERD), Bayero University Kano (BUK), and The Bill & Melinda Gates Institute for Population and Reproductive Health at The Johns Hopkins Bloomberg School of Public Health. Performance Monitoring and Accountability 2020 (PMA2020) Survey round 3, PMA2016/Nigeria-R3 (National). 2016. Nigeria and Baltimore, Maryland, USA.	286022
Nigeria	2016-17	Nigeria Multiple Indicator Cluster Survey with National Immunization	Survey microdata	GPS	X		X		X	X	X	X	National Agency for the Control of AIDS (Nigeria), National Bureau of Statistics (Nigeria), National Primary Health Care Development Agency (NPHCDA) (Nigeria), United Nations Children's Fund (UNICEF). Nigeria Multiple Indicator Cluster Survey with National Immunization Coverage Survey Supplement 2016-	218613

Country	Years	Name	Type	Geographic Detail	Male Circumcision	STI Symptoms	Had Intercourse (Women)	Partner Away	Condom Last Time	Multiple Partners in Year (Men)	Multiple Partners in Year (Women)	In Union	Citation	NID*
		Coverage Survey Supplement 2016-2017											2017. New York, United States of America: United Nations Children's Fund (UNICEF), 2018.	
Nigeria	2018	Nigeria Demographic and Health Survey 2018	Survey microdata	GPS	X	X	X	X	X	X	X	X	Federal Ministry of Health (Nigeria), ICF International, National Population Commission (NPC). Nigeria Demographic and Health Survey 2018. Fairfax, United States of America: ICF International, 2019.	408484
Nigeria	2018	Nigeria Performance Monitoring and Accountability 2020 Survey, Round 5 2018	Survey microdata	Admin 1			X					X	Centre for Research, Evaluation Resources and Development (CRERD), Bayero University Kano (BUK), and The Bill & Melinda Gates Institute for Population and Reproductive Health at The Johns Hopkins Bloomberg School of Public Health. Performance Monitoring and Accountability 2020 (PMA2020) Survey round 5, PMA2018/Nigeria-R5 (National). 2018. Nigeria and Baltimore, Maryland, USA.	394679
Rwanda	2000	Rwanda Demograp	Survey microdata	Admin 1		X	X		X	X	X	X	Macro International, Inc, National Office of Population (Rwanda).	20722

Country	Years	Name	Type	Geographic Detail	Male Circumcision	STI Symptoms	Had Intercourse (Women)	Partner Away	Condom Last Time	Multiple Partners in Year (Men)	Multiple Partners in Year (Women)	In Union	Citation	NID*
		hic and Health Survey 2000											Rwanda Demographic and Health Survey 2000. Fairfax, United States of America: ICF International.	
Rwanda	2000	Rwanda Multiple Indicator Cluster Survey 2000	Survey microdata	Admin 1								X	Department of Statistics (Rwanda), United Nations Children's Fund (UNICEF). Rwanda Multiple Indicator Cluster Survey 2000. New York, United States: United Nations Children's Fund (UNICEF).	26930
Rwanda	2005	Rwanda Demograp hic and Health Survey 2005	Survey microdata	GPS	X	X	X		X	X	X	X	Macro International, Inc, National Institute of Statistics of Rwanda. Rwanda Demographic and Health Survey 2005. Fairfax, United States of America: ICF International.	20740
Rwanda	2007-08	Rwanda Interim Demograp hic and Health Survey 2007-2008	Survey microdata	GPS	X							X	Macro International, Inc, Ministry of Health (Rwanda), National Institute of Statistics of Rwanda. Rwanda Interim Demographic and Health Survey 2007-2008. Fairfax, United States of America: ICF International.	21222
Rwanda	2010-11	Rwanda Demograp	Survey microdata	GPS	X	X	X		X	X	X	X	ICF Macro, Ministry of Health (Rwanda), National Institute of	56040

Country	Years	Name	Type	Geographic Detail	Male Circumcision	STI Symptoms	Had Intercourse (Women)	Partner Away	Condom Last Time	Multiple Partners in Year (Men)	Multiple Partners in Year (Women)	In Union	Citation	NID*
		hic and Health Survey 2010-2011											Statistics of Rwanda. Rwanda Demographic and Health Survey 2010-2011. Fairfax, United States of America: ICF International.	
Rwanda	2011	Rwanda Integrated Household Living Conditions Survey 2010-2011	Survey microdata	Admin 1								X	National Institute of Statistics of Rwanda. Rwanda Integrated Household Living Conditions Survey 2010-2011. Kigali, Rwanda: National Institute of Statistics of Rwanda.	151437
Rwanda	2011	Rwanda Special Demographic and Health Survey 2011	Survey microdata	Admin 1								X	ICF International, Joint United Nations Program on HIV/AIDS (UNAIDS), National Institute of Statistics of Rwanda, Rwanda Biomedical Center/Institute of HIV/AIDS, Disease Control and Prevention Department, School of Public Health, University of Rwanda. Rwanda Special Demographic and Health Survey 2011. Fairfax, United States of America: ICF International, 2012.	56426
Rwanda	2014-15	Rwanda Demograp	Survey microdata	GPS	X	X	X		X	X	X	X	ICF International, Ministry of Health (Rwanda), National Institute of	157063

Country	Years	Name	Type	Geographic Detail	Male Circumcision	STI Symptoms	Had Intercourse (Women)	Partner Away	Condom Last Time	Multiple Partners in Year (Men)	Multiple Partners in Year (Women)	In Union	Citation	NID*
		hic and Health Survey 2014-2015											Statistics of Rwanda. Rwanda Demographic and Health Survey 2014-2015. Fairfax, United States of America: ICF International, 2016.	
Rwanda	2017	Rwanda Malaria Indicator Survey 2017	Survey microdata	Admin 2								X	ICF International, Ministry of Health (Rwanda), Rwanda Biomedical Center (RBC). Rwanda Malaria Indicator Survey 2017. Fairfax, United States of America: ICF International, 2018.	350836
Sao Tome and Principe	2000	Sao Tome and Principe Multiple Indicator Cluster Survey 2000	Survey microdata	Admin 1								X	National Institute of Statistics (Sao Tome and Principe), United Nations Children's Fund (UNICEF). Sao Tome and Principe Multiple Indicator Cluster Survey 2000. New York, United States of America: United Nations Children's Fund (UNICEF).	27055
Sao Tome and Principe	2008	Sao Tome and Principe Demographic and Health Survey 2008-2009	Survey microdata	Admin 2		X	X	X	X	X	X	X	ICF Macro, Ministry of Health (Sao Tome and Principe), National Institute of Statistics (Sao Tome and Principe). Sao Tome and Principe Demographic and Health Survey 2008-2009. Fairfax, United States of America: ICF International.	26866

Country	Years	Name	Type	Geographic Detail	Male Circumcision	STI Symptoms	Had Intercourse (Women)	Partner Away	Condom Last Time	Multiple Partners in Year (Men)	Multiple Partners in Year (Women)	In Union	Citation	NID*
Sao Tome and Principe	2014	Sao Tome and Principe Multiple Indicator Cluster Survey 2014	Survey microdata	Admin 2	X		X		X	X	X	X	Global Fund to Fight Aids Tuberculosis and Malaria (GFATM), ICF International, National Center for Endemic Diseases (CNE) (Sao Tome and Principe), National Institute of Statistics (Sao Tome and Principe), United Nations Children's Fund (UNICEF), United Nations Development Programme (UNDP). Sao Tome and Principe Multiple Indicator Cluster Survey 2014. New York, United States of America: United Nations Children's Fund (UNICEF), 2016.	214640
Senegal	2000	Senegal Multiple Indicator Cluster Survey 2000	Survey microdata	Admin 1								X	Directorate of Forecasting and Statistics, Ministry of the Economy, Finance and Planning (Senegal), Ministry of Economy and Finance (Senegal), United Nations Children's Fund (UNICEF). Senegal Multiple Indicator Cluster Survey 2000. New York, United States of America: United Nations Children's Fund (UNICEF).	27044

Country	Years	Name	Type	Geographic Detail	Male Circumcision	STI Symptoms	Had Intercourse (Women)	Partner Away	Condom Last Time	Multiple Partners in Year (Men)	Multiple Partners in Year (Women)	In Union	Citation	NID*
Senegal	2005	Senegal Demographic and Health Survey 2005	Survey microdata	GPS	X	X	X	X	X	X	X	X	Ministry of Health and Prevention (Senegal), Research Center for Human Development (Senegal). Senegal Demographic and Health Survey 2005. Fairfax, United States of America: ICF International.	26855
Senegal	2010-11	Senegal Demographic and Health Survey 2010-2011	Survey microdata	GPS		X	X	X	X	X	X	X	Center for Research in Human Development (CRDH), Cheikh Anta Diop University, Hospital Aristide Le Dantec, ICF Macro, National Agency of Statistics and Demography (Senegal). Senegal Demographic and Health Survey 2010-2011. Fairfax, United States of America: ICF International.	56063
Senegal	2012-13	Senegal Continuous Demographic and Health Survey 2012-2013	Survey microdata	GPS			X	X			X	X	ICF International, Ministry of Health and Social Action (Senegal), National Agency of Statistics and Demography (Senegal). Senegal Continuous Demographic and Health Survey 2012-2013. Fairfax, United States of America: ICF International.	111432
Senegal	2014	Senegal Continuous Demograp	Survey microdata	GPS		X	X	X	X	X	X	X	Cheikh Anta Diop University, ICF International, National Agency of Statistics and Demography (Senegal).	191270

Country	Years	Name	Type	Geographic Detail	Male Circumcision	STI Symptoms	Had Intercourse (Women)	Partner Away	Condom Last Time	Multiple Partners in Year (Men)	Multiple Partners in Year (Women)	In Union	Citation	NID*
		hic and Health Survey 2014											Senegal Continuous Demographic and Health Survey 2014. Fairfax, United States of America: ICF International.	
Senegal	2015	Senegal - Dakar Urban Multiple Indicator Cluster Survey 2015-2016	Survey microdata	Admin 2			X		X	X	X	X	National Agency of Statistics and Demography (Senegal), United Nations Children's Fund (UNICEF). Senegal - Dakar Urban Multiple Indicator Cluster Survey 2015-2016. New York, United States of America: United Nations Children's Fund (UNICEF), 2018.	287639
Senegal	2015	Senegal Continuous Demographic and Health Survey 2015	Survey microdata	GPS		X	X	X	X	X	X	X	Cheikh Anta Diop University, ICF International, National Agency of Statistics and Demography (Senegal). Senegal Continuous Demographic and Health Survey 2015. Fairfax, United States of America: ICF International, 2016.	218592
Senegal	2015	Senegal Global Adult Tobacco Survey 2015	Survey microdata	Admin 1								X	Centers for Disease Control and Prevention (CDC), Johns Hopkins Bloomberg School of Public Health, Ministry of Health and Social Action (Senegal), National Agency of Statistics and Demography (Senegal),	237375

Country	Years	Name	Type	Geographic Detail	Male Circumcision	STI Symptoms	Had Intercourse (Women)	Partner Away	Condom Last Time	Multiple Partners in Year (Men)	Multiple Partners in Year (Women)	In Union	Citation	NID*
													Research Triangle Institute, Inc. (RTI), World Health Organization (WHO). Senegal Global Adult Tobacco Survey 2015.	
Senegal	2016	Senegal Continuous Demographic and Health Survey 2016	Survey microdata	GPS		X	X	X	X	X	X	X	ICF International, Ministry of Health and Social Action (Senegal), National Agency of Statistics and Demography (Senegal). Senegal Continuous Demographic and Health Survey 2016. Fairfax, United States of America: ICF International, 2017.	286772
Senegal	2017	Senegal Continuous Demographic and Health Survey 2017	Survey microdata	Admin 1		X	X	X	X	X	X	X	ICF International, Ministry of Health and Social Action (Senegal), National Agency of Statistics and Demography (Senegal), Unit for the Fight Against Malnutrition (Senegal). Senegal Continuous Demographic and Health Survey 2017. Fairfax, United States of America: ICF International, 2018.	353526
Sierra Leone	2000	Sierra Leone Multiple Indicator Cluster	Survey microdata	Admin 1								X	Central Statistics Office (Sierra Leone), United Nations Children's Fund (UNICEF). Sierra Leone Multiple Indicator Cluster Survey 2000. New York, United States of America:	11639

Country	Years	Name	Type	Geographic Detail	Male Circumcision	STI Symptoms	Had Intercourse (Women)	Partner Away	Condom Last Time	Multiple Partners in Year (Men)	Multiple Partners in Year (Women)	In Union	Citation	NID*
		Survey 2000											United Nations Children's Fund (UNICEF).	
Sierra Leone	2005	Sierra Leone Multiple Indicator Cluster Survey 2005	Survey microdata	Admin 2			X				X		United Nations Children's Fund (UNICEF), Statistics Sierra Leone. Sierra Leone Multiple Indicator Cluster Survey 2005. New York, United States: United Nations Children's Fund (UNICEF).	11649
Sierra Leone	2008	Sierra Leone Demograp hic and Health Survey 2008	Survey microdata	GPS	X	X	X	X	X	X	X	X	Macro International, Inc, Statistics Sierra Leone. Sierra Leone Demographic and Health Survey 2008. Fairfax, United States of America: ICF International.	21258
Sierra Leone	2010	Sierra Leone Multiple Indicator Cluster Survey 2010	Survey microdata	Admin 2			X				X		Statistics Sierra Leone, United Nations Children's Fund (UNICEF). Sierra Leone Multiple Indicator Cluster Survey 2010. New York, United States of America: United Nations Children's Fund (UNICEF).	76700
Sierra Leone	2013	Sierra Leone	Survey microdata	GPS	X	X	X	X	X	X	X	X	ICF International, Ministry of Health and Sanitation (Sierra Leone),	131467

Country	Years	Name	Type	Geographic Detail	Male Circumcision	STI Symptoms	Had Intercourse (Women)	Partner Away	Condom Last Time	Multiple Partners in Year (Men)	Multiple Partners in Year (Women)	In Union	Citation	NID*
		Demographic and Health Survey 2013											Statistics Sierra Leone. Sierra Leone Demographic and Health Survey 2013. Fairfax, United States of America: ICF International, 2014.	
Sierra Leone	2014	Sierra Leone Labor Force Survey 2014	Survey microdata	Admin 2								X	German Society for International Cooperation (GIZ), International Labour Organization (ILO), Statistics Sierra Leone, World Bank. Sierra Leone Labor Force Survey 2014.	286094
South Africa	2002	South Africa - Agincourt Integrated Family Survey 2002	Survey microdata	Admin 4								X	Case, A. 2003. Agincourt Integrated Family Survey 2002. [dataset] Version 1. Cape Town: DataFirst [distributor].	135825
South Africa	2002	South Africa General Household Survey 2002	Survey microdata	Admin 1								X	Statistics South Africa. South Africa General Household Survey 2002. Pretoria, South Africa: Statistics South Africa.	115481

Country	Years	Name	Type	Geographic Detail	Male Circumcision	STI Symptoms	Had Intercourse (Women)	Partner Away	Condom Last Time	Multiple Partners in Year (Men)	Multiple Partners in Year (Women)	In Union	Citation	NID*
South Africa	2002	South Africa HIV/AIDS Behavioral Risks, Sero-Status, and Mass Media Impact Survey 2002	Survey microdata	Admin 3	X		X		X	X	X	X	Center for AIDS Development, Research and Evaluation (CADRE) (South Africa), Department of Social Development (South Africa), Family Health International, Geospace International (South Africa), Human Sciences Research Council, Joint United Nations Program on HIV/AIDS (UNAIDS), Medical University of Southern Africa (MEDUNSA), National Agency for AIDS Research (ANRS) (France), National Health Laboratory Service (NHLS) (South Africa), South African Medical Research Council, University of Natal, Wits Health Consortium. South Africa HIV/AIDS Behavioral Risks, Sero-Status, and Mass Media Impact Survey 2002. Pretoria, South Africa: Human Sciences Research Council, 2011.	12102
South Africa	2003	South Africa General Household	Survey microdata	Admin 1								X	Statistics South Africa. South Africa General Household Survey 2003. Pretoria, South Africa: Statistics South Africa.	11787

Country	Years	Name	Type	Geographic Detail	Male Circumcision	STI Symptoms	Had Intercourse (Women)	Partner Away	Condom Last Time	Multiple Partners in Year (Men)	Multiple Partners in Year (Women)	In Union	Citation	NID*
		Survey 2003												
South Africa	2004	South Africa General Household Survey 2004	Survey microdata	Admin 1								X	Statistics South Africa. South Africa General Household Survey 2004. Pretoria, South Africa: Statistics South Africa.	11788
South Africa	2005	South Africa General Household Survey 2005	Survey microdata	Admin 1								X	Statistics South Africa. South Africa General Household Survey 2005. Pretoria, South Africa: Statistics South Africa.	11789
South Africa	2005	South Africa National HIV Prevalence, Incidence, Behavior and Communic ation	Survey microdata	Admin 3			X		X	X	X	X	Center for AIDS Development, Research and Evaluation (CADRE) (South Africa), Centers for Disease Control and Prevention (CDC), Global Clinical and Viral Laboratory (South Africa), Human Sciences Research Council, Maphume Research Services, National Institute for Communicable Diseases (South Africa). South Africa National HIV Prevalence, Incidence, Behavior and	313074

Country	Years	Name	Type	Geographic Detail	Male Circumcision	STI Symptoms	Had Intercourse (Women)	Partner Away	Condom Last Time	Multiple Partners in Year (Men)	Multiple Partners in Year (Women)	In Union	Citation	NID*
		Survey 2004-2005											Communication Survey 2004-2005. Pretoria, South Africa: Human Sciences Research Council, 2011.	
South Africa	2006	South Africa General Household Survey 2006	Survey microdata	Admin 1								X	Statistics South Africa. South Africa General Household Survey 2006. Pretoria, South Africa: Statistics South Africa.	115486
South Africa	2007	South Africa General Household Survey 2007	Survey microdata	Admin 1								X	Statistics South Africa. South Africa General Household Survey 2007. Cape Town, South Africa: DataFirst.	11790
South Africa	2008	South Africa General Household Survey 2008	Survey microdata	Admin 1								X	Statistics South Africa. South Africa General Household Survey 2008. Pretoria, South Africa: Statistics South Africa.	115488
South Africa	2008-09	South Africa National HIV	Survey microdata	Admin 5, GPS	X	X	X		X	X	X	X	Center for AIDS Development, Research and Evaluation (CADRE) (South Africa), Geospace International (South Africa), Global	228102

Country	Years	Name	Type	Geographic Detail	Male Circumcision	STI Symptoms	Had Intercourse (Women)	Partner Away	Condom Last Time	Multiple Partners in Year (Men)	Multiple Partners in Year (Women)	In Union	Citation	NID*
		Prevalence, Incidence, Behavior and Communication Survey 2008-2009											Clinical and Viral Laboratory (South Africa), Human Sciences Research Council, Maphume Research Services, National Institute for Communicable Diseases (South Africa), South African Medical Research Council. South Africa National HIV Prevalence, Incidence, Behavior and Communication Survey 2008-2009. Pretoria, South Africa: Human Sciences Research Council, 2014.	
South Africa	2009	South Africa General Household Survey 2009	Survey microdata	Admin 1								X	Statistics South Africa. South Africa General Household Survey 2009. Pretoria, South Africa: Statistics South Africa.	115489
South Africa	2009	South Africa Living Conditions Survey 2008-2009	Survey microdata	Admin 1								X	Statistics South Africa. South Africa Living Conditions Survey 2008-2009. Pretoria, South Africa: Statistics South Africa.	152021

Country	Years	Name	Type	Geographic Detail	Male Circumcision	STI Symptoms	Had Intercourse (Women)	Partner Away	Condom Last Time	Multiple Partners in Year (Men)	Multiple Partners in Year (Women)	In Union	Citation	NID*
South Africa	2010-11	South Africa National Income Dynamics Study - Wave 2 2010-2011	Survey microdata	Admin 1, Admin 2, Admin 3								X	Southern Africa Labour and Development Research Unit. National Income Dynamics Study 2010-2011, Wave 2. Version 1.0. Cape Town: Southern Africa Labour and Development Research Unit [producer], 2012. Cape Town: DataFirst [distributor], 2013.	133731
South Africa	2011-12	South Africa National HIV Prevalence, Incidence, and Behavior Survey 2011-2012	Survey microdata	Admin 5, GPS	X		X		X	X	X	X	Centers for Disease Control and Prevention (CDC), Global Clinical and Viral Laboratory (South Africa), Human Sciences Research Council, National Institute for Communicable Diseases (South Africa), South African Medical Research Council, University of Cape Town. South Africa National HIV Prevalence, Incidence, and Behavior Survey 2011-2012. Pretoria, South Africa: Human Sciences Research Council, 2016.	313076
South Africa	2013	South Africa General Household	Survey microdata	Admin 1								X	Statistics South Africa. General Household Survey 2013 [dataset]. Version 1. Pretoria. Statistics South Africa [producer], 2014. Cape Town.	238483

Country	Years	Name	Type	Geographic Detail	Male Circumcision	STI Symptoms	Had Intercourse (Women)	Partner Away	Condom Last Time	Multiple Partners in Year (Men)	Multiple Partners in Year (Women)	In Union	Citation	NID*
		Survey 2013											DataFirst [distributor], 2014.10.25828/a704-wm31	
South Africa	2014	South Africa General Household Survey 2014	Survey microdata	Admin 1								X	Statistics South Africa. South Africa General Household Survey 2014. Cape Town, South Africa: DataFirst, 2015.	238485
South Africa	2015	South Africa Living Conditions Survey 2014-2015	Survey microdata	Admin 1								X	Statistics South Africa. South Africa Living Conditions Survey 2014-2015. Pretoria, South Africa: Statistics South Africa.	317239
South Africa	2016	South Africa Community Survey 2016	Survey microdata	Admin 3								X	Statistics South Africa. South Africa Community Survey 2016. Pretoria, South Africa: Statistics South Africa, 2016.	280803
South Africa	2016	South Africa Demograp hic and Health	Survey microdata	GPS	X	X	X	X	X	X	X	X	Department of Health (South Africa), ICF International, South African Medical Research Council, Statistics South Africa. South Africa Demographic and Health Survey	157064

Country	Years	Name	Type	Geographic Detail	Male Circumcision	STI Symptoms	Had Intercourse (Women)	Partner Away	Condom Last Time	Multiple Partners in Year (Men)	Multiple Partners in Year (Women)	In Union	Citation	NID*
		Survey 2016											2016. Fairfax, United States of America: ICF International, 2019.	
South Africa	2016	South Africa General Household Survey 2016	Survey microdata	Admin 1								X	Statistics South Africa. South Africa General Household Survey 2016. Cape Town, South Africa: DataFirst, 2015.	317089
South Sudan	2010	Sudan - South Multiple Indicator Cluster Survey 2010	Survey microdata	Admin 1			X				X	X	Central Bureau of Statistics (Sudan), Federal Ministry of Health (Sudan), Government of Sudan, Ministry of Health (South Sudan), Southern Sudan Centre for Census, Statistics and Evaluation. Sudan - South Multiple Indicator Cluster Survey 2010. New York, United States of America: United Nations Children's Fund (UNICEF), 2015.	32189
Sudan	2000	Sudan Multiple Indicator Cluster Survey 2000	Survey microdata	Admin 1								X	Central Bureau of Statistics (Sudan), Federal Ministry of Health (Sudan), United Nations Children's Fund (UNICEF). Sudan Multiple Indicator Cluster Survey 2000. New York, United States of America: United Nations Children's Fund (UNICEF).	12243

Country	Years	Name	Type	Geographic Detail	Male Circumcision	STI Symptoms	Had Intercourse (Women)	Partner Away	Condom Last Time	Multiple Partners in Year (Men)	Multiple Partners in Year (Women)	In Union	Citation	NID*
Sudan	2010	Sudan - North Multiple Indicator Cluster Survey 2010	Survey microdata	Admin 1								X	Central Bureau of Statistics (Sudan), Ministry of Health (South Sudan). Sudan - North Multiple Indicator Cluster Survey 2010. New York, United States of America: United Nations Children's Fund (UNICEF), 2015.	153643
Togo	2000	Togo Multiple Indicator Cluster Survey 2000	Survey microdata	Admin 1, Admin 2								X	United Nations Children's Fund (UNICEF). Togo Multiple Indicator Cluster Survey 2000. New York, United States of America: United Nations Children's Fund (UNICEF).	12886
Togo	2006	Togo Multiple Indicator Cluster Survey 2006	Survey microdata	Admin 1			X				X		Directorate General of Statistics and National Accounting (Togo), United Nations Children's Fund (UNICEF). Togo Multiple Indicator Cluster Survey 2006. New York, United States: United Nations Children's Fund (UNICEF).	12896
Togo	2010	Togo Multiple Indicator Cluster	Survey microdata	Admin 1			X		X	X	X	X	Directorate General of Statistics and National Accounting (Togo), United Nations Children's Fund (UNICEF). Togo Multiple Indicator Cluster Survey 2010. New York, United	40021

Country	Years	Name	Type	Geographic Detail	Male Circumcision	STI Symptoms	Had Intercourse (Women)	Partner Away	Condom Last Time	Multiple Partners in Year (Men)	Multiple Partners in Year (Women)	In Union	Citation	NID*
		Survey 2010											States: United Nations Children's Fund (UNICEF).	
Togo	2013-14	Togo Demographic and Health Survey 2013-2014	Survey microdata	GPS	X	X	X	X	X	X	X	X	Directorate General of Statistics and National Accounts (Togo), ICF International, Ministry of Health (Togo), Ministry of Planning, Development and Zoning (Togo). Togo Demographic and Health Survey 2013-2014. Fairfax, United States of America: ICF International, 2015.	77515
Togo	2017	Togo Malaria Indicator Survey 2017	Survey microdata	GPS								X	ICF International, National Institute of Hygiene, Ministry of Health (Togo), National Institute of Statistics and Economic and Demographic Studies (INSEED) (Togo), National Program to Fight Malaria, Ministry of Health (Togo). Togo Malaria Indicator Survey 2017. Fairfax, United States of America: ICF International, 2018.	359318
Uganda	2000-01	Uganda Demographic and Health	Survey microdata	GPS		X	X	X	X	X	X	X	Macro International, Inc, Uganda Bureau of Statistics. Uganda Demographic and Health Survey 2000-2001. Fairfax, United States of America: ICF International.	20993

Country	Years	Name	Type	Geographic Detail	Male Circumcision	STI Symptoms	Had Intercourse (Women)	Partner Away	Condom Last Time	Multiple Partners in Year (Men)	Multiple Partners in Year (Women)	In Union	Citation	NID*
		Survey 2000-2001												
Uganda	2004	Uganda National Service Delivery Survey 2004	Survey microdata	Admin 1								X	Ministry of Public Service (Uganda), Uganda Bureau of Statistics. Uganda National Service Delivery Survey 2004. OpenMicroData.	13114
Uganda	2004-05	Uganda AIDS Indicator Survey 2004-2005	Survey microdata	Admin 1	X	X	X			X	X	X	Division of Reproductive Health, Centers for Disease Control and Prevention (CDC), Ministry of Health (Uganda). Uganda AIDS Indicator Survey 2004-2005.	13084
Uganda	2006	Uganda Demographic and Health Survey 2006	Survey microdata	GPS	X	X	X	X	X	X	X	X	Macro International, Inc, Uganda Bureau of Statistics. Uganda Demographic and Health Survey 2006. Fairfax, United States of America: ICF International.	21014
Uganda	2008	Uganda National Service Delivery Survey 2008	Survey microdata	Admin 1								X	Ministry of Public Service (Uganda), Uganda Bureau of Statistics. Uganda National Service Delivery Survey 2008.	93320

Country	Years	Name	Type	Geographic Detail	Male Circumcision	STI Symptoms	Had Intercourse (Women)	Partner Away	Condom Last Time	Multiple Partners in Year (Men)	Multiple Partners in Year (Women)	In Union	Citation	NID*
Uganda	2011	Uganda AIDS Indicator Survey 2011	Survey microdata	GPS	X	X	X	X	X	X	X	X	Centers for Disease Control and Prevention (CDC), ICF Macro, Ministry of Health (Uganda), Uganda Bureau of Statistics, Uganda Virus Research Institute. Uganda AIDS Indicator Survey 2011. Fairfax, United States of America: ICF International.	55973
Uganda	2011	Uganda Demographic and Health Survey 2011	Survey microdata	GPS	X	X	X	X	X	X	X	X	ICF Macro, Uganda Bureau of Statistics. Uganda Demographic and Health Survey 2011. Fairfax, United States of America: ICF International.	56021
Uganda	2013	Uganda National Household Survey 2012-2013	Survey microdata	Admin 1								X	Uganda Bureau of Statistics. Uganda National Household Survey 2012-2013. Kampala, Uganda: Uganda Bureau of Statistics.	165406
Uganda	2014	Uganda Performance Monitoring and Accountability 2020	Survey microdata	Admin 1			X	X				X	Makerere University, School of Public Health at the College of Health Sciences and The Bill & Melinda Gates Institute for Population and Reproductive Health at The Johns Hopkins Bloomberg School of Public Health. Performance Monitoring and	256199

Country	Years	Name	Type	Geographic Detail	Male Circumcision	STI Symptoms	Had Intercourse (Women)	Partner Away	Condom Last Time	Multiple Partners in Year (Men)	Multiple Partners in Year (Women)	In Union	Citation	NID*
		Survey, Round 1 2014											Accountability 2020 (PMA2020) Survey round 1, PMA2014/Uganda-R1. 2014. Uganda and Baltimore, Maryland, USA.	
Uganda	2015	Uganda Performance Monitoring and Accountability 2020 Survey, Round 2 2015	Survey microdata	Admin 1			X	X				X	Makerere University, School of Public Health at the College of Health Sciences and The Bill & Melinda Gates Institute for Population and Reproductive Health at The Johns Hopkins Bloomberg School of Public Health. Performance Monitoring and Accountability 2020 (PMA2020) Survey round 2, PMA2015/Uganda-R2. 2015. Uganda and Baltimore, Maryland, USA.	256200
Uganda	2015	Uganda Performance Monitoring and Accountability 2020 Survey, Round 3 2015	Survey microdata	Admin 1			X	X				X	Makerere University, School of Public Health at the College of Health Sciences and The Bill & Melinda Gates Institute for Population and Reproductive Health at The Johns Hopkins Bloomberg School of Public Health. Performance Monitoring and Accountability 2020 (PMA2020) Survey round 3, PMA2015/Uganda-	256201

Country	Years	Name	Type	Geographic Detail	Male Circumcision	STI Symptoms	Had Intercourse (Women)	Partner Away	Condom Last Time	Multiple Partners in Year (Men)	Multiple Partners in Year (Women)	In Union	Citation	NID*
													R3. 2015. Uganda and Baltimore, Maryland, USA.	
Uganda	2016	Uganda Demographic and Health Survey 2016	Survey microdata	GPS	X	X	X	X	X	X	X	X	ICF International, Uganda Bureau of Statistics. Uganda Demographic and Health Survey 2016. Fairfax, United States of America: ICF International, 2018.	286780
Uganda	2016	Uganda Performance Monitoring and Accountability 2020 Survey, Round 4 2016	Survey microdata	Admin 1			X	X				X	Makerere University, School of Public Health at the College of Health Sciences and The Bill & Melinda Gates Institute for Population and Reproductive Health at The Johns Hopkins Bloomberg School of Public Health. Performance Monitoring and Accountability 2020 (PMA2020) Survey round 4, PMA2016/Uganda-R4. 2016. Uganda and Baltimore, Maryland, USA.	285893
Uganda	2016	Uganda Population-based HIV Impact Assessmen	Survey report	Admin 1	X								Centers for Disease Control and Prevention (CDC), ICAP, Columbia University Mailman School of Public Health, Joint United Nations Program on HIV/AIDS (UNAIDS), Ministry of Health (Uganda), Uganda Bureau of	327593

Country	Years	Name	Type	Geographic Detail	Male Circumcision	STI Symptoms	Had Intercourse (Women)	Partner Away	Condom Last Time	Multiple Partners in Year (Men)	Multiple Partners in Year (Women)	In Union	Citation	NID*
		t 2016-2017											Statistics, Uganda Virus Research Institute, World Health Organization (WHO). Uganda Population-Based HIV Impact Assessment 2016-2017.	
Uganda	2017	Uganda Performance Monitoring and Accountability 2020 Survey, Round 5 2017	Survey microdata	Admin >1			X	X				X	Makerere University, School of Public Health at the College of Health Sciences and The Bill & Melinda Gates Institute for Population and Reproductive Health at The Johns Hopkins Bloomberg School of Public Health. Performance Monitoring and Accountability 2020 (PMA2020) Survey round 5, PMA2017/Uganda-R5. 2017. Uganda and Baltimore, Maryland, USA.	347043
Uganda	2018	Uganda Performance Monitoring and Accountability 2020 Survey, Round 6 2018	Survey microdata	Admin 1			X					X	Makerere University, School of Public Health at the College of Health Sciences and The Bill & Melinda Gates Institute for Population and Reproductive Health at The Johns Hopkins Bloomberg School of Public Health. Performance Monitoring and Accountability 2020 (PMA2020) Survey round 6, PMA2018/Uganda-	398722

Country	Years	Name	Type	Geographic Detail	Male Circumcision	STI Symptoms	Had Intercourse (Women)	Partner Away	Condom Last Time	Multiple Partners in Year (Men)	Multiple Partners in Year (Women)	In Union	Citation	NID*
													R6. 2018. Uganda and Baltimore, Maryland, USA.	
United Republic of Tanzania	2001	Tanzania Household Budget Survey 2000-2001	Survey microdata	Admin 1								X	National Bureau of Statistics (Tanzania), Oxford Policy Management. Tanzania Household Budget Survey 2000-2001. Dar es Salaam, United Republic of Tanzania: National Bureau of Statistics (Tanzania).	31740
United Republic of Tanzania	2003-04	Tanzania AIDS Indicator Survey 2003-2004	Survey microdata	GPS	X	X	X		X	X	X	X	National Bureau of Statistics (Tanzania), ORC Macro, Tanzania Commission for AIDS (TACAIDS). Tanzania AIDS Indicator Survey 2003-2004. Fairfax, United States of America: ICF International.	12630
United Republic of Tanzania	2004	Tanzania - Shinyanga Core Welfare Indicators Questionnaire Survey 2004	Survey microdata	Admin 2								X	Economic Development Initiatives (EDI), National Bureau of Statistics (Tanzania), Institute of Finance Management (Tanzania), World Bank (WB). Tanzania - Shinyanga Core Welfare Indicators Questionnaire Survey 2004. Bukoba, Tanzania: Economic Development Initiatives (EDI).	31786

Country	Years	Name	Type	Geographic Detail	Male Circumcision	STI Symptoms	Had Intercourse (Women)	Partner Away	Condom Last Time	Multiple Partners in Year (Men)	Multiple Partners in Year (Women)	In Union	Citation	NID*
United Republic of Tanzania	2004-05	Tanzania Demographic and Health Survey 2004-2005	Survey microdata	Admin 1	X	X	X	X	X	X	X	X	Macro International, Inc, National Bureau of Statistics (Tanzania). Tanzania Demographic and Health Survey 2004-2005. Fairfax, United States of America: ICF International.	20875
United Republic of Tanzania	2005	Tanzania Core Welfare Indicators Questionnaire Survey 2005	Survey microdata	Admin 2								X	Economic Development Initiatives (EDI), World Bank (WB). Tanzania Core Welfare Indicators Questionnaire Survey 2005. Bukoba, Tanzania: Economic Development Initiatives (EDI).	31797
United Republic of Tanzania	2006-07	Tanzania Core Welfare Indicators Questionnaire Survey 2006-2007	Survey microdata	Admin 2								X	Economic Development Initiatives (EDI), World Bank (WB). Tanzania Core Welfare Indicators Questionnaire Survey 2006-2007. Bukoba, Tanzania: Economic Development Initiatives (EDI).	31831
United Republic of Tanzania	2007	Tanzania Household Budget Survey 2007	Survey microdata	Admin 1								X	National Bureau of Statistics (Tanzania). Tanzania Household Budget Survey 2007. Dar es Salaam, Tanzania: National Bureau of Statistics (Tanzania).	31887

Country	Years	Name	Type	Geographic Detail	Male Circumcision	STI Symptoms	Had Intercourse (Women)	Partner Away	Condom Last Time	Multiple Partners in Year (Men)	Multiple Partners in Year (Women)	In Union	Citation	NID*
United Republic of Tanzania	2007-08	Tanzania HIV/AIDS and Malaria Indicator Survey 2007-2008	Survey microdata	GPS	X	X	X	X	X	X	X	X	Macro International, Inc, National Bureau of Statistics (Tanzania), Office of the Chief Government Statistician (OCGS) (Zanzibar), Tanzania Commission for AIDS (TACAIDS), Zanzibar AIDS Commission (ZAC). Tanzania HIV/AIDS and Malaria Indicator Survey 2007-2008. Fairfax, United States of America: ICF International.	12644
United Republic of Tanzania	2009-10	Tanzania Demographic and Health Survey 2009-2010	Survey microdata	GPS	X	X	X	X	X	X	X	X	ICF Macro, National Bureau of Statistics (Tanzania). Tanzania Demographic and Health Survey 2009-2010. Fairfax, United States of America: ICF International.	21331
United Republic of Tanzania	2011-12	Tanzania AIDS Indicator Survey 2011-2012	Survey microdata	GPS	X	X	X	X	X	X	X	X	ICF International, National Bureau of Statistics (Tanzania), Office of the Chief Government Statistician (OCGS) (Zanzibar), Tanzania Commission for AIDS (TACAIDS), Zanzibar AIDS Commission (ZAC). Tanzania AIDS Indicator Survey 2011-2012. Fairfax, United States of America: ICF International, 2013.	77395

Country	Years	Name	Type	Geographic Detail	Male Circumcision	STI Symptoms	Had Intercourse (Women)	Partner Away	Condom Last Time	Multiple Partners in Year (Men)	Multiple Partners in Year (Women)	In Union	Citation	NID*
United Republic of Tanzania	2012	Tanzania Household Budget Survey 2011-2012	Survey microdata	Admin 1								X	National Bureau of Statistics (Tanzania). Tanzania Household Budget Survey 2011-2012. Dar es Salaam, Tanzania: National Bureau of Statistics (Tanzania), 2014.	243012
United Republic of Tanzania	2015	Tanzania National Panel Survey 2014-2016	Survey microdata	GPS								X	National Bureau of Statistics (Tanzania), World Bank. Tanzania National Panel Survey 2014-2016. Washington DC, United States of America: World Bank.	311265
United Republic of Tanzania	2015-16	Tanzania Demographic and Health Survey 2015-2016	Survey microdata	GPS	X		X	X	X			X	ICF International, Ministry of Health (Zanzibar), Ministry of Health, Community Development, Gender, Elderly and Children (MoHCDEC) (Tanzania), National Bureau of Statistics (Tanzania), Office of the Chief Government Statistician (OCGS) (Zanzibar). Tanzania Demographic and Health Survey 2015-2016. Fairfax, United States of America: ICF International, 2016.	218593
United Republic of Tanzania	2017	Tanzania HIV Impact Survey 2016-2017	Survey microdata	Admin 1	X								Centers for Disease Control and Prevention (CDC), ICAP, Columbia University Mailman School of Public Health, Ministry of Health (Zanzibar),	327591

Country	Years	Name	Type	Geographic Detail	Male Circumcision	STI Symptoms	Had Intercourse (Women)	Partner Away	Condom Last Time	Multiple Partners in Year (Men)	Multiple Partners in Year (Women)	In Union	Citation	NID*
													Ministry of Health, Community Development, Gender, Elderly and Children (MoHCDEC) (Tanzania), National AIDS Control Program (NACP) (Tanzania), National Bureau of Statistics (Tanzania), National Health Laboratory Quality Assurance and Training Centre (NHLQATC) (Tanzania), Office of the Chief Government Statistician (OCGS) (Zanzibar), Tanzania Commission for AIDS (TACAIDS), Zanzibar AIDS Commission (ZAC), Zanzibar Integrated HIV, Tuberculosis and Leprosy Program (ZIHTLP). Tanzania HIV Impact Survey 2016-2017.	
Zambia	2000	Zambia Sexual Behavior Survey 2000	Survey microdata	Admin 2	X	X	X	X		X	X	X	Central Board of Health (Zambia), Central Statistical Office (Zambia), MEASURE Evaluation Project, Carolina Population Center, University of North Carolina, University of Zambia, Zambian National AIDS Council. Zambia Sexual Behavior Survey 2000. Lusaka,	27924

Country	Years	Name	Type	Geographic Detail	Male Circumcision	STI Symptoms	Had Intercourse (Women)	Partner Away	Condom Last Time	Multiple Partners in Year (Men)	Multiple Partners in Year (Women)	In Union	Citation	NID*
													Zambia: Central Statistical Office (Zambia).	
Zambia	2001-02	Zambia Demographic and Health Survey 2001-2002	Survey microdata	Admin 2		X	X	X	X	X	X	X	Central Board of Health (Zambia), Central Statistical Office (Zambia), Macro International, Inc. Zambia Demographic and Health Survey 2001-2002. Fairfax, United States of America: ICF International.	21102
Zambia	2003	Zambia Living Conditions Monitoring Survey 2002-2003	Survey microdata	Admin 2								X	Central Statistical Office (Zambia). Zambia Living Conditions Monitoring Survey 2002-2003. Lusaka, Zambia: Central Statistical Office (Zambia).	14027
Zambia	2003	Zambia Sexual Behavior Survey 2003	Survey microdata	Admin 2	X	X	X	X				X	Central Board of Health (Zambia), Central Statistical Office (Zambia), MEASURE Evaluation Project, Carolina Population Center, University of North Carolina, Zambian National AIDS Council. Zambia Sexual Behavior Survey 2003. Lusaka, Zambia: Central Statistical Office (Zambia).	27952
Zambia	2005	Zambia Sexual	Survey microdata	Admin 2	X	X	X	X		X	X	X	Central Statistical Office (Zambia), Department for International	27987

Country	Years	Name	Type	Geographic Detail	Male Circumcision	STI Symptoms	Had Intercourse (Women)	Partner Away	Condom Last Time	Multiple Partners in Year (Men)	Multiple Partners in Year (Women)	In Union	Citation	NID*
		Behavior Survey 2005											Development (DFiD) (United Kingdom), Family Health International, MEASURE Evaluation Project, Carolina Population Center, University of North Carolina, Ministry of Health (Zambia), University of Zambia, Zambian National AIDS Council. Zambia Sexual Behavior Survey 2005. Lusaka, Zambia: Central Statistical Office (Zambia).	
Zambia	2007	Zambia Demographic and Health Survey 2007	Survey microdata	GPS	X	X	X	X	X	X	X	X	Central Statistical Office (Zambia), Macro International, Inc. Zambia Demographic and Health Survey 2007. Fairfax, United States of America: ICF International.	21117
Zambia	2008	Zambia Global Fund Household Health Coverage Survey 2008	Survey microdata	Admin 3			X	X					Central Statistical Office (Zambia). Zambia Global Fund Household Health Coverage Survey 2008. Lusaka, Zambia: Central Statistical Office (Zambia).	26702

Country	Years	Name	Type	Geographic Detail	Male Circumcision	STI Symptoms	Had Intercourse (Women)	Partner Away	Condom Last Time	Multiple Partners in Year (Men)	Multiple Partners in Year (Women)	In Union	Citation	NID*
Zambia	2009	Zambia Sexual Behavior Survey 2009	Survey microdata	Admin 2	X	X	X	X		X	X	X	Central Statistical Office (Zambia), MEASURE Evaluation Project, Carolina Population Center, University of North Carolina, Ministry of Health (Zambia), University of Zambia, Zambian National AIDS Council. Zambia Sexual Behavior Survey 2009.	59339
Zambia	2010	Zambia Living Conditions Monitoring Survey 2010	Survey microdata	Admin 3								X	Central Statistical Office (Zambia). Zambia Living Conditions Monitoring Survey 2010.	58660
Zambia	2013-14	Zambia Demographic and Health Survey 2013-2014	Survey microdata	GPS	X	X	X	X	X	X	X	X	Central Statistical Office (Zambia), ICF International, Ministry of Health (Zambia), Tropical Diseases Research Centre, University Teaching Hospital (Zambia), University of Zambia. Zambia Demographic and Health Survey 2013-2014. Fairfax, United States of America: ICF International.	77516
Zambia	2016	Zambia Population-based HIV	Survey microdata	Admin 2	X	X	X	X	X	X	X	X	Ministry of Health, Zambia, Centers for Disease Control and Prevention (CDC), and ICAP at Columbia	287630

Country	Years	Name	Type	Geographic Detail	Male Circumcision	STI Symptoms	Had Intercourse (Women)	Partner Away	Condom Last Time	Multiple Partners in Year (Men)	Multiple Partners in Year (Women)	In Union	Citation	NID*
		Impact Assessment 2016											University. Zambia Population-based HIV Impact Assessment (ZAMPHIA) 2015-16: First Report. Lusaka, Zambia, Atlanta, Georgia and New York, New York, USA: Ministry of Health, CDC and ICAP. December 2016.	
Zimbabwe	2005	Zimbabwe - Chimanimani Behavioral Risks and HIV Serostatus Survey 2005	Survey report	Admin 2	X								Biomedical Research and Training Institute (Zimbabwe), Human Sciences Research Council, National Institute of Health Research, Ministry of Health and Child Welfare (NIHR) (Zimbabwe), Zimbabwe Central Statistics Office. Zimbabwe - Chimanimani Behavioral Risks and HIV Serostatus Survey 2005.	333662
Zimbabwe	2005-06	Zimbabwe Demographic and Health Survey 2005-2006	Survey microdata	GPS	X	X	X	X	X	X	X	X	Central Statistical Office (Zimbabwe), Macro International, Inc. Zimbabwe Demographic and Health Survey 2005-2006. Fairfax, United States of America: ICF International.	21163
Zimbabwe	2009	Zimbabwe Multiple	Survey microdata	Admin 1								X	Central Statistical Office (Zimbabwe). Zimbabwe Multiple Indicator	35493

Country	Years	Name	Type	Geographic Detail	Male Circumcision	STI Symptoms	Had Intercourse (Women)	Partner Away	Condom Last Time	Multiple Partners in Year (Men)	Multiple Partners in Year (Women)	In Union	Citation	NID*
		Indicator Monitoring Survey 2009											Monitoring Survey 2009. New York, United States of America: United Nations Children's Fund (UNICEF).	
Zimbabwe	2010-11	Zimbabwe Demographic and Health Survey 2010-2011	Survey microdata	GPS	X	X	X	X	X	X	X	X	ICF Macro, Zimbabwe National Statistics Agency. Zimbabwe Demographic and Health Survey 2010-2011. Calverton, United States of America: ICF Macro, 2012.	55992
Zimbabwe	2014	Zimbabwe Multiple Indicator Cluster Survey 2014	Survey microdata	Admin 1	X		X		X	X	X	X	United Nations Children's Fund (UNICEF), Zimbabwe National Statistics Agency. Zimbabwe Multiple Indicator Cluster Survey 2014. New York, United States of America: United Nations Children's Fund (UNICEF), 2015.	152720
Zimbabwe	2015	Zimbabwe Demographic and Health Survey 2015	Survey microdata	GPS	X	X	X	X	X	X	X	X	ICF International, National Microbiology Reference Laboratory, Harare Central Hospital (NMRL) (Zimbabwe), Zimbabwe National Statistics Agency. Zimbabwe Demographic and Health Survey 2015. Fairfax, United States of America: ICF International, 2016.	157066

Country	Years	Name	Type	Geographic Detail	Male Circumcision	STI Symptoms	Had Intercourse (Women)	Partner Away	Condom Last Time	Multiple Partners in Year (Men)	Multiple Partners in Year (Women)	In Union	Citation	NID*
Zimbabwe	2016	Zimbabwe Population-based HIV Impact Assessment 2015-2016	Survey report	Admin 1	X								Ministry of Health and Child Care (MOHCC), Zimbabwe, Centers for Disease Control and Prevention (CDC), and ICAP at Columbia University. Zimbabwe Population-based HIV Impact Assessment (ZIMPHIA) 2015-16: First Report. Harare, Zimbabwe, Atlanta, Georgia and New York, New York, USA: Ministry of Health, CDC and ICAP. December 2016.	287631

*NID = Data source unique identifier in the Global Health Data Exchange (http://ghdx.healthdata.org/). Additional information about each data sources is available via the GHDx. NIDs can be entered in the search bar to retrieve the record for a particular source.

Appendix Table 7: HIV treatment administrative data

Country	UNAIDS Year	UNAIDS Geography Level	Year(s) of data	Source	NID*	Admin. level(s)
Benin	2019	1	2013	National AIDS Control Program (Benin)	264954	0.5
Benin	2019	1	2014, 2015, 2016	National AIDS Control Program (Benin)	336678, 336679, 336680	1
Benin	2019	1	2017, 2018	Ministry of Health (Benin)	436859, 436860	2
Burkina Faso	2019	0	2011, 2012, 2013, 2015, 2016	Burkina Faso Health Statistical Yearbook 2011-2013, 2015, 2016	261322, 261326, 261327, 263729, 336692	2
Burundi	2019	0	2012, 2013, 2014, 2015, 2016	Burundi National Council for the Fight Against AIDS	407806, 407806	Health District
Cameroon	2019	0	2009, 2010, 2013, 2014, 2015, 2016	National AIDS control program	436374, 436375, 436386, 436388, 437654, 436389	1
Cameroon	2019	0	2006, 2007, 2008, 2012	National AIDS control program	436325, 436374	2*
Côte d'Ivoire	2019	1	2016, 2017, 2018	PEPFAR	413822	2
Côte d'Ivoire	2019	1	2015, 2016	Annual Report on the Health Situation (RASS) 2015 -2016	423019	2*
Democratic Republic of the Congo	2019	0	2015, 2017	National AIDS and STI Control Program	436391, 436392	1
Eswatini	2019	0	2010	HIV Program, Ministry of Health (Eswatini)	155083	1
Eswatini	2019	0	2011, 2012, 2013, 2014, 2015, 2016	HIV Program, Ministry of Health (Eswatini)	436478, 436479	1
Ethiopia	2019	1	2006, 2007, 2008, 2009, 2010, 2011	Ethiopia Health and Health Related Indicators 2006-2011	22261, 152618, 258115, 258117, 258127, 356526, 356529	1
Gambia	2019	0	2012, 2013, 2014, 2015, 2016, 2017, 2018	National HIV and AIDS Secretariat (NAS)	436862, 436863, 436864, 436865, 436866, 436867, 436868	1.5

Country	UNAIDS Year	UNAIDS Geography Level	Year(s) of data	Source	NID*	Admin. level(s)
Guinea Bissau	2019	0	2014, 2015	National AIDS Control Program (SNLS)	436870, 436872	1†
Guinea Bissau	2019	0	2016, 2017, 2018	National AIDS Control Program (SNLS)	436873, 436874, 436875	1
Kenya	2019	1	2016, 2017, 2018	PEPFAR	415118	2
Madagascar	2019	0	2014	National AIDS Control Committee (Madagascar)	233281	2
Malawi	2019	0	2004, 2005, 2013, 2014, 2015, 2016, 2017	Report of a Country Wide Survey of HIV/AIDS Services in Malawi	397876, 397875, 397872, 397871, 397767, 398090, 398085	2†
Mali	2019	0	2008	Mali MoH, National HIV/AIDS Control Program	74361	1
Mali	2019	0	2009	Mali MoH, Planning and Statistics Unit	24136	1
Mozambique	2019	1	2013, 2014, 2015, 2016, 2017	Relatorio Anual HIV	399432, 399434, 399436, 399437, 399516	2
Namibia	2019	0	2016, 2017, 2018	PEPFAR	415125	1
Niger	2019	0	2010, 2011, 2012, 2013, 2014, 2015, 2016	Annual Health Statistics of Niger 2010-2016	418131, 418130, 418128, 418127, 418124, 418116	1†
Nigeria	2019	0	2016, 2017, 2018	PEPFAR	436511	2
Republic of the Congo	2019	0	2013	National AIDS Control Council (Congo)	232196	1
Republic of the Congo	2019	0	2009, 2010, 2011, 2012	Ministry of Health and Population (Congo)	422553	1
Rwanda	2019	0	2004 - 2017	Rwanda Antiretroviral Therapy Data 2004-2018	429891, 429892, 429893, 429894, 429895, 429896, 429897, 429898, 429899, 429900,	2†

Country	UNAIDS Year	UNAIDS Geography Level	Year(s) of data	Source	NID*	Admin. level(s)
					429901, 429902, 429903, 429904, 429905	
Senegal	2019	0	2015, 2016, 2017	National AIDS Council	436400, 436411, 436406	1
Sierra Leone	2019	0	2013	National Strategic Plan on HIV/AIDS 2016-2020	436422	2
Somalia	2019	0	2018	Somalia Patients Alive on ART 2018	436926	1†
Somalia	2019	0	2013, 2014, 2015, 2016, 2017	Annual Report on HIV/AIDS in Puntland 2017	436463	1-
South Africa	Thembisa 4.2	1	2013, 2014, 2015	Provincial Annual AIDS Progress Reports	406867, 406898, 406904, 406905	2
Togo	2019	0	2008, 2010, 2011, 2012, 2015, 2016, 2017	Ministry of Health, National Program for the Fight against AIDS and STIs	436490, 436484, 436487, 436495, 436504, 436505, 436508	1
Uganda	2019	0	2016, 2017, 2018	PEPFAR	415138	1
Uganda	2019	0	2008, 2010	STD/AIDS Control Program, MoH (Uganda)	356783, 321955	1†
United Republic of Tanzania	2019	0	2016, 2017, 2018	PEPFAR	415130	2
Zambia	2019	1	2017, 2018	National File Currently on ART	425195	2†
Zimbabwe	2019	1	2016, 2017, 2018	PEPFAR	436514	2

*NID = Data source unique identifier in the Global Health Data Exchange (http://ghdx.healthdata.org/). Additional information about each data sources is available via the GHDx. NIDs can be entered in the search bar to retrieve the record for a particular source.

†Indicates that facility-level data were matched to an administrative unit.

Appendix Table 8: ART model parameters

Country	φ*	τ*	Precision of Gaussian Observations*	β*
Benin	0.57 (0.21)	0.02 (0)	590.02 (50.47)	2.91 (0.6)
Burkina Faso	0.1 (0.1)	6.91 (1.46)	3642.68 (349.88)	0.54 (0.05)
Burundi	0.88 (0.07)	1.02 (0.14)	48.52 (2.35)	0.95 (0.03)
Cameroon	0.06 (0.07)	2.99 (0.58)	2979.68 (240.37)	0.79 (0.08)
Côte d'Ivoire	0.17 (0.15)	1.51 (0.49)	107.12 (11.47)	1.25 (0.14)
Democratic Republic of the Congo	0.84 (0.07)	3.49 (0.4)	290.5 (13.3)	0.88 (0.02)
Eswatini	0.94 (0.07)	208.63 (50.79)	365.18 (22.97)	0.99 (0.01)
Ethiopia	0.87 (0.12)	25.12 (5.33)	2252.13 (122.27)	1.13 (0.01)
Gambia	0.76 (0.13)	1.77 (0.53)	564.42 (44.8)	1.12 (0.06)
Guinea-Bissau	0.87 (0.12)	6.65 (1.61)	346.12 (30.34)	0.66 (0.03)
Kenya	0.08 (0.07)	0.54 (0.05)	144.39 (5.49)	1.2 (0.08)
Madagascar	0.26 (0.19)	1.25 (0.38)	248944.15 (57136.61)	0.9 (0.19)
Malawi	0.96 (0.06)	0.04 (0.01)	38.28 (3.79)	2.51 (0.26)
Mali	0.92 (0.08)	6.32 (1.36)	27406.67 (3317.95)	0.52 (0.03)
Mozambique	0.05 (0.06)	3.5 (0.46)	114.32 (5.37)	0.99 (0.05)
Namibia	0.81 (0.13)	7.32 (1.44)	49.88 (3.4)	1.1 (0.02)
Niger	0.96 (0.04)	3.54 (0.64)	454.97 (27.45)	0.76 (0.02)
Nigeria	0.59 (0.21)	0.71 (0.17)	226.18 (16.6)	0.85 (0.25)
Republic of Congo	0.95 (0.05)	5.46 (0.93)	764.87 (44.71)	0.57 (0.01)
Rwanda	0.29 (0.22)	16.18 (4.34)	179.24 (11.76)	1.06 (0.04)
Senegal	0.9 (0.1)	14.76 (3.66)	1093.32 (103.23)	0.89 (0.01)
Sierra Leone	0.53 (0.28)	5.65 (2.08)	207695.48 (53091.13)	0.81 (0.08)
Somalia	0.66 (0.15)	2.06 (0.5)	196505.2 (34516.83)	0.86 (0.08)
South Africa	0.6 (0.23)	63.97 (16.53)	499.62 (39.99)	0.87 (0.01)
Togo	0.95 (0.07)	41.38 (11.98)	645.64 (55.33)	0.8 (0.01)
Uganda	0.38 (0.12)	2.96 (0.38)	91.23 (3.53)	1.07 (0.03)
United Republic of Tanzania	0.78 (0.07)	1.5 (0.18)	233.29 (11.45)	0.94 (0.03)
Zambia	0.06 (0.06)	0.11 (0.02)	46.77 (3.88)	2.79 (0.35)
Zimbabwe	0.33 (0.26)	203.13 (452.19)	3599.32 (1508.84)	1.03 (0.14)

*All values are: mean (standard error)

References

1 Ghys PD, Brown T, Grassly NC, *et al.* The UNAIDS Estimation and Projection Package: a software package to estimate and project national HIV epidemics. *Sexually Transmitted Infections* 2004; **80**: i5–9.

2 Eaton JW, Brown T, Puckett R, *et al.* The estimation and projection package age-sex model and the r-hybrid model: new tools for estimating HIV incidence trends in sub-Saharan Africa. *AIDS* 2019; **33**: S235.

3 GBD 2019 Diseases and Injuries Collaborators. Global burden of 369 diseases and injuries in 204 countries and territories, 1990–2019: a systematic analysis for the global burden of disease study 2019. *The Lancet* 2020; **396**: 1204–22.

4 Raftery AE, Bao L. Estimating and Projecting Trends in HIV/AIDS Generalized Epidemics Using Incremental Mixture Importance Sampling. *Biometrics* 2010; **66**: 1162–73.

5 Frank TD, Carter A, Jahagirdar D, *et al.* Global, regional, and national incidence, prevalence, and mortality of HIV, 1980–2017, and forecasts to 2030, for 195 countries and territories: a systematic analysis for the Global Burden of Diseases, Injuries, and Risk Factors Study 2017. *The Lancet HIV* 2019; **6**: e831–59.

6 Case KK, Johnson LF, Mahy M, Marsh K, Supervie V, Eaton JW. Summarizing the results and methods of the 2019 Joint United Nations Programme on HIV/AIDS HIV estimates. *AIDS* 2019; **33**: S197.

7 Stover J, Brown T, Puckett R, Peerapatanapokin W. Updates to the Spectrum/Estimations and Projections Package model for estimating trends and current values for key HIV indicators. *AIDS* 2017; **31**: S5.

8 Brown T, Bao L, Eaton JW, *et al.* Improvements in prevalence trend fitting and incidence estimation in EPP 2013. *AIDS* 2014; **28**: S415.

9 Wang H, Wolock TM, Carter A, *et al.* Estimates of global, regional, and national incidence, prevalence, and mortality of HIV, 1980–2015: the Global Burden of Disease Study 2015. *The Lancet HIV* 2016; **3**: e361–87.

10 Vos T, Abajobir AA, Abate KH, *et al.* Global, regional, and national incidence, prevalence, and years lived with disability for 328 diseases and injuries for 195 countries, 1990–2016: a systematic analysis for the Global Burden of Disease Study 2016. *The Lancet* 2017; **390**: 1211–59.

11 Dwyer-Lindgren L, Cork MA, Sligar A, *et al.* Mapping HIV prevalence in sub-Saharan Africa between 2000 and 2017. *Nature* 2019; **570**: 189–93.

12 Global Administrative Areas (GADM). GADM database of global administrative areas. 2018. http://www.gadm.org (accessed May 6, 2018).

13 WorldPop. WorldPop dataset. http://www.worldpop.org.uk/data/get_data/ (accessed July 24, 2017).

14 GBD 2019 Demographics Collaborators. Global age-sex-specific fertility, mortality, healthy life expectancy (HALE), and population estimates in 204 countries and territories, 1950–2019: a comprehensive demographic analysis for the Global Burden of Disease Study 2019. *The Lancet* 2020; **396**: 1160–1203.

15 Graetz N, Friedman J, Osgood-Zimmerman A, *et al.* Mapping local variation in educational attainment across Africa. *Nature* 2018; **555**: 48–53.

16 Golding N, Burstein R, Longbottom J, *et al.* Mapping under-5 and neonatal mortality in Africa, 2000-15: a baseline analysis for the Sustainable Development Goals. *Lancet* 2017; **390**: 2171–82.

17 Osgood-Zimmerman A, Millear AI, Stubbs RW, *et al.* Mapping child growth failure in Africa between 2000 and 2015. *Nature* 2018; **555**: 41–7.

18 Institute for Health Metrics and Evaluation (IHME). Global Health Data Exchange. GHDx. http://ghdx.healthdata.org/ (accessed May 12, 2020).

19 Institute for Health Metrics and Evaluation (IHME). Call for Collaborators. IHME. 2019. http://www.healthdata.org/gbd/call-for-collaborators (accessed May 12, 2020).

20 The President's Emergency Plan for AIDS Relief (PEPFAR). Dashboards. PEPFAR Panorama Spotlight. https://data.pepfar.gov/dashboards (accessed May 12, 2020).

21 Rue H. The R-INLA project. http://www.r-inla.org/ (accessed May 12, 2020).

22 Riebler A, Sørbye SH, Simpson D, Rue H. An intuitive Bayesian spatial model for disease mapping that accounts for scaling. *arXiv:160101180 [stat]* 2016; published online Jan 6. http://arxiv.org/abs/1601.01180 (accessed May 12, 2020).

23 Fuglstad G-A, Simpson D, Lindgren F, Rue H. Constructing Priors that Penalize the Complexity of Gaussian Random Fields. *arXiv:150300256 [stat]* 2017; published online Nov 27. http://arxiv.org/abs/1503.00256 (accessed May 12, 2020).

24 Tatem AJ. WorldPop, open data for spatial demography. *Scientific Data* 2017; **4**: 170004.

25 Land Processes Distributed Active Archive Center. Combined MODIS 5.1. MCD12Q1 | LP DAAC :: NASA Land Data Products and Services (accessed June 1, 2017)

26 World Wildlife Fund. Global Lakes and Wetlands Database, Level 3. 2004. https://www.worldwildlife.org/pages/global-lakes-and-wetlands-database (accessed June 1, 2017).

27 Lehner B, Döll P. Development and validation of a global database of lakes, reservoirs and wetlands. *Journal of Hydrology* 2004; **296**: 1–22.

28 University of Oxford, Big Data Institute. Data Directory. Malaria Atlas Project (MAP). https://malariaatlas.org/data-directory/#Input (accessed May 12, 2020).
